# Supplementary material for: Network Pharmacology and Molecular Docking Analysis of Active Compounds in Tualang Honey against Atherosclerosis
Source: Foods. 2023 Apr 25;12(9):1779. doi: 10.3390/foods12091779 (PMC10178747; doi:10.3390/foods12091779)
Supplement: Supplementary file 1 [file foods-12-01779-s001.zip › foods-2298264-supplementary.pdf]

Supplementary Materials

Network Pharmacology and Molecular Docking Analysis of Active Compounds in Tualang Honey against Atherosclerosis

Ain Nabila Syahira Shamsol Azman [1], Jun Jie Tan [2], Muhammad Nazrul Hakim Abdullah [3], Hasnah Bahari [1] Vuanghao Lim [2,*] and Yoke Keong Yong [1,*]

1 Department of Human Anatomy, Faculty of Medicine and Health Sciences, Universiti Putra Malaysia, Serdang 43400, Selangor, Malaysia
2 Advanced Medical and Dental Institute, Universiti Sains Malaysia, Bertam, Kepala Batas 13200, Penang, Malaysia
3 Department of Biomedical Science, Faculty of Medicine and Health Sciences, Universiti Putra Malaysia, Serdang 43400, Selangor, Malaysia
* Correspondence: yoke_keong@upm.edu.my (V.L.); vlim@usm.my (Y.K.Y.)

Supplementary Table S1: Bioactive compound of Tualang Honey
Supplementary Table S2: Target genes of Tualang Honey Bioactive Compound
Supplementary Table S3: Target genes of Atherosclerosis
Supplementary Table S4: The degree value of nodes in protein–protein interaction network.

Supplementary Table S1: Bioactive compound of Tualang Honey

No	Compound	References
1	(5-methylfuran-2-yl) methanol	[44]
2	1,6-Anhydro-beta-D-glucofuranose	[39]
3	1-dodecane	[42]
4	1-hexadecane	[42]
5	2 furancarboxaldehyde	[39,41,42]
6	2(3H)-Furanone	[39,41,42]
7	2(5H)-Furanone	[39,41,42,44]
8	2, 3-dihydro-3, 5-hydroxy-6-methyl-4H-pyran-4one	[39,42,44]
9	2,4-dihydroxy-2,5-dimethyl-3[2H]-furan-3 one	[41,42,44]
10	2-acetylfuran	[44]
11	2-amino-1,3-dihydroxyactadecane	[40]
12	2-cyclopentene-1,4,-dione	[41,42]
13	2-Furanmethanol	[39]
14	2-furoic acid	[44]
15	2-furylmethylketone	[41]
16	2-hydroxy-1-[hydroxymethyl] ethyl ester	[41]
17	2-hydroxy-2-cyclopenten-1-one	[41,42,44]
18	2-hydroxycinnamic acid	[43]
19	2-Propanone, 1,3-dihydroxy	[39]
20	2-Propanone, 1-hydroxy- (CAS) acetol	[39]
21	3,5-Dihydroxy-2-methyl-4H-pyran-4-one	[44]
22	3,7,4-trihydroxyflavone	[40]
23	3-Furaldehyde	[39]
24	3-furoic acid methyl ester	[44]
25	3-Hydroxy-2-methyl-4H-pyran-4-one	[44]
26	4-oxo-5-methoxy-2-penten-5-olide	[44]
27	5-(HydroxymethyI) 2-furancarboxaldehyde	[39]
28	5-(hydroxymethyl)-furfural(HMF)	[41,42,44]
29	5,5′-oxy-dimethylene-bis[2-furaldehyde]	[44]
30	5-acetoxymethyl-2-furaldehyde	[44]
31	6-hydroxy-4-methylcoumarin	[40]
32	9-octadecane	[42]
33	Abscisic acid	[40]
34	Acetic acid	[41,42]
35	Apigenin	[13,40,43]
36	Benzoic acid	[13,41]
37	Caffeic acid	[13,40,41,43]
38	Catechin	[13,41,43]
39	Chrysin	[40,43]
40	Cycloeicosane	[42]
41	Cyclotetradecane	[42]
42	Dioctyl phthalate	[42]

43	Docosane	[42]
44	Dodecane	[42]
45	Dotriacontane	[42]
46	Eicosane	[42]
47	Ethyl oleate	[41]
48	ethyl palmitate	[44]
49	Fisetin	[40]
50	Formic acid	[39]
51	Furfural	[41]
52	Gallic acid	[13,41,43]
53	Gamma-crotonolactone	[41]
54	Gluconic acid	[40]
55	Glucose	[40]
56	Heneicosane	[42]
57	Heptacosane	[42]
58	Hesperetin	[13,40]
59	Hexacosane	[42]
60	Hyacinthin	[41,42,44]
61	Hydrogen chloride	[39]
62	Hydroxybenzoic acid	[40]
63	Hydroxyoctanic acid	[40]
64	Isoorientin	[40]
65	Kaempferol	[13,40,41,43]
66	Ketoisophorone	[44]
67	Lauryl diethanolamide	[40]
68	Levoglucosenone	[39]
69	Linalool oxide	[42]
70	Linoleic acid	[44,40,40]
71	Luteolin	[13,41,40]
72	Maltol	[39]
73	Methyl 2-furoate	[39]
74	Naringenin	[13,40,41]
75	Naringin	[13]
76	Nonacosane	[42]
77	n-tetratetracontane	[42]
78	Octacosane	[42]
79	Octadecanoic acid	[41,44]
80	Oleic acid	[41,44]
81	Orotic acid	[40]
82	Palmitic acid	[41,42,44]
83	p-coumaric acid	[13,41,43]
84	Pentacosane	[42]
85	Phenylacetaldehyde	[39,41]
86	Propanoic acid	[39]
87	Protoanemonine	[44]

88	Pyridoxal	[40]
89	Pyr-idoxamine	[40]
90	Pyrocatechol	[44]
91	Quercetin-3-0-rutinoside	[43]
92	Salicylic acid	[40]
93	Stearic acid	[42,44]
94	Succinic acid	[40]
95	Syringic acid	[13,41,43]
96	Tetracosane	[42]
97	Trans-cinnamic acid	[13,41,43]
98	Triacontane	[42]
99	Tricosane	[42]
100	Tritetracontane	[42]
101	Vanillic acid	[13]
102	Vitexin	[40]
103	Xanthohumol	[40]

Supplementary Table S2: Target genes of Tualang Honey Bioactive Compound

No	Target	Common name	Compound	Database 1	Database 2
1.	P-glycoprotein 1	ABCB1	fisetin, hesperetin, kaempferol, luteolin	Swiss Target	
2.	Multidrug resistance-associated protein 1	ABCC1	ethyl oleate, fisetin, hesperetin, kaempferol, luteolin	Swiss Target	SuperPred
3.	ATP-binding cassette sub-family G member 2	ABCG2	fisetin, hesperetin, kaempferol, luteolin	Swiss Target	
4.	Acetyl-CoA carboxylase 1	ACACA	ethyl oleate	Swiss Target	
5.	Acetyl-CoA carboxylase 2	ACACB	ethyl oleate	Swiss Target	
6.	Acetylcholinesterase	ACHE	fisetin, hesperetin, kaempferol, luteolin	Swiss Target	
7.	Low molecular weight phosphotyrosine protein phosphatase	ACP1	ethyl oleate	Swiss Target	
8.	ADAM Metallopeptidase Domain 10	ADAM10	ethyl oleate		SuperPred
9.	Adenosine A1 receptor	ADORA1	fisetin, hesperetin, kaempferol, luteolin	Swiss Target	
10.	Adenosine A2a receptor	ADORA2A	fisetin, kaempferol, luteolin	Swiss Target	
11.	Adenosine A3 receptor	ADORA3	hesperetin	Swiss Target	
12.	Adenosylhomocysteinase	AHCY	ethyl oleate		SuperPred
13.	Aryl hydrocarbon receptor	AHR	fisetin, kaempferol, luteolin	Swiss Target	
14.	Aldehyde reductase	AKR1A1	fisetin, kaempferol, luteolin	Swiss Target	
15.	Aldose reductase	AKR1B1	fisetin, hesperetin, kaempferol, luteolin	Swiss Target	
16.	Aldo-keto reductase family 1 member B10	AKR1B10	ethyl oleate, fisetin, kaempferol, luteolin	Swiss Target	
17.	Aldo-keto reductase family 1 member C1	AKR1C1	ethyl oleate, fisetin, kaempferol, luteolin	Swiss Target	
18.	Aldo-keto reductase family 1 member C2	AKR1C2	ethyl oleate, fisetin, kaempferol, luteolin	Swiss Target	SuperPred
19.	Aldo-keto-reductase family 1 member C3	AKR1C3	ethyl oleate, fisetin, hesperetin, kaempferol, luteolin	Swiss Target	
20.	Aldo-keto-reductase family 1 member C4	AKR1C4	kaempferol, luteolin	Swiss Target	
21.	Serine/threonine-protein kinase AKT	AKT1	fisetin, kaempferol, luteolin	Swiss Target	
22.	ALK tyrosine kinase receptor	ALK	kaempferol, luteolin	Swiss Target	
23.	Arachidonate 12-lipoxygenase	ALOX12	fisetin, hesperetin, kaempferol, luteolin	Swiss Target	

24.	Arachidonate 15-lipoxygenase	ALOX15	fisetin, kaempferol, luteolin	Swiss Target	
25.	Arachidonate 5-lipoxygenase	ALOX5	ethyl oleate, fisetin, kaempferol, luteolin	Swiss Target	SuperPred
26.	Alkaline phosphatase, tissue-nonspecific isozyme	ALPL	catechin, ethyl oleate	Swiss Target	SuperPred
27.	Alpha-Amylase 1A	AMY1A	kaempferol, luteolin	Swiss Target	
28.	Aminopeptidase N	ANPEP	ethyl oleate		SuperPred
29.	Amine oxidase, copper containing	AOC3	ethyl oleate		SuperPred
30.	DNA-(apurinic or apyrimidinic site) lyase	APEX1	ethyl oleate, fisetin	Swiss Target	SuperPred
31.	APH1A Gamma Secretase Subunit	APH1A	ethyl oleate	Swiss Target	
32.	APH1B Gamma Secretase Subunit	APH1B	ethyl oleate	Swiss Target	
33.	Beta amyloid A4 protein	APP	ethyl oleate, fisetin, hesperetin, kaempferol, luteolin	Swiss Target	
34.	Androgen Receptor	AR	ethyl oleate, luteolin	Swiss Target	SuperPred
35.	Arginase-1	ARG1	fisetin, kaempferol, luteolin	Swiss Target	
36.	Potassium-transporting ATPase alpha chain 2	ATP12A	ethyl oleate	Swiss Target	
37.	Potassium-transporting ATPase	ATP4A	hesperetin	Swiss Target	
38.	Potassium-transporting ATPase	ATP4B	hesperetin	Swiss Target	
39.	Serine/threonine-protein kinase Aurora-A	AURKA	hesperetin	Swiss Target	
40.	Aurora kinase B/Inner centromere protein	AURKB	ethyl oleate, fisetin, kaempferol, luteolin	Swiss Target	SuperPred
41.	Vasopressin V2 receptor	AVPR2	fisetin, kaempferol, luteolin	Swiss Target	
42.	Tyrosine-protein kinase receptor UFO	AXL	fisetin, kaempferol, luteolin	Swiss Target	
43.	Beta-secretase 1	BACE1	fisetin, hesperetin, kaempferol, luteolin	Swiss Target	
44.	Apoptosis regulator Bcl-2	BCL2	hesperetin	Swiss Target	
45.	C5a anaphylatoxin chemotactic receptor	C5AR1	ethyl oleate		SuperPred
46.	Carbonic anhydrase I	CA1	fisetin, hesperetin, kaempferol, luteolin	Swiss Target	
47.	Carbonic anhydrase XII	CA12	fisetin, hesperetin, kaempferol, luteolin	Swiss Target	SuperPred
48.	Carbonic anhydrase XIII	CA13	fisetin, hesperetin, kaempferol, luteolin	Swiss Target	
49.	Carbonic anhydrase XIV	CA14	fisetin, kaempferol, luteolin	Swiss Target	
50.	Carbonic anhydrase II	CA2	fisetin, hesperetin, kaempferol, luteolin	Swiss Target	
51.	Carbonic anhydrase III	CA3	ethyl oleate, fisetin, hesperetin, kaempferol, luteolin	Swiss Target	SuperPred

52.	Carbonic anhydrase IV	CA4	fisetin, hesperetin, kaempferol, luteolin	Swiss Target	SuperPred
53.	Carbonic anhydrase VA	CA5A	fisetin, hesperetin, kaempferol, luteolin	Swiss Target	
54.	Carbonic anhydrase VB	CA5B	hesperetin	Swiss Target	
55.	Carbonic anhydrase VI	CA6	fisetin, hesperetin, kaempferol, luteolin	Swiss Target	
56.	Carbonic anhydrase VII	CA7	catechin, fisetin, hesperetin, kaempferol, luteolin	Swiss Target	SuperPred
57.	Carbonic anhydrase IX	CA9	fisetin, hesperetin, kaempferol, luteolin	Swiss Target	
58.	Voltage-gated L-type calcium channel alpha-1C subunit	CACNA1C	ethyl oleate		SuperPred
59.	Calcitonin gene-related peptide 1	CALCA	ethyl oleate	Swiss Target	
60.	CaM kinase II beta	CAMK2B	fisetin, hesperetin, kaempferol, luteolin	Swiss Target	
61.	Calpain 1	CAPN1	ethyl oleate	Swiss Target	SuperPred
62.	Carbonyl reductase [NADPH] 1	CBR1	hesperetin	Swiss Target	
63.	Cyclin-dependent kinase 2/cyclin A	CCNA1	hesperetin	Swiss Target	
64.	Cyclin-dependent kinase 2/cyclin A	CCNA2	hesperetin	Swiss Target	
65.	Cyclin-dependent kinase 1/cyclin B	CCNB1	fisetin, hesperetin	Swiss Target	
66.	Cyclin-dependent kinase 1/cyclin B	CCNB2	fisetin, kaempferol, luteolin	Swiss Target	
67.	Cyclin-dependent kinase 1/cyclin B	CCNB3	fisetin, kaempferol, luteolin	Swiss Target	
68.	Cyclin-dependent kinase 2/cyclin E1	CCNE1	hesperetin	Swiss Target	
69.	Cyclin T1	CCNT1	ethyl oleate	Swiss Target	
70.	Lymphocyte differentiation antigen CD38	CD38	ethyl oleate		SuperPred
71.	CD81 antigen	CD81	ethyl oleate	Swiss Target	
72.	Cyclin-dependent kinase 1/cyclin B1	CDK1	ethyl oleate, fisetin, hesperetin, kaempferol, luteolin	Swiss Target	SuperPred
73.	Cyclin-dependent kinase 2/cyclin A	CDK2	hesperetin	Swiss Target	
74.	Cyclin-dependent kinase 5/CDK5 activator 1	CDK5	ethyl oleate, fisetin, kaempferol, luteolin	Swiss Target	SuperPred
75.	Cyclin-dependent kinase 5/CDK5 activator 1	CDK5R1	fisetin, kaempferol, luteolin	Swiss Target	
76.	Cyclin-dependent kinase 6	CDK6	fisentin, kaempferol, luteolin	Swiss Target	SuperPred
77.	Acyl coenzyme A: cholesterol acyltransferase	CES1	ethyl oleate, hesperetin	Swiss Target	SuperPred
78.	Carboxylesterase 2	CES2	ethyl oleate, hesperetin	Swiss Target	SuperPred
79.	Cholesteryl ester transfer protein	CETP	ethyl oleate	Swiss Target	

80.	Cystic fibrosis transmembrane conductance regulator	CFTR	kaempferol, luteolin	Swiss Target	
81.	Serine/threonine-protein kinase Chk1	CHEK1	hesperetin	Swiss Target	
82.	Neuronal acetylcholine receptor protein alpha-7 subunit	CHRNA7	hesperetin	Swiss Target	
83.	Neuronal acetylcholine receptor; alpha4/beta4	CHRNB4	ethyl oleate		SuperPred
84.	CDGSH iron-sulfur domain-containing protein 1	CISD1	kaempferol		SuperPred
85.	Dual specificity protein kinase CLK4	CLK4	ethyl oleate		SuperPred
86.	Cannabinoid receptor 1	CNR1	ethyl oleate	Swiss Target	
87.	Cannabinoid CB2 receptor	CNR2	ethyl oleate	Swiss Target	SuperPred
88.	Carnitine palmitoyltransferase 2	CPT2	ethyl oleate		SuperPred
89.	Casein kinase II alpha	CSNK2A1	fisetin, kaempferol, luteolin	Swiss Target	SuperPred
90.	Casein kinase II alpha/beta	CSNK2B	ethyl oleate		SuperPred
91.	Chymotrypsin C	CTRC	ethyl oleate	Swiss Target	
92.	Cathepsin D	CTSD	ethyl oleate		SuperPred
93.	Interleukin-8 receptor A	CXCR1	fisetin, kaempferol, luteolin	Swiss Target	
94.	Cytochrome P450 17A1	CYP17A1	ethyl oleate	Swiss Target	
95.	Cytochrome P450 19A1	CYP19A1	ethyl oleate, fisetin, hesperetin, kaempferol, luteolin	Swiss Target	
96.	Cytochrome P450 1B1	CYP1B1	fisetin, hesperetin, kaempferol, luteolin	Swiss Target	SuperPred
97.	Cytochrome P450 2A6	CYP2A6	ethyl oleate		SuperPred
98.	Cytochrome P450 2C19	CYP2C19	ethyl oleate	Swiss Target	
99.	Cytochrome P450 3A4	CYP3A4	ethyl oleate		SuperPred
100.	Cytochrome P450 51	CYP51A1	ethyl oleate	Swiss Target	
101.	Cysteinyl leukotriene receptor 2	CYSLTR2	ethyl oleate		SuperPred
102.	Death-associated protein kinase 1	DAPK1	fisetin, kaempferol, luteolin	Swiss Target	
103.	Deoxycytidine kinase	DCK	ethyl oleate	Swiss Target	
104.	dCTP pyrophosphatase 1	DCTPP1	ethyl oleate		SuperPred
105.	Dihydrofolate reductase	DHFR	ethyl oleate		SuperPred
106.	Dynamin-1	DNM1	hesperetin	Swiss Target	

107.	DNA (cytosine-5)-methyltransferase 1	DNMT1	hesperetin	Swiss Target	
108.	Dipeptidyl peptidase IV	DPP4	hesperetin, kaempferol, luteolin		SuperPred
109.	Dipeptidyl peptidase VIII	DPP8	ethyl oleate		SuperPred
110.	Dipeptidyl peptidase IX	DPP9	ethyl oleate		SuperPred
111.	Dopamine D4 receptor	DRD4	fisetin, kaempferol, luteolin	Swiss Target	
112.	Dual specificity protein phosphatase 3	DUSP3	hesperetin	Swiss Target	SuperPred
113.	Dual-specificity tyrosine-phosphorylation regulated kinase 1A	DYRK1A	hesperetin	Swiss Target	
114.	Endothelin receptor ET-A	EDNRA	hesperetin	Swiss Target	
115.	Epidermal growth factor receptor erbB1	EGFR	fisetin, kaempferol, luteolin	Swiss Target	
116.	Endothelial PAS domain-containing protein 1	EPAS1	ethyl oleate	Swiss Target	
117.	Epoxide hydrolase 1	EPHX1	ethyl oleate	Swiss Target	SuperPred
118.	Endoplasmic reticulum aminopeptidase 2	ERAP2	ethyl oleate	Swiss Target	SuperPred
119.	Serine/threonine-protein kinase/endoribonuclease IRE1	ERN1	hesperetin	Swiss Target	
120.	Estrogen receptor alpha	ESR1	hesperetin, kaempferol, luteolin	Swiss Target	
121.	Estrogen receptor beta	ESR2		Swiss Target	
122.	Estrogen-related receptor alpha	ESRRA	fisetin, kaempferol, luteolin	Swiss Target	
123.	Thrombin and coagulation factor X	F10	ethyl oleate	Swiss Target	
124.	Thrombin	F2	ethyl oleate, fisetin, kaempferol, luteolin	Swiss Target	
125.	Proteinase-activated receptor 1	F2R	ethyl oleate	Swiss Target	
126.	Anandamide amidohydrolase	FAAH	ethyl oleate	Swiss Target	
127.	Fatty acid-binding protein 1 (liver)	FABP1	ethyl oleate	Swiss Target	
128.	Fatty acid-binding protein 3 (muscle and heart)	FABP3	ethyl oleate	Swiss Target	
129.	Fatty acid-binding protein 4 (adipocyte)	FABP4	ethyl oleate	Swiss Target	
130.	Fatty acid-binding protein 5 (epidermal)	FABP5	ethyl oleate	Swiss Target	
131.	Free fatty acid receptor 1	FFAR1	ethyl oleate, hesperetin	Swiss Target	
132.	Tyrosine-protein kinase FGR	FGR	ethyl oleate		SuperPred
133.	Vascular endothelial growth factor receptor 1	FLT1	ethyl oleate	Swiss Target	SuperPred

134.	Tyrosine-protein kinase receptor FLT3	FLT3	fisetin, kaempferol, luteolin	Swiss Target	SuperPred
135.	Protein farnesyltransferase	FNTA	ethyl oleate	Swiss Target	
136.	Protein farnesyltransferase	FNTB	ethyl oleate	Swiss Target	
137.	Formyl peptide receptor 1	FPR1	ethyl oleate		SuperPred
138.	Lipoxin A4 receptor	FPR2	ethyl oleate		SuperPred
139.	Fucosyltransferase 4	FUT4	hesperetin	Swiss Target	
140.	Alpha-(1,3)-fucosyltransferase 7	FUT7	hesperetin	Swiss Target	
141.	Glucose-6-phosphate 1-dehydrogenase	G6PD	ethyl oleate	Swiss Target	
142.	Geranylgeranyl pyrophosphate synthetase	GGPS1	ethyl oleate		SuperPred
143.	Glyoxalase I	GLO1	fisetin, kaempferol, luteolin	Swiss Target	
144.	Glycine receptor subunit alpha-1	GLRA1	ethyl oleate		SuperPred
145.	Gonadotropin-releasing hormone receptor	GNRHR	ethyl oleate	Swiss Target	
146.	G-protein coupled bile acid receptor 1	GPBAR1	ethyl oleate		SuperPred
147.	Glucose-dependent insulinotropic receptor	GPR119	ethyl oleate	Swiss Target	
148.	Uracil nucleotide/cysteinyl leukotriene receptor	GPR17	ethyl oleate		SuperPred
149.	G-protein coupled receptor 35	GPR35	ethyl oleate, fisetin, kaempferol, luteolin	Swiss Target	SuperPred
150.	Glutamate NMDA receptor; GRIN1/GRIN2B	GRIN1	ethyl oleate		SuperPred
151.	G protein-coupled receptor kinase 6	GRK6	kaempferol, luteolin	Swiss Target	
152.	Metabotropic glutamate receptor 2	GRM2	hesperetin	Swiss Target	
153.	Metabotropic glutamate receptor 5	GRM5	hesperetin	Swiss Target	
154.	Glycogen synthase kinase-3 beta	GSK3B	fisetin, kaempferol, luteolin	Swiss Target	
155.	Beta-glucuronidase	GUSB	ethyl oleate, hesperetin	Swiss Target	SuperPred
156.	Histone deacetylase 10	HDAC10	ethyl oleate		SuperPred
157.	Histone deacetylase 3	HDAC3	ethyl oleate		SuperPred
158.	Hypoxia-inducible factor 1 alpha	HIF1A	luteolin	Swiss Target	SuperPred
159.	HMG-CoA reductase	HMGCR	ethyl oleate	Swiss Target	
160.	Histamine H4 receptor	HRH4	ethyl oleate	Swiss Target	
161.	11-beta-hydroxysteroid dehydrogenase 1	HSD11B1	ethyl oleate	Swiss Target	
162.	11-beta-hydroxysteroid dehydrogenase 2	HSD11B2	ethyl oleate	Swiss Target	

163.	Estradiol 17-beta-dehydrogenase 1	HSD17B1	fisetin, hesperetin, kaempferol, luteolin	Swiss Target	
164.	Estradiol 17-beta-dehydrogenase 2	HSD17B2	ethyl oleate, fisetin, hesperetin, kaempferol, luteolin	Swiss Target	
165.	Estradiol 17-beta-dehydrogenase 3	HSD17B3	hesperetin	Swiss Target	
166.	Serotonin 2c (5-HT2c) receptor	HTR2C	ethyl oleate		SuperPred
167.	Insulin-like growth factor I receptor	IGF1R	fisetin, hesperetin, kaempferol, luteolin	Swiss Target	
168.	Insulin receptor	INSR	fisetin	Swiss Target	
169.	Histone acetyltransferase KAT6A	KAT6A	ethyl oleate		SuperPred
170.	HERG	KCNH2	ethyl oleate		SuperPred
171.	LSD1/CoREST complex	KDM1A	ethyl oleate		SuperPred
172.	Lysine-specific demethylase 4D-like	KDM4E	fisetin, luteolin	Swiss Target	
173.	Vascular endothelial growth factor receptor 2	KDR	fisetin, hesperetin, kaempferol, luteolin	Swiss Target	
174.	Kelch-like ECH-associated protein 1	KEAP1	ethyl oleate		SuperPred
175.	Stem cell growth factor receptor	KIT	hesperetin	Swiss Target	
176.	Kruppel-like factor 5	KLF5	ethyl oleate		SuperPred
177.	Kallikrein 1	KLK1	hesperetin	Swiss Target	
178.	Kallikrein 2	KLK2	hesperetin	Swiss Target	
179.	Kallikrein 5	KLK5	ethyl oleate		SuperPred
180.	Kallikrein 7	KLK7	ethyl oleate		SuperPred
181.	LIM domain kinase 1	LIMK1	ethyl oleate	Swiss Target	
182.	LIM domain kinase 2	LIMK2	ethyl oleate	Swiss Target	
183.	Cystinyl aminopeptidase	LNPEP	ethyl oleate		SuperPred
184.	Leukotriene B4 receptor 1	LTB4R	ethyl oleate	Swiss Target	
185.	Acyl-protein thioesterase 1	LYPLA1	ethyl oleate	Swiss Target	
186.	Acyl-protein thioesterase 2	LYPLA2	ethyl oleate	Swiss Target	
187.	Monoamine oxidase A	MAOA	fisetin, kaempferol, luteolin	Swiss Target	
188.	Monoamine oxidase B	MAOB	hesperetin	Swiss Target	
189.	Dual specificity mitogen-activated protein kinase kinase 2	MAP2K2	ethyl oleate		SuperPred

190.	Mitogen-activated protein kinase kinase kinase kinase 4	MAP4K4	hesperetin	Swiss Target	
191.	c-Jun N-terminal kinase 1	MAPK8	ethyl oleate	Swiss Target	
192.	c-Jun N-terminal kinase 2	MAPK9	ethyl oleate	Swiss Target	SuperPred
193.	MAP kinase-activated protein kinase 2	MAPKAPK 2	hesperetin	Swiss Target	
194.	Microtubule-associated protein tau	MAPT	fisetin, kaempferol	Swiss Target	
195.	Melanin-concentrating hormone receptor 1	MCHR1	ethyl oleate	Swiss Target	
196.	Hepatocyte growth factor receptor	MET	fisetin, hesperetin, kaempferol, luteolin	Swiss Target	
197.	Methionine aminopeptidase 2	METAP2	ethyl oleate		SuperPred
198.	Monoglyceride lipase	MGLL	ethyl oleate	Swiss Target	
199.	Macrophage migration inhibitory factor	MIF	ethyl oleate		SuperPred
200.	Matrix metalloproteinase 12	MMP12	fisetin, hesperetin, kaempferol, luteolin	Swiss Target	SuperPred
201.	Matrix metalloproteinase 13	MMP13	fisetin, hesperetin, kaempferol, luteolin	Swiss Target	
202.	Matrix metalloproteinase 2	MMP2	fisetin, hesperetin, kaempferol, luteolin	Swiss Target	
203.	Matrix metalloproteinase 3	MMP3	ethyl oleate, fisetin, hesperetin, kaempferol, luteolin	Swiss Target	
204.	Matrix metalloproteinase 8	MMP8	ethyl oleate, hesperetin	Swiss Target	SuperPred
205.	Matrix metalloproteinase 9	MMP9	fisetin, hesperetin, kaempferol, luteolin	Swiss Target	
206.	DNA-3-methyladenine glycosylase	MPG	fisetin, kaempferol	Swiss Target	
207.	Myeloperoxidase	MPO	fisetin, kaempferol, luteolin	Swiss Target	
208.	Myosin light chain kinase, smooth muscle	MYLK	fisetin	Swiss Target	
209.	N-acylsphingosine-amidohydrolase	NAAA	ethyl oleate	Swiss Target	
210.	Nicotinamide phosphoribosyltransferase	NAMPT	ethyl oleate		SuperPred
211.	Gamma-secretase	NCSTN	ethyl oleate	Swiss Target	
212.	Serine/threonine-protein kinase NEK2	NEK2	fisetin, kaempferol, luteolin	Swiss Target	
213.	Serine/threonine-protein kinase NEK6	NEK6	fisetin, kaempferol, luteolin	Swiss Target	
214.	Nuclear factor erythroid 2-related factor 2	NFE2L2	ethyl oleate		SuperPred
215.	Nuclear factor NF-kappa-B p105 subunit	NFKB1	catechin, ethyl oleate		SuperPred

216.	NACHT, LRR and PYD domains-containing protein 3	NLRP3	ethyl oleate	Swiss Target	
217.	Nitric-oxide synthase, brain	NOS1	ethyl oleate	Swiss Target	
218.	Nitric oxide synthase, inducible	NOS2	ethyl oleate	Swiss Target	SuperPred
219.	NADPH oxidase 4	NOX4	fisetin, hesperetin, kaempferol, luteolin	Swiss Target	
220.	Quinone reductase 2	NQO2	ethyl oleate		SuperPred
221.	LXR-beta	NR1H2	ethyl oleate	Swiss Target	
222.	LXR-alpha	NR1H3	ethyl oleate	Swiss Target	
223.	Pregnane X receptor	NR1I2	ethyl oleate		SuperPred
224.	Photoreceptor-specific nuclear receptor	NR2E3	ethyl oleate		SuperPred
225.	Glucocorticoid receptor	NR3C1	ethyl oleate	Swiss Target	
226.	Mineralocorticoid receptor	NR3C2	ethyl oleate	Swiss Target	
227.	Nuclear receptor subfamily 4 group A member 1	NR4A1	ethyl oleate		SuperPred
228.	Nerve growth factor receptor Trk-A	NTRK1	hesperetin	Swiss Target	
229.	NT-3 growth factor receptor	NTRK3	ethyl oleate		SuperPred
230.	Neurotensin receptor 2	NTSR2	ethyl oleate		SuperPred
231.	NUAK family SNF1-like kinase 1	NUAK1	fisetin, kaempferol, luteolin	Swiss Target	
232.	Ornithine decarboxylase	ODC1	hesperetin	Swiss Target	
233.	Purinergic receptor P2Y12	P2RY12	ethyl oleate	Swiss Target	SuperPred
234.	Poly [ADP-ribose] polymerase-1	PARP1	fisetin, hesperetin, kaempferol, luteolin	Swiss Target	
235.	Phosphodiesterase 10A	PDE10A	ethyl oleate	Swiss Target	
236.	Phosphodiesterase 3B	PDE3B	ethyl oleate		SuperPred
237.	Platelet-derived growth factor receptor alpha	PDGFRA	ethyl oleate		SuperPred
238.	Platelet-derived growth factor receptor beta	PDGFRB	ethyl oleate, hesperetin	Swiss Target	SuperPred
239.	6-phosphofructo-2-kinase/fructose-2,6-bisphosphatase 3	PFKFB3	ethyl oleate	Swiss Target	
240.	6-phosphogluconate dehydrogenase	PGD	hesperetin	Swiss Target	
241.	Progesterone receptor	PGR	ethyl oleate	Swiss Target	
242.	PI3-kinase p110-alpha subunit	PIK3CA	ethyl oleate		SuperPred

243.	PI3-kinase p110-beta subunit	PIK3CB	ethyl oleate		SuperPred
244.	PI3-kinase p110-delta subunit	PIK3CD	ethyl oleate	Swiss Target	SuperPred
245.	PI3-kinase p110-gamma subunit	PIK3CG	ethyl oleate, fisetin	Swiss Target	SuperPred
246.	PI3-kinase p110-alpha/p85-alpha	PIK3R1	ethyl oleate, fisetin, kaempferol, luteolin	Swiss Target	SuperPred
247.	Serine/threonine-protein kinase PIM1	PIM1	fisetin, hesperetin	Swiss Target	
248.	Serine/threonine-protein kinase PIM2	PIM2	hesperetin	Swiss Target	
249.	Serine/threonine-protein kinase PIM3	PIM3	hesperetin	Swiss Target	
250.	Peptidyl-prolyl cis-trans isomerase NIMA-interacting 1	PIN1	ethyl oleate	Swiss Target	
251.	Protein kinase N1	PKN1	fisetin, kaempferol, luteolin	Swiss Target	
252.	Group X secretory phospholipase A2	PLA2G10	hesperetin	Swiss Target	
253.	Phospholipase A2 group 1B	PLA2G1B	ethyl oleate, fisetin, hesperetin, kaempferol, luteolin	Swiss Target	
254.	Phospholipase A2 group IIA	PLA2G2A	ethyl oleate, hesperetin	Swiss Target	SuperPred
255.	Cytosolic phospholipase A2	PLA2G4A	ethyl oleate	Swiss Target	
256.	Phospholipase A2 group V	PLA2G5	hesperetin	Swiss Target	
257.	Plasminogen	PLG	hesperetin, luteolin	Swiss Target	
258.	Serine/threonine-protein kinase PLK1	PLK1	fisetin, kaempferol, luteolin	Swiss Target	
259.	Purine nucleoside phosphorylase	PNP	hesperetin	Swiss Target	
260.	DNA polymerase alpha subunit	POLA1	ethyl oleate	Swiss Target	
261.	DNA polymerase beta	POLB	hesperetin	Swiss Target	
262.	Peroxisome proliferator-activated receptor alpha	PPARA	ethyl oleate	Swiss Target	
263.	Peroxisome proliferator-activated receptor delta	PPARD	ethyl oleate	Swiss Target	
264.	Peroxisome proliferator-activated receptor gamma	PPARG	ethyl oleate, hesperetin	Swiss Target	
265.	Protein kinase C alpha	PRKCA	ethyl oleate	Swiss Target	SuperPred
266.	Protein kinase C delta	PRKCD	ethyl oleate	Swiss Target	SuperPred
267.	Protein kinase C eta	PRKCH	ethyl oleate	Swiss Target	
268.	Protein kinase C theta	PRKCQ	ethyl oleate	Swiss Target	
269.	Protein-arginine N-methyltransferase 1	PRMT1	hesperetin	Swiss Target	

270.	Presenilin 1	PSEN1	ethyl oleate	Swiss Target	
271.	Presenilin 2	PSEN2	ethyl oleate	Swiss Target	
272.	Presenilin Enhancer, Gamma-Secretase Subunit	PSENEN	ethyl oleate	Swiss Target	
273.	Proteasome subunit beta type-9	PSMB9	ethyl oleate		SuperPred
274.	Prostanoid EP1 receptor	PTGER1	ethyl oleate		SuperPred
275.	Prostanoid EP2 receptor	PTGER2	ethyl oleate	Swiss Target	
276.	Prostaglandin E synthase	PTGES	ethyl oleate	Swiss Target	
277.	Prostanoid IP receptor	PTGIR	ethyl oleate	Swiss Target	
278.	Cyclooxygenase-1	PTGS1	ethyl oleate, hesperetin	Swiss Target	SuperPred
279.	Cyclooxygenase-2	PTGS2	ethyl oleate, kaempferol, luteolin	Swiss Target	SuperPred
280.	Focal adhesion kinase 1	PTK2	fisetin, kaempferol, luteolin	Swiss Target	
281.	Protein-tyrosine phosphatase 1B	PTPN1	ethyl oleate	Swiss Target	SuperPred
282.	Protein-tyrosine phosphatase 2C	PTPN11	ethyl oleate		SuperPred
283.	Protein-tyrosine phosphatase 1E	PTPN13	ethyl oleate	Swiss Target	
284.	T-cell protein-tyrosine phosphatase	PTPN2	ethyl oleate	Swiss Target	SuperPred
285.	Protein-tyrosine phosphatase 1C	PTPN6	ethyl oleate	Swiss Target	
286.	Leukocyte common antigen	PTPRC	ethyl oleate	Swiss Target	
287.	Receptor-type tyrosine-protein phosphatase F (LAR)	PTPRF	ethyl oleate	Swiss Target	
288.	Receptor-type tyrosine-protein phosphatase S	PTPRS	fisetin, kaempferol, luteolin	Swiss Target	
289.	Liver glycogen phosphorylase	PYGL	fisetin, kaempferol, luteolin	Swiss Target	
290.	Serine/threonine-protein kinase RAF	RAF1	ethyl oleate, hesperetin	Swiss Target	
291.	Rho-associated protein kinase 1	ROCK1	ethyl oleate	Swiss Target	
292.	Rho-associated protein kinase 2	ROCK2	ethyl oleate	Swiss Target	
293.	Nuclear receptor ROR-beta	RORB	ethyl oleate		SuperPred
294.	Ribosomal protein S6 kinase alpha 5	RPS6KA5	ethyl oleate		SuperPred
295.	Relaxin receptor 1	RXFP1	ethyl oleate		SuperPred
296.	Retinoid X receptor alpha	RXRA	hesperetin	Swiss Target	
297.	Retinoid X receptor beta	RXRB	ethyl oleate		SuperPred

298.	Sphingosine 1-phosphate receptor Edg-5	S1PR2	ethyl oleate		SuperPred
299.	Sphingosine 1-phosphate receptor Edg-3	S1PR3	ethyl oleate		SuperPred
300.	Sphingosine 1-phosphate receptor Edg-8	S1PR5	ethyl oleate		SuperPred
301.	Acyl-CoA desaturase	SCD	ethyl oleate	Swiss Target	
302.	Sodium channel protein type II alpha subunit	SCN2A	ethyl oleate		SuperPred
303.	Sodium channel protein type III alpha subunit	SCN3A	ethyl oleate		SuperPred
304.	Sodium channel protein type IV alpha subunit	SCN4A	ethyl oleate		SuperPred
305.	Corticosteroid binding globulin	SERPINA6	ethyl oleate	Swiss Target	
306.	Plasminogen activator inhibitor 1	SERPINE1	hesperetin	Swiss Target	SuperPred
307.	Testis-specific androgen-binding protein	SHBG	ethyl oleate, hesperetin	Swiss Target	
308.	Solute carrier family 22 member 12	SLC22A12	fisetin, kaempferol	Swiss Target	
309.	Solute carrier family 40 member 1	SLC40A1	ethyl oleate		SuperPred
310.	Sodium/glucose cotransporter 2	SLC5A2	hesperetin	Swiss Target	
311.	Glycine transporter 2	SLC6A5	ethyl oleate		SuperPred
312.	Sodium/hydrogen exchanger 1	SLC9A1	ethyl oleate		SuperPred
313.	Acyl coenzyme A: cholesterol acyltransferase 1	SOAT1	ethyl oleate	Swiss Target	
314.	Acyl coenzyme A: cholesterol acyltransferase 2	SOAT2	ethyl oleate	Swiss Target	
315.	Squalene monooxygenase	SQLE	hesperetin	Swiss Target	
316.	Tyrosine-protein kinase SRC	SRC	fisetin, hesperetin, kaempferol, luteolin	Swiss Target	
317.	CMP-N-acetylneuraminate-beta-1,4-galactoside alpha-2,3-sialyltransferase	ST3GAL3	hesperetin	Swiss Target	
318.	Signal transducer and activator of transcription 1-alpha/beta	STAT1	hesperetin	Swiss Target	SuperPred
319.	Tyrosine-protein kinase SYK	SYK	fisetin, kaempferol, luteolin	Swiss Target	
320.	Neurokinin 2 receptor	TACR2	ethyl oleate	Swiss Target	
321.	Neurokinin 3 receptor	TACR3	ethyl oleate	Swiss Target	
322.	Serine/threonine-protein kinase TAO1	TAOK1	ethyl oleate		SuperPred
323.	Serine/threonine-protein kinase TAO3	TAOK3	ethyl oleate		SuperPred
324.	Taste receptor type 2 member 31	TAS2R31	ethyl oleate, hesperetin	Swiss Target	

325.	Tyrosyl-DNA phosphodiesterase 1	TDP1	ethyl oleate		SuperPred
326.	Telomerase reverse transcriptase	TERT	ethyl oleate, fisetin, hesperetin, kaempferol, luteolin	Swiss Target	
327.	Toll-like receptor 4	TLR4	ethyl oleate		SuperPred
328.	Tankyrase-1	TNKS	fisetin, hesperetin, kaempferol, luteolin	Swiss Target	
329.	Tankyrase-2	TNKS2	fisetin, hesperetin, kaempferol, luteolin	Swiss Target	
330.	DNA topoisomerase I	TOP1	ethyl oleate, fisetin, hesperetin, kaempferol, luteolin	Swiss Target	SuperPred
331.	DNA topoisomerase II alpha	TOP2A	ethyl oleate, fisetin		
332.	Transcription intermediary factor 1-alpha	TRIM24	ethyl oleate	,	SuperPred
333.	Transthyretin	TTR	fisetin, kaempferol, luteolin	Swiss Target	SuperPred
334.	Tyrosinase	TYR	fisetin, kaempferol, luteolin	Swiss Target	
335.	Vascular endothelial growth factor receptor 2	VEGFR2	ethyl oleate		SuperPred
336.	Xanthine dehydrogenase	XDH	fisetin, kaempferol, luteolin	Swiss Target	

Supplementary Table S3: Target genes of Atherosclerosis

No	Target	Common name	Database 1	Database 2	Database 3
1	APOBEC1 Complementation Factor	A1CF		GeneCard	
2	Alpha-2-Macroglobulin	A2M		GeneCard	DisGenet
3	Aortic aneurysm, familial abdominal 1	AAA1			DisGenet
4	Aladin WD repeat nucleoporin	AAAS			DisGenet
5	AP2 Associated Kinase 1	AAK1		GeneCard	
6	Angio Associated Migratory Cell Protein	AAMP		GeneCard	
7	Aortic Aneurysm, Familial Thoracic 1	AAT1		GeneCard	
8	Aortic Aneurysm, Familial Thoracic 2	AAT2		GeneCard	
9	Adeno-Associated Virus Integration Site 1	AAVS1		GeneCard	
10	4-Aminobutyrate Aminotransferase	ABAT		GeneCard	
11	ATP Binding Cassette Subfamily A Member 1	ABCA1	OMIM	GeneCard	DisGenet
12	ATP Binding Cassette Subfamily A Member 12	ABCA12		GeneCard	DisGenet
13	ATP Binding Cassette Subfamily A Member 13	ABCA13		GeneCard	
14	ATP Binding Cassette Subfamily A Member 2	ABCA2		GeneCard	
15	ATP Binding Cassette Subfamily A Member 3	ABCA3		GeneCard	
16	ATP Binding Cassette Subfamily A Member 4	ABCA4		GeneCard	DisGenet
17	ATP Binding Cassette Subfamily A Member 5	ABCA5		GeneCard	
18	ATP Binding Cassette Subfamily A Member 7	ABCA7		GeneCard	
19	ATP Binding Cassette Subfamily A Member 8	ABCA8		GeneCard	
20	ATP Binding Cassette Subfamily B Member 1	ABCB1		GeneCard	
21	ATP Binding Cassette Subfamily B Member 11	ABCB11		GeneCard	
22	ATP Binding Cassette Subfamily B Member 4	ABCB4		GeneCard	
23	ATP Binding Cassette Subfamily B member 6 (Langereis blood group)	ABCB6			DisGenet
24	ATP Binding Cassette Subfamily C Member 1	ABCC1		GeneCard	DisGenet

25	ATP Binding cassette Subfamily C member 11	ABCC11			DisGenet
26	ATP Binding Cassette Subfamily C Member 2	ABCC2		GeneCard	
27	ATP Binding Cassette Subfamily C Member 6	ABCC6		GeneCard	DisGenet
28	ATP Binding Cassette Subfamily C Member 8	ABCC8		GeneCard	DisGenet
29	ATP Binding Cassette Subfamily C Member 9	ABCC9		GeneCard	
30	ATP Binding Cassette Subfamily D Member 1	ABCD1		GeneCard	DisGenet
31	ATP Binding Cassette Subfamily G Member 1	ABCG1		GeneCard	DisGenet
32	ATP Binding Cassette Subfamily G Member 2 (Junior Blood Group)	ABCG2		GeneCard	DisGenet
33	ATP Binding Cassette Subfamily G Member 4	ABCG4		GeneCard	
34	ATP-Binding Cassette, Subfamily G, member 5	ABCG5	OMIM	GeneCard	DisGenet
35	ATP-Binding Cassette, Subfamily G, member 8	ABCG8	OMIM	GeneCard	DisGenet
36	Abhydrolase Domain Containing 2	ABHD2			DisGenet
37	Abhydrolase Domain Containing 5, Lysophosphatidic Acid Acyltransferase	ABHD5		GeneCard	
38	Abl Interactor 1	ABI1		GeneCard	DisGenet
39	Abl Interactor 2	ABI2		GeneCard	DisGenet
40	ABL Proto-Oncogene 1, Non-Receptor Tyrosine Kinase	ABL1		GeneCard	
41	ABO, Alpha 1-3-N-Acetylgalactosaminyltransferase and Alpha 1-3-Galactosyltransferase	ABO		GeneCard	DisGenet
42	ABR activator of RhoGEF and GTPase	ABR			DisGenet
43	Abraxas 2, BRISC Complex Subunit	ABRAXAS2		GeneCard	
44	Acetyl-CoA Carboxylase Alpha	ACACA		GeneCard	DisGenet
45	Acyl-CoA Dehydrogenase Family Member 8	ACAD8		GeneCard	
46	Acyl-CoA Dehydrogenase Very Long Chain	ACADVL		GeneCard	
47	Aggrecan	ACAN		GeneCard	DisGenet
48	Acetyl-Coenzyme A acetyltransferase-1 (acetoacetyl Coenzyme A thiolase)	ACAT1	OMIM	GeneCard	DisGenet
49	Acetyl-CoA Acetyltransferase 2	ACAT2		GeneCard	
50	Acyl-CoA Binding Domain Containing 3	ACBD3		GeneCard	
51	1-aminocyclopropane-1-carboxylate synthase homolog (inactive)	ACCS			DisGenet
52	Angiotensin I Converting Enzyme	ACE		GeneCard	DisGenet
53	Angiotensin Converting Enzyme 2	ACE2		GeneCard	DisGenet

54	Alkaline Ceramidase 2	ACER2			DisGenet
55	Acetylcholinesterase (Cartwright Blood Group)	ACHE		GeneCard	
56	Atypical Chemokine Receptor 1 (Duffy Blood Group)	ACKR1		GeneCard	DisGenet
57	Atypical Chemokine Receptor 2	ACKR2		GeneCard	
58	Atypical Chemokine Receptor 3	ACKR3		GeneCard	DisGenet
59	Atypical Chemokine Receptor 4	ACKR4		GeneCard	
60	ATP Citrate Lyase	ACLY			DisGenet
61	Aconitase 2	ACO2		GeneCard	
62	Acyl-CoA Thioesterase 7	ACOT7			DisGenet
63	Acid Phosphatase 1	ACP1		GeneCard	DisGenet
64	Acid phosphatase 3	ACP3			DisGenet
65	Acid Phosphatase 5, Tartrate Resistant	ACP5		GeneCard	DisGenet
66	Acid Phosphatase 6, Lysophosphatidic	ACP6		GeneCard	
67	Acyl-CoA Synthetase Long Chain Family Member 1	ACSL1		GeneCard	DisGenet
68	Acyl-CoA Synthetase Long Chain Family Member 3	ACSL3		GeneCard	
69	Acyl-CoA Synthetase Long Chain Family Member 4	ACSL4		GeneCard	
70	Acyl-CoA Synthetase Long Chain Family Member 6	ACSL6		GeneCard	
71	Acyl-CoA Synthetase Medium Chain Family Member 2B	ACSM2B		GeneCard	
72	Acyl-CoA Synthetase Short Chain Family Member 2	ACSS2		GeneCard	DisGenet
73	Actin Alpha 1, Skeletal Muscle	ACTA1		GeneCard	
74	Actin Alpha 2, Smooth Muscle	ACTA2		GeneCard	DisGenet
75	ACTA2 Antisense RNA 1	ACTA2-AS1		GeneCard	
76	Actin Beta	ACTB		GeneCard	DisGenet
77	Actin Alpha Cardiac Muscle 1	ACTC1		GeneCard	
78	Actin Gamma 1	ACTG1		GeneCard	
79	Actin Gamma 2, Smooth Muscle	ACTG2		GeneCard	
80	Actinin Alpha 1	ACTN1		GeneCard	
81	Actinin Alpha 2	ACTN2		GeneCard	
82	Actinin Alpha 4	ACTN4		GeneCard	

83	Actin-related protein T1	ACTRT1			DisGenet
84	Activin A Receptor Type 1	ACVR1		GeneCard	
85	Activin A Receptor Type 2A	ACVR2A		GeneCard	
86	Activin A Receptor Like Type 1	ACVRL1		GeneCard	
87	Aminoacylase 3	ACY3		GeneCard	
88	Adenosine Deaminase	ADA		GeneCard	DisGenet
89	Adenosine Deaminase 2	ADA2		GeneCard	
90	ADAM Metallopeptidase Domain 10	ADAM10		GeneCard	DisGenet
91	ADAM Metallopeptidase Domain 12	ADAM12		GeneCard	DisGenet
92	ADAM metallopeptidase domain 15	ADAM15	OMIM	GeneCard	DisGenet
93	ADAM Metallopeptidase Domain 17	ADAM17		GeneCard	DisGenet
94	ADAM Metallopeptidase Domain 19	ADAM19		GeneCard	
95	ADAM Metallopeptidase Domain 2	ADAM2		GeneCard	
96	ADAM Metallopeptidase Domain 28	ADAM28		GeneCard	
97	ADAM Metallopeptidase Domain 33	ADAM33		GeneCard	DisGenet
98	ADAM Metallopeptidase Domain 3B (Pseudogene)	ADAM3B		GeneCard	
99	ADAM Metallopeptidase Domain 8	ADAM8		GeneCard	DisGenet
100	ADAM Metallopeptidase Domain 9	ADAM9		GeneCard	DisGenet
101	ADAM Metallopeptidase with Thrombospondin Type 1 Motif 1	ADAMTS1		GeneCard	DisGenet
102	ADAM Metallopeptidase with Thrombospondin Type 1 Motif 10	ADAMTS10		GeneCard	
103	ADAM Metallopeptidase with Thrombospondin Type 1 Motif 12	ADAMTS12		GeneCard	
104	ADAM Metallopeptidase with Thrombospondin Type 1 Motif 13	ADAMTS13		GeneCard	DisGenet
105	ADAM Metallopeptidase with Thrombospondin Type 1 Motif 14	ADAMTS14		GeneCard	
106	ADAM Metallopeptidase with Thrombospondin Type 1 Motif 17	ADAMTS17		GeneCard	
107	ADAM Metallopeptidase with Thrombospondin Type 1 Motif 18	ADAMTS18		GeneCard	DisGenet
108	ADAM Metallopeptidase with Thrombospondin Type 1 Motif 19	ADAMTS19		GeneCard	
109	ADAM Metallopeptidase with Thrombospondin Type 1 Motif 3	ADAMTS3		GeneCard	DisGenet
110	ADAM Metallopeptidase with Thrombospondin Type 1 Motif 4	ADAMTS4		GeneCard	DisGenet
111	ADAM Metallopeptidase with Thrombospondin Type 1 Motif 5	ADAMTS5		GeneCard	DisGenet

112	ADAM Metallopeptidase with Thrombospondin Type 1 Motif 6	ADAMTS6		GeneCard	
113	ADAM Metallopeptidase with Thrombospondin Type 1 Motif 7	ADAMTS7		GeneCard	DisGenet
114	ADAM Metallopeptidase with Thrombospondin Type 1 Motif 8	ADAMTS8		GeneCard	
115	ADAM Metallopeptidase with Thrombospondin Type 1 Motif 9	ADAMTS9		GeneCard	
116	ADAMTS Like 1	ADAMTSL1		GeneCard	
117	ADAMTS Like 2	ADAMTSL2		GeneCard	
118	ADAMTS Like 4	ADAMTSL4		GeneCard	
119	Adenosine Deaminase RNA Specific	ADAR		GeneCard	
120	Adenosine Deaminase RNA Specific B2 (Inactive)	ADARB2		GeneCard	
121	Adenylate Cyclase 1	ADCY1		GeneCard	
122	Adenylate Cyclase 10	ADCY10		GeneCard	DisGenet
123	Adenylate Cyclase 8	ADCY8			DisGenet
124	Adenylate Cyclase 9	ADCY9		GeneCard	DisGenet
125	Adenylate Cyclase Activating Polypeptide 1	ADCYAP1		GeneCard	DisGenet
126	Adducin 1	ADD1		GeneCard	DisGenet
127	Adhesion G protein-Coupled Receptor A2	ADGRA2			DisGenet
128	Adhesion G Protein-Coupled Receptor E1	ADGRE1		GeneCard	
129	Adhesion G Protein-Coupled Receptor E2	ADGRE2		GeneCard	
130	Adhesion G Protein-Coupled Receptor E3	ADGRE3		GeneCard	
131	Adhesion G Protein-Coupled Receptor E5	ADGRE5		GeneCard	
132	Adhesion G Protein-Coupled Receptor G6	ADGRG6		GeneCard	
133	Adhesion G Protein-Coupled Receptor L2	ADGRL2		GeneCard	
134	Alcohol Dehydrogenase 1B (Class I), Beta Polypeptide	ADH1B		GeneCard	
135	Alcohol Dehydrogenase 1C (Class I), Gamma Polypeptide	ADH1C		GeneCard	
136	Alcohol Dehydrogenase 4 (Class II), Pi Polypeptide	ADH4		GeneCard	
137	Alcohol Dehydrogenase 5 (class III), Chi polypeptide	ADH5			DisGenet
138	Alcohol Dehydrogenase 7 (Class IV), Mu or Sigma Polypeptide	ADH7		GeneCard	
139	Adipocyte-, C1q-, and collagen domain-containing	ADIPOQ	OMIM	GeneCard	DisGenet
140	ADIPOQ Antisense RNA 1	ADIPOQ-AS1		GeneCard	

141	Adiponectin Receptor 1	ADIPOR1		GeneCard	DisGenet
142	Adiponectin Receptor 2	ADIPOR2		GeneCard	DisGenet
143	Adenosine Kinase	ADK			DisGenet
144	Adrenomedullin	ADM		GeneCard	DisGenet
145	Adrenomedullin 2	ADM2		GeneCard	
146	Adenosine A1 Receptor	ADORA1		GeneCard	
147	Adenosine A2a Receptor	ADORA2A		GeneCard	
148	Adenosine A2b Receptor	ADORA2B		GeneCard	
149	ADP-Ribosylarginine Hydrolase	ADPRH		GeneCard	
150	Adrenoceptor Alpha 1A	ADRA1A			DisGenet
151	Adrenoceptor Alpha 1B	ADRA1B	OMIM	GeneCard	
152	Adrenoceptor Alpha 1D	ADRA1D		GeneCard	
153	Adrenoceptor Alpha 2A	ADRA2A		GeneCard	
154	Adrenoceptor Alpha 2B	ADRA2B		GeneCard	DisGenet
155	Adrenoceptor Alpha 2C	ADRA2C		GeneCard	
156	Adrenoceptor Beta 1	ADRB1		GeneCard	
157	Adrenoceptor Beta 2	ADRB2		GeneCard	DisGenet
158	Adrenoceptor Beta 3	ADRB3		GeneCard	DisGenet
159	Adenylosuccinate Lyase	ADSL		GeneCard	
160	Androgen Dependent TFPI Regulating Protein	ADTRP		GeneCard	DisGenet
161	AE Binding Protein 1	AEBP1		GeneCard	
162	Afadin, Adherens Junction Formation Factor	AFDN		GeneCard	
163	AF4/FMR2 Family Member 2	AFF2		GeneCard	
164	AF4/FMR2 Family Member 3	AFF3		GeneCard	
165	Alpha Fetoprotein	AFP		GeneCard	
166	ArfGAP With GTPase Domain, Ankyrin Repeat and PH Domain 3	AGAP3		GeneCard	
167	AGBL Carboxypeptidase 1	AGBL1		GeneCard	
168	Advanced Glycosylation End-Product Specific Receptor	AGER		GeneCard	DisGenet
169	Angiogenic Factor With G-Patch and FHA Domains 1	AGGF1		GeneCard	

170	Acylglycerol Kinase	AGK		GeneCard	
171	Amylo-Alpha-1, 6-Glucosidase, 4-Alpha-Glucanotransferase	AGL		GeneCard	
172	1-Acylglycerol-3-Phosphate O-Acyltransferase 1	AGPAT1		GeneCard	
173	1-Acylglycerol-3-Phosphate O-Acyltransferase 2	AGPAT2		GeneCard	DisGenet
174	Agouti related neuropeptide	AGRP			DisGenet
175	Angiotensinogen	AGT		GeneCard	DisGenet
176	Angiotensin Receptor 1	AGTR1	OMIM	GeneCard	DisGenet
177	Angiotensin II Receptor Type 2	AGTR2		GeneCard	DisGenet
178	Angiotensin II Receptor Associated Protein	AGTRAP		GeneCard	
179	Alanine–Glyoxylate and Serine–Pyruvate Aminotransferase	AGXT		GeneCard	DisGenet
180	Alanine–Glyoxylate Aminotransferase 2	AGXT2		GeneCard	DisGenet
181	Adenosylhomocysteinase	AHCY		GeneCard	DisGenet
182	Aryl Hydrocarbon Receptor	AHR		GeneCard	DisGenet
183	Aryl Hydrocarbon Receptor Repressor	AHRR		GeneCard	DisGenet
184	Activator of HSP90 ATPase Activity 1	AHSA1			DisGenet
185	Alpha-2HS-glycoprotein	AHSG	OMIM	GeneCard	DisGenet
186	Alpha Hemoglobin Stabilizing Protein	AHSP		GeneCard	
187	Axin Interactor, Dorsalization Associated	AIDA		GeneCard	
188	Allograft Inflammatory Factor 1	AIF1		GeneCard	DisGenet
189	Apoptosis Inducing Factor Mitochondria Associated 1	AIFM1		GeneCard	
190	Absent In Melanoma 2	AIM2		GeneCard	DisGenet
191	Aminoacyl tRNA Synthetase Complex Interacting Multifunctional Protein 1	AIMP1		GeneCard	DisGenet
192	Aminoacyl tRNA Synthetase Complex Interacting Multifunctional Protein 2	AIMP2			DisGenet
193	Aryl Hydrocarbon Receptor Interacting Protein Like 1	AIPL1		GeneCard	
194	Autoimmune Regulator	AIRE		GeneCard	
195	A-Kinase Anchoring Protein 12	AKAP12		GeneCard	DisGenet
196	A-Kinase Anchoring Protein 7	AKAP7		GeneCard	
197	A-Kinase Anchoring Protein 9	AKAP9		GeneCard	
198	AKNA Domain Containing 1	AKNAD1		GeneCard	

199	Aldo-Keto Reductase Family 1 Member A1	AKR1A1		GeneCard	
200	Aldo-Keto Reductase Family 1 Member B	AKR1B1		GeneCard	DisGenet
201	Aldo-Keto Reductase Family 1 Member B10	AKR1B10		GeneCard	DisGenet
202	Aldo-Keto Reductase Family 1 Member C4	AKR1C4		GeneCard	
203	AKT Serine/Threonine Kinase 1	AKT1		GeneCard	DisGenet
204	AKT1 Substrate 1	AKT1S1		GeneCard	DisGenet
205	AKT Serine/Threonine Kinase 2	AKT2		GeneCard	DisGenet
206	AKT Serine/Threonine Kinase 3	AKT3		GeneCard	DisGenet
207	Albumin	ALB	OMIM	GeneCard	DisGenet
208	Activated Leukocyte Cell Adhesion Molecule	ALCAM		GeneCard	DisGenet
209	Aldehyde Dehydrogenase 2 Family Member	ALDH2		GeneCard	DisGenet
210	Aldehyde Dehydrogenase 4 Family Member A1	ALDH4A1		GeneCard	
211	Aldehyde Dehydrogenase 8 Family Member A1	ALDH8A1		GeneCard	
212	Aldehyde Dehydrogenase 9 Family Member A1	ALDH9A1		GeneCard	
213	ALG6 Alpha-1,3-Glucosyltransferase	ALG6		GeneCard	
214	ALK Receptor Tyrosine Kinase	ALK		GeneCard	
215	ALKB Homolog 1, Histone H2A Dioxygenase	ALKBH1			DisGenet
216	ALMS1 centrosome and basal body associated protein	ALMS1	OMIM	GeneCard	DisGenet
217	ALMS1 Pseudogene 1	ALMS1P1		GeneCard	
218	Arachidonate 12-Lipoxygenase, 12S Type	ALOX12		GeneCard	DisGenet
219	Arachidonate 15-Lipoxygenase	ALOX15		GeneCard	DisGenet
220	Arachidonate 15-Lipoxygenase Type B	ALOX15B		GeneCard	DisGenet
221	Arachidonate 5-Lipoxygenase	ALOX5	OMIM	GeneCard	DisGenet
222	Arachidonate 5-Lipoxygenase-Activating Protein	ALOX5AP	OMIM	GeneCard	DisGenet
223	Alpha Kinase 3	ALPK3		GeneCard	
224	Alkaline Phosphatase, Biomineralization Associated	ALPL		GeneCard	DisGenet
225	Alkaline Phosphatase, Placental	ALPP		GeneCard	
226	Aly/REF Export Factor	ALYREF		GeneCard	
227	Alpha-1-Microglobulin/Bikunin Precursor	AMBP		GeneCard	

228	Autocrine Motility Factor Receptor	AMFR		GeneCard	
229	Anti-Mullerian Hormone	AMH		GeneCard	DisGenet
230	Anti-Mullerian Hormone Receptor Type 2	AMHR2		GeneCard	
231	Amnion Associated Transmembrane Protein	AMN		GeneCard	
232	Angiomotin	AMOT		GeneCard	DisGenet
233	Adenosine Monophosphate Deaminase 1	AMPD1		GeneCard	
234	Adenosine Monophosphate Deaminase 2	AMPD2		GeneCard	
235	Anaphase Promoting Complex Subunit 16	ANAPC16		GeneCard	
236	Angiogenin	ANG		GeneCard	
237	Angiopoietin 1	ANGPT1		GeneCard	DisGenet
238	Angiopoietin 2	ANGPT2		GeneCard	DisGenet
239	Angiopoietin Like 1	ANGPTL1		GeneCard	
240	Angiopoietin Like 2	ANGPTL2		GeneCard	DisGenet
241	Angiopoietin Like 3	ANGPTL3	OMIM	GeneCard	DisGenet
242	Angiopoietin Like 4	ANGPTL4		GeneCard	DisGenet
243	Angiopoietin Like 5	ANGPTL5		GeneCard	
244	Angiopoietin Like 6	ANGPTL6		GeneCard	DisGenet
245	Angiopoietin Like 7	ANGPTL7		GeneCard	
246	Angiopoietin Like 8	ANGPTL8		GeneCard	DisGenet
247	Ankyrin 1	ANK1		GeneCard	
248	Ankyrin 2	ANK2		GeneCard	
249	Ankyrin 3	ANK3		GeneCard	
250	ANKH Inorganic Pyrophosphate Transport Regulator	ANKH		GeneCard	
251	Ankyrin Repeat Domain-Containing Protein 1	ANKRD1	OMIM	GeneCard	
252	Ankyrin Repeat Domain 2	ANKRD2		GeneCard	
253	Ankyrin Repeat Domain 23	ANKRD23		GeneCard	
254	Ankyrin Repeat Domain 30A	ANKRD30A		GeneCard	
255	Ankyrin Repeat Domain 6	ANKRD6		GeneCard	
256	Ankyrin Repeat and Sterile Alpha Motif Domain Containing 1B	ANKS1B			DisGenet

257	Anillin, Actin Binding Protein	ANLN		GeneCard	
258	Anoctamin 5	ANO5		GeneCard	
259	Anoctamin 6	ANO6		GeneCard	
260	Alanyl Aminopeptidase, Membrane	ANPEP		GeneCard	DisGenet
261	ANTXR Cell Adhesion Molecule 1	ANTXR1		GeneCard	DisGenet
262	ANTXR Cell Adhesion Molecule 2	ANTXR2		GeneCard	
263	Annexin A1	ANXA1		GeneCard	DisGenet
264	Annexin A10	ANXA10		GeneCard	
265	Annexin A2	ANXA2		GeneCard	DisGenet
266	Annexin A5	ANXA5		GeneCard	DisGenet
267	Annexin A6	ANXA6		GeneCard	
268	Amine Oxidase Copper Containing 1	AOC1		GeneCard	
269	Amine Oxidase Copper Containing 2	AOC2			DisGenet
270	Amine Oxidase Copper Containing 3	AOC3		GeneCard	DisGenet
271	Adaptor Related Protein Complex 1 Subunit Beta 1	AP1B1		GeneCard	
272	Adaptor Related Protein Complex 2 Subunit Alpha 1	AP2A1			DisGenet
273	Adaptor Related Protein Complex 2 Subunit Beta 1	AP2B1		GeneCard	
274	Adaptor Related Protein Complex 3 Subunit Beta 1	AP3B1		GeneCard	
275	Adaptor Related Protein Complex 3 Subunit Delta 1	AP3D1		GeneCard	
276	Adaptor Related Protein Complex 3 Subunit Sigma 2	AP3S2		GeneCard	
277	AP4B1 Antisense RNA 1	AP4B1-AS1		GeneCard	
278	Amyloid Beta Precursor Protein Binding Family B Member 1	APBB1		GeneCard	
279	Amyloid Beta Precursor Protein Binding Family B Member 2	APBB2		GeneCard	
280	APC Regulator of WNT Signaling Pathway	APC		GeneCard	DisGenet
281	APC Down-Regulated 1	APCDD1		GeneCard	
282	Amyloid P Component, Serum	APCS		GeneCard	DisGenet
283	Acylaminoacyl-Peptide Hydrolase	APEH		GeneCard	
284	Apurinic/Apyrimidinic Endodeoxyribonuclease 1	APEX1		GeneCard	DisGenet
285	Aph-1 Homolog B, Gamma-Secretase Subunit	APH1B		GeneCard	

286	Apelin	APLN		GeneCard	DisGenet
287	Apelin Receptor	APLNR		GeneCard	DisGenet
288	Apolipoprotein A-I	APOA1	OMIM	GeneCard	DisGenet
289	APOA1 Antisense RNA	APOA1-AS		GeneCard	DisGenet
290	Apolipoprotein A-II	APOA2	OMIM	GeneCard	DisGenet
291	Apolipoprotein A-IV	APOA4	OMIM	GeneCard	DisGenet
292	Apolipoprotein A-V	APOA5	OMIM	GeneCard	DisGenet
293	Apolipoprotein B (including Ag(x) antigen)	APOB	OMIM	GeneCard	DisGenet
294	Apolipoprotein B MRNA Editing Enzyme Catalytic Subunit 1	APOBEC1		GeneCard	DisGenet
295	Apolipoprotein B Receptor	APOBR		GeneCard	DisGenet
296	Apolipoprotein C1	APOC1		GeneCard	DisGenet
297	Apolipoprotein C2	APOC2		GeneCard	DisGenet
298	Apolipoprotein C-III	APOC3	OMIM	GeneCard	DisGenet
299	Apolipoprotein C4	APOC4		GeneCard	
300	APOC4-APOC2 (NMD Candidate)	APOC4-APOC2		GeneCard	
301	Apolipoprotein D	APOD		GeneCard	
302	Apolipoprotein E	APOE		GeneCard	DisGenet
303	Apolipoprotein F	APOF		GeneCard	
304	Apolipoprotein H	APOH		GeneCard	DisGenet
305	Apolipoprotein L1	APOL1		GeneCard	DisGenet
306	Apolipoprotein L2	APOL2		GeneCard	
307	Apolipoprotein L3	APOL3		GeneCard	
308	Apolipoprotein L4	APOL4		GeneCard	
309	Apolipoprotein L5	APOL5		GeneCard	
310	Apolipoprotein L6	APOL6		GeneCard	DisGenet
311	Apolipoprotein M	APOM	OMIM	GeneCard	
312	Apolipoprotein O Pseudogene 1	APOOP1		GeneCard	
313	Amyloid Beta Precursor Protein	APP		GeneCard	DisGenet
314	Adaptor Protein, Phosphotyrosine Interacting with PH Domain and Leucine Zipper 1	APPL1			DisGenet

315	Adenine Phosphoribosyltransferase	APRT		GeneCard	DisGenet
316	Aprataxin	APTX		GeneCard	
317	Aquaporin 1 (Colton Blood Group)	AQP1		GeneCard	DisGenet
318	Aquaporin 7	AQP7		GeneCard	
319	Aquaporin 9	AQP9		GeneCard	
320	Androgen Receptor	AR		GeneCard	DisGenet
321	ADP Ribosylation Factor 1	ARF1		GeneCard	
322	ADP Ribosylation Factor Guanine Nucleotide Exchange Factor 1	ARFGEF1		GeneCard	
323	ADP Ribosylation Factor Guanine Nucleotide Exchange Factor 2	ARFGEF2		GeneCard	
324	Arginase 1	ARG1		GeneCard	DisGenet
325	Arginase 2	ARG2		GeneCard	
326	Rho GTPase Activating Protein 18	ARHGAP18		GeneCard	DisGenet
327	Rho GTPase Activating Protein 20	ARHGAP20		GeneCard	
328	Rho GTPase Activating Protein 24	ARHGAP24			DisGenet
329	Rho Guanine Nucleotide Exchange Factor 1	ARHGEF1			DisGenet
330	Rho Guanine Nucleotide Exchange Factor 10	ARHGEF10		GeneCard	
331	Rho/Rac Guanine Nucleotide Exchange Factor 2	ARHGEF2			DisGenet
332	Rho Guanine Nucleotide Exchange Factor 26	ARHGEF26			DisGenet
333	Rho Guanine Nucleotide Exchange Factor 28	ARHGEF28			DisGenet
334	Rho Guanine Nucleotide Exchange Factor 38	ARHGEF38		GeneCard	
335	Rho Guanine Nucleotide Exchange Factor 5	ARHGEF5			DisGenet
336	AT-Rich Interaction Domain 5B	ARID5B		GeneCard	DisGenet
337	Ariadne RBR E3 Ubiquitin Protein Ligase 1	ARIH1		GeneCard	
338	ADP Ribosylation Factor Like GTPase 13B	ARL13B		GeneCard	
339	ADP Ribosylation Factor Like GTPase 15	ARL15		GeneCard	
340	ADP Ribosylation Factor Like GTPase 6	ARL6		GeneCard	
341	ADP Ribosylation Factor Like GTPase 6 Interacting Protein 1	ARL6IP1			DisGenet
342	Armadillo Like Helical Domain Containing 1	ARMH1			DisGenet
343	Armadillo Like Helical Domain Containing 3	ARMH3		GeneCard	

344	Age-Related Maculopathy Susceptibility 2	ARMS2		GeneCard	
345	Aryl Hydrocarbon Receptor Nuclear Translocator	ARNT		GeneCard	
346	Aryl Hydrocarbon Receptor Nuclear Translocator Like	ARNTL		GeneCard	DisGenet
347	Actin Related Protein 2/3 Complex Subunit 4	ARPC4		GeneCard	
348	Arrestin 3	ARR3			DisGenet
349	Arrestin Beta 1	ARRB1		GeneCard	
350	Arrestin Beta 2	ARRB2		GeneCard	
351	Arrestin Domain Containing 4	ARRDC4		GeneCard	
352	Arylsulfatase A	ARSA		GeneCard	
353	Arylsulfatase B	ARSB		GeneCard	DisGenet
354	Arylsulfatase Family Member H	ARSH		GeneCard	
355	Arylsulfatase Family Member I	ARSI			DisGenet
356	Arylsulfatase L	ARSL			DisGenet
357	Artemin	ARTN			DisGenet
358	Arsenite Methyltransferase	AS3MT		GeneCard	DisGenet
359	N-Acylsphingosine Amidohydrolase 1	ASAH1		GeneCard	
360	Activating Signal Cointegrator 1 Complex Subunit 3	ASCC3		GeneCard	
361	Asialoglycoprotein Receptor 1	ASGR1		GeneCard	
362	Asialoglycoprotein Receptor 2	ASGR2		GeneCard	
363	Aspartoacylase	ASPA		GeneCard	
364	Argininosuccinate Synthase 1	ASS1		GeneCard	DisGenet
365	Astrotactin 1	ASTN1		GeneCard	
366	ASXL Transcriptional Regulator 1	ASXL1		GeneCard	
367	ASXL Transcriptional Regulator 2	ASXL2		GeneCard	
368	Activating Transcription Factor 1	ATF1		GeneCard	
369	Activating Transcription Factor 2	ATF2		GeneCard	DisGenet
370	Activating Transcription Factor 3	ATF3		GeneCard	
371	Activating Transcription Factor 4	ATF4		GeneCard	DisGenet
372	Activating Transcription Factor 6	ATF6		GeneCard	

373	Autophagy Related 13	ATG13			DisGenet
374	Autophagy Related 16 Like 1	ATG16L1		GeneCard	DisGenet
375	Autophagy Related 5	ATG5		GeneCard	DisGenet
376	Autophagy Related 7	ATG7		GeneCard	DisGenet
377	Autophagy Related 9B	ATG9B		GeneCard	
378	Atherosclerosis Susceptibility (Lipoprotein Associated)	ATHS		GeneCard	
379	5-Aminoimidazole-4-Carboxamide Ribonucleotide Formyltransferase/IMP Cyclohydrolase	ATIC		GeneCard	DisGenet
380	ATM serine/threonine kinase	ATM	OMIM	GeneCard	DisGenet
381	Atrophin 1	ATN1		GeneCard	DisGenet
382	Atonal bHLH Transcription Factor 1	ATOH1			DisGenet
383	Antioxidant 1 Copper Chaperone	ATOX1		GeneCard	DisGenet
384	ATPase Phospholipid Transporting 10A (Putative)	ATP10A		GeneCard	
385	ATPase Phospholipid Transporting 10D (Putative)	ATP10D		GeneCard	
386	ATPase Cation Transporting 13A2	ATP13A2		GeneCard	
387	ATPase Na+/K+ Transporting Subunit Alpha 1	ATP1A1		GeneCard	
388	ATPase Sarcoplasmic/Endoplasmic Reticulum Ca2+ Transporting 2	ATP2A2		GeneCard	
389	ATPase Plasma Membrane Ca2+ Transporting 1	ATP2B1		GeneCard	DisGenet
390	ATP Synthase F1 Subunit Alpha	ATP5F1A		GeneCard	
391	ATP Synthase F1 Subunit Beta	ATP5F1B		GeneCard	
392	ATP Synthase Inhibitory Factor Subunit 1	ATP5IF1		GeneCard	
393	ATP Synthase Membrane Subunit K	ATP5MK		GeneCard	
394	ATP Synthase Peripheral Stalk Subunit F6	ATP5PF		GeneCard	
395	ATPase H+ Transporting Accessory Protein 2	ATP6AP2		GeneCard	
396	ATPase H+ Transporting V0 Subunit A2	ATP6V0A2		GeneCard	DisGenet
397	ATPase H+ Transporting V1 Subunit C2	ATP6V1C2		GeneCard	
398	ATPase H+ Transporting V1 Subunit E1	ATP6V1E1		GeneCard	
399	ATPase H+ Transporting V1 Subunit G2	ATP6V1G2		GeneCard	
400	ATP6V1G2-DDX39B Readthrough (NMD Candidate)	ATP6V1G2-DDX39B		GeneCard	

401	ATPase Copper Transporting Alpha	ATP7A		GeneCard	DisGenet
402	ATPase Phospholipid Transporting 8B1	ATP8B1		GeneCard	
403	ATR Serine/Threonine Kinase	ATR		GeneCard	
404	ATR Interacting Protein	ATRIP		GeneCard	
405	ATRIP-TREX1 Readthrough	ATRIP-TREX1		GeneCard	
406	Ataxin 1	ATXN1		GeneCard	
407	Ataxin 2	ATXN2		GeneCard	
408	Ataxin 3	ATXN3		GeneCard	
409	Aurora Kinase A and Ninein Interacting Protein	AUNIP			DisGenet
410	Aurora Kinase A	AURKA		GeneCard	DisGenet
411	Arginine Vasopressin	AVP		GeneCard	
412	Acyl-CoA Wax Alcohol Acyltransferase 1	AWAT1		GeneCard	
413	Acyl-CoA Wax Alcohol Acyltransferase 2	AWAT2		GeneCard	
414	AXL Receptor Tyrosine Kinase	AXL		GeneCard	
415	Alpha-2-Glycoprotein 1, Zinc-Binding	AZGP1		GeneCard	DisGenet
416	Azurocidin 1	AZU1		GeneCard	DisGenet
417	Beta-2-Microglobulin	B2M		GeneCard	DisGenet
418	Beta-1,3-Galactosyltransferase 4	B3GALT4		GeneCard	
419	Beta-1,4-N-Acetyl-Galactosaminyltransferase 3	B4GALNT3		GeneCard	
420	Beta-1,4-Galactosyltransferase 1	B4GALT1		GeneCard	
421	Beta-1,4-Galactosyltransferase 6	B4GALT6		GeneCard	
422	Beta-Secretase 1	BACE1		GeneCard	DisGenet
423	BTB Domain and CNC Homolog 1	BACH1		GeneCard	
424	BCL2 Associated Agonist of Cell Death	BAD		GeneCard	
425	BAG Cochaperone 3	BAG3		GeneCard	
426	BAR/IMD Domain Containing Adaptor Protein 2 Like 1	BAIAP2L1		GeneCard	
427	BCL2 Antagonist/Killer 1	BAK1		GeneCard	
428	BMP and Activin Membrane Bound Inhibitor	BAMBI			DisGenet
429	BRAF-ctivated Noncoding RNA	BANCR	OMIM		

430	Barrier to Autointegration Factor 1	BANF1	OMIM	GeneCard	DisGenet
431	B Cell Scaffold Protein with Ankyrin Repeats 1	BANK1		GeneCard	
432	BarH Like Homeobox 2	BARHL2		GeneCard	
433	Basic leucine zipper ATF-like transcription factor 3	BATF3			DisGenet
434	BCL2 Associated X, Apoptosis Regulator	BAX		GeneCard	DisGenet
435	Bromodomain Adjacent to Zinc Finger Domain 1A	BAZ1A		GeneCard	
436	Bromodomain Adjacent to Zinc Finger Domain 1B	BAZ1B		GeneCard	
437	Bromodomain Adjacent to Zinc Finger Domain 2B	BAZ2B		GeneCard	
438	Bardet–Biedl Syndrome 1	BBS1		GeneCard	
439	Bardet–Biedl Syndrome 10	BBS10		GeneCard	
440	Bardet–Biedl Syndrome 2	BBS2		GeneCard	
441	Bardet–Biedl Syndrome 4	BBS4		GeneCard	
442	Bardet–Biedl Syndrome 5	BBS5		GeneCard	
443	Bardet–Biedl Syndrome 7	BBS7		GeneCard	
444	Bardet–Biedl Syndrome 9	BBS9		GeneCard	
445	Basal Cell Adhesion Molecule (Lutheran Blood Group)	BCAM		GeneCard	
446	BCAR1 Scaffold Protein, Cas Family Member	BCAR1		GeneCard	
447	BCAR3 Adaptor Protein, NSP Family Member	BCAR3		GeneCard	
448	Breast Cancer Anti-Estrogen Resistance 4	BCAR4		GeneCard	
449	Butyrylcholinesterase	BCHE		GeneCard	
450	BCL2 Apoptosis Regulator	BCL2		GeneCard	DisGenet
451	BCL2 Related Protein A1	BCL2A1		GeneCard	
452	BCL2 Like 1	BCL2L1		GeneCard	
453	BCL2 Like 11	BCL2L11		GeneCard	
454	BCL3 Transcription Coactivator	BCL3		GeneCard	DisGenet
455	BCL6 Transcription Repressor	BCL6		GeneCard	
456	Beta-Carotene Oxygenase 1	BCO1		GeneCard	
457	BCL6 Corepressor	BCOR		GeneCard	
458	BCR activator of RhoGEF and GTPase	BCR			DisGenet

459	Bradykinin Receptor B1	BDKRB1		GeneCard	
460	Bradykinin Receptor B2	BDKRB2		GeneCard	DisGenet
461	Brain Derived Neurotrophic Factor	BDNF		GeneCard	DisGenet
462	Beclin 1	BECN1		GeneCard	DisGenet
463	Bestrophin 1	BEST1			DisGenet
464	Bestrophin 3	BEST3		GeneCard	
465	Beaded Filament Structural Protein 1	BFSP1		GeneCard	
466	Bone Gamma-Carboxyglutamate Protein	BGLAP		GeneCard	DisGenet
467	Biglycan	BGN		GeneCard	DisGenet
468	Basic Helix-Loop-Helix Family Member E22	BHLHE22			DisGenet
469	Betaine--Homocysteine S-Methyltransferase	BHMT		GeneCard	
470	Betaine--Homocysteine S-Methyltransferase 2	BHMT2		GeneCard	
471	BH3 Interacting Domain Death Agonist	BID		GeneCard	
472	BCL2 Interacting Killer	BIK			DisGenet
473	Baculoviral IAP Repeat Containing 3	BIRC3		GeneCard	DisGenet
474	Baculoviral IAP Repeat Containing 5	BIRC5		GeneCard	
475	BLK Proto-Oncogene, Src Family Tyrosine Kinase	BLK		GeneCard	
476	BLM RecQ Like Helicase	BLM		GeneCard	DisGenet
477	BLOC1S5-TXNDC5 Readthrough (NMD Candidate)	BLOC1S5-<br>TXNDC5		GeneCard	
478	Biliverdin Reductase A	BLVRA		GeneCard	
479	Biliverdin Reductase B	BLVRB		GeneCard	
480	Bone Morphogenetic Protein 1	BMP1		GeneCard	
481	Bone Morphogenetic Protein 15	BMP15		GeneCard	
482	Bone Morphogenetic Protein 2	BMP2		GeneCard	DisGenet
483	Bone Morphogenetic Protein 3	BMP3		GeneCard	
484	Bone Morphogenetic Protein 4	BMP4		GeneCard	DisGenet
485	Bone Morphogenetic Protein 5	BMP5		GeneCard	
486	Bone Morphogenetic Protein 6	BMP6		GeneCard	

487	Bone Morphogenetic Protein 7	BMP7		GeneCard	DisGenet
488	BMP Binding Endothelial Regulator	BMPER		GeneCard	
489	Bone Morphogenetic Protein Receptor Type 1B	BMPR1B		GeneCard	
490	Bone Morphogenetic Protein Receptor Type 2	BMPR2		GeneCard	
491	BMS1 ribosome biogenesis factor	BMS1			DisGenet
492	Basonuclin 1	BNC1		GeneCard	
493	Basonuclin 2	BNC2		GeneCard	
494	BCL2 Interacting Protein 3	BNIP3		GeneCard	
495	BOP1 Ribosomal Biogenesis Factor	BOP1		GeneCard	
496	Bactericidal Permeability Increasing Protein	BPI		GeneCard	
497	BPI Fold Containing Family B Member 4	BPIFB4		GeneCard	DisGenet
498	BRCA1 Associated Protein	BRAP		GeneCard	DisGenet
499	BRCA1 DNA Repair Associated	BRCA1		GeneCard	DisGenet
500	BRCA2 DNA Repair Associated	BRCA2		GeneCard	
501	BRCA1/BRCA2-Containing Complex Subunit 3	BRCC3		GeneCard	
502	Bromodomain containing 7	BRD7			DisGenet
503	Bromodomain containing 8	BRD8			DisGenet
504	Bromodomain Testis Associated	BRDT		GeneCard	
505	BRF2 RNA Polymerase III Transcription Initiation Factor Subunit	BRF2		GeneCard	
506	BMP/Retinoic Acid Inducible Neural Specific 1	BRINP1			DisGenet
507	BMP/Retinoic Acid Inducible Neural Specific 3	BRINP3		GeneCard	DisGenet
508	Bromodomain and PHD Finger Containing 3	BRPF3		GeneCard	
509	Bombesin Receptor Subtype 3	BRS3			DisGenet
510	BR Serine/Threonine Kinase 1	BRSK1		GeneCard	
511	Bromodomain and WD Repeat Domain Containing 1	BRWD1		GeneCard	
512	BSCL2 Lipid Droplet Biogenesis Associated, Seipin	BSCL2		GeneCard	DisGenet
513	Basigin (Ok Blood Group)	BSG		GeneCard	DisGenet
514	Betacellulin	BTC		GeneCard	
515	Basic Transcription Factor 3 Pseudogene 11	BTF3P11			DisGenet

516	BTG Anti-Proliferation Factor 1	BTG1		GeneCard	
517	Bruton Tyrosine Kinase	BTK		GeneCard	
518	B And T Lymphocyte Associated	BTLA		GeneCard	DisGenet
519	Butyrophilin Like 2	BTNL2		GeneCard	
520	Beta-Transducin Repeat Containing E3 Ubiquitin Protein Ligase	BTRC		GeneCard	
521	BUB1 Mitotic Checkpoint Serine/Threonine Kinase B	BUB1B		GeneCard	
522	BUD13 Homolog	BUD13		GeneCard	
523	Chromosome 12 Open Reading Frame 4	C12orf4		GeneCard	
524	Chromosome 12 Open Reading Frame 43	C12orf43		GeneCard	
525	Chromosome 19 Open Reading Frame 38	C19orf38		GeneCard	
526	C1D Nuclear Receptor Corepressor	C1D			DisGenet
527	Chromosome 1 Open Reading Frame 116	C1orf116		GeneCard	
528	Chromosome 1 Open Reading Frame 21	C1orf21		GeneCard	
529	Complement C1q A Chain	C1QA	OMIM	GeneCard	
530	Complement C1q B Chain	C1QB		GeneCard	
531	Complement C1q Binding Protein	C1QBP		GeneCard	
532	Complement C1q C Chain	C1QC		GeneCard	
533	Complement C1q Like 3	C1QL3		GeneCard	DisGenet
534	C1q and TNF Related 1	C1QTNF1		GeneCard	DisGenet
535	C1q and TNF Related 12	C1QTNF12		GeneCard	DisGenet
536	C1q and TNF Related 3	C1QTNF3		GeneCard	
537	C1q and TNF Related 5	C1QTNF5		GeneCard	DisGenet
538	C1q and TNF Related 6	C1QTNF6		GeneCard	DisGenet
539	C1q and TNF Related 9	C1QTNF9		GeneCard	DisGenet
540	Complement C1r	C1R		GeneCard	
541	Complement C1s	C1S		GeneCard	
542	Complement C2	C2		GeneCard	
543	Chromosome 20 Open Reading Frame 181	C20orf181			DisGenet
544	Complement C3	C3		GeneCard	DisGenet

545	Complement C3a Receptor 1	C3AR1		GeneCard	
546	Complement C4A (Rodgers Blood Group)	C4A		GeneCard	
547	Complement C4B (Chido Blood Group)	C4B		GeneCard	
548	Complement Component 4B (Chido Blood Group), Copy 2	C4B_2		GeneCard	
549	Complement Component 4 Binding Protein Alpha	C4BPA		GeneCard	
550	Complement Component 4 Binding Protein Beta	C4BPB		GeneCard	
551	Complement C5	C5		GeneCard	
552	Complement C5a Receptor 1	C5AR1		GeneCard	
553	Complement C5a Receptor 2	C5AR2		GeneCard	
554	Complement C6	C6		GeneCard	
555	Complement C7	C7		GeneCard	
556	Complement C8 Beta Chain	C8B		GeneCard	
557	Chromosome 8 Open Reading Frame 48	C8orf48		GeneCard	
558	Complement C9	C9		GeneCard	
559	Chromosome 9 Open Reading Frame 50	C9orf50		GeneCard	
560	C9orf72-SMCR8 Complex Subunit	C9orf72		GeneCard	
561	Carbonic Anhydrase 1	CA1		GeneCard	DisGenet
562	Carbonic Anhydrase 10	CA10		GeneCard	
563	Carbonic Anhydrase 12	CA12		GeneCard	
564	Carbonic Anhydrase 2	CA2		GeneCard	DisGenet
565	Carbonic Anhydrase 3	CA3		GeneCard	
566	Carbonic Anhydrase 9	CA9		GeneCard	
567	Calcineurin Binding Protein 1	CABIN1		GeneCard	
568	Calcium Voltage-Gated Channel Subunit Alpha1 C	CACNA1C	OMIM	GeneCard	DisGenet
569	Calcium Voltage-Gated Channel Subunit Alpha1 E	CACNA1E		GeneCard	
570	Calcium Voltage-Gated Channel Subunit Alpha1 H	CACNA1H		GeneCard	
571	Calcium Voltage-Gated Channel Auxiliary Subunit Alpha2delta 1	CACNA2D1		GeneCard	
572	Calcium Voltage-Gated Channel Auxiliary Subunit Alpha2delta 3	CACNA2D3		GeneCard	
573	Calcium Voltage-Gated Channel Auxiliary Subunit Alpha2 Delta 4	CACNA2D4			DisGenet

574	Calcium Voltage-Gated Channel Auxiliary Subunit Gamma 8	CACNG8		GeneCard	
575	Carbamoyl-Phosphate Synthetase 2, Aspartate Transcarbamylase, and Dihydroorotase	CAD			DisGenet
576	Calcium Dependent Secretion Activator	CADPS		GeneCard	
577	Calcitonin Related Polypeptide Alpha	CALCA		GeneCard	
578	Calcium Binding and Coiled-Coil Domain 2	CALCOCO2		GeneCard	
579	Calcitonin Receptor	CALCR		GeneCard	
580	Calmodulin 1	CALM1		GeneCard	
581	Calmodulin 3	CALM3		GeneCard	
582	Calreticulin	CALR		GeneCard	DisGenet
583	Calumenin	CALU		GeneCard	DisGenet
584	Calcium/Calmodulin Dependent Protein Kinase I	CAMK1		GeneCard	
585	Calcium/Calmodulin Dependent Protein Kinase II Beta	CAMK2B		GeneCard	
586	Calcium/Calmodulin Dependent Protein Kinase II Delta	CAMK2D		GeneCard	
587	Calcium/Calmodulin Dependent Protein Kinase II Gamma	CAMK2G		GeneCard	
588	Calcium/Calmodulin Dependent Protein Kinase II Inhibitor 2	CAMK2N2		GeneCard	
589	Calcium/Calmodulin Dependent Protein Kinase IV	CAMK4		GeneCard	
590	Calcium/Calmodulin Dependent Protein Kinase Kinase 2	CAMKK2		GeneCard	DisGenet
591	Calcium Modulating Ligand	CAMLG		GeneCard	
592	Cathelicidin Antimicrobial Peptide	CAMP		GeneCard	DisGenet
593	Calmodulin Binding Transcription Activator 1	CAMTA1		GeneCard	
594	Cullin Associated and Neddylation Dissociated 1	CAND1		GeneCard	
595	Calcium Activated Nucleotidase 1	CANT1		GeneCard	
596	Calnexin	CANX		GeneCard	DisGenet
597	Cyclase Associated Actin Cytoskeleton Regulatory Protein 1	CAP1		GeneCard	DisGenet
598	Capping Actin Protein, Gelsolin Like	CAPG		GeneCard	DisGenet
599	Calpain 1	CAPN1		GeneCard	DisGenet
600	Calpain 10	CAPN10		GeneCard	DisGenet
601	Calpain 2	CAPN2		GeneCard	
602	Calpain 5	CAPN5		GeneCard	

603	Calpain 6	CAPN6		GeneCard	
604	Cell Cycle Associated Protein 1	CAPRIN1		GeneCard	
605	Calcyphosine 2	CAPS2		GeneCard	
606	Capping Actin Protein of Muscle Z-Line Subunit Beta	CAPZB		GeneCard	
607	Caspase Recruitment Domain Family Member 16	CARD16		GeneCard	
608	Caspase Recruitment Domain Family Member 8	CARD8		GeneCard	DisGenet
609	Caspase Recruitment Domain Family Member 9	CARD9		GeneCard	DisGenet
610	Calcium Regulated Heat Stable Protein 1	CARHSP1		GeneCard	DisGenet
611	Cardiac Mesoderm Enhancer-Associated Non-Coding RNA	CARMN		GeneCard	
612	CART Prepropeptide	CARTPT		GeneCard	
613	Cancer Susceptibility 11	CASC11		GeneCard	DisGenet
614	Cancer Susceptibility 2	CASC2		GeneCard	
615	Caspase 1	CASP1		GeneCard	DisGenet
616	Caspase 2	CASP2		GeneCard	
617	Caspase 3	CASP3		GeneCard	DisGenet
618	Caspase 4	CASP4		GeneCard	
619	Caspase 6	CASP6		GeneCard	
620	Caspase 7	CASP7		GeneCard	
621	Caspase 8	CASP8		GeneCard	
622	Caspase 9	CASP9		GeneCard	DisGenet
623	Calsequestrin 2	CASQ2		GeneCard	
624	Calcium Sensing Receptor	CASR		GeneCard	DisGenet
625	Calpastatin	CAST		GeneCard	DisGenet
626	Castor Zinc Finger 1	CASZ1		GeneCard	DisGenet
627	Catalase	CAT		GeneCard	DisGenet
628	Caveolin 1	CAV1		GeneCard	DisGenet
629	Caveolin 2	CAV2		GeneCard	
630	Caveolin 3	CAV3		GeneCard	
631	Caveolae Associated Protein 1	CAVIN1		GeneCard	DisGenet

632	Caveolae Associated Protein 3	CAVIN3		GeneCard	
633	Caveolae Associated Protein 4	CAVIN4		GeneCard	
634	Cbl Proto-Oncogene	CBL		GeneCard	
635	Cbl Proto-Oncogene Like 1	CBLL1		GeneCard	
636	Carbonyl Reductase 1	CBR1		GeneCard	
637	Cystathionine Beta-Synthase	CBS		GeneCard	DisGenet
638	Cystathionine Beta-Synthase Like	CBSL			DisGenet
639	Chromobox 1	CBX1		GeneCard	
640	Chromobox 3	CBX3		GeneCard	
641	Chromobox 4	CBX4		GeneCard	
642	Chromobox 5	CBX5		GeneCard	
643	Cell Cycle and Apoptosis Regulator 2	CCAR2		GeneCard	DisGenet
644	Coiled-Coil Domain Containing 105	CCDC105		GeneCard	
645	Coiled-Coil Domain Containing 157	CCDC157		GeneCard	
646	Coiled-Coil Domain Containing 159	CCDC159		GeneCard	
647	Coiled-Coil Domain Containing 170	CCDC170		GeneCard	
648	Coiled-Coil Domain Containing 178	CCDC178		GeneCard	
649	Coiled-Coil Domain Containing 3	CCDC3		GeneCard	
650	Coiled-Coil Domain Containing 71 Like	CCDC71L		GeneCard	
651	Coiled-Coil Domain Containing 8	CCDC8			DisGenet
652	Coiled-Coil Domain Containing 80	CCDC80		GeneCard	DisGenet
653	Coiled-Coil Domain Containing 88B	CCDC88B		GeneCard	
654	Coiled-Coil Domain Containing 92	CCDC92		GeneCard	
655	Coiled-Coil Alpha-Helical Rod Protein 1	CCHCR1			DisGenet
656	Cholecystokinin	CCK		GeneCard	
657	C-C Motif Chemokine Ligand 1	CCL1		GeneCard	DisGenet
658	C-C Motif Chemokine Ligand 11	CCL11		GeneCard	DisGenet
659	C-C Motif Chemokine Ligand 13	CCL13		GeneCard	
660	C-C Motif Chemokine Ligand 14	CCL14		GeneCard	

661	C-C Motif Chemokine Ligand 15	CCL15		GeneCard	
662	C-C Motif Chemokine Ligand 16	CCL16		GeneCard	
663	C-C Motif Chemokine Ligand 17	CCL17		GeneCard	DisGenet
664	C-C Motif Chemokine Ligand 18	CCL18		GeneCard	DisGenet
665	C-C Motif Chemokine Ligand 19	CCL19		GeneCard	DisGenet
666	C-C Motif Chemokine Ligand 2	CCL2		GeneCard	DisGenet
667	C-C Motif Chemokine Ligand 20	CCL20		GeneCard	DisGenet
668	C-C Motif Chemokine Ligand 21	CCL21		GeneCard	DisGenet
669	C-C Motif Chemokine Ligand 22	CCL22		GeneCard	DisGenet
670	C-C Motif Chemokine Ligand 23	CCL23		GeneCard	DisGenet
671	C-C Motif Chemokine Ligand 24	CCL24		GeneCard	
672	C-C Motif Chemokine Ligand 25	CCL25		GeneCard	DisGenet
673	C-C Motif Chemokine Ligand 26	CCL26		GeneCard	
674	C-C Motif Chemokine Ligand 27	CCL27		GeneCard	
675	C-C Motif Chemokine Ligand 28	CCL28		GeneCard	
676	C-C Motif Chemokine Ligand 3	CCL3		GeneCard	DisGenet
677	C-C Motif Chemokine Ligand 4	CCL4		GeneCard	DisGenet
678	C-C Motif Chemokine Ligand 4 Like 1	CCL4L1		GeneCard	DisGenet
679	C-C motif chemokine ligand 4 like 2	CCL4L2			DisGenet
680	C-C Motif Chemokine Ligand 5	CCL5		GeneCard	DisGenet
681	C-C Motif Chemokine Ligand 7	CCL7		GeneCard	DisGenet
682	C-C Motif Chemokine Ligand 8	CCL8		GeneCard	DisGenet
683	CCM2 Scaffold Protein	CCM2		GeneCard	
684	Cellular Communication Network Factor 1	CCN1		GeneCard	DisGenet
685	Cellular Communication Network Factor 2	CCN2		GeneCard	DisGenet
686	Cellular Communication Network Factor 3	CCN3		GeneCard	DisGenet
687	Cellular Communication Network Factor 4	CCN4		GeneCard	
688	Cellular Communication Network Factor 5	CCN5		GeneCard	DisGenet
689	Cellular Communication Network Factor 6	CCN6		GeneCard	

690	Cyclin A2	CCNA2		GeneCard	
691	Cyclin B1	CCNB1		GeneCard	
692	Cyclin B2	CCNB2		GeneCard	
693	Cyclin D1	CCND1		GeneCard	DisGenet
694	Cyclin D2	CCND2		GeneCard	
695	Cyclin D3	CCND3		GeneCard	
696	Cyclin E1	CCNE1		GeneCard	
697	Cyclin F	CCNF		GeneCard	
698	Cyclin H	CCNH		GeneCard	
699	Cyclin T1	CCNT1		GeneCard	
700	C-C Motif Chemokine Receptor 1	CCR1		GeneCard	
701	C-C Motif Chemokine Receptor 2	CCR2	OMIM	GeneCard	DisGenet
702	C-C Motif Chemokine Receptor 3	CCR3		GeneCard	DisGenet
703	C-C Motif Chemokine Receptor 4	CCR4		GeneCard	
704	C-C Motif Chemokine Receptor 5	CCR5		GeneCard	DisGenet
705	C-C Motif Chemokine Receptor 6	CCR6		GeneCard	DisGenet
706	C-C Motif Chemokine Receptor 7	CCR7		GeneCard	DisGenet
707	C-C Motif Chemokine Receptor 8	CCR8		GeneCard	DisGenet
708	C-C Motif Chemokine Receptor 9	CCR9		GeneCard	DisGenet
709	C-C Motif Chemokine Receptor Like 2	CCRL2		GeneCard	
710	Chaperonin Containing TCP1 Subunit 2	CCT2		GeneCard	
711	Chaperonin Containing TCP1 Subunit 4	CCT4		GeneCard	
712	Chaperonin Containing TCP1 Subunit 6A	CCT6A		GeneCard	
713	Chaperonin Containing TCP1 Subunit 7	CCT7		GeneCard	
714	Chaperonin Containing TCP1 Subunit 8	CCT8		GeneCard	
715	CD14 antigen	CD14	OMIM	GeneCard	DisGenet
716	CD151 Molecule (Raph Blood Group)	CD151		GeneCard	
717	CD160 Molecule	CD160		GeneCard	
718	CD163 Molecule	CD163		GeneCard	DisGenet

719	CD163 Molecule Like 1	CD163L1		GeneCard	
720	CD180 molecule	CD180			DisGenet
721	CD19 Molecule	CD19		GeneCard	DisGenet
722	CD1a Molecule	CD1A		GeneCard	
723	CD1c Molecule	CD1C		GeneCard	
724	CD1d Molecule	CD1D		GeneCard	DisGenet
725	CD2 Molecule	CD2		GeneCard	
726	CD209 Molecule	CD209		GeneCard	
727	CD22 Molecule	CD22		GeneCard	
728	CD247 Molecule	CD247		GeneCard	
729	CD248 Molecule	CD248		GeneCard	DisGenet
730	CD27 Molecule	CD27		GeneCard	DisGenet
731	CD274 Molecule	CD274		GeneCard	DisGenet
732	CD276 Molecule	CD276		GeneCard	
733	CD28 Molecule	CD28		GeneCard	DisGenet
734	CD34 Molecule	CD34		GeneCard	DisGenet
735	CD36 antigen (collagen type I)	CD36	OMIM	GeneCard	DisGenet
736	CD38 Molecule	CD38		GeneCard	
737	CD4 Molecule	CD4		GeneCard	
738	CD40 Molecule	CD40		GeneCard	DisGenet
739	CD40 Ligand	CD40LG		GeneCard	DisGenet
740	CD44 Molecule (Indian Blood Group)	CD44		GeneCard	DisGenet
741	CD46 Molecule	CD46		GeneCard	DisGenet
742	CD47 Antigen (Rh-related antigen; integrin-associated protein)	CD47	OMIM	GeneCard	DisGenet
743	CD5 Molecule	CD5		GeneCard	
744	CD55 Molecule (Cromer Blood Group)	CD55		GeneCard	
745	CD58 Molecule	CD58		GeneCard	
746	CD59 Molecule (CD59 Blood Group)	CD59		GeneCard	DisGenet
747	CD5 Antigen-like (scavenger receptor cysteine rich family)	CD5L	OMIM	GeneCard	DisGenet

748	CD63 Molecule	CD63		GeneCard	
749	CD68 Molecule	CD68		GeneCard	DisGenet
750	CD69 Molecule	CD69		GeneCard	DisGenet
751	CD70 Molecule	CD70		GeneCard	DisGenet
752	CD72 Molecule	CD72		GeneCard	
753	CD74 Molecule	CD74		GeneCard	DisGenet
754	CD79a Molecule	CD79A		GeneCard	
755	CD80 Molecule	CD80		GeneCard	DisGenet
756	CD81 Molecule	CD81		GeneCard	DisGenet
757	CD83 Molecule	CD83		GeneCard	
758	CD84 Molecule	CD84		GeneCard	
759	CD86 Molecule	CD86		GeneCard	DisGenet
760	CD8a Molecule	CD8A		GeneCard	
761	CD9 Molecule	CD9		GeneCard	
762	CD99 Molecule (Xg blood group)	CD99			DisGenet
763	Cell Division Cycle 42	CDC42		GeneCard	DisGenet
764	CDC42 Effector Protein 3	CDC42EP3		GeneCard	
765	Cell Division Cycle 5 Like	CDC5L		GeneCard	
766	Cadherin 1	CDH1		GeneCard	DisGenet
767	Cadherin 13	CDH13		GeneCard	DisGenet
768	Cadherin 2	CDH2		GeneCard	
769	Cadherin Related 23	CDH23		GeneCard	
770	Cadherin 4	CDH4		GeneCard	
771	Cadherin 5	CDH5		GeneCard	DisGenet
772	Cadherin 7	CDH7		GeneCard	
773	Cadherin 9	CDH9		GeneCard	
774	Cyclin Dependent Kinase 1	CDK1		GeneCard	
775	Cyclin Dependent Kinase 14	CDK14		GeneCard	
776	Cyclin Dependent Kinase 2	CDK2		GeneCard	DisGenet

777	Cyclin Dependent Kinase 3	CDK3		GeneCard	
778	Cyclin Dependent Kinase 4	CDK4		GeneCard	
779	Cyclin Dependent Kinase 5	CDK5		GeneCard	DisGenet
780	CDK5 Regulatory Subunit Associated Protein 3	CDK5RAP3		GeneCard	
781	Cyclin Dependent Kinase 6	CDK6		GeneCard	
782	Cyclin Dependent Kinase 7	CDK7		GeneCard	
783	Cyclin Dependent Kinase 9	CDK9		GeneCard	DisGenet
784	CDK5 Regulatory Subunit Associated Protein 1 Like 1	CDKAL1		GeneCard	DisGenet
785	Cyclin Dependent Kinase Inhibitor 1A	CDKN1A		GeneCard	DisGenet
786	Cyclin Dependent Kinase Inhibitor 1B	CDKN1B		GeneCard	DisGenet
787	Cyclin Dependent Kinase Inhibitor 1C	CDKN1C		GeneCard	DisGenet
788	Cyclin Dependent Kinase Inhibitor 2A	CDKN2A		GeneCard	DisGenet
789	Cyclin Dependent Kinase Inhibitor 2B	CDKN2B		GeneCard	DisGenet
790	CDKN2B Antisense RNA 1	CDKN2B-AS1		GeneCard	DisGenet
791	Cyclin Dependent Kinase Inhibitor 3	CDKN3		GeneCard	
792	Cysteine Dioxygenase Type 1	CDO1		GeneCard	
793	Cerebellar Degeneration Related Protein 1	CDR1		GeneCard	
794	CDR1 antisense RNA	CDR1-AS			DisGenet
795	CMT1A Duplicated Region Transcript 7	CDRT7		GeneCard	
796	CMT1A Duplicated Region Transcript 8	CDRT8		GeneCard	
797	CEA Cell Adhesion Molecule 1	CEACAM1		GeneCard	DisGenet
798	CEA Cell Adhesion Molecule 3	CEACAM3		GeneCard	
799	CEA Cell Adhesion Molecule 5	CEACAM5		GeneCard	
800	CEA Cell Adhesion Molecule 8	CEACAM8		GeneCard	
801	CCAAT Enhancer Binding Protein Alpha	CEBPA		GeneCard	DisGenet
802	CCAAT Enhancer Binding Protein Beta	CEBPB		GeneCard	DisGenet
803	CCAAT Enhancer Binding Protein Delta	CEBPD		GeneCard	
804	CCAAT Enhancer Binding Protein Zeta	CEBPZ		GeneCard	
805	Carboxyl Ester Lipase	CEL		GeneCard	DisGenet

806	Chymotrypsin-like elastase family, member 2A	CELA2A	OMIM	GeneCard	DisGenet
807	Cadherin EGF LAG Seven-Pass G-Type Receptor 1	CELSR1		GeneCard	
808	Cadherin EGF LAG Seven-Pass G-Type Receptor 2	CELSR2		GeneCard	
809	Cell Migration Inducing Hyaluronidase 1	CEMIP		GeneCard	
810	Centromere Protein A	CENPA		GeneCard	
811	Centromere Protein B	CENPB		GeneCard	
812	Centrosomal Protein 19	CEP19		GeneCard	
813	Centrosomal Protein 290	CEP290		GeneCard	
814	Centrosomal Protein 295	CEP295		GeneCard	
815	Centrosomal Protein 43	CEP43		GeneCard	
816	Centrosomal Protein 85 Like	CEP85L		GeneCard	
817	Ceramide Synthase 5	CERS5		GeneCard	
818	Carboxylesterase 1	CES1		GeneCard	DisGenet
819	Carboxylesterase 2	CES2		GeneCard	
820	Carboxylesterase 3	CES3			DisGenet
821	Cholesteryl Ester Transfer Protein	CETP		GeneCard	DisGenet
822	Cilia And Flagella Associated Protein 47	CFAP47		GeneCard	
823	Cilia And Flagella Associated Protein 91	CFAP91		GeneCard	
824	Complement Factor B	CFB		GeneCard	
825	Complement Factor D	CFD		GeneCard	
826	Craniofacial Development Protein 1	CFDP1		GeneCard	DisGenet
827	Complement factor H	CFH	OMIM	GeneCard	DisGenet
828	Complement Factor H Related 1	CFHR1		GeneCard	
829	Complement Factor H Related 2	CFHR2		GeneCard	
830	Complement Factor I	CFI		GeneCard	
831	Cofilin 1	CFL1		GeneCard	
832	Cofilin 2	CFL2		GeneCard	
833	CASP8 And FADD Like Apoptosis Regulator	CFLAR		GeneCard	
834	Complement Factor Properdin	CFP		GeneCard	

835	CF Transmembrane Conductance Regulator	CFTR		GeneCard	
836	Glycoprotein Hormones, Alpha Polypeptide	CGA		GeneCard	
837	Cholesterol 25-Hydroxylase	CH25H		GeneCard	DisGenet
838	ChaC Glutathione Specific Gamma-Glutamylcyclotransferase 1	CHAC1		GeneCard	
839	Chondroadherin	CHAD		GeneCard	
840	Choline O-Acetyltransferase	CHAT		GeneCard	
841	Coiled-Coil-Helix-Coiled-Coil-Helix Domain Containing 10	CHCHD10		GeneCard	
842	Chromodomain Helicase DNA Binding Protein 1	CHD1		GeneCard	
843	Chromodomain Helicase DNA Binding Protein 3	CHD3		GeneCard	
844	Chromodomain Helicase DNA Binding Protein 4	CHD4		GeneCard	
845	Chromodomain Helicase DNA Binding Protein 7	CHD7		GeneCard	
846	Choline Dehydrogenase	CHDH			DisGenet
847	Coronary Heart Disease, Susceptibility To, 1	CHDS1		GeneCard	
848	Coronary Heart Disease, Susceptibility To, 2	CHDS2		GeneCard	
849	Coronary Heart Disease, Susceptibility To, 3	CHDS3		GeneCard	
850	Coronary Heart Disease, Susceptibility To, 4	CHDS4		GeneCard	
851	Coronary Heart Disease, Susceptibility To, 8	CHDS8		GeneCard	
852	Coronary Heart Disease, Susceptibility To, 9	CHDS9		GeneCard	
853	Checkpoint Kinase 2	CHEK2		GeneCard	
854	Checkpoint With Forkhead and Ring Finger Domains	CHFR		GeneCard	
855	Chromogranin A	CHGA		GeneCard	DisGenet
856	Chitinase 3 Like 1	CHI3L1		GeneCard	DisGenet
857	Chitinase 1	CHIT1		GeneCard	DisGenet
858	Choline Kinase Alpha	CHKA		GeneCard	
859	Choline Kinase Beta	CHKB		GeneCard	
860	Charged Multivesicular Body Protein 2B	CHMP2B		GeneCard	DisGenet
861	Choline Phosphotransferase 1	CHPT1		GeneCard	
862	Cholinergic Receptor Muscarinic 1	CHRM1		GeneCard	
863	Cholinergic Receptor Muscarinic 2	CHRM2		GeneCard	

864	Cholinergic Receptor Muscarinic 3	CHRM3		GeneCard	DisGenet
865	Cholinergic Receptor Nicotinic Alpha 2 Subunit	CHRNA2		GeneCard	
866	Cholinergic Receptor Nicotinic Alpha 3 Subunit	CHRNA3		GeneCard	
867	Cholinergic Receptor Nicotinic Alpha 4 subunit	CHRNA4			DisGenet
868	Cholinergic Receptor Nicotinic Alpha 5 Subunit	CHRNA5		GeneCard	DisGenet
869	Cholinergic Receptor Nicotinic Alpha 7 Subunit	CHRNA7		GeneCard	
870	Cholinergic Receptor Nicotinic Beta 4 Subunit	CHRNB4		GeneCard	
871	Cholesterol Induced Regulator of Metabolism RNA	CHROMR		GeneCard	
872	Carbohydrate Sulfotransferase 1	CHST1		GeneCard	
873	Carbohydrate Sulfotransferase 2	CHST2		GeneCard	
874	Carbohydrate Sulfotransferase 3	CHST3		GeneCard	DisGenet
875	Component Of Inhibitor Of Nuclear Factor Kappa B Kinase Complex	CHUK		GeneCard	
876	Cytokine Induced Apoptosis Inhibitor 1	CIAPIN1		GeneCard	
877	Calcium And Integrin Binding Family Member 4	CIB4		GeneCard	
878	Cell Death Inducing DFFA Like Effector C	CIDEC		GeneCard	DisGenet
879	Class II Major Histocompatibility Complex Transactivator	CIITA		GeneCard	
880	Cartilage Intermediate Layer Protein 2	CILP2		GeneCard	
881	Carotid Intimal Medial Thickness	CIMT		GeneCard	DisGenet
882	Cellular inhibitor of PP2A	CIP2A			DisGenet
883	CDGSH Iron Sulfur Domain 1	CISD1			DisGenet
884	Cytokine Inducible SH2 Containing Protein	CISH		GeneCard	DisGenet
885	Citron Rho-Interacting Serine/Threonine Kinase	CIT			DisGenet
886	Cbp/P300 Interacting Transactivator with Glu/Asp Rich Carboxy-Terminal Domain 2	CITED2		GeneCard	
887	Creatine Kinase B	CKB		GeneCard	
888	Chemokine Like Factor	CKLF		GeneCard	
889	Creatine Kinase, M-Type	CKM		GeneCard	
890	Creatine Kinase, Mitochondrial 2	CKMT2		GeneCard	
891	CLK4 Associating Serine/Arginine Rich Protein	CLASRP		GeneCard	
892	Chloride Channel CLIC Like 1	CLCC1		GeneCard	

893	Cardiotrophin Like Cytokine Factor 1	CLCF1			DisGenet
894	Chloride Voltage-Gated Channel 1	CLCN1		GeneCard	
895	Chloride Voltage-Gated Channel 2	CLCN2			DisGenet
896	Claudin 1	CLDN1			DisGenet
897	Claudin 5	CLDN5		GeneCard	
898	Claudin 7	CLDN7			DisGenet
899	C-Type Lectin Domain Family 12 Member A	CLEC12A		GeneCard	
900	C-Type Lectin Domain Containing 16A	CLEC16A		GeneCard	
901	C-Type Lectin Domain Family 1 Member A	CLEC1A		GeneCard	
902	C-Type Lectin Domain Family 3 Member B	CLEC3B		GeneCard	
903	C-Type Lectin Domain Family 4 Member A	CLEC4A		GeneCard	
904	C-Type Lectin Domain Family 4 Member C	CLEC4C		GeneCard	
905	C-type Lectin Domain Family 4 Member D	CLEC4D			DisGenet
906	C-Type Lectin Domain Family 4 Member E	CLEC4E		GeneCard	DisGenet
907	C-Type Lectin Domain Containing 5A	CLEC5A		GeneCard	DisGenet
908	C-Type Lectin Domain Containing 6A	CLEC6A		GeneCard	DisGenet
909	C-Type Lectin Domain Containing 7A	CLEC7A		GeneCard	DisGenet
910	C-type Lectin Domain Containing 9A	CLEC9A			DisGenet
911	Calmegin	CLGN		GeneCard	
912	Chloride Intracellular Channel 1	CLIC1			DisGenet
913	Chloride Intracellular Channel 4	CLIC4		GeneCard	DisGenet
914	Chloride Intracellular Channel 5	CLIC5		GeneCard	
915	Clathrin Interactor 1	CLINT1		GeneCard	
916	CAP-Gly Domain Containing Linker Protein 1	CLIP1		GeneCard	
917	Clock Circadian Regulator	CLOCK		GeneCard	DisGenet
918	Caseinolytic Mitochondrial Matrix Peptidase Proteolytic Subunit	CLPP		GeneCard	
919	Colipase	CLPS		GeneCard	
920	CLPTM1 Like	CLPTM1L		GeneCard	
921	Clathrin Heavy Chain	CLTC		GeneCard	

922	Clathrin Heavy Chain Like 1	CLTCL1		GeneCard	
923	Clusterin (complement lysis inhibitor, SP-40,40; sulfated glycoprotein 2; testosterone-repressed prostate message-2; apolipoprotein J)	CLU	OMIM	GeneCard	DisGenet
924	Chymase 1	CMA1		GeneCard	DisGenet
925	Chemerin Chemokine-Like Receptor 1	CMKLR1		GeneCard	DisGenet
926	Cytidine/Uridine Monophosphate Kinase 2	CMPK2		GeneCard	
927	CKLF Like MARVEL Transmembrane Domain Containing 5	CMTM5		GeneCard	
928	CKLF Like MARVEL Transmembrane Domain Containing 7	CMTM7		GeneCard	
929	CCHC-type zinc finger nucleic acid binding protein	CNBP			DisGenet
930	Chondromodulin	CNMD		GeneCard	
931	Calponin 1	CNN1		GeneCard	
932	Calponin 2	CNN2			DisGenet
933	CCR4-NOT Transcription Complex Subunit 3	CNOT3		GeneCard	
934	2',3'-cyclic nucleotide 3' phosphodiesterase	CNP			DisGenet
935	Cannabinoid Receptor 1	CNR1		GeneCard	DisGenet
936	Cannabinoid receptor 2	CNR2	OMIM	GeneCard	DisGenet
937	Centlein	CNTLN		GeneCard	
938	Contactin 1	CNTN1		GeneCard	
939	Contactin 3	CNTN3			DisGenet
940	Contactin 5	CNTN5		GeneCard	
941	Contactin Associated Protein 2	CNTNAP2		GeneCard	
942	Component Of Oligomeric Golgi Complex 2	COG2		GeneCard	
943	Collagen Type X Alpha 1 Chain	COL10A1		GeneCard	
944	Collagen Type XV Alpha 1 Chain	COL15A1	OMIM	GeneCard	DisGenet
945	Collagen Type XVIII Alpha 1 Chain	COL18A1		GeneCard	DisGenet
946	Collagen Type I Alpha 1 Chain	COL1A1		GeneCard	DisGenet
947	Collagen Type I Alpha 2 Chain	COL1A2		GeneCard	
948	Collagen Type XX Alpha 1 Chain	COL20A1		GeneCard	
949	Collagen Type II Alpha 1 Chain	COL2A1		GeneCard	

950	Collagen Type III Alpha 1 Chain	COL3A1		GeneCard	DisGenet
951	Collagen Type IV Alpha 1 Chain	COL4A1	OMIM	GeneCard	DisGenet
952	Collagen Type IV Alpha 2 Chain	COL4A2		GeneCard	
953	COL4A2 Antisense RNA 2	COL4A2-AS2		GeneCard	
954	Collagen Type IV Alpha 3 Chain	COL4A3		GeneCard	
955	Collagen Type IV Alpha 4 Chain	COL4A4		GeneCard	
956	Collagen Type V Alpha 1 Chain	COL5A1		GeneCard	
957	Collagen Type V Alpha 2 Chain	COL5A2		GeneCard	
958	Collagen Type V Alpha 3 Chain	COL5A3		GeneCard	
959	Collagen Type VI Alpha 1 Chain	COL6A1		GeneCard	
960	Collagen Type VIII Alpha 1 Chain	COL8A1		GeneCard	
961	Collectin Subfamily Member 10	COLEC10		GeneCard	
962	Collectin Subfamily Member 12	COLEC12		GeneCard	DisGenet
963	COMM Domain Containing 10	COMMD10		GeneCard	
964	COMM Domain Containing 6	COMMD6		GeneCard	
965	Cartilage Oligomeric Matrix Protein	COMP		GeneCard	DisGenet
966	Catechol-O-Methyltransferase	COMT		GeneCard	DisGenet
967	COPI coat complex subunit beta 2	COPB2			DisGenet
968	Pulmonary disease, chronic obstructive, severe early-onset	COPD			DisGenet
969	COPI coat complex subunit epsilon	COPE			DisGenet
970	COP9 Signalosome Subunit 2	COPS2		GeneCard	
971	COP9 Signalosome Subunit 5	COPS5		GeneCard	DisGenet
972	Coenzyme Q10A	COQ10A		GeneCard	
973	Corin, Serine Peptidase	CORIN		GeneCard	
974	Coronin 1B	CORO1B		GeneCard	
975	Cortistatin	CORT		GeneCard	DisGenet
976	Cytochrome C Oxidase Subunit I	COX1			DisGenet
977	Cytochrome C Oxidase Copper Chaperone COX17	COX17		GeneCard	
978	Cytochrome C Oxidase Subunit II	COX2			DisGenet

979	Cytochrome C Oxidase Subunit 5A	COX5A		GeneCard	DisGenet
980	Cytochrome C Oxidase Subunit 8A	COX8A			DisGenet
981	Ceruloplasmin	CP		GeneCard	DisGenet
982	Carboxypeptidase A1	CPA1		GeneCard	
983	Carboxypeptidase A3	CPA3		GeneCard	
984	Carboxypeptidase A6	CPA6		GeneCard	
985	Cerebral Palsy, Ataxic 1	CPAT1			DisGenet
986	Carboxypeptidase B1	CPB1		GeneCard	
987	Carboxypeptidase B2	CPB2		GeneCard	DisGenet
988	Carboxypeptidase E	CPE		GeneCard	
989	Cytoplasmic Polyadenylation Element Binding Protein 1	CPEB1		GeneCard	
990	Cytoplasmic Polyadenylation Element Binding Protein 2	CPEB2		GeneCard	
991	Copine 1	CPNE1		GeneCard	
992	Carboxypeptidase O	CPO		GeneCard	
993	Coproporphyrinogen Oxidase	CPOX		GeneCard	DisGenet
994	Carboxypeptidase Q	CPQ		GeneCard	
995	Carbamoyl-Phosphate Synthase 1	CPS1	OMIM	GeneCard	
996	Carnitine Palmitoyltransferase 1A	CPT1A		GeneCard	
997	Carnitine Palmitoyltransferase 2	CPT2		GeneCard	DisGenet
998	Ceramide-1-Phosphate Transfer Protein	CPTP		GeneCard	
999	Complement C3b/C4b Receptor 1 (Knops Blood Group)	CR1		GeneCard	DisGenet
1000	Complement C3d Receptor 2	CR2		GeneCard	
1001	Carnitine O-Acetyltransferase	CRAT		GeneCard	
1002	Crumbs Cell Polarity Complex Component 1	CRB1		GeneCard	
1003	CAMP Responsive Element Binding Protein 1	CREB1		GeneCard	DisGenet
1004	CAMP Responsive Element Binding Protein 3	CREB3		GeneCard	DisGenet

100<br>5	CAMP Responsive Element Binding Protein 3 Like 3	CREB3L3		GeneCard	DisGenet
100<br>6	CREB Binding Protein	CREBBP		GeneCard	
100<br>7	Cellular Repressor Of E1A Stimulated Genes 1	CREG1	OMIM	GeneCard	DisGenet
100<br>8	CAMP Responsive Element Modulator	CREM		GeneCard	
100<br>9	Corticotropin Releasing Hormone	CRH		GeneCard	
101<br>0	Cysteine Rich Secretory Protein 2	CRISP2		GeneCard	DisGenet
101<br>1	CRK proto-oncogene, adaptor protein	CRK			DisGenet
101<br>2	Cytokine Receptor Like Factor 2	CRLF2		GeneCard	
101<br>3	Collapsin Response Mediator Protein 1	CRMP1			DisGenet
101<br>4	Crooked Neck Pre-MRNA Splicing Factor 1	CRNKL1		GeneCard	
101<br>5	Ciliary Rootlet Coiled-Coil, Rootletin	CROCC		GeneCard	
101<br>6	Carnitine O-Octanoyltransferase	CROT		GeneCard	
101<br>7	C-Reactive Protein	CRP	OMIM	GeneCard	DisGenet
101<br>8	CRTC1 Pseudogene 1	CRTC1P1		GeneCard	
101<br>9	Cryptochrome Circadian Regulator 1	CRY1		GeneCard	DisGenet
102<br>0	Cryptochrome Circadian Regulator 2	CRY2		GeneCard	
102<br>1	Crystallin Alpha A	CRYAA		GeneCard	
102<br>2	Crystallin Alpha B	CRYAB		GeneCard	
102<br>3	Crystallin Gamma D	CRYGD			DisGenet
102<br>4	Citrate Synthase	CS		GeneCard	

102 5	Chromosome Segregation 1 Like	CSE1L		GeneCard	
102 6	Colony Stimulating Factor 1	CSF1	OMIM	GeneCard	DisGenet
102 7	Colony Stimulating Factor 1 Receptor	CSF1R		GeneCard	DisGenet
102 8	Colony Stimulating Factor 2	CSF2		GeneCard	DisGenet
102 9	Colony Stimulating Factor 2 Receptor Subunit Alpha	CSF2RA		GeneCard	
103 0	Colony Stimulating Factor 2 Receptor Subunit Beta	CSF2RB		GeneCard	DisGenet
103 1	Colony Stimulating Factor 3	CSF3		GeneCard	DisGenet
103 2	Colony Stimulating Factor 3 Receptor	CSF3R		GeneCard	
103 3	Chondroitin Sulfate N-Acetylgalactosaminyltransferase 1	CSGALNACT1		GeneCard	
103 4	Chondroitin Sulfate N-Acetylgalactosaminyltransferase 2	CSGALNACT2		GeneCard	DisGenet
103 5	C-Terminal Src Kinase	CSK		GeneCard	
103 6	CUB and Sushi Multiple Domains 1	CSMD1		GeneCard	
103 7	CUB and Sushi Multiple Domains 2	CSMD2		GeneCard	
103 8	CUB and Sushi Multiple Domains 3	CSMD3		GeneCard	
103 9	Casein Alpha S1	CSN1S1		GeneCard	
104 0	Casein Kinase 2 Alpha 1	CSNK2A1		GeneCard	
104 1	Casein Kinase 2 Alpha 2	CSNK2A2		GeneCard	
104 2	Casein Kinase 2 Beta	CSNK2B		GeneCard	
104 3	Cysteine And Glycine Rich Protein 1	CSRP1		GeneCard	
104 4	Cysteine And Glycine Rich Protein 3	CSRP3		GeneCard	

1045	Cystatin 12, Pseudogene	CST12P			DisGenet
1046	Cystatin C	CST3		GeneCard	DisGenet
1047	Cystatin S	CST4		GeneCard	
1048	Cystatin A	CSTA		GeneCard	
1049	Cystatin B	CSTB		GeneCard	
1050	Cleavage Stimulation Factor Subunit 3	CSTF3		GeneCard	
1051	Chitobiase	CTBS			DisGenet
1052	CCCTC-Binding Factor	CTCF		GeneCard	DisGenet
1053	Cardiotrophin 1	CTF1		GeneCard	DisGenet
1054	Cystathionine Gamma-Lyase	CTH		GeneCard	DisGenet
1055	Cytotoxic T-Lymphocyte Associated Protein 4	CTLA4		GeneCard	
1056	Catenin Alpha 1	CTNNA1		GeneCard	
1057	Catenin Alpha 2	CTNNA2		GeneCard	
1058	Catenin Alpha 3	CTNNA3		GeneCard	
1059	Catenin Beta 1	CTNNB1		GeneCard	DisGenet
1060	Catenin Beta Interacting Protein 1	CTNNBIP1		GeneCard	
1061	Catenin delta 1	CTNND1			DisGenet
1062	Catenin Delta 2	CTNND2		GeneCard	
1063	Cystinosin, Lysosomal Cystine Transporter	CTNS		GeneCard	
1064	CTP synthase 1	CTPS1			DisGenet

106<br>5	Chymotrypsin C	CTRC		GeneCard	
106<br>6	Chymotrypsin Like	CTRL		GeneCard	
106<br>7	Cathepsin A	CTSA		GeneCard	
106<br>8	Cathepsin B	CTSB		GeneCard	DisGenet
106<br>9	Cathepsin C	CTSC		GeneCard	DisGenet
107<br>0	Cathepsin D	CTSD		GeneCard	DisGenet
107<br>1	Cathepsin F	CTSF		GeneCard	
107<br>2	Cathepsin G	CTSG		GeneCard	DisGenet
107<br>3	Cathepsin H	CTSH		GeneCard	
107<br>4	Cathepsin K	CTSK		GeneCard	DisGenet
107<br>5	Cathepsin L	CTSL		GeneCard	DisGenet
107<br>6	Cathepsin S	CTSS		GeneCard	DisGenet
107<br>7	Cathepsin V	CTSV		GeneCard	
107<br>8	Cortactin	CTTN		GeneCard	
107<br>9	Cubilin	CUBN		GeneCard	
108<br>0	Cullin 4A	CUL4A		GeneCard	
108<br>1	Cullin 4B	CUL4B		GeneCard	
108<br>2	Cullin 5	CUL5		GeneCard	
108<br>3	CWC22 Spliceosome Associated Protein Homolog	CWC22		GeneCard	
108<br>4	C-X3-C Motif Chemokine Ligand 1	CX3CL1		GeneCard	DisGenet

108<br>5	Chemokine (C-X3-C) receptor 1 (G protein-coupled receptor-13)	CX3CR1	OMIM	GeneCard	DisGenet
108<br>6	CXADR Ig-Like Cell Adhesion Molecule	CXADR		GeneCard	DisGenet
108<br>7	CXADRP1	CXADRP1			DisGenet
108<br>8	C-X-C Motif Chemokine Ligand 1	CXCL1		GeneCard	DisGenet
108<br>9	C-X-C Motif Chemokine Ligand 10	CXCL10		GeneCard	DisGenet
109<br>0	C-X-C Motif Chemokine Ligand 11	CXCL11		GeneCard	
109<br>1	C-X-C Motif Chemokine Ligand 12	CXCL12		GeneCard	DisGenet
109<br>2	C-X-C Motif Chemokine Ligand 13	CXCL13		GeneCard	DisGenet
109<br>3	C-X-C Motif Chemokine Ligand 14	CXCL14		GeneCard	DisGenet
109<br>4	C-X-C Motif Chemokine Ligand 16	CXCL16		GeneCard	DisGenet
109<br>5	C-X-C Motif Chemokine Ligand 17	CXCL17		GeneCard	
109<br>6	C-X-C Motif Chemokine Ligand 2	CXCL2		GeneCard	DisGenet
109<br>7	C-X-C Motif Chemokine Ligand 3	CXCL3		GeneCard	
109<br>8	C-X-C Motif Chemokine Ligand 5	CXCL5		GeneCard	
109<br>9	C-X-C Motif Chemokine Ligand 6	CXCL6		GeneCard	
110<br>0	C-X-C Motif Chemokine Ligand 8	CXCL8		GeneCard	DisGenet
110<br>1	C-X-C Motif Chemokine Ligand 9	CXCL9		GeneCard	DisGenet
110<br>2	C-X-C Motif Chemokine Receptor 1	CXCR1		GeneCard	
110<br>3	C-X-C Motif Chemokine Receptor 2	CXCR2		GeneCard	DisGenet
110<br>4	C-X-C Motif Chemokine Receptor 3	CXCR3		GeneCard	DisGenet

110 5	C-X-C Motif Chemokine Receptor 4	CXCR4		GeneCard	DisGenet
110 6	C-X-C Motif Chemokine Receptor 5	CXCR5		GeneCard	
110 7	C-X-C Motif Chemokine Receptor 6	CXCR6		GeneCard	DisGenet
110 8	Chromosome X Open Reading Frame 38	CXorf38		GeneCard	
110 9	Chromosome X Open Reading Frame 49	CXorf49		GeneCard	
111 0	Cytochrome B-245 Alpha Chain	CYBA		GeneCard	DisGenet
111 1	Cytochrome B-245 Beta Chain	CYBB		GeneCard	DisGenet
111 2	Cytochrome C, Somatic	CYCS		GeneCard	
111 3	CYCS Pseudogene 14	CYCSP14		GeneCard	
111 4	CYCS Pseudogene 42	CYCSP42		GeneCard	
111 5	CYCS Pseudogene 8	CYCSP8		GeneCard	
111 6	Cytoglobin	CYGB		GeneCard	DisGenet
111 7	CYLD Lysine 63 Deubiquitinase	CYLD		GeneCard	DisGenet
111 8	Cytochrome P450 Family 11 Subfamily A Member 1	CYP11A1		GeneCard	
111 9	Cytochrome P450 Family 11 Subfamily B Member 2	CYP11B2		GeneCard	
112 0	Cytochrome P450 Family 17 Subfamily A Member 1	CYP17A1		GeneCard	
112 1	Cytochrome P450 Family 19 Subfamily A Member 1	CYP19A1		GeneCard	DisGenet
112 2	Cytochrome P450 Family 1 Subfamily A Member 1	CYP1A1		GeneCard	DisGenet
112 3	Cytochrome P450 Family 1 Subfamily A Member 2	CYP1A2		GeneCard	DisGenet
112 4	Cytochrome P450 Family 1 Subfamily B Member 1	CYP1B1		GeneCard	

1125	Cytochrome P450 Family 20 Subfamily A Member 1	CYP20A1		GeneCard	
1126	Cytochrome P450 Family 21 Subfamily A Member 2	CYP21A2		GeneCard	
1127	Cytochrome P450 Family 24 Subfamily A Member 1	CYP24A1		GeneCard	
1128	Cytochrome P450 Family 26 Subfamily A Member 1	CYP26A1			DisGenet
1129	Cytochrome P450 Family 26 Subfamily B Member 1	CYP26B1		GeneCard	DisGenet
1130	Cytochrome P450 Family 27 Subfamily B Member 1	CYP27A1	OMIM	GeneCard	DisGenet
1131	Cytochrome P450 Family 27 Subfamily B Member 1	CYP27B1		GeneCard	DisGenet
1132	Cytochrome P450 Family 27 Subfamily C Member 1	CYP27C1		GeneCard	
1133	Cytochrome P450 Family 2 Subfamily B Member 6	CYP2B6		GeneCard	DisGenet
1134	Cytochrome P450 Family 2 Subfamily C Member 19	CYP2C19		GeneCard	DisGenet
1135	Cytochrome P450 Family 2 Subfamily C Member 8	CYP2C8		GeneCard	DisGenet
1136	Cytochrome P450 Family 2 Subfamily C Member 9	CYP2C9		GeneCard	DisGenet
1137	Cytochrome P450 Family 2 Subfamily D Member 6	CYP2D6			DisGenet
1138	Cytochrome P450 Family 2 Subfamily E Member 1	CYP2E1		GeneCard	DisGenet
1139	Cytochrome P450 Family 2 Subfamily J Member 2	CYP2J2		GeneCard	DisGenet
1140	Cytochrome P450 Family 2 Subfamily R Member 1	CYP2R1		GeneCard	
1141	Cytochrome P450 Family 3 Subfamily A Member 4	CYP3A4		GeneCard	DisGenet
1142	Cytochrome P450 Family 3 Subfamily A Member 5	CYP3A5		GeneCard	DisGenet
1143	Cytochrome P450 Family 46 Subfamily A Member 1	CYP46A1		GeneCard	
1144	Cytochrome P450 Family 4 Subfamily A Member 11	CYP4A11		GeneCard	

1145	Cytochrome P450 Family 4 Subfamily F Member 2	CYP4F2		GeneCard	DisGenet
1146	Cytochrome P450 Family 4 Subfamily V Member 2	CYP4V2		GeneCard	
1147	Cytochrome P450 Family 51 Subfamily A Member 1	CYP51A1		GeneCard	
1148	Cytochrome P450 Family 7 Subfamily A Member 1	CYP7A1		GeneCard	DisGenet
1149	Cytochrome P450 Family 7 Subfamily B Member 1	CYP7B1		GeneCard	
1150	Cytochrome P450 Family 8 Subfamily B Member 1	CYP8B1		GeneCard	
1151	Cysteinyl Leukotriene Receptor 1	CYSLTR1		GeneCard	DisGenet
1152	Cysteinyl Leukotriene Receptor 2	CYSLTR2		GeneCard	
1153	Cytochrome B	CYTB			DisGenet
1154	DAB Adaptor Protein 2	DAB2		GeneCard	DisGenet
1155	DAB2 Interacting Protein	DAB2IP		GeneCard	DisGenet
1156	Dachshund Family Transcription Factor 2	DACH2		GeneCard	
1157	Dystroglycan 1	DAG1		GeneCard	DisGenet
1158	Diacylglycerol Lipase Beta	DAGLB		GeneCard	
1159	Differentiation Antagonizing Non-Protein Coding RNA	DANCR		GeneCard	
1160	D-Amino Acid Oxidase Activator	DAOA		GeneCard	
1161	Death Associated Protein	DAP		GeneCard	
1162	Death Associated Protein Kinase 1	DAPK1		GeneCard	
1163	DAPK1 Intronic Transcript 1	DAPK1-IT1		GeneCard	
1164	Death Associated Protein Kinase 2	DAPK2			DisGenet

116 5	Death Associated Protein Kinase 3	DAPK3		GeneCard	
116 6	Death Domain Associated Protein	DAXX		GeneCard	
116 7	Deleted In Azoospermia Like	DAZL		GeneCard	
116 8	Diazepam Binding Inhibitor, Acyl-CoA Binding Protein	DBI		GeneCard	
116 9	Drebrin 1	DBN1		GeneCard	DisGenet
117 0	D-box binding PAR bZIP transcription factor	DBP			DisGenet
117 1	Discoidin, CUB And LCCL Domain Containing 1	DCBLD1		GeneCard	
117 2	Dermcidin	DCD		GeneCard	
117 3	Doublecortin Domain Containing 2C	DCDC2C		GeneCard	
117 4	Doublecortin Like Kinase 3	DCLK3			DisGenet
117 5	Decorin	DCN		GeneCard	DisGenet
117 6	Dynactin Subunit 4	DCTN4			DisGenet
117 7	Dynactin Subunit 6	DCTN6			DisGenet
117 8	Dimethylarginine Dimethylaminohydrolase 1	DDAH1		GeneCard	DisGenet
117 9	Dimethylarginine Dimethylaminohydrolase 2	DDAH2		GeneCard	DisGenet
118 0	Damage Specific DNA Binding Protein 1	DDB1		GeneCard	
118 1	Damage Specific DNA Binding Protein 2	DDB2		GeneCard	
118 2	Dopa Decarboxylase	DDC		GeneCard	
118 3	DDHD Domain Containing 1	DDHD1		GeneCard	
118 4	DNA Damage Inducible Transcript 3	DDIT3		GeneCard	DisGenet

1185	DNA Damage Inducible Transcript 4 Like	DDIT4L		GeneCard	
1186	Discoidin Domain Receptor Tyrosine Kinase 1	DDR1		GeneCard	DisGenet
1187	Discoidin Domain Receptor Tyrosine Kinase 2	DDR2		GeneCard	DisGenet
1188	DEAD-Box Helicase 4	DDX4		GeneCard	
1189	DExD/H-Box Helicase 58	DDX58		GeneCard	
1190	2,4-dienoyl-CoA reductase 1	DECR1			DisGenet
1191	Defensin Alpha 1	DEFA1		GeneCard	
1192	Defensin Alpha 3	DEFA3		GeneCard	
1193	Defensin Beta 103A	DEFB103A			DisGenet
1194	Defensin Beta 103B	DEFB103B		GeneCard	DisGenet
1195	Defensin Beta 128	DEFB128		GeneCard	
1196	Defensin Beta 4A	DEFB4A		GeneCard	DisGenet
1197	Defensin Beta 4B	DEFB4B			DisGenet
1198	Delta 4-Desaturase, Sphingolipid 1	DEGS1		GeneCard	
1199	Density Regulated Re-initiation and Release Factor	DENR			DisGenet
1200	Desmin	DES		GeneCard	
1201	Deuterosome Assembly Protein 1	DEUP1		GeneCard	
1202	DNA Fragmentation Factor Subunit Beta	DFFB		GeneCard	
1203	Diacylglycerol O-Acyltransferase 1	DGAT1		GeneCard	DisGenet
1204	Diacylglycerol O-Acyltransferase 2	DGAT2		GeneCard	

1205	Diacylglycerol O-Acyltransferase 2 Like 6	DGAT2L6		GeneCard	
1206	Diacylglycerol O-Acyltransferase 2 Like 7, Pseudogene	DGAT2L7P		GeneCard	
1207	Diacylglycerol Kinase Alpha	DGKA		GeneCard	
1208	Diacylglycerol Kinase Epsilon	DGKE		GeneCard	
1209	Diacylglycerol Kinase Eta	DGKH		GeneCard	
1210	Diacylglycerol Kinase Theta	DGKQ		GeneCard	
1211	24-Dehydrocholesterol Reductase	DHCR24		GeneCard	DisGenet
1212	7-Dehydrocholesterol Reductase	DHCR7		GeneCard	DisGenet
1213	Dihydrofolate Reductase	DHFR		GeneCard	
1214	DEAH-Box Helicase 15	DHX15		GeneCard	
1215	DEAH-Box Helicase 38	DHX38		GeneCard	
1216	DEAH-box helicase 40	DHX40			DisGenet
1217	DExH-Box Helicase 9	DHX9		GeneCard	
1218	Diablo IAP-Binding Mitochondrial Protein	DIABLO		GeneCard	
1219	Diaphanous Related Formin 1	DIAPH1		GeneCard	
1220	Diaphanous Related Formin 2	DIAPH2		GeneCard	
1221	Dicer 1, Ribonuclease III	DICER1		GeneCard	
1222	Iodothyronine Deiodinase 2	DIO2		GeneCard	DisGenet
1223	Disco Interacting Protein 2 Homolog A	DIP2A		GeneCard	
1224	Dickkopf WNT Signaling Pathway Inhibitor 1	DKK1		GeneCard	DisGenet

122<br>5	Dickkopf WNT Signaling Pathway Inhibitor 3	DKK3		GeneCard	DisGenet
122<br>6	DLC1 Rho GTPase Activating Protein	DLC1		GeneCard	DisGenet
122<br>7	Dihydrolipoamide Dehydrogenase	DLD			DisGenet
122<br>8	DLEC1 Cilia and Flagella Associated Protein	DLEC1			DisGenet
122<br>9	Deleted In Lymphocytic Leukemia 1	DLEU1		GeneCard	
123<br>0	Deleted In Lymphocytic Leukemia 7	DLEU7		GeneCard	
123<br>1	Discs Large MAGUK Scaffold Protein 2	DLG2		GeneCard	
123<br>2	DLG Associated Protein 1	DLGAP1		GeneCard	
123<br>3	Delta Like Canonical Notch Ligand 4	DLL4		GeneCard	DisGenet
123<br>4	Distal-Less Homeobox 5	DLX5		GeneCard	
123<br>5	Distal Membrane Arm Assembly Component 2 Like	DMAC2L		GeneCard	
123<br>6	DNA Meiotic Recombinase 1	DMC1		GeneCard	
123<br>7	Dystrophin	DMD		GeneCard	DisGenet
123<br>8	Dentin Matrix Acidic Phosphoprotein 1	DMP1		GeneCard	
123<br>9	DM1 Protein Kinase	DMPK		GeneCard	
124<br>0	Doublesex And Mab-3 Related Transcription Factor 1	DMRT1		GeneCard	DisGenet
124<br>1	Doublesex And Mab-3 Related Transcription Factor 3	DMRT3		GeneCard	
124<br>2	DMRT Like Family A1	DMRTA1		GeneCard	
124<br>3	DM1 Locus, WD Repeat Containing	DMWD		GeneCard	
124<br>4	Dynein Axonemal Heavy Chain 10	DNAH10		GeneCard	

1245	Dynein Axonemal Heavy Chain 5	DNAH5		GeneCard	
1246	Dynein Axonemal Heavy Chain 8	DNAH8		GeneCard	DisGenet
1247	Dynein Axonemal Heavy Chain 9	DNAH9		GeneCard	
1248	DnaJ Heat Shock Protein Family (Hsp40) Member C19	DNAJC19		GeneCard	
1249	Deoxyribonuclease 1	DNASE1		GeneCard	DisGenet
1250	Deoxyribonuclease 1 Like 3	DNASE1L3		GeneCard	
1251	Delta/Notch Like EGF Repeat	DNER			DisGenet
1252	Dynamin 1 Like	DNM1L		GeneCard	DisGenet
1253	Dynamin 2	DNM2		GeneCard	
1254	Dynamin 3	DNM3		GeneCard	
1255	DNA Methyltransferase 1	DNMT1		GeneCard	DisGenet
1256	DNA Methyltransferase 3 Alpha	DNMT3A		GeneCard	
1257	DNA Methyltransferase 3 Beta	DNMT3B		GeneCard	DisGenet
1258	Dedicator Of Cytokinesis 1	DOCK1		GeneCard	
1259	Dedicator Of Cytokinesis 2	DOCK2		GeneCard	
1260	Dedicator Of Cytokinesis 4	DOCK4	OMIM	GeneCard	DisGenet
1261	Dedicator Of Cytokinesis 6	DOCK6		GeneCard	
1262	Dedicator Of Cytokinesis 7	DOCK7		GeneCard	
1263	Dedicator Of Cytokinesis 9	DOCK9		GeneCard	
1264	Dolichol Kinase	DOLK		GeneCard	

1265	DOT1 Like Histone Lysine Methyltransferase	DOT1L		GeneCard	
1266	Diphthamide Biosynthesis 3	DPH3		GeneCard	
1267	Dolichyl-Phosphate Mannosyltransferase Subunit 3, Regulatory	DPM3		GeneCard	
1268	Dipeptidyl Peptidase 4	DPP4		GeneCard	DisGenet
1269	Dipeptidyl Peptidase 8	DPP8		GeneCard	DisGenet
1270	Dipeptidyl Peptidase 9	DPP9		GeneCard	DisGenet
1271	Developmental Pluripotency Associated 2	DPPA2		GeneCard	
1272	Developmental Pluripotency Associated 3	DPPA3		GeneCard	
1273	Divergent-Paired Related Homeobox Pseudogene 7	DPRXP7		GeneCard	
1274	Dopamine Receptor D2	DRD2		GeneCard	
1275	Desmocollin 1	DSC1			DisGenet
1276	Desmocollin 2	DSC2		GeneCard	
1277	DS Cell Adhesion Molecule Like 1	DSCAML1		GeneCard	
1278	Down Syndrome Critical Region 10	DSCR10		GeneCard	
1279	Desmoglein 2	DSG2		GeneCard	
1280	DSG2 Antisense RNA 1	DSG2-AS1		GeneCard	
1281	Desmoplakin	DSP		GeneCard	
1282	Dentin Sialophosphoprotein	DSPP		GeneCard	DisGenet
1283	Destrin, Actin Depolymerizing Factor	DSTN		GeneCard	
1284	DTW Domain Containing 1	DTWD1		GeneCard	

128<br>5	Dual Oxidase 1	DUOX1		GeneCard	
128<br>6	Dual Oxidase 2	DUOX2		GeneCard	
128<br>7	Dual Specificity Phosphatase 1	DUSP1		GeneCard	DisGenet
128<br>8	Dual Specificity Phosphatase 16	DUSP16		GeneCard	
128<br>9	Dual Specificity Phosphatase 19	DUSP19		GeneCard	
129<br>0	Dual Specificity Phosphatase 2	DUSP2		GeneCard	
129<br>1	Dual Specificity Phosphatase 5	DUSP5			DisGenet
129<br>2	Dual Specificity Phosphatase 6	DUSP6		GeneCard	
129<br>3	Deoxyuridine Triphosphatase	DUT		GeneCard	
129<br>4	Dymeclin	DYM		GeneCard	DisGenet
129<br>5	Dynein Cytoplasmic 2 Light Intermediate Chain 1	DYNC2LI1		GeneCard	
129<br>6	Dynein Light Chain LC8-Type 1	DYNLL1			DisGenet
129<br>7	Dual Specificity Tyrosine Phosphorylation Regulated Kinase 1A	DYRK1A		GeneCard	
129<br>8	E2F Transcription Factor 1	E2F1		GeneCard	
129<br>9	E2F Transcription Factor 3	E2F3		GeneCard	
130<br>0	E2F Transcription Factor 8	E2F8		GeneCard	
130<br>1	Glutamyl-tRNA Synthetase 2, Mitochondrial	EARS2			DisGenet
130<br>2	EBF Transcription Factor 1	EBF1		GeneCard	DisGenet
130<br>3	Epstein-Barr Virus Induced 3	EBI3		GeneCard	DisGenet
130<br>4	Endogenous Bornavirus Like Nucleoprotein 1	EBLN1		GeneCard	

130<br>5	EBNA1 binding protein 2	EBNA1BP2			DisGenet
130<br>6	EBP Like	EBPL		GeneCard	
130<br>7	Endothelin Converting Enzyme 1	ECE1		GeneCard	DisGenet
130<br>8	Endothelin Converting Enzyme 2	ECE2		GeneCard	DisGenet
130<br>9	Endothelial Cell Surface Expressed Chemotaxis and Apoptosis Regulator	ECSCR			DisGenet
131<br>0	Ectodysplasin A	EDA			DisGenet
131<br>1	Ectodysplasin A Receptor	EDAR		GeneCard	
131<br>2	EDAR Associated Death Domain	EDARADD		GeneCard	
131<br>3	ER Degradation Enhancing Alpha-Mannosidase Like Protein 2	EDEM2		GeneCard	
131<br>4	Endothelin 1	EDN1		GeneCard	DisGenet
131<br>5	Endothelin 2	EDN2		GeneCard	
131<br>6	Endothelin 3	EDN3		GeneCard	DisGenet
131<br>7	Endothelin Receptor Type A	EDNRA		GeneCard	DisGenet
131<br>8	Endothelin Receptor Type B	EDNRB		GeneCard	DisGenet
131<br>9	Embryonic Ectoderm Development	EED		GeneCard	
132<br>0	Eukaryotic Translation Elongation Factor 1 Alpha 1	EEF1A1		GeneCard	
132<br>1	Eukaryotic Translation Elongation Factor 1 Beta 2 Pseudogene 2	EEF1B2P2			DisGenet
132<br>2	Eukaryotic Translation Elongation Factor 1 Epsilon 1	EEF1E1		GeneCard	
132<br>3	Eukaryotic Elongation Factor 2 Kinase	EEF2K		GeneCard	
132<br>4	EGF Containing Fibulin Extracellular Matrix Protein 1	EFEMP1		GeneCard	

132<br>5	EGF Containing Fibulin Extracellular Matrix Protein 2	EFEMP2		GeneCard	
132<br>6	Elongation Factor Like GTPase 1	EFL1		GeneCard	
132<br>7	Ephrin A2	EFNA2		GeneCard	
132<br>8	Ephrin A5	EFNA5		GeneCard	
132<br>9	Ephrin B1	EFNB1		GeneCard	DisGenet
133<br>0	Ephrin B2	EFNB2		GeneCard	
133<br>1	Epidermal Growth Factor	EGF		GeneCard	DisGenet
133<br>2	EGF Like Domain Multiple 7	EGFL7		GeneCard	DisGenet
133<br>3	Epidermal Growth Factor Receptor	EGFR		GeneCard	DisGenet
133<br>4	APOB 3' Scaffold/Matrix Attachment Region	EGID-106632268		GeneCard	
133<br>5	Egl-9 Family Hypoxia Inducible Factor 3	EGLN3			DisGenet
133<br>6	Early Growth Response 1	EGR1		GeneCard	DisGenet
133<br>7	Early Growth Response 3	EGR3		GeneCard	
133<br>8	Euchromatic Histone Lysine Methyltransferase 1	EHMT1			DisGenet
133<br>9	Euchromatic Histone Lysine Methyltransferase 2	EHMT2		GeneCard	
134<br>0	EP300 Interacting Inhibitor of Differentiation 1	EID1			DisGenet
134<br>1	Eukaryotic Translation Initiation Factor 2 Alpha Kinase 2	EIF2AK2		GeneCard	
134<br>2	Eukaryotic Translation Initiation Factor 2 Alpha Kinase 3	EIF2AK3		GeneCard	
134<br>3	Eukaryotic Translation Initiation Factor 2B Subunit Beta	EIF2B2		GeneCard	
134<br>4	Eukaryotic Translation Initiation Factor 2B Subunit Delta	EIF2B4		GeneCard	

1345	Eukaryotic Translation Initiation Factor 2 Subunit Alpha	EIF2S1		GeneCard	
1346	Eukaryotic Translation Initiation Factor 3 Subunit F Pseudogene 3	EIF3FP3		GeneCard	
1347	Eukaryotic Translation Initiation Factor 4B	EIF4B		GeneCard	
1348	Eukaryotic Translation Initiation Factor 4E	EIF4E		GeneCard	
1349	Eukaryotic Translation Initiation Factor 4E Binding Protein 1	EIF4EBP1		GeneCard	
1350	Eukaryotic Translation Initiation Factor 4E Nuclear Import Factor 1	EIF4ENIF1		GeneCard	
1351	Eukaryotic Translation Initiation Factor 4 Gamma 2	EIF4G2		GeneCard	
1352	Eukaryotic Translation Initiation Factor 6	EIF6		GeneCard	
1353	Elastase, Neutrophil Expressed	ELANE		GeneCard	DisGenet
1354	ELAV Like RNA Binding Protein 1	ELAVL1		GeneCard	DisGenet
1355	ELAV like RNA Binding Protein 2	ELAVL2			DisGenet
1356	ETS Transcription Factor ELK1	ELK1		GeneCard	DisGenet
1357	Engulfment and Cell Motility 1	ELMO1			DisGenet
1358	Elastin	ELN	OMIM	GeneCard	DisGenet
1359	Elongin A	ELOA		GeneCard	
1360	ELOVL Fatty Acid Elongase 2	ELOVL2		GeneCard	
1361	ELOVL Fatty Acid Elongase 5	ELOVL5		GeneCard	
1362	ELOVL Fatty Acid Elongase 6	ELOVL6			DisGenet
1363	ELOVL Fatty Acid Elongase 7	ELOVL7		GeneCard	
1364	ER Membrane Protein Complex Subunit 10	EMC10		GeneCard	

1365	Emerin	EMD		GeneCard	
1366	EMAP Like 1	EML1		GeneCard	
1367	Epithelial Membrane Protein 1	EMP1		GeneCard	DisGenet
1368	Endonuclease G	ENDOG		GeneCard	
1369	Endoglin	ENG	OMIM	GeneCard	DisGenet
1370	Energy Homeostasis Associated	ENHO		GeneCard	DisGenet
1371	Enolase 1	ENO1		GeneCard	
1372	Enolase 2	ENO2		GeneCard	
1373	Enolase 3	ENO3		GeneCard	
1374	Enolase Superfamily Member 1	ENOSF1		GeneCard	
1375	Ecto-NOX Disulfide-Thiol Exchanger 1	ENOX1		GeneCard	
1376	Ectonucleotide Pyrophosphatase/Phosphodiesterase 1	ENPP1		GeneCard	DisGenet
1377	Ectonucleotide Pyrophosphatase/Phosphodiesterase 2	ENPP2		GeneCard	
1378	Ectonucleotide Pyrophosphatase/Phosphodiesterase 7	ENPP7		GeneCard	DisGenet
1379	Novel Transcript, Antisense To STEAP1B	ENSG00000232949		GeneCard	
1380	Novel Transcript, Sense Overlapping CCDC71L	ENSG00000243797		GeneCard	
1381	Novel Transcript	ENSG00000258672		GeneCard	
1382	Hsa-Mir-423	ENSG00000266919		GeneCard	
1383	Novel Transcript	ENSG00000267052		GeneCard	
1384	Novel Transcript	ENSG00000269918		GeneCard	

1385	La Ribonucleoprotein Domain Family, Member 1B (LARP1B) Pseudogene	ENSG00000271680		GeneCard	
1386	TEC	ENSG00000280087		GeneCard	
1387	Ectonucleoside Triphosphate Diphosphohydrolase 1	ENTPD1		GeneCard	DisGenet
1388	Ectonucleoside Triphosphate Diphosphohydrolase 7	ENTPD7		GeneCard	
1389	E1A Binding Protein P300	EP300		GeneCard	
1390	Endothelial PAS Domain Protein 1	EPAS1		GeneCard	DisGenet
1391	Erythrocyte Membrane Protein Band 4.1 Like 3	EPB41L3		GeneCard	
1392	EPB41L4A Antisense RNA 1	EPB41L4A-AS1		GeneCard	
1393	EPH Receptor A1	EPHA1		GeneCard	DisGenet
1394	EPH Receptor A2	EPHA2		GeneCard	DisGenet
1395	EPH Receptor A3	EPHA3		GeneCard	
1396	EPH Receptor A6	EPHA6		GeneCard	
1397	EPH Receptor B2	EPHB2		GeneCard	DisGenet
1398	EPH Receptor B3	EPHB3		GeneCard	
1399	Epoxide Hydrolase 1	EPHX1		GeneCard	
1400	Epoxide hydrolase 2, cytoplasmic	EPHX2	OMIM	GeneCard	DisGenet
1401	EPM2A Glucan Phosphatase, Laforin	EPM2A		GeneCard	
1402	Epsin 1	EPN1		GeneCard	
1403	Erythropoietin	EPO		GeneCard	DisGenet
1404	Erythropoietin Receptor	EPOR		GeneCard	

140<br>5	Epididymal Peptidase Inhibitor	EPPIN		GeneCard	
140<br>6	Glutamyl-Prolyl-TRNA Synthetase 1	EPRS1		GeneCard	DisGenet
140<br>7	Epithelial Stromal Interaction 1	EPSTI1		GeneCard	
140<br>8	Endoplasmic Reticulum Aminopeptidase 1	ERAP1		GeneCard	
140<br>9	Erb-B2 Receptor Tyrosine Kinase 2	ERBB2		GeneCard	
141<br>0	Erb-B2 Receptor Tyrosine Kinase 4	ERBB4		GeneCard	
141<br>1	Erbb2 Interacting Protein	ERBIN		GeneCard	
141<br>2	ERCC Excision Repair 1, Endonuclease Non-Catalytic Subunit	ERCC1		GeneCard	
141<br>3	ERCC Excision Repair 2, TFIIH Core Complex Helicase Subunit	ERCC2		GeneCard	
141<br>4	ERCC Excision Repair 3, TFIIH Core Complex Helicase Subunit	ERCC3		GeneCard	
141<br>5	ERCC Excision Repair 4, Endonuclease Catalytic Subunit	ERCC4		GeneCard	
141<br>6	ERCC Excision Repair 5, Endonuclease	ERCC5		GeneCard	
141<br>7	ERCC Excision Repair 6, Chromatin Remodeling Factor	ERCC6		GeneCard	
141<br>8	ERCC Excision Repair 8, CSA Ubiquitin Ligase Complex Subunit	ERCC8	OMIM	GeneCard	
141<br>9	Epiregulin	EREG	OMIM	GeneCard	DisGenet
142<br>0	Erythroferrone	ERFE		GeneCard	
142<br>1	ETS Transcription Factor ERG	ERG		GeneCard	DisGenet
142<br>2	Endoplasmic Reticulum To Nucleus Signaling 1	ERN1		GeneCard	DisGenet
142<br>3	Endothelial Cell Adhesion Molecule	ESAM		GeneCard	DisGenet
142<br>4	Endothelial Cell Specific Molecule 1	ESM1		GeneCard	

1425	Estrogen receptor 1	ESR1	OMIM	GeneCard	DisGenet
1426	Estrogen Receptor 2	ESR2		GeneCard	DisGenet
1427	Estrogen Related Receptor Alpha	ESRRA		GeneCard	
1428	Estrogen Related Receptor Beta	ESRRB		GeneCard	
1429	Ethanolamine-Phosphate Phospho-Lyase	ETNPPL		GeneCard	
1430	ETS Proto-Oncogene 1, Transcription Factor	ETS1		GeneCard	DisGenet
1431	ETS Proto-Oncogene 2, Transcription Factor	ETS2		GeneCard	
1432	ETS Variant Transcription Factor 1	ETV1		GeneCard	
1433	ETS Variant Transcription Factor 6	ETV6		GeneCard	
1434	Eva-1 Homolog A, Regulator Of Programmed Cell Death	EVA1A		GeneCard	
1435	Enah/Vasp-Like	EVL		GeneCard	
1436	Envoplakin	EVPL			DisGenet
1437	Even-Skipped Homeobox 1	EVX1		GeneCard	
1438	Even-Skipped Homeobox 2	EVX2		GeneCard	
1439	Exonuclease 1	EXO1		GeneCard	
1440	Exosome Component 4	EXOSC4		GeneCard	
1441	EYA Transcriptional Coactivator and Phosphatase 1	EYA1		GeneCard	
1442	EYA Transcriptional Coactivator and Phosphatase 4	EYA4		GeneCard	
1443	Eyes Shut Homolog	EYS		GeneCard	
1444	Enhancer Of Zeste 2 Polycomb Repressive Complex 2 Subunit	EZH2		GeneCard	DisGenet

144 5	Coagulation Factor X	F10		GeneCard	DisGenet
144 6	Coagulation Factor XI	F11		GeneCard	DisGenet
144 7	F11 Antisense RNA 1	F11-AS1		GeneCard	
144 8	F11 Receptor	F11R		GeneCard	DisGenet
144 9	Coagulation Factor XII	F12		GeneCard	DisGenet
145 0	Coagulation Factor XIII A Chain	F13A1	OMIM	GeneCard	DisGenet
145 1	Coagulation Factor XIII B Chain	F13B		GeneCard	
145 2	Coagulation Factor II, Thrombin	F2	OMIM	GeneCard	DisGenet
145 3	Coagulation Factor II Thrombin Receptor	F2R		GeneCard	DisGenet
145 4	F2R Like Trypsin Receptor 1	F2RL1		GeneCard	DisGenet
145 5	Coagulation Factor II Thrombin Receptor Like 2	F2RL2		GeneCard	
145 6	F2R Like Thrombin Or Trypsin Receptor 3	F2RL3		GeneCard	
145 7	Coagulation Factor III, Tissue Factor	F3		GeneCard	DisGenet
145 8	Coagulation Factor V	F5	OMIM	GeneCard	DisGenet
145 9	Coagulation Factor VII	F7	OMIM	GeneCard	DisGenet
146 0	Coagulation Factor VIII	F8		GeneCard	
146 1	Coagulation Factor IX	F9		GeneCard	
146 2	Fatty Acid 2-Hydroxylase	FA2H		GeneCard	DisGenet
146 3	Fatty Acid Binding Protein 12	FABP12		GeneCard	
146 4	Fatty Acid Binding Protein 2	FABP2		GeneCard	DisGenet

1465	Fatty Acid Binding Protein 3	FABP3		GeneCard	DisGenet
1466	Fatty Acid Binding Protein 4	FABP4	OMIM	GeneCard	DisGenet
1467	Fatty Acid Binding Protein 5	FABP5		GeneCard	DisGenet
1468	Fatty Acid Binding Protein 6	FABP6		GeneCard	
1469	Fas Associated Via Death Domain	FADD		GeneCard	
1470	Fatty Acid Desaturase 1	FADS1		GeneCard	DisGenet
1471	Fatty Acid Desaturase 2	FADS2		GeneCard	DisGenet
1472	Fatty Acid Desaturase 3	FADS3		GeneCard	
1473	Fumarylacetoacetate Hydrolase	FAH		GeneCard	
1474	Family With Sequence Similarity 126 Member A	FAM126A		GeneCard	
1475	Family With Sequence Similarity 13 Member A	FAM13A		GeneCard	
1476	Family With Sequence Similarity 186 Member A	FAM186A		GeneCard	
1477	FAM20C Golgi Associated Secretory Pathway Kinase	FAM20C		GeneCard	DisGenet
1478	Family With Sequence Similarity 223 Member A	FAM223A		GeneCard	
1479	Family With Sequence Similarity 89 Member A	FAM89A		GeneCard	
1480	FA Complementation Group A	FANCA		GeneCard	
1481	Fibroblast Activation Protein Alpha	FAP		GeneCard	DisGenet
1482	Phenylalanyl-TRNA Synthetase 2, Mitochondrial	FARS2		GeneCard	
1483	Fas Cell Surface Death Receptor	FAS		GeneCard	DisGenet
1484	Fas Ligand	FASLG		GeneCard	DisGenet

148 5	Fatty Acid Synthase	FASN		GeneCard	
148 6	FAT Atypical Cadherin 1	FAT1			DisGenet
148 7	FAT Atypical Cadherin 4	FAT4		GeneCard	
148 8	Filamin Binding LIM Protein 1	FBLIM1		GeneCard	
148 9	Fibulin 1	FBLN1		GeneCard	
149 0	Fibulin 2	FBLN2			DisGenet
149 1	Fibulin 5	FBLN5		GeneCard	
149 2	Fibrillin 1	FBN1		GeneCard	
149 3	Fibrillin 2	FBN2		GeneCard	
149 4	Fibrosin	FBRS			DisGenet
149 5	F-Box Protein 15	FBXO15		GeneCard	
149 6	F-Box Protein 3	FBXO3		GeneCard	DisGenet
149 7	F-Box Protein 32	FBXO32		GeneCard	
149 8	F-Box Protein 33	FBXO33		GeneCard	
149 9	F-Box Protein 38	FBXO38		GeneCard	
150 0	F-box Protein 8	FBXO8			DisGenet
150 1	F-Box and WD Repeat Domain Containing 2	FBXW2		GeneCard	
150 2	F-Box and WD Repeat Domain Containing 7	FBXW7		GeneCard	
150 3	Fc Alpha and Mu Receptor	FCAMR		GeneCard	
150 4	Fc Gamma Receptor Ia	FCGR1A		GeneCard	

150<br>5	Fc Gamma Receptor IIa	FCGR2A		GeneCard	DisGenet
150<br>6	Fc Gamma Receptor IIb	FCGR2B		GeneCard	DisGenet
150<br>7	Fc Gamma Receptor IIc (Gene/Pseudogene)	FCGR2C		GeneCard	
150<br>8	Fc Gamma Receptor IIIa	FCGR3A		GeneCard	DisGenet
150<br>9	Fc Gamma Receptor IIIb	FCGR3B		GeneCard	DisGenet
151<br>0	Fc Gamma Receptor and Transporter	FCGRT		GeneCard	
151<br>1	Fc Mu Receptor	FCMR		GeneCard	
151<br>2	Ficolin 2	FCN2		GeneCard	
151<br>3	Ficolin 3	FCN3		GeneCard	
151<br>4	Fc Receptor Like 3	FCRL3		GeneCard	
151<br>5	Fc Receptor Like 6	FCRL6		GeneCard	DisGenet
151<br>6	Farnesyl-Diphosphate Farnesyltransferase 1	FDFT1		GeneCard	
151<br>7	Farnesyl Diphosphate Synthase	FDPS			DisGenet
151<br>8	Ferredoxin 1	FDX1		GeneCard	
151<br>9	Fem-1 Homolog A	FEM1A		GeneCard	
152<br>0	Fem-1 Homolog B	FEM1B		GeneCard	
152<br>1	Flap Structure-Specific Endonuclease 1	FEN1		GeneCard	
152<br>2	FERM Domain Containing Kindlin 3	FERMT3		GeneCard	
152<br>3	Fetuin B	FETUB		GeneCard	DisGenet
152<br>4	Free Fatty Acid Receptor 1	FFAR1		GeneCard	DisGenet

1525	Free Fatty Acid Receptor 4	FFAR4		GeneCard	DisGenet
1526	Fibrinogen Alpha Chain	FGA	OMIM	GeneCard	DisGenet
1527	Fibrinogen Beta Chain	FGB	OMIM	GeneCard	DisGenet
1528	Fibroblast Growth Factor 1	FGF1		GeneCard	DisGenet
1529	Fibroblast Growth Factor 10	FGF10		GeneCard	
1530	Fibroblast Growth Factor 12	FGF12		GeneCard	DisGenet
1531	Fibroblast Growth Factor 13	FGF13			DisGenet
1532	Fibroblast Growth Factor 14	FGF14		GeneCard	
1533	Fibroblast Growth Factor 19	FGF19		GeneCard	DisGenet
1534	Fibroblast Growth Factor 2	FGF2		GeneCard	DisGenet
1535	Fibroblast Growth Factor 21	FGF21		GeneCard	DisGenet
1536	Fibroblast Growth Factor 23	FGF23		GeneCard	DisGenet
1537	Fibroblast Growth Factor 3	FGF3		GeneCard	
1538	Fibroblast Growth Factor 4	FGF4		GeneCard	
1539	Fibroblast Growth Factor 6	FGF6		GeneCard	
1540	Fibroblast Growth Factor 7	FGF7		GeneCard	
1541	Fibroblast Growth Factor 9	FGF9		GeneCard	
1542	Fibroblast Growth Factor Receptor 1	FGFR1		GeneCard	DisGenet
1543	Fibroblast Growth Factor Receptor 2	FGFR2		GeneCard	
1544	Fibroblast Growth Factor Receptor 3	FGFR3		GeneCard	

1545	Fibroblast Growth Factor Receptor 4	FGFR4		GeneCard	DisGenet
1546	Fibrinogen Gamma Chain	FGG		GeneCard	DisGenet
1547	Fumarate Hydratase	FH		GeneCard	
1548	Four And A Half LIM Domains 1	FHL1		GeneCard	
1549	Four And A Half LIM Domains 2	FHL2		GeneCard	DisGenet
1550	Four And A Half LIM Domains 5	FHL5		GeneCard	
1551	Formin Homology 2 Domain Containing 3	FHOD3		GeneCard	
1552	Folliculogenesis Specific BHLH Transcription Factor	FIGLA		GeneCard	
1553	Factor Interacting with PAPOLA and CPSF1	FIP1L1		GeneCard	
1554	Fasting Glucose and Specific Insulin Levels	FIQTL1			DisGenet
1555	FKBP Prolyl Isomerase 1A	FKBP1A		GeneCard	
1556	FKBP Prolyl Isomerase 1B	FKBP1B		GeneCard	
1557	Fukutin Related Protein	FKRP		GeneCard	
1558	Fukutin	FKTN		GeneCard	
1559	Filamin A	FLNA		GeneCard	DisGenet
1560	Filamin B	FLNB		GeneCard	
1561	Filamin C	FLNC		GeneCard	
1562	FLNC Antisense RNA 1	FLNC-AS1		GeneCard	
1563	Fibronectin Leucine Rich Transmembrane Protein 2	FLRT2		GeneCard	
1564	Fms Related Receptor Tyrosine Kinase 1	FLT1		GeneCard	DisGenet

1565	Fms Related Receptor Tyrosine Kinase 4	FLT4		GeneCard	DisGenet
1566	FLYWCH-Type Zinc Finger 1	FLYWCH1		GeneCard	
1567	Formin 1	FMN1		GeneCard	
1568	Formin 2	FMN2		GeneCard	
1569	Formin Like 2	FMNL2		GeneCard	
1570	Flavin Containing Dimethylaniline Monoxygenase 3	FMO3		GeneCard	DisGenet
1571	Fibromodulin	FMOD		GeneCard	DisGenet
1572	Fragile X Messenger Ribonucleoprotein 1	FMR1		GeneCard	
1573	Fibronectin 1	FN1		GeneCard	DisGenet
1574	Fibronectin Type III Domain Containing 4	FNDC4		GeneCard	
1575	Fibronectin Type III Domain Containing 5	FNDC5		GeneCard	DisGenet
1576	Folate Hydrolase 1	FOLH1		GeneCard	
1577	Folate Receptor Beta	FOLR2		GeneCard	DisGenet
1578	FOS Proto-Oncogene, AP-1 Transcription Factor Subunit	FOS		GeneCard	DisGenet
1579	FOSB Proto-Oncogene, AP-1 Transcription Factor Subunit	FOSB		GeneCard	DisGenet
1580	FOS Like 1, AP-1 Transcription Factor Subunit	FOSL1		GeneCard	
1581	FOS Like 2, AP-1 Transcription Factor Subunit	FOSL2		GeneCard	
1582	Forkhead Box A1	FOXA1		GeneCard	
1583	Forkhead Box A2	FOXA2		GeneCard	
1584	Forkhead Box A3	FOXA3		GeneCard	DisGenet

158<br>5	Forkhead Box B1	FOXB1		GeneCard	
158<br>6	Forkhead Box C1	FOXC1		GeneCard	
158<br>7	Forkhead Box C2	FOXC2		GeneCard	
158<br>8	Forkhead Box D4	FOXD4		GeneCard	
158<br>9	Forkhead Box E1	FOXE1		GeneCard	
159<br>0	Forkhead Box E3	FOXE3		GeneCard	
159<br>1	Forkhead Box J1	FOXJ1		GeneCard	
159<br>2	Forkhead Box L2	FOXL2		GeneCard	
159<br>3	Forkhead Box M1	FOXM1		GeneCard	DisGenet
159<br>4	Forkhead Box O1	FOXO1		GeneCard	DisGenet
159<br>5	Forkhead Box O3	FOXO3		GeneCard	DisGenet
159<br>6	Forkhead Box O4	FOXO4		GeneCard	DisGenet
159<br>7	Forkhead Box P1	FOXP1		GeneCard	DisGenet
159<br>8	Forkhead Box P2	FOXP2		GeneCard	
159<br>9	Forkhead Box P3	FOXP3		GeneCard	DisGenet
160<br>0	Folylpolyglutamate Synthase	FPGS		GeneCard	
160<br>1	Formyl Peptide Receptor 1	FPR1		GeneCard	
160<br>2	Formyl Peptide Receptor 2	FPR2		GeneCard	DisGenet
160<br>3	Fibroblast Growth Factor Receptor Substrate 2	FRS2		GeneCard	DisGenet
160<br>4	Fibronectin Type III and SPRY Domain Containing 2	FSD2		GeneCard	

1605	Follicle Stimulating Hormone Subunit Beta	FSHB		GeneCard	
1606	Facioscapulohumeral Muscular Dystrophy 1A	FSHMD1A			DisGenet
1607	Follicle Stimulating Hormone Receptor	FSHR		GeneCard	
1608	Follistatin	FST		GeneCard	DisGenet
1609	Follistatin Like 1	FSTL1		GeneCard	
1610	Follistatin Like 3	FSTL3			DisGenet
1611	Ferritin Heavy Chain 1	FTH1		GeneCard	DisGenet
1612	FTO Alpha-Ketoglutarate Dependent Dioxygenase	FTO		GeneCard	DisGenet
1613	Furin, Paired Basic Amino Acid Cleaving Enzyme	FURIN		GeneCard	DisGenet
1614	FUS RNA Binding Protein	FUS		GeneCard	
1615	Fucosyltransferase 3 (Lewis Blood Group)	FUT3		GeneCard	DisGenet
1616	Fucosyltransferase 4	FUT4		GeneCard	
1617	Fucosyltransferase 7	FUT7		GeneCard	DisGenet
1618	Fucosyltransferase 8	FUT8			DisGenet
1619	FYN Proto-Oncogene, Src Family Tyrosine Kinase	FYN		GeneCard	DisGenet
1620	Frizzled Class Receptor 5	FZD5			DisGenet
1621	Frizzled Class Receptor 8	FZD8		GeneCard	
1622	G0/G1 Switch 2	G0S2		GeneCard	
1623	G3BP Stress Granule Assembly Factor 1	G3BP1		GeneCard	
1624	Glucose-6-Phosphatase Catalytic Subunit 1	G6PC1		GeneCard	

1625	Glucose-6-Phosphatase Catalytic Subunit 2	G6PC2		GeneCard	
1626	Glucose-6-Phosphate Dehydrogenase	G6PD		GeneCard	
1627	Alpha Glucosidase	GAA		GeneCard	
1628	GRB2 Associated Binding Protein 1	GAB1		GeneCard	
1629	GA Binding Protein Transcription Factor Subunit Alpha	GABPA			DisGenet
1630	Gamma-Aminobutyric Acid Type A Receptor Subunit Gamma1	GABRG1		GeneCard	
1631	Glutamate Decarboxylase 2	GAD2		GeneCard	
1632	Growth Arrest and DNA Damage Inducible Alpha	GADD45A		GeneCard	DisGenet
1633	Growth Arrest and DNA Damage Inducible Gamma	GADD45G		GeneCard	
1634	Galanin And GMAP Prepropeptide	GAL		GeneCard	
1635	Galactose-3-O-Sulfotransferase 1	GAL3ST1			DisGenet
1636	Galactokinase 1	GALK1		GeneCard	
1637	Galactosamine (N-Acetyl)-6-Sulfatase	GALNS		GeneCard	
1638	Polypeptide N-Acetylgalactosaminyltransferase 10	GALNT10		GeneCard	
1639	Polypeptide N-Acetylgalactosaminyltransferase 2	GALNT2		GeneCard	
1640	Polypeptide N-Acetylgalactosaminyltransferase 3	GALNT3		GeneCard	
1641	Galactose-1-Phosphate Uridylyltransferase	GALT		GeneCard	
1642	Glucosidase II Alpha Subunit	GANAB		GeneCard	
1643	Glucosidase Alpha, Neutral C	GANC		GeneCard	
1644	Glyceraldehyde-3-Phosphate Dehydrogenase	GAPDH		GeneCard	

164<br>5	Glyceraldehyde 3 Phosphate Dehydrogenase Pseudogene 28	GAPDHP28		GeneCard	
164<br>6	Growth Arrest Specific 5	GAS5		GeneCard	DisGenet
164<br>7	GAS5 Antisense RNA 1	GAS5-AS1		GeneCard	
164<br>8	Growth Arrest Specific 6	GAS6		GeneCard	DisGenet
164<br>9	Gastrin	GAST		GeneCard	DisGenet
165<br>0	GATA Binding Protein 2	GATA2		GeneCard	DisGenet
165<br>1	GATA Binding Protein 3	GATA3		GeneCard	
165<br>2	GATA Binding Protein 4	GATA4		GeneCard	DisGenet
165<br>3	GATA Binding Protein 5	GATA5		GeneCard	
165<br>4	GATA Binding Protein 6	GATA6		GeneCard	
165<br>5	GATA Zinc Finger Domain Containing 1	GATAD1		GeneCard	
165<br>6	Glutamine Amidotransferase Like Class 1 Domain Containing 3A	GATD3A			DisGenet
165<br>7	Glutamine Amidotransferase Like Class 1 Domain Containing 3B	GATD3B			DisGenet
165<br>8	Glycine Amidinotransferase	GATM		GeneCard	
165<br>9	Glucosylceramidase Beta	GBA		GeneCard	
166<br>0	Guanylate Binding Protein 1	GBP1		GeneCard	
166<br>1	GC Vitamin D Binding Protein	GC		GeneCard	DisGenet
166<br>2	Grancalcin	GCA			DisGenet
166<br>3	Glucagon	GCG	OMIM	GeneCard	DisGenet
166<br>4	Glucagon Receptor	GCGR			DisGenet

166<br>5	GTP Cyclohydrolase 1	GCH1		GeneCard	DisGenet
166<br>6	Glucokinase	GCK		GeneCard	DisGenet
166<br>7	Glucokinase Regulator	GCKR		GeneCard	DisGenet
166<br>8	Glutamate-Cysteine Ligase Catalytic Subunit	GCLC	OMIM	GeneCard	
166<br>9	Glutamate-Cysteine Ligase Modifier Subunit	GCLM	OMIM	GeneCard	DisGenet
167<br>0	GCOM1, MYZAP-POLR2M Combined Locus	GCOM1		GeneCard	
167<br>1	Glycerophosphodiester Phosphodiesterase 1	GDE1		GeneCard	
167<br>2	Growth Differentiation Factor 10	GDF10		GeneCard	
167<br>3	Growth Differentiation Factor 11	GDF11		GeneCard	DisGenet
167<br>4	Growth Differentiation Factor 15	GDF15		GeneCard	DisGenet
167<br>5	Growth Differentiation Factor 2	GDF2		GeneCard	DisGenet
167<br>6	Growth Differentiation Factor 9	GDF9		GeneCard	
167<br>7	Glial Cell Derived Neurotrophic Factor	GDNF		GeneCard	
167<br>8	Glial Fibrillary Acidic Protein	GFAP		GeneCard	
167<br>9	Growth Factor, Augmenter of Liver Regeneration	GFER			DisGenet
168<br>0	G elongation Factor Mitochondrial 1	GFM1			DisGenet
168<br>1	Glutamine--Fructose-6-Phosphate Transaminase 1	GFPT1		GeneCard	
168<br>2	Glutamine-Fructose-6-Phosphate Transaminase 2	GFPT2		GeneCard	
168<br>3	GDNF Family Receptor Alpha Like	GFRAL		GeneCard	
168<br>4	Gamma-Glutamyl Carboxylase	GGCX	OMIM	GeneCard	DisGenet

1685	Geranylgeranyl Diphosphate Synthase 1	GGPS1		GeneCard	
1686	Gamma-Glutamyltransferase 1	GGT1		GeneCard	
1687	Gamma-Glutamyltransferase 2, Pseudogene	GGT2P		GeneCard	
1688	Gamma-Glutamyltransferase Light Chain 1	GGTLC1			DisGenet
1689	Growth Hormone 1	GH1		GeneCard	DisGenet
1690	Growth Hormone Receptor	GHR	OMIM	GeneCard	
1691	Growth Hormone Releasing Hormone	GHRH		GeneCard	
1692	Growth Hormone Releasing Hormone Receptor	GHRHR	OMIM	GeneCard	DisGenet
1693	Ghrelin And Obestatin Prepropeptide	GHRL		GeneCard	DisGenet
1694	Growth Hormone Secretagogue Receptor	GHSR		GeneCard	DisGenet
1695	GTPase, IMAP Family Member 5	GIMAP5		GeneCard	
1696	Gastric Inhibitory Polypeptide	GIP		GeneCard	DisGenet
1697	GIPC PDZ Domain Containing Family Member 1	GIPC1		GeneCard	
1698	Gap Junction Protein Alpha 1	GJA1		GeneCard	DisGenet
1699	Gap Junction Protein Alpha 4	GJA4	OMIM	GeneCard	DisGenet
1700	Gap Junction Protein Alpha 5	GJA5		GeneCard	DisGenet
1701	Gap Junction Protein Beta 1	GJB1		GeneCard	
1702	GJD2 Divergent Transcript	GJD2-DT		GeneCard	
1703	Galactosidase Alpha	GLA		GeneCard	
1704	Galactosidase Beta 1	GLB1	OMIM	GeneCard	

1705	Glycine Decarboxylase	GLDC		GeneCard	
1706	GLI Family Zinc Finger 2	GLI2			DisGenet
1707	Glomulin, FKBP Associated Protein	GLMN		GeneCard	
1708	Glyoxalase I	GLO1		GeneCard	DisGenet
1709	Glucagon Like Peptide 1 Receptor	GLP1R		GeneCard	DisGenet
1710	Glycine Receptor Alpha 3	GLRA3		GeneCard	
1711	Glutaredoxin	GLRX		GeneCard	DisGenet
1712	Glutaredoxin Pseudogene 2	GLRXP2		GeneCard	
1713	Glutaminase 2	GLS2		GeneCard	
1714	Glycolipid Transfer Protein Domain Containing 2	GLTPD2		GeneCard	
1715	G Protein Subunit Alpha 11	GNA11		GeneCard	
1716	G Protein Subunit Alpha 12	GNA12		GeneCard	
1717	G Protein Subunit Alpha 15	GNA15		GeneCard	
1718	G Protein Subunit Alpha I1	GNAI1		GeneCard	
1719	G Protein Subunit Alpha I2	GNAI2		GeneCard	
1720	G Protein Subunit Alpha Q	GNAQ		GeneCard	
1721	GNAS Complex Locus	GNAS		GeneCard	
1722	G Protein Subunit Beta 3	GNB3		GeneCard	DisGenet
1723	G Protein Subunit Gamma Transducin 1	GNGT1		GeneCard	
1724	Glycine N-Methyltransferase	GNMT		GeneCard	

1725	Glucosamine-6-Phosphate Deaminase 2	GNPDA2		GeneCard	
1726	Gonadotropin Releasing Hormone 1	GNRH1		GeneCard	
1727	Golgin A6 Family Member A	GOLGA6A			DisGenet
1728	Golgin B1	GOLGB1		GeneCard	DisGenet
1729	Golgin, RAB6 Interacting	GORAB		GeneCard	
1730	Golgi Reassembly Stacking Protein 1	GORASP1		GeneCard	DisGenet
1731	Golgi SNAP Receptor Complex Member 2	GOSR2		GeneCard	DisGenet
1732	Glutamic-Oxaloacetic Transaminase 2	GOT2		GeneCard	DisGenet
1733	Glycoprotein Ib Platelet Subunit Alpha	GP1BA		GeneCard	DisGenet
1734	Glycoprotein Ib Platelet Subunit Beta	GP1BB		GeneCard	
1735	Glycoprotein V Platelet	GP5		GeneCard	
1736	Glycoprotein VI Platelet	GP6		GeneCard	DisGenet
1737	Glycoprotein IX Platelet	GP9		GeneCard	
1738	Glycerol-3-Phosphate Acyltransferase, Mitochondrial	GPAM		GeneCard	
1739	G Protein-Coupled Bile Acid Receptor 1	GPBAR1		GeneCard	DisGenet
1740	GC-Rich Promoter Binding Protein 1	GPBP1	OMIM	GeneCard	
1741	Glypican 1	GPC1		GeneCard	
1742	Glypican 5	GPC5		GeneCard	
1743	Glycerol-3-Phosphate Dehydrogenase 2	GPD2		GeneCard	
1744	G Protein-Coupled Estrogen Receptor 1	GPER1		GeneCard	DisGenet

1745	Glucose-6-Phosphate Isomerase	GPI			DisGenet
1746	Glycosylphosphatidylinositol Anchored High Density Lipoprotein Binding Protein 1	GPIHBP1		GeneCard	DisGenet
1747	Glycosylphosphatidylinositol Specific Phospholipase D1	GPLD1		GeneCard	
1748	Glycoprotein M6A	GPM6A		GeneCard	
1749	G Protein-Coupled Receptor 119	GPR119		GeneCard	DisGenet
1750	G Protein-Coupled Receptor 132	GPR132		GeneCard	
1751	G Protein-Coupled Receptor 146	GPR146		GeneCard	
1752	G Protein-Coupled Receptor 162	GPR162			DisGenet
1753	G Protein-Coupled Receptor 182	GPR182		GeneCard	
1754	G Protein-Coupled Receptor 25	GPR25		GeneCard	
1755	G Protein-Coupled Receptor 26	GPR26		GeneCard	
1756	G Protein-Coupled Receptor 3	GPR3		GeneCard	
1757	G Protein-Coupled Receptor 32	GPR32		GeneCard	
1758	G Protein-Coupled Receptor 35	GPR35		GeneCard	
1759	G Protein-Coupled Receptor 37 Like 1	GPR37L1		GeneCard	
1760	G Protein-Coupled Receptor 39	GPR39			DisGenet
1761	G Protein-Coupled Receptor 42 (gene/pseudogene)	GPR42			DisGenet
1762	G Protein-Coupled Receptor 55	GPR55		GeneCard	DisGenet
1763	G Protein-Coupled Receptor 65	GPR65		GeneCard	
1764	G Protein-Coupled Receptor 68	GPR68		GeneCard	

176 5	G Protein-Coupled Receptor Class C Group 5 Member B	GPRC5B		GeneCard	
176 6	G Protein Pathway Suppressor 2	GPS2		GeneCard	
176 7	G Protein Signaling Modulator 2	GPSM2		GeneCard	
176 8	Glutamic--Pyruvic Transaminase	GPT		GeneCard	
176 9	Glutathione Peroxidase 1	GPX1		GeneCard	DisGenet
177 0	Glutathione Peroxidase 3	GPX3		GeneCard	
177 1	Glutathione Peroxidase 4	GPX4		GeneCard	DisGenet
177 2	GRAM Domain Containing 1C	GRAMD1C		GeneCard	
177 3	GRB2 Related Adaptor Protein 2	GRAP2			DisGenet
177 4	Growth Factor Receptor Bound Protein 2	GRB2		GeneCard	
177 5	Gremlin 1, DAN Family BMP Antagonist	GREM1		GeneCard	DisGenet
177 6	Glutamate Ionotropic Receptor AMPA Type Subunit 3	GRIA3		GeneCard	
177 7	Glutamate Ionotropic Receptor Delta Type Subunit 1	GRID1		GeneCard	
177 8	Glutamate Ionotropic Receptor NMDA Type Subunit 3A	GRIN3A		GeneCard	
177 9	Glutamate Receptor Interacting Protein 1	GRIP1		GeneCard	
178 0	G Protein-Coupled Receptor Kinase 2	GRK2		GeneCard	
178 1	G Protein-Coupled Receptor Kinase 4	GRK4		GeneCard	
178 2	G Protein-Coupled Receptor Kinase 6	GRK6		GeneCard	
178 3	Glutamate Metabotropic Receptor 8	GRM8		GeneCard	
178 4	Granulin Precursor	GRN		GeneCard	DisGenet

1785	Gastrin Releasing Peptide	GRP		GeneCard	
1786	Goosecoid Homeobox 2	GSC2		GeneCard	
1787	GSC Divergent Transcript	GSC-DT		GeneCard	DisGenet
1788	Gasdermin E	GSDME		GeneCard	
1789	Glycogen Synthase Kinase 3 Alpha	GSK3A		GeneCard	
1790	Glycogen Synthase Kinase 3 Beta	GSK3B		GeneCard	DisGenet
1791	Gelsolin	GSN		GeneCard	
1792	Glutathione-Disulfide Reductase	GSR		GeneCard	DisGenet
1793	Glutathione Synthetase	GSS		GeneCard	
1794	Glutathione S-Transferase Alpha 4	GSTA4		GeneCard	
1795	Glutathione S-Transferase C-Terminal Domain Containing	GSTCD		GeneCard	
1796	Glutathione S-Transferase Kappa 1	GSTK1			DisGenet
1797	Glutathione S-Transferase Mu 1	GSTM1		GeneCard	DisGenet
1798	Glutathione S-Transferase Mu 2	GSTM2		GeneCard	DisGenet
1799	Glutathione S-Transferase Mu 3	GSTM3		GeneCard	
1800	Glutathione S-Transferase Mu 4	GSTM4		GeneCard	
1801	Glutathione S-Transferase Mu 5	GSTM5		GeneCard	
1802	Glutathione S-Transferase Omega 1	GSTO1		GeneCard	DisGenet
1803	Glutathione S-Transferase Omega 2	GSTO2		GeneCard	
1804	Glutathione S-Transferase Pi 1	GSTP1		GeneCard	

180<br>5	Glutathione S-Transferase Theta 1	GSTT1		GeneCard	DisGenet
180<br>6	Glutathione S-Transferase Zeta 1	GSTZ1		GeneCard	
180<br>7	General Transcription Factor IIE Subunit 2	GTF2E2		GeneCard	
180<br>8	General Transcription Factor IIH Subunit 1	GTF2H1		GeneCard	DisGenet
180<br>9	General Transcription Factor IIH Subunit 2	GTF2H2		GeneCard	
181<br>0	General Transcription Factor IIH Subunit 3	GTF2H3		GeneCard	
181<br>1	General Transcription Factor IIH Subunit 4	GTF2H4		GeneCard	
181<br>2	General Transcription Factor IIIA	GTF3A			DisGenet
181<br>3	Gilles de la Tourette Syndrome	GTS			DisGenet
181<br>4	Guanylate Cyclase 1 Soluble Subunit Alpha 1	GUCY1A1		GeneCard	DisGenet
181<br>5	GULP PTB Domain Containing Engulfment Adaptor 1	GULP1		GeneCard	
181<br>6	Glucuronidase Beta	GUSB		GeneCard	
181<br>7	Glycogenin 1	GYG1		GeneCard	
181<br>8	Glycophorin A (MNS Blood Group)	GYPA		GeneCard	
181<br>9	Glycophorin C (Gerbich Blood Group)	GYPC		GeneCard	DisGenet
182<br>0	Granzyme B	GZMB		GeneCard	DisGenet
182<br>1	H1.0 Linker Histone	H1-0		GeneCard	
182<br>2	H1.1 Linker Histone, Cluster Member	H1-1		GeneCard	
182<br>3	H19 Imprinted Maternally Expressed Transcript	H19	OMIM	GeneCard	DisGenet
182<br>4	H2A Clustered Histone 1	H2AC1		GeneCard	

1825	H2A Clustered Histone 18	H2AC18		GeneCard	
1826	H2A Clustered Histone 20	H2AC20		GeneCard	
1827	H2A.X Variant Histone	H2AX		GeneCard	
1828	H2B Clustered Histone 21	H2BC21		GeneCard	
1829	H2B Clustered Histone 3	H2BC3		GeneCard	
1830	H3.3 Histone A	H3-3A		GeneCard	
1831	H3.3 Histone B	H3-3B		GeneCard	
1832	H3.7 Histone (Putative)	H3-7		GeneCard	
1833	H3 Clustered Histone 1	H3C1		GeneCard	
1834	H3 Clustered Histone 10	H3C10		GeneCard	
1835	H3 Clustered Histone 11	H3C11		GeneCard	
1836	H3 Clustered Histone 12	H3C12		GeneCard	
1837	H3 Clustered Histone 2	H3C2		GeneCard	
1838	H3 Clustered Histone 3	H3C3		GeneCard	
1839	H3 Clustered Histone 4	H3C4		GeneCard	
1840	H3 Clustered Histone 6	H3C6		GeneCard	
1841	H3 Clustered Histone 7	H3C7		GeneCard	
1842	H3 Clustered Histone 8	H3C8		GeneCard	
1843	H3 Histone Pseudogene 10	H3P10			DisGenet
1844	H3 Histone Pseudogene 23	H3P23			DisGenet

184<br>5	H3 Histone Pseudogene 28	H3P28			DisGenet
184<br>6	H3 Histone Pseudogene 31	H3P31			DisGenet
184<br>7	H4 Histone 16	H4-16		GeneCard	DisGenet
184<br>8	H4 Clustered Histone 1	H4C1		GeneCard	DisGenet
184<br>9	H4 Clustered Histone 11	H4C11		GeneCard	DisGenet
185<br>0	H4 Clustered Histone 12	H4C12		GeneCard	DisGenet
185<br>1	H4 Clustered Histone 13	H4C13		GeneCard	DisGenet
185<br>2	H4 Clustered Histone 14	H4C14		GeneCard	DisGenet
185<br>3	H4 Clustered Histone 15	H4C15		GeneCard	DisGenet
185<br>4	H4 Clustered Histone 2	H4C2		GeneCard	DisGenet
185<br>5	H4 Clustered Histone 3	H4C3		GeneCard	DisGenet
185<br>6	H4 Clustered Histone 4	H4C4		GeneCard	DisGenet
185<br>7	H4 Clustered Histone 5	H4C5		GeneCard	DisGenet
185<br>8	H4 Clustered Histone 6	H4C6		GeneCard	DisGenet
185<br>9	H4 Clustered Histone 8	H4C8		GeneCard	DisGenet
186<br>0	H4 Clustered Histone 9	H4C9		GeneCard	DisGenet
186<br>1	Hexose-6-Phosphate Dehydrogenase/Glucose 1-Dehydrogenase	H6PD		GeneCard	DisGenet
186<br>2	Hyaluronan Binding Protein 2	HABP2	OMIM	GeneCard	DisGenet
186<br>3	Hyaluronan Binding Protein 4	HABP4		GeneCard	
186<br>4	3-Hydroxyacyl-CoA Dehydratase 4	HACD4		GeneCard	DisGenet

1865	Hydroxyacyl-CoA Dehydrogenase Trifunctional Multienzyme Complex Subunit Alpha	HADHA		GeneCard	
1866	Hydroxyacyl-CoA Dehydrogenase Trifunctional Multienzyme Complex Subunit Beta	HADHB		GeneCard	
1867	Histidine Ammonia-Lyase	HAL	OMIM	GeneCard	DisGenet
1868	Hepcidin Antimicrobial Peptide	HAMP		GeneCard	DisGenet
1869	Heart And Neural Crest Derivatives Expressed 1	HAND1		GeneCard	
1870	Heart And Neural Crest Derivatives Expressed 2	HAND2		GeneCard	
1871	HAND2 Antisense RNA 1	HAND2-AS1		GeneCard	
1872	Hyaluronan Synthase 1	HAS1		GeneCard	
1873	Hyaluronan Synthase 2	HAS2		GeneCard	DisGenet
1874	HAS2 Antisense RNA 1	HAS2-AS1		GeneCard	
1875	Hyaluronan Synthase 3	HAS3		GeneCard	DisGenet
1876	Histone Acetyltransferase 1	HAT1		GeneCard	DisGenet
1877	Hepatitis A Virus Cellular Receptor 1	HAVCR1		GeneCard	DisGenet
1878	Hepatitis A Virus Cellular Receptor 2	HAVCR2		GeneCard	DisGenet
1879	Hemoglobin Subunit Alpha 1	HBA1		GeneCard	
1880	Hemoglobin Subunit Beta	HBB		GeneCard	
1881	Heparin Binding EGF Like Growth Factor	HBEGF		GeneCard	DisGenet
1882	Hemoglobin Subunit Gamma 2	HBG2		GeneCard	
1883	HMG-Box Transcription Factor 1	HBP1		GeneCard	
1884	HBS1 Like Translational GTPase	HBS1L		GeneCard	

1885	Hydroxycarboxylic Acid Receptor 1	HCAR1		GeneCard	
1886	Hydroxycarboxylic Acid Receptor 2	HCAR2		GeneCard	DisGenet
1887	Hydroxycarboxylic Acid Receptor 3	HCAR3		GeneCard	DisGenet
1888	Hepatocellular Carcinoma Associated Transcript 5	HCCAT5			DisGenet
1889	Host Cell Factor C1	HCFC1		GeneCard	
1890	HLA Complex Group 27	HCG27		GeneCard	
1891	HLA Complex Group 9	HCG9		GeneCard	
1892	HCK Proto-Oncogene, Src Family Tyrosine Kinase	HCK		GeneCard	
1893	Hyperpolarization Activated Cyclic Nucleotide Gated Potassium Channel 4	HCN4		GeneCard	
1894	Hypocretin Neuropeptide Precursor	HCRT		GeneCard	DisGenet
1895	Histone Deacetylase 1	HDAC1		GeneCard	DisGenet
1896	Histone Deacetylase 2	HDAC2		GeneCard	
1897	Histone Deacetylase 3	HDAC3		GeneCard	DisGenet
1898	Histone Deacetylase 4	HDAC4		GeneCard	
1899	Histone Deacetylase 5	HDAC5		GeneCard	DisGenet
1900	Histone Deacetylase 6	HDAC6		GeneCard	DisGenet
1901	Histone Deacetylase 7	HDAC7		GeneCard	
1902	Histone Deacetylase 9	HDAC9		GeneCard	DisGenet
1903	Histidine Decarboxylase	HDC		GeneCard	DisGenet
1904	HDGF Like 1	HDGFL1		GeneCard	

1905	High-Density Lipoprotein Binding Protein	HDLBP		GeneCard	DisGenet
1906	High-Density Lipoprotein Cholesterol, Low Serum, 3	HDLC3		GeneCard	
1907	High-Density Lipoprotein Cholesterol Level QTL On Chromosome 8	HDLCQ2		GeneCard	
1908	HELLP Associated Long Non-Coding RNA	HELLPAR		GeneCard	
1909	Helicase, Lymphoid Specific	HELLS		GeneCard	
1910	HECT And RLD Domain Containing E3 Ubiquitin Protein Ligase 5	HERC5		GeneCard	
1911	HECT And RLD Domain Containing E3 Ubiquitin Protein Ligase Family Member 6	HERC6		GeneCard	
1912	Homocysteine Inducible ER Protein with Ubiquitin Like Domain 1	HERPUD1		GeneCard	
1913	Hes Family BHLH Transcription Factor 1	HES1		GeneCard	DisGenet
1914	HEXIM P-TEFb Complex Subunit 1	HEXIM1		GeneCard	DisGenet
1915	Hes Related Family BHLH Transcription Factor with YRPW Motif 2	HEY2		GeneCard	
1916	Homeostatic Iron Regulator	HFE		GeneCard	DisGenet
1917	Helicase For Meiosis 1	HFM1		GeneCard	
1918	Homogentisate 1,2-Dioxygenase	HGD		GeneCard	DisGenet
1919	Hepatocyte Growth Factor	HGF		GeneCard	DisGenet
1920	HGF Activator	HGFAC		GeneCard	
1921	Hepatocyte Growth Factor-Regulated Tyrosine Kinase Substrate	HGS			DisGenet
1922	Hematopoietically Expressed Homeobox	HHEX		GeneCard	
1923	Hedgehog Interacting Protein	HHIP		GeneCard	
1924	HHIP Antisense RNA 1	HHIP-AS1		GeneCard	

192<br>5	HHIP Like 1	HHIPL1		GeneCard	DisGenet
192<br>6	Hypoxia Inducible Factor 1 Subunit Alpha	HIF1A		GeneCard	DisGenet
192<br>7	HIF1A Antisense RNA 1	HIF1A-AS1		GeneCard	DisGenet
192<br>8	HIF1A Antisense RNA 2	HIF1A-AS2		GeneCard	
192<br>9	HIG1 Hypoxia Inducible Domain Family Member 1A	HIGD1A		GeneCard	
193<br>0	Hypoxia Inducible Lipid Droplet Associated	HILPDA			DisGenet
193<br>1	Homeodomain Interacting Protein Kinase 3	HIPK3		GeneCard	
193<br>2	Hexokinase 1	HK1		GeneCard	
193<br>3	Hexokinase 2	HK2		GeneCard	
193<br>4	Major Histocompatibility Complex, Class I, A	HLA-A		GeneCard	DisGenet
193<br>5	Major Histocompatibility Complex, Class I, B	HLA-B		GeneCard	DisGenet
193<br>6	Major Histocompatibility Complex, Class I, C	HLA-C		GeneCard	DisGenet
193<br>7	Major Histocompatibility Complex, Class II, DM Alpha	HLA-DMA		GeneCard	
193<br>8	Major Histocompatibility Complex, Class II, DO Alpha	HLA-DOA		GeneCard	
193<br>9	Major Histocompatibility Complex, Class II, DP Alpha 1	HLA-DPA1		GeneCard	
194<br>0	Major Histocompatibility Complex, Class II, DP Beta 1	HLA-DPB1		GeneCard	
194<br>1	Major Histocompatibility Complex, Class II, DQ Alpha 1	HLA-DQA1		GeneCard	
194<br>2	Major Histocompatibility Complex, Class II, DQ Beta 1	HLA-DQB1		GeneCard	
194<br>3	Major Histocompatibility Complex, Class II, DR Alpha	HLA-DRA		GeneCard	
194<br>4	Major Histocompatibility Complex, Class II, DR Beta 1	HLA-DRB1		GeneCard	

1945	Major Histocompatibility Complex, Class I, G	HLA-G		GeneCard	
1946	Hyperkeratosis Lenticularis Perstans	HLP			DisGenet
1947	Helicase Like Transcription Factor	HLTF		GeneCard	
1948	H2.0 Like Homeobox	HLX		GeneCard	
1949	HLX Antisense RNA 1	HLX-AS1		GeneCard	
1950	Homeobox Containing 1	HMBOX1		GeneCard	DisGenet
1951	Hemicentin 1	HMCN1		GeneCard	
1952	High Mobility Group AT-Hook 1	HMGA1		GeneCard	DisGenet
1953	High Mobility Group AT-Hook 2	HMGA2		GeneCard	DisGenet
1954	High Mobility Group Box 1	HMGB1		GeneCard	DisGenet
1955	3-Hydroxy-3-Methylglutaryl-CoA Lyase	HMGCL		GeneCard	
1956	3-Hydroxy-3-Methylglutaryl-CoA Reductase	HMGCR		GeneCard	DisGenet
1957	High Mobility Group Nucleosome Binding Domain 1	HMGN1		GeneCard	
1958	Heme Oxygenase 1	HMOX1		GeneCard	DisGenet
1959	Heme Oxygenase 2	HMOX2		GeneCard	
1960	HNF1 Homeobox A	HNF1A	OMIM	GeneCard	DisGenet
1961	HNF1 Homeobox B	HNF1B		GeneCard	
1962	Hepatocyte Nuclear Factor 4 Alpha	HNF4A		GeneCard	DisGenet
1963	Hypertensive Nephropathy	HNP1		GeneCard	DisGenet
1964	Heterogeneous Nuclear Ribonucleoprotein A1	HNRNPA1		GeneCard	

1965	Heterogeneous Nuclear Ribonucleoprotein A1 Pseudogene 10	HNRNPA1P10		GeneCard	
1966	Heterogeneous Nuclear Ribonucleoprotein A1 Pseudogene 4	HNRNPA1P4		GeneCard	
1967	Heterogeneous Nuclear Ribonucleoprotein A2/B1	HNRNPA2B1		GeneCard	
1968	Heterogeneous Nuclear Ribonucleoprotein A/B	HNRNPAB		GeneCard	
1969	Heterogeneous Nuclear Ribonucleoprotein C	HNRNPC		GeneCard	
1970	Heterogeneous Nuclear Ribonucleoprotein K	HNRNPK		GeneCard	
1971	Heterogeneous Nuclear Ribonucleoprotein L	HNRNPL		GeneCard	
1972	Heterogeneous Nuclear Ribonucleoprotein U	HNRNPU			DisGenet
1973	HNRNPUL2-BSCL2	HNRNPUL2-BSCL2		GeneCard	
1974	Homer Scaffold Protein 1	HOMER1		GeneCard	
1975	Homer Scaffold Protein 2	HOMER2		GeneCard	
1976	HOP Homeobox	HOPX		GeneCard	
1977	HOX Transcript Antisense RNA	HOTAIR		GeneCard	DisGenet
1978	HOXA Distal Transcript Antisense RNA	HOTTIP		GeneCard	
1979	Homeobox A1	HOXA1		GeneCard	DisGenet
1980	Homeobox A11	HOXA11		GeneCard	
1981	Homeobox A5	HOXA5		GeneCard	
1982	Homeobox A9	HOXA9		GeneCard	
1983	HOXA Cluster Antisense RNA 2	HOXA-AS2		GeneCard	
1984	HOXA Cluster Antisense RNA 3	HOXA-AS3	OMIM	GeneCard	DisGenet

1985	Homeobox B1	HOXB1		GeneCard	
1986	Homeobox B7	HOXB7		GeneCard	
1987	Homeobox B9	HOXB9		GeneCard	DisGenet
1988	Homeobox C6	HOXC6		GeneCard	
1989	Homeobox C9	HOXC9		GeneCard	
1990	HOXC Cluster Antisense RNA 1	HOXC-AS1		GeneCard	
1991	Haptoglobin	HP		GeneCard	DisGenet
1992	Phosphate Binding Apolipoprotein	HPBP		GeneCard	
1993	4-Hydroxyphenylpyruvate Dioxygenase	HPD		GeneCard	
1994	Hematopoietic Prostaglandin D Synthase	HPGDS			DisGenet
1995	Haptoglobin-Related Protein	HPR		GeneCard	
1996	Hypoxanthine Phosphoribosyltransferase 1	HPRT1		GeneCard	
1997	HPS5 Biogenesis of Lysosomal Organelles Complex 2 Subunit 2	HPS5		GeneCard	
1998	Heparanase	HPSE		GeneCard	DisGenet
1999	Hemopexin	HPX		GeneCard	DisGenet
2000	HRas Proto-Oncogene, GTPase	HRAS		GeneCard	
2001	Histidine Rich Calcium Binding Protein	HRC		GeneCard	
2002	Histidine Rich Glycoprotein	HRG		GeneCard	
2003	Histamine Receptor H1	HRH1		GeneCard	DisGenet
2004	Histamine Receptor H2	HRH2		GeneCard	DisGenet

200<br>5	Hornerin	HRNR		GeneCard	
200<br>6	Homologous Recombination Factor With OB-Fold	HROB		GeneCard	
200<br>7	Heparan Sulfate-Glucosamine 3-Sulfotransferase 1	HS3ST1		GeneCard	DisGenet
200<br>8	Hydroxysteroid 11-Beta Dehydrogenase 1	HSD11B1		GeneCard	DisGenet
200<br>9	Hydroxysteroid 11-Beta Dehydrogenase 2	HSD11B2		GeneCard	
201<br>0	Hydroxysteroid 17-Beta Dehydrogenase 4	HSD17B4		GeneCard	
201<br>1	Hydroxysteroid 17-Beta Dehydrogenase 6	HSD17B6		GeneCard	
201<br>2	Hydroxy-Delta-5-Steroid Dehydrogenase, 3 Beta- And Steroid Delta-Isomerase 1	HSD3B1		GeneCard	
201<br>3	Hydroxy-Delta-5-Steroid Dehydrogenase, 3 Beta- And Steroid Delta-Isomerase 7	HSD3B7		GeneCard	
201<br>4	Hydroxysteroid Dehydrogenase Like 2	HSDL2		GeneCard	
201<br>5	Heat Shock Transcription Factor 1	HSF1		GeneCard	DisGenet
201<br>6	Hematopoietic SH2 Domain	HSH2D			DisGenet
201<br>7	Heat Shock Protein 90 Alpha Family Class A Member 1	HSP90AA1		GeneCard	DisGenet
201<br>8	Heat Shock Protein 90 Alpha Family Class B Member 1	HSP90AB1		GeneCard	
201<br>9	Heat Shock Protein 90 Beta Family Member 1	HSP90B1		GeneCard	DisGenet
202<br>0	Heat Shock Protein Family A (Hsp70) Member 12A	HSPA12A	OMIM	GeneCard	
202<br>1	Heat Shock Protein Family A (Hsp70) Member 12B	HSPA12B		GeneCard	
202<br>2	Heat Shock Protein Family A (Hsp70) Member 14	HSPA14		GeneCard	DisGenet
202<br>3	Heat Shock Protein Family A (Hsp70) Member 1A	HSPA1A		GeneCard	DisGenet
202<br>4	Heat Shock Protein Family A (Hsp70) Member 1B	HSPA1B		GeneCard	DisGenet

2025	heat shock protein family A (Hsp70) member 2	HSPA2			DisGenet
2026	Heat Shock Protein Family A (Hsp70) Member 4	HSPA4		GeneCard	DisGenet
2027	Heat Shock Protein Family A (Hsp70) Member 5	HSPA5		GeneCard	DisGenet
2028	Heat Shock Protein Family A (Hsp70) Member 6	HSPA6		GeneCard	
2029	Heat Shock Protein Family A (Hsp70) Member 7 (Pseudogene)	HSPA7		GeneCard	
2030	Heat Shock Protein Family A (Hsp70) Member 8	HSPA8		GeneCard	
2031	Heat Shock Protein Family B (Small) Member 1	HSPB1	OMIM	GeneCard	DisGenet
2032	Heat Shock Protein Family B (Small) Member 2	HSPB2		GeneCard	DisGenet
2033	Heat Shock Protein Family B (Small) Member 3	HSPB3			DisGenet
2034	Heat Shock Protein Family B (Small) Member 6	HSPB6		GeneCard	
2035	Heat Shock Protein Family B (Small) Member 7	HSPB7		GeneCard	
2036	Heat Shock Protein Family B (Small) Member 8	HSPB8			DisGenet
2037	Heat Shock Protein Family D (Hsp60) Member 1	HSPD1		GeneCard	DisGenet
2038	Heat Shock Protein Family E (Hsp10) Member 1	HSPE1		GeneCard	
2039	Heparan Sulfate Proteoglycan 2	HSPG2		GeneCard	DisGenet
2040	HIV-1 TAT Interactive Protein 2	HTATIP2			DisGenet
2041	Histatin 3	HTN3		GeneCard	
2042	5-Hydroxytryptamine Receptor 1A	HTR1A		GeneCard	
2043	5-Hydroxytryptamine Receptor 2A	HTR2A		GeneCard	
2044	5-Hydroxytryptamine Receptor 3A	HTR3A		GeneCard	

2045	5-Hydroxytryptamine Receptor 4	HTR4		GeneCard	
2046	HtrA Serine Peptidase 1	HTRA1		GeneCard	
2047	Huntingtin	HTT		GeneCard	
2048	Hepatocellular Carcinoma Up-Regulated Long Non-Coding RNA	HULC		GeneCard	
2049	Hyaluronidase 1	HYAL1		GeneCard	
2050	Hyaluronidase 2	HYAL2		GeneCard	
2051	HYLS1 Centriolar and Ciliogenesis Associated	HYLS1			DisGenet
2052	Hypoxia Up-Regulated 1	HYOU1		GeneCard	
2053	Islet Amyloid Polypeptide	IAPP		GeneCard	
2054	Integrin Binding Sialoprotein	IBSP		GeneCard	
2055	Islet Cell Autoantigen 1	ICA1		GeneCard	
2056	Intercellular Adhesion Molecule 1	ICAM1		GeneCard	DisGenet
2057	Intercellular Adhesion Molecule 2	ICAM2		GeneCard	
2058	Intercellular Adhesion Molecule 3	ICAM3		GeneCard	
2059	Isoprenylcysteine Carboxyl Methyltransferase	ICMT		GeneCard	
2060	Inducible T Cell Costimulator	ICOS		GeneCard	DisGenet
2061	Inducible T Cell Costimulator Ligand	ICOSLG		GeneCard	
2062	Inhibitor Of DNA Binding 1, HLH Protein	ID1		GeneCard	DisGenet
2063	Inhibitor Of DNA Binding 3, HLH Protein	ID3		GeneCard	DisGenet
2064	Insulin Degrading Enzyme	IDE		GeneCard	

2065	Isocitrate Dehydrogenase (NADP(+)) 1	IDH1		GeneCard	
2066	Isocitrate Dehydrogenase (NADP(+)) 2	IDH2		GeneCard	DisGenet
2067	Isopentenyl-Diphosphate Delta Isomerase 1	IDI1		GeneCard	
2068	IDI2 Antisense RNA 1	IDI2-AS1		GeneCard	
2069	Indoleamine 2,3-Dioxygenase 1	IDO1		GeneCard	DisGenet
2070	Iduronate 2-Sulfatase	IDS		GeneCard	
2071	Immediate Early Response 3	IER3		GeneCard	
2072	Interferon Gamma Inducible Protein 16	IFI16		GeneCard	
2073	Interferon Alpha Inducible Protein 27	IFI27		GeneCard	DisGenet
2074	Interferon Induced Protein 35	IFI35		GeneCard	
2075	Interferon Induced Protein 44 Like	IFI44L		GeneCard	
2076	Interferon Induced with Helicase C Domain 1	IFIH1		GeneCard	
2077	Interferon Induced Protein with Tetratricopeptide Repeats 3	IFIT3		GeneCard	
2078	Interferon, Type 1, Cluster	IFN1@		GeneCard	
2079	Interferon Alpha 1	IFNA1		GeneCard	DisGenet
2080	Interferon Alpha 13	IFNA13			DisGenet
2081	Interferon Alpha 2	IFNA2		GeneCard	
2082	Interferon Alpha 21	IFNA21		GeneCard	
2083	Interferon Alpha and Beta Receptor Subunit 1	IFNAR1		GeneCard	
2084	Interferon Alpha and Beta Receptor Subunit 2	IFNAR2		GeneCard	

2085	Interferon Beta 1	IFNB1		GeneCard	
2086	Interferon Gamma	IFNG		GeneCard	DisGenet
2087	Interferon Gamma Receptor 1	IFNGR1		GeneCard	
2088	Interferon Gamma Receptor 2	IFNGR2		GeneCard	
2089	Interferon Kappa	IFNK		GeneCard	
2090	Interferon Related Developmental Regulator 1	IFRD1		GeneCard	
2091	Intraflagellar Transport 20	IFT20		GeneCard	
2092	Intraflagellar Transport 80	IFT80		GeneCard	
2093	Intraflagellar Transport 88	IFT88		GeneCard	
2094	Immunoglobulin Binding Protein 1	IGBP1		GeneCard	
2095	Immunoglobulin E Concentration, Serum	IGES		GeneCard	
2096	Insulin Like Growth Factor 1	IGF1		GeneCard	DisGenet
2097	Insulin Like Growth Factor 1 Receptor	IGF1R		GeneCard	DisGenet
2098	Insulin Like Growth Factor 2	IGF2	OMIM	GeneCard	DisGenet
2099	IGF2 Antisense RNA	IGF2-AS		GeneCard	
2100	Insulin Like Growth Factor 2 MRNA Binding Protein 1	IGF2BP1		GeneCard	
2101	Insulin Like Growth Factor 2 MRNA Binding Protein 2	IGF2BP2		GeneCard	
2102	Insulin Like Growth Factor 2 Receptor	IGF2R		GeneCard	DisGenet
2103	Insulin Like Growth Factor Binding Protein Acid Labile Subunit	IGFALS		GeneCard	
2104	Insulin Like Growth Factor Binding Protein 1	IGFBP1		GeneCard	DisGenet

210<br>5	Insulin Like Growth Factor 2 MRNA Binding Protein 2	IGFBP2		GeneCard	
210<br>6	Insulin Like Growth Factor 2 Receptor	IGFBP3		GeneCard	DisGenet
210<br>7	Insulin Like Growth Factor Binding Protein Acid Labile Subunit	IGFBP4		GeneCard	
210<br>8	Insulin Like Growth Factor Binding Protein 1	IGFBP5		GeneCard	DisGenet
210<br>9	Insulin Like Growth Factor Binding Protein 2	IGFBP7		GeneCard	DisGenet
211<br>0	Insulin Like Growth Factor Binding Protein 3	IGHE		GeneCard	DisGenet
211<br>1	Insulin Like Growth Factor Binding Protein 4	IGHG1		GeneCard	
211<br>2	Insulin Like Growth Factor Binding Protein 5	IGHG3		GeneCard	DisGenet
211<br>3	Insulin Like Growth Factor Binding Protein 7	IGKV@		GeneCard	
211<br>4	Immunoglobulin Heavy Constant Epsilon	IGKV2D-29		GeneCard	DisGenet
211<br>5	Immunoglobulin Heavy Constant Gamma 1 (G1m Marker)	IGLV2-18		GeneCard	DisGenet
211<br>6	Immunoglobulin Kappa Variable Cluster	IGSF1		GeneCard	DisGenet
211<br>7	IK Cytokine	IK		GeneCard	
211<br>8	Inhibitor Of Nuclear Factor Kappa B Kinase Subunit Beta	IKBKB		GeneCard	DisGenet
211<br>9	Inhibitor Of Nuclear Factor Kappa B Kinase Subunit Epsilon	IKBKE		GeneCard	
212<br>0	Inhibitor Of Nuclear Factor Kappa B Kinase Regulatory Subunit Gamma	IKBKG		GeneCard	
212<br>1	Interleukin-10	IL10	OMIM	GeneCard	DisGenet
212<br>2	Interleukin 10 Receptor Subunit Alpha	IL10RA		GeneCard	
212<br>3	Interleukin 11	IL11		GeneCard	DisGenet
212<br>4	Interleukin 12A	IL12A		GeneCard	

212<br>5	IL12A Antisense RNA 1	IL12A-AS1		GeneCard	
212<br>6	Interleukin 12B	IL12B		GeneCard	
212<br>7	Interleukin 13	IL13		GeneCard	DisGenet
212<br>8	Interleukin 15	IL15		GeneCard	DisGenet
212<br>9	Interleukin 16	IL16		GeneCard	
213<br>0	Interleukin 17A	IL17A	OMIM	GeneCard	DisGenet
213<br>1	Interleukin 17B	IL17B		GeneCard	DisGenet
213<br>2	Interleukin 17D	IL17D			DisGenet
213<br>3	Interleukin 17F	IL17F		GeneCard	DisGenet
213<br>4	Interleukin 17 Receptor A	IL17RA		GeneCard	
213<br>5	Interleukin 17 Receptor B	IL17RB		GeneCard	
213<br>6	Interleukin 17 Receptor C	IL17RC		GeneCard	
213<br>7	Interleukin-18	IL18	OMIM	GeneCard	DisGenet
213<br>8	Interleukin 18 Binding Protein	IL18BP		GeneCard	DisGenet
213<br>9	Interleukin 18 Receptor 1	IL18R1		GeneCard	
214<br>0	Interleukin 18 Receptor Accessory Protein	IL18RAP		GeneCard	
214<br>1	Interleukin 19	IL19		GeneCard	DisGenet
214<br>2	Interleukin 1 Alpha	IL1A		GeneCard	DisGenet
214<br>3	Interleukin-1, beta	IL1B	OMIM	GeneCard	DisGenet
214<br>4	Interleukin 1 Family Member 10	IL1F10			DisGenet

2145	Interleukin 1 Receptor Type 1	IL1R1		GeneCard	
2146	Interleukin 1 Receptor Type 2	IL1R2		GeneCard	
2147	Interleukin 1 Receptor Accessory Protein	IL1RAP		GeneCard	
2148	Interleukin 1 Receptor Accessory Protein Like 2	IL1RAPL2		GeneCard	
2149	Interleukin 1 Receptor Like 1	IL1RL1		GeneCard	DisGenet
2150	Interleukin 1 Receptor Antagonist	IL1RN		GeneCard	DisGenet
2151	Interleukin 2	IL2		GeneCard	DisGenet
2152	Interleukin 20	IL20		GeneCard	DisGenet
2153	Interleukin 20 Receptor Subunit Alpha	IL20RA		GeneCard	
2154	Interleukin 20 Receptor Subunit Beta	IL20RB		GeneCard	
2155	Interleukin 21	IL21		GeneCard	DisGenet
2156	Interleukin 21 Receptor	IL21R		GeneCard	
2157	Interleukin 22	IL22		GeneCard	DisGenet
2158	Interleukin 22 Receptor Subunit Alpha 1	IL22RA1		GeneCard	
2159	Interleukin 23 Subunit Alpha	IL23A		GeneCard	DisGenet
2160	Interleukin 23 Receptor	IL23R		GeneCard	DisGenet
2161	Interleukin 24	IL24		GeneCard	
2162	Interleukin 25	IL25		GeneCard	DisGenet
2163	Interleukin 26	IL26		GeneCard	
2164	Interleukin 27	IL27		GeneCard	DisGenet

216<br>5	Interleukin 27 Receptor Subunit Alpha	IL27RA			DisGenet
216<br>6	Interleukin 2 Receptor Subunit Alpha	IL2RA		GeneCard	DisGenet
216<br>7	Interleukin 2 Receptor Subunit Beta	IL2RB		GeneCard	
216<br>8	Interleukin 3	IL3		GeneCard	
216<br>9	Interleukin 32	IL32		GeneCard	DisGenet
217<br>0	Interleukin 33	IL33		GeneCard	DisGenet
217<br>1	Interleukin 34	IL34		GeneCard	DisGenet
217<br>2	Interleukin 36 Alpha	IL36A		GeneCard	
217<br>3	Interleukin 37	IL37		GeneCard	DisGenet
217<br>4	Interleukin 4	IL4		GeneCard	DisGenet
217<br>5	Interleukin 4 Receptor	IL4R		GeneCard	
217<br>6	Interleukin 5	IL5		GeneCard	DisGenet
217<br>7	Interleukin 5 Receptor Subunit Alpha	IL5RA		GeneCard	
217<br>8	Interleukin-6 (interferon, beta-2)	IL6	OMIM	GeneCard	DisGenet
217<br>9	IL6 Antisense RNA 1	IL6-AS1		GeneCard	
218<br>0	Interleukin 6 Receptor	IL6R		GeneCard	DisGenet
218<br>1	Interleukin 6 Cytokine Family Signal Transducer	IL6ST		GeneCard	DisGenet
218<br>2	Interleukin 7	IL7		GeneCard	DisGenet
218<br>3	Interleukin 7 Receptor	IL7R		GeneCard	
218<br>4	Interleukin 9	IL9		GeneCard	DisGenet

218 5	Interleukin Enhancer Binding Factor 3	ILF3		GeneCard	
218 6	Integrin Linked Kinase	ILK		GeneCard	DisGenet
218 7	Inner Membrane Mitochondrial Protein	IMMT		GeneCard	
218 8	Inhibin Subunit Alpha	INHA		GeneCard	
218 9	Inhibin Subunit Beta A	INHBA		GeneCard	
219 0	INO80 complex, subunit D	INO80D	OMIM	GeneCard	
219 1	Inositol Polyphosphate-5-Phosphatase D	INPP5D		GeneCard	DisGenet
219 2	Inositol Polyphosphate-5-Phosphatase K	INPP5K		GeneCard	DisGenet
219 3	Inositol Polyphosphate Phosphatase Like 1	INPPL1		GeneCard	
219 4	Insulin	INS	OMIM	GeneCard	DisGenet
219 5	Insulin Induced Gene 1	INSIG1		GeneCard	
219 6	Insulin Induced Gene 2	INSIG2		GeneCard	DisGenet
219 7	Insulin Like 6	INSL6		GeneCard	
219 8	Insulin Receptor	INSR		GeneCard	DisGenet
219 9	Integrator Complex Subunit 12	INTS12		GeneCard	
220 0	Inturned Planar Cell Polarity Protein	INTU			DisGenet
220 1	Inversin	INVS		GeneCard	
220 2	Inositol Hexakisphosphate Kinase 3	IP6K3		GeneCard	
220 3	Inositol Polyphosphate Multikinase	IPMK		GeneCard	
220 4	Importin 5	IPO5		GeneCard	

2205	IPO9 Antisense RNA 1	IPO9-AS1		GeneCard	
2206	IQ Motif Containing B1	IQCB1		GeneCard	
2207	Inositol 1,4,5-Triphosphate Receptor Associated 1	IRAG1		GeneCard	
2208	Interleukin 1 Receptor Associated Kinase 1	IRAK1		GeneCard	DisGenet
2209	Interleukin 1 Receptor Associated Kinase 1 Binding Protein 1	IRAK1BP1		GeneCard	
2210	Interleukin 1 Receptor Associated Kinase 4	IRAK4		GeneCard	DisGenet
2211	Interferon Regulatory Factor 1	IRF1	OMIM	GeneCard	DisGenet
2212	IRF1 Antisense RNA 1	IRF1-AS1		GeneCard	
2213	Interferon Regulatory Factor 2	IRF2			DisGenet
2214	Interferon Regulatory Factor 2 Binding Protein 2	IRF2BP2		GeneCard	DisGenet
2215	Interferon Regulatory Factor 3	IRF3		GeneCard	DisGenet
2216	Interferon Regulatory Factor 4	IRF4		GeneCard	
2217	Interferon Regulatory Factor 5	IRF5		GeneCard	DisGenet
2218	interferon regulatory factor 6	IRF6			DisGenet
2219	Interferon Regulatory Factor 7	IRF7		GeneCard	
2220	Interferon Regulatory Factor 8	IRF8		GeneCard	DisGenet
2221	Interferon Regulatory Factor 9	IRF9		GeneCard	
2222	Immunity Related GTPase M	IRGM		GeneCard	DisGenet
2223	Insulin Receptor Substrate 1	IRS1	OMIM	GeneCard	DisGenet
2224	Insulin Receptor Substrate 2	IRS2			DisGenet

2225	ISG15 Ubiquitin Like Modifier	ISG15		GeneCard	
2226	Interferon Stimulated Exonuclease Gene 20	ISG20			DisGenet
2227	ISL LIM Homeobox 1	ISL1		GeneCard	
2228	Isthmin 1	ISM1		GeneCard	
2229	Integrin Subunit Alpha 1	ITGA1		GeneCard	
2230	Integrin Subunit Alpha 11	ITGA11		GeneCard	
2231	Integrin Subunit Alpha 2	ITGA2		GeneCard	DisGenet
2232	Integrin Subunit Alpha 2b	ITGA2B		GeneCard	DisGenet
2233	Integrin Subunit Alpha 3	ITGA3		GeneCard	
2234	Integrin Subunit Alpha 4	ITGA4		GeneCard	
2235	Integrin Subunit Alpha 5	ITGA5		GeneCard	DisGenet
2236	Integrin Subunit Alpha 6	ITGA6		GeneCard	
2237	Integrin Subunit Alpha D	ITGAD		GeneCard	DisGenet
2238	Integrin Subunit Alpha E	ITGAE			DisGenet
2239	Integrin Subunit Alpha L	ITGAL		GeneCard	DisGenet
2240	Integrin Subunit Alpha M	ITGAM		GeneCard	DisGenet
2241	Integrin Subunit Alpha V	ITGAV		GeneCard	DisGenet
2242	Integrin Subunit Alpha X	ITGAX		GeneCard	DisGenet
2243	Integrin Subunit Beta 1	ITGB1		GeneCard	
2244	Integrin Subunit Beta 1 Binding Protein 2	ITGB1BP2		GeneCard	

224<br>5	Integrin Subunit Beta 2	ITGB2		GeneCard	DisGenet
224<br>6	Integrin Subunit Beta 3	ITGB3		GeneCard	DisGenet
224<br>7	Integrin Subunit Beta 4	ITGB4		GeneCard	DisGenet
224<br>8	Integrin Subunit Beta 5	ITGB5		GeneCard	DisGenet
224<br>9	Integrin Subunit Beta 7	ITGB7	OMIM		
225<br>0	Inter-Alpha-Trypsin Inhibitor Heavy Chain 1	ITIH1		GeneCard	
225<br>1	Inter-Alpha-Trypsin Inhibitor Heavy Chain 3	ITIH3		GeneCard	
225<br>2	Inter-Alpha-Trypsin Inhibitor Heavy Chain 4	ITIH4		GeneCard	DisGenet
225<br>3	Intelectin 1	ITLN1		GeneCard	
225<br>4	Integral Membrane Protein 2B	ITM2B		GeneCard	
225<br>5	Inositol-Trisphosphate 3-Kinase C	ITPKC		GeneCard	
225<br>6	Inositol 1,4,5-Trisphosphate Receptor Type 1	ITPR1		GeneCard	DisGenet
225<br>7	Inositol 1,4,5-Trisphosphate Receptor Type 2	ITPR2			DisGenet
225<br>8	Inositol 1,4,5-Trisphosphate Receptor Type 3	ITPR3		GeneCard	DisGenet
225<br>9	Isovaleryl-CoA Dehydrogenase	IVD			DisGenet
226<br>0	Iodotyrosine Deiodinase	IYD		GeneCard	
226<br>1	Izumo Sperm-Egg Fusion 1	IZUMO1		GeneCard	
226<br>2	Jagged Canonical Notch Ligand 1	JAG1		GeneCard	DisGenet
226<br>3	Janus Kinase 1	JAK1		GeneCard	
226<br>4	Janus Kinase 2	JAK2		GeneCard	DisGenet

2265	Janus Kinase 3	JAK3		GeneCard	
2266	Junctional Adhesion Molecule 3	JAM3		GeneCard	DisGenet
2267	Junction Adhesion Molecule Like	JAML		GeneCard	
2268	JAZF Zinc Finger 1	JAZF1		GeneCard	
2269	Junctional Cadherin 5 Associated	JCAD		GeneCard	DisGenet
2270	Jun Dimerization Protein 2	JDP2		GeneCard	
2271	Jumonji Domain Containing 1C	JMJD1C		GeneCard	
2272	Jumonji Domain Containing 6, Arginine Demethylase And Lysine Hydroxylase	JMJD6		GeneCard	
2273	Junctophilin 1	JPH1		GeneCard	
2274	Junctophilin 2	JPH2		GeneCard	
2275	Junctophilin 3	JPH3		GeneCard	DisGenet
2276	Jun Proto-Oncogene, AP-1 Transcription Factor Subunit	JUN		GeneCard	DisGenet
2277	JunB Proto-Oncogene, AP-1 Transcription Factor Subunit	JUNB			DisGenet
2278	JunD Proto-Oncogene, AP-1 Transcription Factor Subunit	JUND			DisGenet
2279	Junction Plakoglobin	JUP		GeneCard	DisGenet
2280	Kalirin RhoGEF Kinase	KALRN	OMIM	GeneCard	DisGenet
2281	KN Motif And Ankyrin Repeat Domains 1	KANK1		GeneCard	
2282	Lysine Acetyltransferase 2B	KAT2B		GeneCard	
2283	Lysine Acetyltransferase 5	KAT5			DisGenet
2284	Katanin Catalytic Subunit A1 Like 1	KATNAL1		GeneCard	

228<br>5	Kelch Repeat and BTB Domain Containing 3	KBTBD3		GeneCard	
228<br>6	Keratoconus Gene 6	KC6		GeneCard	
228<br>7	Potassium Voltage-Gated Channel Subfamily A Member 1	KCNA1		GeneCard	
228<br>8	Potassium Voltage-Gated Channel Subfamily A Member 2	KCNA2		GeneCard	
228<br>9	Potassium Voltage-Gated Channel Subfamily A Member 3	KCNA3		GeneCard	
229<br>0	Potassium Voltage-Gated Channel Subfamily A Member 5	KCNA5		GeneCard	
229<br>1	Potassium Voltage-Gated Channel Subfamily E Regulatory Subunit 1	KCNE1		GeneCard	
229<br>2	Potassium Voltage-Gated Channel Subfamily E Regulatory Subunit 2	KCNE2		GeneCard	DisGenet
229<br>3	Potassium Voltage-Gated Channel Subfamily H Member 2	KCNH2		GeneCard	
229<br>4	Potassium Voltage-Gated Channel Subfamily H Member 4	KCNH4			DisGenet
229<br>5	Potassium Voltage-Gated Channel Subfamily H Member 5	KCNH5		GeneCard	
229<br>6	Potassium Voltage-Gated Channel Subfamily H Member 8	KCNH8			DisGenet
229<br>7	Potassium Voltage-Gated Channel Interacting Protein 1	KCNIP1		GeneCard	
229<br>8	Potassium Voltage-Gated Channel Interacting Protein 2	KCNIP2		GeneCard	
229<br>9	Potassium Inwardly Rectifying Channel Subfamily J Member 1	KCNJ1		GeneCard	
230<br>0	Potassium Inwardly Rectifying Channel Subfamily J Member 11	KCNJ11		GeneCard	DisGenet
230<br>1	Potassium Inwardly Rectifying Channel Subfamily J Member 12	KCNJ12		GeneCard	
230<br>2	Potassium Inwardly Rectifying Channel Subfamily J Member 2	KCNJ2		GeneCard	
230<br>3	Potassium Inwardly Rectifying Channel Subfamily J Member 5	KCNJ5		GeneCard	
230<br>4	Potassium Inwardly Rectifying Channel Subfamily J Member 8	KCNJ8		GeneCard	

2305	Potassium Two Pore Domain Channel Subfamily K Member 1	KCNK1		GeneCard	
2306	Potassium Two Pore Domain Channel Subfamily K Member 13	KCNK13		GeneCard	
2307	Potassium Two Pore Domain Channel Subfamily K Member 2	KCNK2		GeneCard	
2308	Potassium Two Pore Domain Channel Subfamily K Member 3	KCNK3		GeneCard	
2309	Potassium Two Pore Domain Channel Subfamily K Member 9	KCNK9		GeneCard	
2310	Potassium Calcium-Activated Channel Subfamily M Alpha 1	KCNMA1		GeneCard	
2311	Potassium Calcium-Activated Channel Subfamily M Regulatory Beta Subunit 1	KCNMB1		GeneCard	DisGenet
2312	Potassium Calcium-Activated Channel Subfamily N Member 3	KCNN3		GeneCard	
2313	Potassium Calcium-Activated Channel Subfamily N Member 4	KCNN4		GeneCard	
2314	Potassium Voltage-Gated Channel Subfamily Q Member 1	KCNQ1		GeneCard	
2315	KCNQ1 Opposite Strand/Antisense Transcript 1	KCNQ1OT1		GeneCard	
2316	Potassium Voltage-Gated Channel Subfamily Q Member 3	KCNQ3		GeneCard	
2317	Potassium Voltage-Gated Channel Modifier Subfamily V Member 2	KCNV2		GeneCard	
2318	Potassium Channel Tetramerization Domain Containing 15	KCTD15			DisGenet
2319	Lysine Demethylase 1A	KDM1A		GeneCard	
2320	Lysine Demethylase 4A	KDM4A			DisGenet
2321	Lysine Demethylase 4C	KDM4C		GeneCard	
2322	Lysine Demethylase 5B	KDM5B		GeneCard	
2323	Lysine Demethylase 5D	KDM5D		GeneCard	DisGenet
2324	Lysine Demethylase 6B	KDM6B		GeneCard	DisGenet

2325	Kinase Insert Domain Receptor	KDR		GeneCard	DisGenet
2326	Kelch Like ECH Associated Protein 1	KEAP1		GeneCard	
2327	Keratocan	KERA		GeneCard	DisGenet
2328	KH RNA Binding Domain Containing, Signal Transduction Associated 1	KHDRBS1		GeneCard	DisGenet
2329	Ketohexokinase	KHK		GeneCard	
2330	KH-Type Splicing Regulatory Protein	KHSRP		GeneCard	
2331	KIAA0319 Like	KIAA0319L		GeneCard	
2332	Kinesin Family Member 11	KIF11		GeneCard	
2333	Kinesin Family Member 13B	KIF13B		GeneCard	
2334	Kinesin Family Member 20A	KIF20A	OMIM	GeneCard	
2335	Kinesin Family Member 3A	KIF3A		GeneCard	
2336	Kinesin Family Member 5A	KIF5A		GeneCard	
2337	Kinesin Family Member 6	KIF6		GeneCard	DisGenet
2338	Killer Cell Immunoglobulin Like Receptor, Two Ig Domains And Short Cytoplasmic Tail 1	KIR2DS1		GeneCard	
2339	KiSS-1 Metastasis Suppressor	KISS1		GeneCard	DisGenet
2340	KISS1 Receptor	KISS1R		GeneCard	DisGenet
2341	KIT Proto-Oncogene, Receptor Tyrosine Kinase	KIT		GeneCard	DisGenet
2342	KIT Ligand	KITLG		GeneCard	
2343	Klotho	KL	OMIM	GeneCard	DisGenet
2344	Kruppel Like Factor 10	KLF10		GeneCard	

2345	Kruppel Like Factor 14	KLF14		GeneCard	DisGenet
2346	Kruppel Like Factor 15	KLF15		GeneCard	DisGenet
2347	Kruppel Like Factor 2	KLF2		GeneCard	DisGenet
2348	Kruppel Like Factor 3	KLF3		GeneCard	
2349	Kruppel Like Factor 4	KLF4		GeneCard	DisGenet
2350	Kruppel Like Factor 5	KLF5		GeneCard	DisGenet
2351	Kruppel Like Factor 6	KLF6		GeneCard	
2352	Kelch Like Family Member 12	KLHL12		GeneCard	
2353	Kelch Like Family Member 2	KLHL2		GeneCard	
2354	Kallikrein 1	KLK1		GeneCard	DisGenet
2355	Kallikrein Related Peptidase 15	KLK15		GeneCard	
2356	Kallikrein Related Peptidase 3	KLK3		GeneCard	DisGenet
2357	Kallikrein B1	KLKB1		GeneCard	
2358	Killer Cell Lectin Like Receptor C1	KLRC1		GeneCard	
2359	Killer Cell Lectin Like Receptor C2	KLRC2		GeneCard	
2360	Killer Cell Lectin Like Receptor C4	KLRC4		GeneCard	
2361	KLRC4-KLRK1 readthrough	KLRC4-KLRK1			DisGenet
2362	Killer Cell Lectin Like Receptor D1	KLRD1		GeneCard	
2363	Killer Cell Lectin Like Receptor G1	KLRG1			DisGenet
2364	Killer Cell Lectin Like Receptor K1	KLRK1		GeneCard	DisGenet

2365	Lysine Methyltransferase 2A	KMT2A			DisGenet
2366	Lysine Methyltransferase 2B	KMT2B			DisGenet
2367	Lysine Methyltransferase 2D	KMT2D			DisGenet
2368	Lysine Methyltransferase 2E	KMT2E			DisGenet
2369	Lysine Methyltransferase 5B	KMT5B		GeneCard	
2370	Lysine Methyltransferase 5C	KMT5C		GeneCard	
2371	Kininogen 1	KNG1		GeneCard	DisGenet
2372	Kinetochore Associated 1	KNTC1		GeneCard	
2373	Karyopherin Subunit Alpha 2	KPNA2		GeneCard	
2374	Karyopherin Subunit Alpha 3	KPNA3		GeneCard	DisGenet
2375	Karyopherin Subunit Alpha 4	KPNA4		GeneCard	DisGenet
2376	KRAB Box Domain Containing 4	KRBOX4		GeneCard	
2377	Kringle Containing Transmembrane Protein 1	KREMEN1		GeneCard	
2378	KRIT1 Ankyrin Repeat Containing	KRIT1		GeneCard	DisGenet
2379	Keratin 18	KRT18		GeneCard	
2380	Keratin 20	KRT20			DisGenet
2381	Keratin 74	KRT74		GeneCard	
2382	Keratin 8	KRT8		GeneCard	
2383	Keratin Associated Protein 11-1	KRTAP11-1		GeneCard	
2384	Lactamase Beta	LACTB		GeneCard	

2385	Ladinin 1	LAD1			DisGenet
2386	Lymphocyte Activating 3	LAG3		GeneCard	DisGenet
2387	Leukocyte associated immunoglobulin like receptor 1	LAIR1			DisGenet
2388	Laminin Subunit Alpha 2	LAMA2		GeneCard	
2389	Laminin Subunit Alpha 3	LAMA3		GeneCard	
2390	Laminin Subunit Alpha 4	LAMA4		GeneCard	
2391	Laminin Subunit Gamma 1	LAMC1		GeneCard	
2392	Lysosomal Associated Membrane Protein 2	LAMP2		GeneCard	
2393	LanC Like 1	LANCL1			DisGenet
2394	LARGE Xylosyl- And Glucuronyltransferase 1	LARGE1		GeneCard	
2395	LARP1B Pseudogene 2	LARP1BP2			DisGenet
2396	La Ribonucleoprotein 6, Translational Regulator	LARP6		GeneCard	
2397	La Ribonucleoprotein 7, Transcriptional Regulator	LARP7		GeneCard	
2398	Leucyl-TRNA Synthetase 1	LARS1		GeneCard	
2399	Leucyl-TRNA Synthetase 2, Mitochondrial	LARS2		GeneCard	
2400	LIM And SH3 Protein 1	LASP1		GeneCard	
2401	Lipopolysaccharide Binding Protein	LBP		GeneCard	DisGenet
2402	Lamin B Receptor	LBR		GeneCard	
2403	Ladybird Homeobox 1	LBX1		GeneCard	
2404	Lecithin-Cholesterol Acyltransferase	LCAT		GeneCard	DisGenet

2405	LCK Proto-Oncogene, Src Family Tyrosine Kinase	LCK		GeneCard	
2406	Lipocalin 1	LCN1		GeneCard	
2407	Lipocalin 2	LCN2		GeneCard	DisGenet
2408	Ligand Dependent Nuclear Receptor Corepressor	LCOR			DisGenet
2409	Lactase (lactase-phlorizin hydrolase)	LCT	OMIM	GeneCard	
2410	Lipid Droplet Associated Hydrolase	LDAH		GeneCard	
2411	LIM Domain Binding 2	LDB2		GeneCard	DisGenet
2412	LIM Domain Binding 3	LDB3		GeneCard	
2413	Lactate Dehydrogenase A	LDHA		GeneCard	DisGenet
2414	Lactate Dehydrogenase C	LDHC		GeneCard	
2415	Low-Density Lipoprotein Receptor	LDLR		GeneCard	DisGenet
2416	Low-density lipoprotein receptor adaptor protein 1	LDLRAP1	OMIM	GeneCard	DisGenet
2417	LDLR Antisense RNA 1	LDLR-AS1		GeneCard	
2418	Leukocyte Cell Derived Chemotaxin 2	LECT2		GeneCard	DisGenet
2419	Lymphoid Enhancer Binding Factor 1	LEF1		GeneCard	
2420	LEF1 Antisense RNA 1	LEF1-AS1		GeneCard	DisGenet
2421	Leucine, Glutamate and Lysine Rich 1	LEKR1		GeneCard	
2422	Late Cornified Envelope Like Proline Rich 1	LELP1		GeneCard	
2423	LEM Domain Nuclear Envelope Protein 2	LEMD2	OMIM	GeneCard	
2424	Leptin (murine obesity homolog)	LEP	OMIM	GeneCard	DisGenet

2425	Leptin Serum Levels	LEPQTL1		GeneCard	DisGenet
2426	Leptin Receptor	LEPR		GeneCard	DisGenet
2427	Leptin Receptor Overlapping Transcript	LEPROT		GeneCard	
2428	Galectin 1	LGALS1		GeneCard	
2429	Galectin 12	LGALS12		GeneCard	
2430	Galectin 14	LGALS14			DisGenet
2431	Galectin 2	LGALS2		GeneCard	
2432	Galectin 3	LGALS3		GeneCard	
2433	Galectin 3 Binding Protein	LGALS3BP		GeneCard	DisGenet
2434	Galectin 8	LGALS8		GeneCard	
2435	Galectin 9	LGALS9		GeneCard	
2436	Legumain	LGMN		GeneCard	DisGenet
2437	Luteinizing Hormone/Choriogonadotropin Receptor	LHCGR		GeneCard	
2438	LHFPL Tetraspan Subfamily Member 1	LHFPL1		GeneCard	
2439	LIM Homeobox 8	LHX8		GeneCard	
2440	Lipoic Acid Synthetase	LIAS		GeneCard	
2441	LIF Interleukin 6 Family Cytokine	LIF		GeneCard	DisGenet
2442	LIF Receptor Subunit Alpha	LIFR		GeneCard	
2443	DNA Ligase 4	LIG4		GeneCard	
2444	Leukocyte Immunoglobulin Like Receptor B1	LILRB1		GeneCard	DisGenet

2445	Leukocyte Immunoglobulin Like Receptor B3	LILRB3		GeneCard	
2446	Leukocyte Immunoglobulin Like Receptor B4	LILRB4			DisGenet
2447	LIM Domain Kinase 1	LIMK1		GeneCard	
2448	LIM Zinc Finger Domain Containing 1	LIMS1		GeneCard	
2449	LIM Zinc Finger Domain Containing 2	LIMS2		GeneCard	
2450	Lin-28 Homolog A	LIN28A		GeneCard	
2451	Lin-28 Homolog B	LIN28B		GeneCard	
2452	Lin-9 DREAM MuvB Core Complex Component	LIN9		GeneCard	
2453	Long Intergenic Non-Protein Coding RNA 113	LINC00113		GeneCard	
2454	Long Intergenic Non-Protein Coding RNA 294	LINC00294		GeneCard	
2455	Long Intergenic Non-Protein Coding RNA 299	LINC00299		GeneCard	DisGenet
2456	Long Intergenic Non-Protein Coding RNA 305	LINC00305		GeneCard	DisGenet
2457	Long Intergenic Non-Protein Coding RNA 400	LINC00400		GeneCard	
2458	Long Intergenic Non-Protein Coding RNA 540	LINC00540		GeneCard	
2459	Long Intergenic Non-Protein Coding RNA 599	LINC00599			DisGenet
2460	Long Intergenic Non-Protein Coding RNA 907	LINC00907		GeneCard	
2461	Long Intergenic Non-Protein Coding RNA 1005	LINC01005		GeneCard	
2462	Long Intergenic Non-Protein Coding RNA 1123	LINC01123		GeneCard	
2463	Long Intergenic Non-Protein Coding RNA 1179	LINC01179		GeneCard	
2464	Long Intergenic Non-Protein Coding RNA 1194	LINC01194			DisGenet

2465	Long Intergenic Non-Protein Coding RNA 1228	LINC01228		GeneCard	
2466	Long Intergenic Non-Protein Coding RNA 1389	LINC01389		GeneCard	
2467	Long Intergenic Non-Protein Coding RNA 1512	LINC01512		GeneCard	
2468	Long Intergenic Non-Protein Coding RNA 1535	LINC01535		GeneCard	
2469	Long Intergenic Non-Protein Coding RNA 1672	LINC01672			DisGenet
2470	Long Intergenic Non-Protein Coding RNA 1826	LINC01826		GeneCard	
2471	Long Intergenic Non-Protein Coding RNA 1912	LINC01912		GeneCard	
2472	Long Intergenic Non-Protein Coding RNA 2370	LINC02370		GeneCard	
2473	Long Intergenic Non-Protein Coding RNA 2398	LINC02398		GeneCard	
2474	Long Intergenic Non-Protein Coding RNA 2577	LINC02577		GeneCard	
2475	Long Intergenic Non-Protein Coding RNA 2605	LINC02605			DisGenet
2476	Long Intergenic Non-Protein Coding RNA 2618	LINC02618		GeneCard	
2477	Long Intergenic Non-Protein Coding RNA 2692	LINC02692		GeneCard	
2478	Long Intergenic Non-Protein Coding RNA 2915	LINC02915		GeneCard	
2479	Long Intergenic Non-Protein Coding RNA 2962	LINC02962		GeneCard	
2480	Long Intergenic Non-Protein Coding RNA 3020	LINC03020		GeneCard	
2481	Long Intergenic Non-Protein Coding RNA 3040	LINC03040		GeneCard	
2482	Long Intergenic Non-Protein Coding RNA, Regulator Of Reprogramming	LINC-ROR			DisGenet
2483	Lipase A, lysosomal acid, cholesterol esterase	LIPA	OMIM	GeneCard	DisGenet
2484	Lipase C, Hepatic Type	LIPC		GeneCard	DisGenet

2485	Lipase E, Hormone Sensitive Type	LIPE		GeneCard	DisGenet
2486	Lipase F, Gastric Type	LIPF		GeneCard	
2487	Lipase G, Endothelial Type	LIPG		GeneCard	DisGenet
2488	Lipase H	LIPH		GeneCard	
2489	Lipase I	LIPI		GeneCard	
2490	Lipase Family Member J	LIPJ		GeneCard	
2491	Lipase Family Member K	LIPK		GeneCard	
2492	Lipase Family Member M	LIPM		GeneCard	
2493	Lipopolysaccharide Induced TNF Factor	LITAF		GeneCard	
2494	Lectin, Mannose Binding 1	LMAN1		GeneCard	
2495	Lectin, Mannose Binding 2	LMAN2		GeneCard	
2496	LIM And Cysteine Rich Domains 1	LMCD1		GeneCard	
2497	Lipase Maturation Factor 1	LMF1		GeneCard	
2498	Lipase Maturation Factor 2	LMF2		GeneCard	
2499	Lamin A/C	LMNA	OMIM	GeneCard	DisGenet
2500	Lamin B1	LMNB1		GeneCard	DisGenet
2501	LIM Domain 7	LMO7		GeneCard	
2502	Leiomodin 1	LMOD1		GeneCard	DisGenet
2503	Leiomodin 2	LMOD2		GeneCard	
2504	LIM Homeobox Transcription Factor 1 Beta	LMX1B		GeneCard	

2505	LncRNA Regulator of Akt Signaling Associated With HCC And RCC	LNCARSR		GeneCard	
2506	LncRNA Neighboring Enhancer of FOXA2	LNCNEF		GeneCard	
2507	LncRNA Activated By TGF-Beta	LNCRNA-ATB		GeneCard	DisGenet
2508	Lunapark, ER Junction Formation Factor	LNPK		GeneCard	
2509	Uracil DNA Glycosylase Pseudogene	LOC100130177		GeneCard	
2510	Uncharacterized LOC100287329	LOC100287329		GeneCard	
2511	Uncharacterized LOC100506472	LOC100506472		GeneCard	
2512	Uncharacterized LOC100507053	LOC100507053		GeneCard	
2513	Uncharacterized LOC100507346	LOC100507346		GeneCard	
2514	Uncharacterized LOC101927055	LOC101927055		GeneCard	
2515	Inactive Glutathione Hydrolase 2	LOC102724197			DisGenet
2516	Uncharacterized LOC102724465	LOC102724465		GeneCard	
2517	Uncharacterized LOC102724802	LOC102724802		GeneCard	
2518	Uncharacterized LOC105377143	LOC105377143		GeneCard	
2519	APOB 5' Regulatory Region	LOC106560211		GeneCard	
2520	GBA Recombination Region	LOC106627981		GeneCard	
2521	Adiponectin Enhancer Region	LOC106660625		GeneCard	
2522	MPO Proximal Enhancer and Promoter Region	LOC106694315		GeneCard	
2523	LEP 5' Regulatory Region	LOC106728418		GeneCard	
2524	Tenascin XB Recombination Region	LOC106780803		GeneCard	

2525	MANTIS	LOC107985770		GeneCard	DisGenet
2526	Enhancer-Blocking Element 11-1-2 Overlapping APOA5	LOC108491825		GeneCard	
2527	FGF1 Promoter B	LOC109113859		GeneCard	
2528	FGF1 Promoter C	LOC109113860		GeneCard	
2529	FGF1 Promoter D	LOC109113862		GeneCard	
2530	CYP19A1 Promoter I.4	LOC110386948		GeneCard	
2531	CYP19A1 Promoter I.7	LOC110386949		GeneCard	
2532	CYP7A1 5' Regulatory Region	LOC110596866		GeneCard	
2533	CYP11B2 Promoter	LOC110673971		GeneCard	
2534	TERT 5' Regulatory Region	LOC110806263		GeneCard	
2535	NOS3 5' Regulatory Region	LOC110973015		GeneCard	
2536	CSF1R Promoter/Intronic Regulatory Region	LOC111188156		GeneCard	
2537	NOS1 1f and 1g Alternate Promoter Region	LOC111258525		GeneCard	
2538	NOS2 5' Regulatory Region	LOC111365141		GeneCard	
2539	Sharpr-MPRA Regulatory Region 9539	LOC113939944		GeneCard	
2540	PPARG EExon Liver Enhancer	LOC114803475		GeneCard	
2541	CTSL Promoter Region	LOC117600004		GeneCard	
2542	Small Nucleolar RNA U13	LOC124903267		GeneCard	
2543	Synaptotagmin Binding Cytoplasmic RNA Interacting Protein Pseudogene	LOC149844		GeneCard	
2544	Uncharacterized LOC157273	LOC157273		GeneCard	

2545	HLA Complex Group 26 (Non-Protein Coding) Pseudogene	LOC353007		GeneCard	
2546	EBP Like Pseudogene	LOC729217		GeneCard	
2547	Loss of Heterozygosity, 19, Chromosomal Region 1	LOH19CR1			DisGenet
2548	Lysyl Oxidase	LOX		GeneCard	DisGenet
2549	Lysyl Oxidase Like 1	LOXL1		GeneCard	
2550	LOXL1 Antisense RNA 1	LOXL1-AS1		GeneCard	
2551	Apolipoprotein Lp(a)	LPA	OMIM	GeneCard	DisGenet
2552	Lipoprotein(A) Like 1	LPAL1		GeneCard	
2553	Lipoprotein(A) Like 2, Pseudogene	LPAL2		GeneCard	DisGenet
2554	Lysophosphatidic Acid Receptor 1	LPAR1		GeneCard	DisGenet
2555	Lysophosphatidic Acid Receptor 2	LPAR2		GeneCard	DisGenet
2556	Lysophosphatidic Acid Receptor 3	LPAR3		GeneCard	
2557	Lysophosphatidic Acid Receptor 4	LPAR4			DisGenet
2558	Lysophosphatidic Acid Receptor 5	LPAR5		GeneCard	
2559	Lysophosphatidylcholine Acyltransferase 3	LPCAT3		GeneCard	DisGenet
2560	Lysophosphatidylglycerol Acyltransferase 1	LPGAT1		GeneCard	
2561	Lipin 1	LPIN1	OMIM	GeneCard	
2562	Lipin 2	LPIN2		GeneCard	
2563	Lipin 3	LPIN3		GeneCard	
2564	Lipoprotein lipase	LPL	OMIM	GeneCard	DisGenet

256<br>5	Lactoperoxidase	LPO		GeneCard	
256<br>6	LIM Domain Containing Preferred Translocation Partner In Lipoma	LPP		GeneCard	DisGenet
256<br>7	Leupaxin	LPXN		GeneCard	
256<br>8	Leucine Rich Alpha-2-Glycoprotein 1	LRG1		GeneCard	
256<br>9	Leucine Rich Repeats and Guanylate Kinase Domain Containing	LRGUK		GeneCard	
257<br>0	Leucine Rich Repeats and Immunoglobulin Like Domains 1	LRIG1		GeneCard	
257<br>1	Leucine Rich Repeats and Immunoglobulin Like Domains 3	LRIG3		GeneCard	
257<br>2	Low density lipoprotein-related protein-1 (alpha-2-macroglobulin receptor)	LRP1	OMIM	GeneCard	DisGenet
257<br>3	LDL Receptor Related Protein 12	LRP12		GeneCard	
257<br>4	LDL Receptor Related Protein 1B	LRP1B		GeneCard	
257<br>5	LDL Receptor Related Protein 2	LRP2		GeneCard	DisGenet
257<br>6	LRP2 Binding Protein	LRP2BP		GeneCard	DisGenet
257<br>7	LRP2BP Antisense RNA 1	LRP2BP-AS1		GeneCard	
257<br>8	LDL Receptor Related Protein 3	LRP3		GeneCard	
257<br>9	LDL Receptor Related Protein 4	LRP4		GeneCard	
258<br>0	LDL Receptor Related Protein 5	LRP5	OMIM	GeneCard	DisGenet
258<br>1	LDL Receptor Related Protein 6	LRP6	OMIM	GeneCard	DisGenet
258<br>2	LDL Receptor Related Protein 8	LRP8	OMIM	GeneCard	DisGenet
258<br>3	LDL Receptor Related Protein Associated Protein 1	LRPAP1		GeneCard	
258<br>4	Leucine Rich Repeat Containing 10	LRRC10		GeneCard	

258<br>5	Leucine Rich Repeat Containing 18	LRRC18		GeneCard	
258<br>6	Leucine Rich Repeat Containing 2	LRRC2		GeneCard	
258<br>7	Leucine Rich Repeat Containing 32	LRRC32			DisGenet
258<br>8	Leucine Rich Repeat Containing 41	LRRC41		GeneCard	
258<br>9	Leucine Rich Repeat Containing 8 VRAC Subunit A	LRRC8A		GeneCard	
259<br>0	Leucine Rich Repeats And IQ Motif Containing 3	LRRIQ3		GeneCard	
259<br>1	Leucine Rich Repeat Kinase 2	LRRK2		GeneCard	
259<br>2	Leucine Rich Repeat Transmembrane Neuronal 1	LRRTM1		GeneCard	
259<br>3	Leucine Rich Repeat Transmembrane Neuronal 4	LRRTM4		GeneCard	
259<br>4	Limbic System Associated Membrane Protein	LSAMP		GeneCard	DisGenet
259<br>5	Lanosterol Synthase	LSS		GeneCard	
259<br>6	Lymphotoxin alpha (formerly tumor necrosis factor beta)	LTA	OMIM	GeneCard	DisGenet
259<br>7	Leukotriene A4 hydrolase	LTA4H	OMIM	GeneCard	DisGenet
259<br>8	Lymphotoxin Beta	LTB		GeneCard	DisGenet
259<br>9	Leukotriene B4 Receptor	LTB4R		GeneCard	DisGenet
260<br>0	Leukotriene B4 Receptor 2	LTB4R2		GeneCard	DisGenet
260<br>1	Latent Transforming Growth Factor Beta Binding Protein 1	LTBP1	OMIM	GeneCard	
260<br>2	Latent Transforming Growth Factor Beta Binding Protein 2	LTBP2		GeneCard	
260<br>3	Latent Transforming Growth Factor Beta Binding Protein 3	LTBP3		GeneCard	
260<br>4	Latent Transforming Growth Factor Beta Binding Protein 4	LTBP4		GeneCard	

2605	Lymphotoxin Beta Receptor	LTBR		GeneCard	
2606	Leukotriene C4 Synthase	LTC4S		GeneCard	
2607	Lactotransferrin	LTF		GeneCard	
2608	Leukocyte Receptor Tyrosine Kinase	LTK		GeneCard	
2609	Lumican	LUM		GeneCard	DisGenet
2610	Lymphocyte Antigen 75	LY75		GeneCard	
2611	Lymphocyte Antigen 86	LY86		GeneCard	
2612	Lymphocyte Antigen 9	LY9		GeneCard	
2613	Lymphocyte Antigen 96	LY96		GeneCard	
2614	LYN Proto-Oncogene, Src Family Tyrosine Kinase	LYN		GeneCard	
2615	Lysophospholipase I	LYPLA1	OMIM		DisGenet
2616	Lysophospholipase 2	LYPLA2		GeneCard	
2617	LysM Domain Containing 4	LYSMD4		GeneCard	
2618	Lysosomal Trafficking Regulator	LYST			DisGenet
2619	Lymphatic Vessel Endothelial Hyaluronan Receptor 1	LYVE1		GeneCard	
2620	Lysozyme	LYZ		GeneCard	DisGenet
2621	Mannose-6-Phosphate Receptor, Cation Dependent	M6PR		GeneCard	
2622	Microphthalmia or Anophthalmia and Associated Anomalies	MAA			DisGenet
2623	MAB-21 Like 2	MAB21L2	OMIM	GeneCard	
2624	Mono-ADP Ribosylhydrolase 2	MACROD2		GeneCard	

2625	MAF BZIP Transcription Factor	MAF		GeneCard	
2626	MAF BZIP Transcription Factor B	MAFB		GeneCard	DisGenet
2627	MAF BZIP Transcription Factor F	MAFF		GeneCard	
2628	MAF Transcriptional Regulator RNA	MAFTRR		GeneCard	
2629	MAGE family member C3	MAGEC3			DisGenet
2630	MAGE family member E2	MAGEE2			DisGenet
2631	Membrane Associated Guanylate Kinase, WW And PDZ Domain Containing 1	MAGI1		GeneCard	DisGenet
2632	Membrane Associated Guanylate Kinase, WW And PDZ Domain Containing 2	MAGI2		GeneCard	DisGenet
2633	Mago Homolog B, Exon Junction Complex Subunit	MAGOHB		GeneCard	
2634	MAK16 Homolog	MAK16			DisGenet
2635	Mal, T Cell Differentiation Protein 2	MAL2		GeneCard	
2636	Metastasis Associated Lung Adenocarcinoma Transcript 1	MALAT1		GeneCard	DisGenet
2637	MAM And LDL Receptor Class A Domain Containing 1	MALRD1		GeneCard	
2638	MALT1 Paracaspase	MALT1		GeneCard	
2639	Mastermind Like Transcriptional Coactivator 1	MAML1			DisGenet
2640	Mannosidase Alpha Class 2A Member 1	MAN2A1		GeneCard	
2641	Mannosidase Alpha Class 2B Member 1	MAN2B1		GeneCard	
2642	Mesencephalic Astrocyte Derived Neurotrophic Factor	MANF		GeneCard	
2643	Monoamine Oxidase A	MAOA		GeneCard	DisGenet
2644	Monoamine Oxidase B	MAOB		GeneCard	

2645	Microtubule Associated Protein 1 Light Chain 3 Alpha	MAP1LC3A		GeneCard	
2646	Microtubule Associated Protein 1 Light Chain 3 Beta	MAP1LC3B		GeneCard	
2647	Microtubule Associated Protein 2	MAP2		GeneCard	
2648	Mitogen-Activated Protein Kinase Kinase 1	MAP2K1		GeneCard	DisGenet
2649	Mitogen-Activated Protein Kinase Kinase 3	MAP2K3		GeneCard	
2650	Mitogen-Activated Protein Kinase Kinase 4	MAP2K4		GeneCard	
2651	Mitogen-Activated Protein Kinase Kinase 5	MAP2K5		GeneCard	
2652	Mitogen-Activated Protein Kinase Kinase 6	MAP2K6		GeneCard	
2653	Mitogen-Activated Protein Kinase Kinase 7	MAP2K7		GeneCard	DisGenet
2654	Mitogen-Activated Protein Kinase Kinase Kinase 1	MAP3K1		GeneCard	
2655	Mitogen-Activated Protein Kinase Kinase Kinase 10	MAP3K10			DisGenet
2656	Mitogen-Activated Protein Kinase Kinase Kinase 14	MAP3K14		GeneCard	
2657	Mitogen-Activated Protein Kinase Kinase Kinase 2	MAP3K2		GeneCard	
2658	Mitogen-Activated Protein Kinase Kinase Kinase 21	MAP3K21		GeneCard	
2659	Mitogen-Activated Protein Kinase Kinase Kinase 3	MAP3K3		GeneCard	
2660	Mitogen-Activated Protein Kinase Kinase Kinase 5	MAP3K5		GeneCard	DisGenet
2661	Mitogen-Activated Protein Kinase Kinase Kinase 7	MAP3K7		GeneCard	DisGenet
2662	Mitogen-Activated Protein Kinase Kinase Kinase Kinase 4	MAP4K4		GeneCard	DisGenet
2663	Mitogen-Activated Protein Kinase 1	MAPK1		GeneCard	DisGenet
2664	Mitogen-Activated Protein Kinase 10	MAPK10		GeneCard	

2665	Mitogen-Activated Protein Kinase 12	MAPK12		GeneCard	
2666	Mitogen-Activated Protein Kinase 13	MAPK13		GeneCard	
2667	Mitogen-Activated Protein Kinase 14	MAPK14		GeneCard	DisGenet
2668	Mitogen-Activated Protein Kinase 3	MAPK3		GeneCard	DisGenet
2669	Mitogen-Activated Protein Kinase 7	MAPK7		GeneCard	DisGenet
2670	Mitogen-Activated Protein Kinase 8	MAPK8		GeneCard	DisGenet
2671	Mitogen-activated protein kinase 9	MAPK9	OMIM	GeneCard	DisGenet
2672	MAPK Activated Protein Kinase 2	MAPKAPK2		GeneCard	
2673	Microtubule Associated Protein Tau	MAPT		GeneCard	
2674	Membrane Associated Ring-CH-type Finger 10	MARCHF10			DisGenet
2675	Myristoylated Alanine Rich Protein Kinase C Substrate	MARCKS		GeneCard	
2676	MARCKS Like 1	MARCKSL1		GeneCard	
2677	Macrophage Receptor With Collagenous Structure	MARCO		GeneCard	
2678	Microtubule Affinity Regulating Kinase 1	MARK1		GeneCard	
2679	Microtubule Affinity Regulating Kinase 2	MARK2			DisGenet
2680	Microtubule Affinity Regulating Kinase 4	MARK4		GeneCard	
2681	Methionyl-TRNA Synthetase 1	MARS1		GeneCard	
2682	MAS1 roto-oncogene, G protein-coupled receptor	MAS1			DisGenet
2683	MALAT1-associated small cytoplasmic RNA	MASCRNA			DisGenet
2684	MBL Associated Serine Protease 1	MASP1		GeneCard	

2685	MBL Associated Serine Protease 2	MASP2		GeneCard	
2686	Microtubule Associated Serine/Threonine Kinase Family Member 4	MAST4		GeneCard	
2687	Methionine Adenosyltransferase 2A	MAT2A		GeneCard	
2688	Methionine Adenosyltransferase 2B	MAT2B		GeneCard	
2689	Mitochondrial Antiviral Signaling Protein	MAVS		GeneCard	
2690	MYC Associated Zinc Finger Protein	MAZ		GeneCard	DisGenet
2691	Myoglobin	MB		GeneCard	
2692	Methyl-CpG Binding Domain Protein 2	MBD2		GeneCard	
2693	Methyl-CpG Binding Domain Protein 3	MBD3		GeneCard	
2694	Mannose Binding Lectin 1, Pseudogene	MBL1P		GeneCard	
2695	Mannose Binding Lectin 2	MBL2	OMIM	GeneCard	DisGenet
2696	Mannose-Binding Lectin Family Member 3, Pseudogene	MBL3P			DisGenet
2697	Muscleblind Like Splicing Regulator 1	MBNL1		GeneCard	
2698	Membrane Bound O-Acyltransferase Domain Containing 1	MBOAT1		GeneCard	
2699	Membrane Bound Transcription Factor Peptidase, Site 1	MBTPS1		GeneCard	DisGenet
2700	Membrane Bound Transcription Factor Peptidase, Site 2	MBTPS2		GeneCard	
2701	Melanocortin 1 Receptor	MC1R			DisGenet
2702	Melanocortin 4 Receptor	MC4R		GeneCard	DisGenet
2703	Melanoma Cell Adhesion Molecule	MCAM		GeneCard	DisGenet
2704	Malonyl-CoA-Acyl Carrier Protein Transacylase	MCAT		GeneCard	

2705	MCF.2 Cell Line Derived Transforming Sequence Like	MCF2L		GeneCard	DisGenet
2706	Myocardial Infarction, Susceptiblity To, 2	MCI2		GeneCard	
2707	MCL1 Apoptosis Regulator, BCL2 Family Member	MCL1		GeneCard	DisGenet
2708	Minichromosome Maintenance Complex Component 3	MCM3			DisGenet
2709	Minichromosome Maintenance 8 Homologous Recombination Repair Factor	MCM8		GeneCard	
2710	Minichromosome Maintenance 9 Homologous Recombination Repair Factor	MCM9		GeneCard	
2711	Microcephalin 1	MCPH1		GeneCard	
2712	Multiple C2 And Transmembrane Domain Containing 2	MCTP2		GeneCard	
2713	Midkine	MDK		GeneCard	DisGenet
2714	MDM2 Proto-Oncogene	MDM2		GeneCard	DisGenet
2715	MDS1 And EVI1 Complex Locus	MECOM		GeneCard	
2716	Methyl-CpG Binding Protein 2	MECP2		GeneCard	DisGenet
2717	Mediator Complex Subunit 1	MED1		GeneCard	DisGenet
2718	Mediator Complex Subunit 17	MED17		GeneCard	
2719	Mediator Complex Subunit 28	MED28		GeneCard	
2720	Mediator Complex Subunit 30	MED30		GeneCard	
2721	Mediator Complex Subunit 6	MED6		GeneCard	
2722	Myocyte Enhancer Factor 2A	MEF2A		GeneCard	DisGenet
2723	Myocyte Enhancer Factor 2C	MEF2C		GeneCard	DisGenet
2724	MEFV Innate Immunity Regulator, Pyrin	MEFV		GeneCard	DisGenet

2725	Maternally Expressed 3	MEG3		GeneCard	DisGenet
2726	Maternally Expressed 8, Small Nucleolar RNA Host Gene	MEG8		GeneCard	
2727	Multiple EGF Like Domains 10	MEGF10		GeneCard	
2728	Meiosis Specific With OB-Fold	MEIOB		GeneCard	
2729	Mesenchyme Homeobox 2	MEOX2		GeneCard	
2730	Meprin A Subunit Beta	MEP1B		GeneCard	
2731	MER Proto-Oncogene, Tyrosine Kinase	MERTK		GeneCard	DisGenet
2732	Mesoderm Development LRP Chaperone	MESD		GeneCard	
2733	MET Proto-Oncogene, Receptor Tyrosine Kinase	MET		GeneCard	
2734	Meteorin, Glial Cell Differentiation Regulator	METRN			DisGenet
2735	Meteorin Like, Glial Cell Differentiation Regulator	METRNL		GeneCard	
2736	Methyltransferase 14, N6-Adenosine-Methyltransferase Subunit	METTL14		GeneCard	
2737	Methyltransferase 3, N6-Adenosine-Methyltransferase Complex Catalytic Subunit	METTL3		GeneCard	
2738	Methyltransferase Like 9	METTL9		GeneCard	
2739	Microfibril Associated Protein 1	MFAP1			DisGenet
2740	Microfibril Associated Protein 2	MFAP2		GeneCard	
2741	Microfibril Associated Protein 4	MFAP4		GeneCard	
2742	Microfibril Associated Protein 5	MFAP5		GeneCard	
2743	Milk Fat Globule EGF And Factor V/VIII Domain Containing	MFGE8		GeneCard	DisGenet
2744	Mitofusin 1	MFN1		GeneCard	

274<br>5	Mitofusin 2	MFN2		GeneCard	DisGenet
274<br>6	Membrane Frizzled-Related Protein	MFRP			DisGenet
274<br>7	Major Facilitator Superfamily Domain Containing 11	MFSD11		GeneCard	
274<br>8	Major Facilitator Superfamily Domain Containing 8	MFSD8		GeneCard	
274<br>9	Alpha-1,6-Mannosylglycoprotein 6-Beta-N-Acetylglucosaminyltransferase B	MGAT5B		GeneCard	
275<br>0	Monoglyceride Lipase	MGLL		GeneCard	DisGenet
275<br>1	O-6-Methylguanine-DNA Methyltransferase	MGMT		GeneCard	
275<br>2	Matrix Gla protein	MGP	OMIM	GeneCard	DisGenet
275<br>3	Myosin Heavy Chain Associated RNA Transcript	MHRT		GeneCard	
275<br>4	MIA SH3 Domain ER Export Factor 3	MIA3		GeneCard	DisGenet
275<br>5	Myocardial Infarction Associated Transcript	MIAT		GeneCard	DisGenet
275<br>6	MHC Class I Polypeptide-Related Sequence A	MICA		GeneCard	DisGenet
275<br>7	MICA Antisense RNA 1	MICA-AS1		GeneCard	
275<br>8	MHC Class I Polypeptide-Related Sequence B	MICB		GeneCard	
275<br>9	Mitochondrial Contact Site and Cristae Organizing System Subunit 10	MICOS10		GeneCard	
276<br>0	MICOS10-NBL1 readthrough	MICOS10-NBL1			DisGenet
276<br>1	Mitochondrial Calcium Uptake 1	MICU1			DisGenet
276<br>2	Macrophage Migration Inhibitory Factor	MIF		GeneCard	DisGenet
276<br>3	MINDY lysine 48 deubiquitinase 4	MINDY4			DisGenet
276<br>4	Mitochondrial Intermediate Peptidase	MIPEP		GeneCard	

2765	MicroRNA 100	MIR100		GeneCard	DisGenet
2766	MicroRNA 101-1	MIR101-1		GeneCard	
2767	MicroRNA 103a-1	MIR103A1		GeneCard	
2768	MicroRNA 103a-2	MIR103A2		GeneCard	
2769	MicroRNA 106a	MIR106A		GeneCard	
2770	MicroRNA 106b	MIR106B		GeneCard	
2771	MicroRNA 107	MIR107			DisGenet
2772	MicroRNA 10a	MIR10A		GeneCard	DisGenet
2773	MicroRNA 10b	MIR10B		GeneCard	DisGenet
2774	MicroRNA 11399	MIR11399		GeneCard	
2775	MicroRNA 11400	MIR11400		GeneCard	
2776	MicroRNA 11401	MIR11401		GeneCard	
2777	MicroRNA 1185-1	MIR1185-1		GeneCard	
2778	MicroRNA 1185-2	MIR1185-2		GeneCard	
2779	MicroRNA 1208	MIR1208		GeneCard	
2780	MicroRNA 122	MIR122		GeneCard	DisGenet
2781	MicroRNA 1228	MIR1228		GeneCard	
2782	MicroRNA 124-1	MIR124-1		GeneCard	
2783	MIR124-1 Host Gene	MIR124-1HG		GeneCard	
2784	MicroRNA 124-2	MIR124-2		GeneCard	

2785	MicroRNA 124-3	MIR124-3		GeneCard	
2786	MicroRNA 1246	MIR1246		GeneCard	
2787	MicroRNA 125a	MIR125A		GeneCard	DisGenet
2788	MicroRNA 125b-1	MIR125B1	OMIM	GeneCard	
2789	MicroRNA 126	MIR126		GeneCard	DisGenet
2790	MicroRNA 1264	MIR1264		GeneCard	
2791	MicroRNA 127	MIR127			DisGenet
2792	MicroRNA 1275	MIR1275		GeneCard	
2793	MicroRNA 1277	MIR1277		GeneCard	
2794	MicroRNA 128-1	MIR128-1		GeneCard	
2795	MicroRNA 1290	MIR1290		GeneCard	
2796	MicroRNA 1306	MIR1306		GeneCard	
2797	MicroRNA 130a	MIR130A		GeneCard	
2798	MicroRNA 130b	MIR130B		GeneCard	
2799	MicroRNA 132	MIR132		GeneCard	
2800	MicroRNA 133a-1	MIR133A1		GeneCard	
2801	MicroRNA 133a-2	MIR133A2		GeneCard	
2802	MicroRNA 133b	MIR133B		GeneCard	DisGenet
2803	MicroRNA 134	MIR134		GeneCard	DisGenet
2804	MicroRNA 135a-1	MIR135A1		GeneCard	

280<br>5	MicroRNA 135a-2	MIR135A2		GeneCard	
280<br>6	MicroRNA 135b	MIR135B		GeneCard	DisGenet
280<br>7	MicroRNA 136	MIR136			DisGenet
280<br>8	MicroRNA 137	MIR137		GeneCard	
280<br>9	MicroRNA 140	MIR140		GeneCard	DisGenet
281<br>0	MicroRNA 141	MIR141		GeneCard	
281<br>1	MicroRNA 142	MIR142		GeneCard	DisGenet
281<br>2	MicroRNA 143	MIR143	OMIM	GeneCard	DisGenet
281<br>3	MicroRNA 144	MIR144		GeneCard	DisGenet
281<br>4	MicroRNA 145	MIR145	OMIM	GeneCard	DisGenet
281<br>5	MicroRNA 146a	MIR146A		GeneCard	DisGenet
281<br>6	MicroRNA 146b	MIR146B		GeneCard	DisGenet
281<br>7	MicroRNA 147a	MIR147A		GeneCard	
281<br>8	MicroRNA 148a	MIR148A		GeneCard	DisGenet
281<br>9	MicroRNA 148b	MIR148B		GeneCard	DisGenet
282<br>0	MicroRNA 149	MIR149		GeneCard	DisGenet
282<br>1	MicroRNA 150	MIR150		GeneCard	DisGenet
282<br>2	MicroRNA 151a	MIR151A		GeneCard	
282<br>3	MicroRNA 152	MIR152		GeneCard	DisGenet
282<br>4	MicroRNA 155	MIR155		GeneCard	DisGenet

282<br>5	MicroRNA 15a	MIR15A		GeneCard	DisGenet
282<br>6	MicroRNA 15b	MIR15B		GeneCard	
282<br>7	MicroRNA 16-1	MIR16-1		GeneCard	
282<br>8	MicroRNA 17	MIR17		GeneCard	DisGenet
282<br>9	MiR-17-92a-1 cluster host gene	MIR17HG			DisGenet
283<br>0	MicroRNA 181a-1	MIR181A1		GeneCard	
283<br>1	MicroRNA 181a-2	MIR181A2			DisGenet
283<br>2	MicroRNA 181b-1	MIR181B1		GeneCard	
283<br>3	MicroRNA 181b-2	MIR181B2		GeneCard	
283<br>4	MicroRNA 181c	MIR181C		GeneCard	
283<br>5	MicroRNA 182	MIR182		GeneCard	DisGenet
283<br>6	MicroRNA 183	MIR183		GeneCard	DisGenet
283<br>7	MicroRNA 184	MIR184		GeneCard	
283<br>8	MicroRNA 185	MIR185		GeneCard	DisGenet
283<br>9	MicroRNA 186	MIR186		GeneCard	
284<br>0	MicroRNA 188	MIR188			DisGenet
284<br>1	MicroRNA 18a	MIR18A		GeneCard	DisGenet
284<br>2	MicroRNA 18b	MIR18B			DisGenet
284<br>3	MicroRNA 191	MIR191		GeneCard	
284<br>4	MicroRNA 192	MIR192		GeneCard	DisGenet

2845	MicroRNA 193b	MIR193B		GeneCard	
2846	MicroRNA 194-1	MIR194-1		GeneCard	
2847	MicroRNA 195	MIR195		GeneCard	
2848	MicroRNA 196a-2	MIR196A2		GeneCard	
2849	MicroRNA 197	MIR197		GeneCard	
2850	MicroRNA 198	MIR198		GeneCard	
2851	MicroRNA 199a-1	MIR199A1		GeneCard	DisGenet
2852	MicroRNA 199a-2	MIR199A2		GeneCard	DisGenet
2853	MicroRNA 19a	MIR19A		GeneCard	DisGenet
2854	MicroRNA 19b-1	MIR19B1		GeneCard	DisGenet
2855	MicroRNA 19b-2	MIR19B2		GeneCard	
2856	MicroRNA 200a	MIR200A		GeneCard	
2857	MicroRNA 200b	MIR200B		GeneCard	
2858	MicroRNA 200c	MIR200C		GeneCard	
2859	MicroRNA 204	MIR204		GeneCard	
2860	MicroRNA 205	MIR205		GeneCard	DisGenet
2861	MicroRNA 206	MIR206		GeneCard	DisGenet
2862	MicroRNA 208a	MIR208A		GeneCard	
2863	MicroRNA 208b	MIR208B		GeneCard	
2864	MicroRNA 20a	MIR20A		GeneCard	DisGenet

2865	MicroRNA 21	MIR21		GeneCard	DisGenet
2866	MicroRNA 210	MIR210		GeneCard	DisGenet
2867	MicroRNA 211	MIR211		GeneCard	
2868	MicroRNA 2113	MIR2113		GeneCard	
2869	MicroRNA 212	MIR212		GeneCard	DisGenet
2870	MicroRNA 214	MIR214		GeneCard	DisGenet
2871	MicroRNA 215	MIR215		GeneCard	
2872	MicroRNA 216a	MIR216A		GeneCard	DisGenet
2873	MicroRNA 217	MIR217	OMIM	GeneCard	DisGenet
2874	MicroRNA 22	MIR22		GeneCard	DisGenet
2875	MicroRNA 221	MIR221		GeneCard	DisGenet
2876	MicroRNA 222	MIR222		GeneCard	DisGenet
2877	MiR222/221 Cluster Host Gene	MIR222HG		GeneCard	DisGenet
2878	MicroRNA 223	MIR223		GeneCard	DisGenet
2879	MicroRNA 224	MIR224		GeneCard	
2880	MicroRNA 23a	MIR23A		GeneCard	DisGenet
2881	MicroRNA 23b	MIR23B		GeneCard	DisGenet
2882	MicroRNA 24-1	MIR24-1		GeneCard	DisGenet
2883	MicroRNA 24-2	MIR24-2		GeneCard	
2884	MicroRNA 25	MIR25		GeneCard	DisGenet

2885	MicroRNA 26a-1	MIR26A1		GeneCard	
2886	MicroRNA 26a-2	MIR26A2		GeneCard	
2887	MicroRNA 27a	MIR27A		GeneCard	DisGenet
2888	MicroRNA 27b	MIR27B		GeneCard	DisGenet
2889	MicroRNA 28	MIR28		GeneCard	DisGenet
2890	MicroRNA 2909	MIR2909		GeneCard	
2891	MicroRNA 296	MIR296		GeneCard	DisGenet
2892	MicroRNA 29a	MIR29A		GeneCard	DisGenet
2893	MicroRNA 29b-1	MIR29B1		GeneCard	DisGenet
2894	microRNA 29b-2	MIR29B2			DisGenet
2895	microRNA 29c	MIR29C			DisGenet
2896	MicroRNA 302a	MIR302A		GeneCard	DisGenet
2897	MicroRNA 30a	MIR30A		GeneCard	
2898	MicroRNA 30b	MIR30B		GeneCard	
2899	MicroRNA 30c-1	MIR30C1		GeneCard	DisGenet
2900	MicroRNA 30c-2	MIR30C2		GeneCard	DisGenet
2901	MicroRNA 30d	MIR30D		GeneCard	
2902	MicroRNA 30e	MIR30E		GeneCard	DisGenet
2903	MicroRNA 31	MIR31		GeneCard	
2904	MicroRNA 3188	MIR3188		GeneCard	DisGenet

290<br>5	MicroRNA 320a	MIR320A		GeneCard	
290<br>6	MicroRNA 320b-1	MIR320B1		GeneCard	
290<br>7	MicroRNA 323a	MIR323A		GeneCard	
290<br>8	MicroRNA 324	MIR324		GeneCard	
290<br>9	MicroRNA 325	MIR325		GeneCard	
291<br>0	MicroRNA 326	MIR326		GeneCard	
291<br>1	MicroRNA 328	MIR328		GeneCard	DisGenet
291<br>2	MicroRNA 330	MIR330		GeneCard	DisGenet
291<br>3	MicroRNA 331	MIR331		GeneCard	
291<br>4	MicroRNA 335	MIR335		GeneCard	DisGenet
291<br>5	MicroRNA 338	MIR338			DisGenet
291<br>6	MicroRNA 339	MIR339		GeneCard	
291<br>7	MicroRNA 33a	MIR33A		GeneCard	DisGenet
291<br>8	MicroRNA 33b	MIR33B		GeneCard	DisGenet
291<br>9	MicroRNA 340	MIR340		GeneCard	
292<br>0	microRNA 342	MIR342			DisGenet
292<br>1	MicroRNA 345	MIR345		GeneCard	
292<br>2	MicroRNA 346	MIR346		GeneCard	
292<br>3	MicroRNA 34a	MIR34A		GeneCard	DisGenet
292<br>4	MicroRNA 34b	MIR34B		GeneCard	

2925	MicroRNA 34c	MIR34C		GeneCard	
2926	MicroRNA 361	MIR361		GeneCard	
2927	MicroRNA 362	MIR362		GeneCard	DisGenet
2928	MicroRNA 365a	MIR365A		GeneCard	
2929	MicroRNA 367	MIR367		GeneCard	
2930	MicroRNA 3688-1	MIR3688-1		GeneCard	
2931	MicroRNA 370	MIR370		GeneCard	DisGenet
2932	MicroRNA 375	MIR375			DisGenet
2933	MicroRNA 377	MIR377		GeneCard	DisGenet
2934	MicroRNA 378a	MIR378A		GeneCard	DisGenet
2935	MicroRNA 378c	MIR378C		GeneCard	
2936	MicroRNA 381	MIR381		GeneCard	
2937	MicroRNA 382	MIR382		GeneCard	
2938	MicroRNA 384	MIR384		GeneCard	
2939	MicroRNA 410	MIR410			DisGenet
2940	MicroRNA 421	MIR421		GeneCard	
2941	MicroRNA 423	MIR423		GeneCard	
2942	MicroRNA 424	MIR424		GeneCard	
2943	MicroRNA 425	MIR425		GeneCard	
2944	MicroRNA 431	MIR431			DisGenet

2945	MicroRNA 433	MIR433		GeneCard	
2946	MicroRNA 4463	MIR4463		GeneCard	DisGenet
2947	MicroRNA 448	MIR448		GeneCard	
2948	MicroRNA 449a	MIR449A			DisGenet
2949	MicroRNA 451a	MIR451A		GeneCard	DisGenet
2950	MicroRNA 455	MIR455		GeneCard	DisGenet
2951	MicroRNA 4632	MIR4632		GeneCard	
2952	MIR4713 Host Gene	MIR4713HG		GeneCard	
2953	MicroRNA 4723	MIR4723		GeneCard	
2954	MicroRNA 4731	MIR4731		GeneCard	
2955	MicroRNA 483	MIR483		GeneCard	
2956	MicroRNA 484	MIR484		GeneCard	
2957	MicroRNA 486-1	MIR486-1		GeneCard	DisGenet
2958	MicroRNA 488	MIR488		GeneCard	
2959	MicroRNA 489	MIR489		GeneCard	DisGenet
2960	MicroRNA 490	MIR490		GeneCard	DisGenet
2961	MicroRNA 491	MIR491		GeneCard	
2962	MicroRNA 492	MIR492		GeneCard	DisGenet
2963	MicroRNA 494	MIR494		GeneCard	DisGenet
2964	MicroRNA 495	MIR495		GeneCard	

2965	MicroRNA 496	MIR496		GeneCard	
2966	MicroRNA 497	MIR497		GeneCard	DisGenet
2967	MicroRNA 499a	MIR499A		GeneCard	DisGenet
2968	MicroRNA 500a	MIR500A		GeneCard	
2969	MicroRNA 502	MIR502		GeneCard	
2970	MicroRNA 503	MIR503		GeneCard	
2971	MicroRNA 505	MIR505		GeneCard	DisGenet
2972	MicroRNA 506	MIR506		GeneCard	
2973	MicroRNA 508	MIR508		GeneCard	
2974	MicroRNA 512-1	MIR512-1		GeneCard	
2975	MicroRNA 518b	MIR518B		GeneCard	
2976	MicroRNA 5197	MIR5197		GeneCard	
2977	MicroRNA 519d	MIR519D			DisGenet
2978	MicroRNA 520b	MIR520B		GeneCard	
2979	MicroRNA 520c	MIR520C		GeneCard	
2980	MicroRNA 532	MIR532		GeneCard	
2981	MicroRNA 541	MIR541		GeneCard	
2982	MicroRNA 544a	MIR544A		GeneCard	
2983	MicroRNA 544b	MIR544B		GeneCard	
2984	MicroRNA 548j	MIR548J		GeneCard	

2985	MicroRNA 548p	MIR548P		GeneCard	
2986	MicroRNA 556	MIR556		GeneCard	
2987	MicroRNA 557	MIR557		GeneCard	
2988	MicroRNA 570	MIR570		GeneCard	
2989	MicroRNA 575	MIR575		GeneCard	DisGenet
2990	MicroRNA 583	MIR583		GeneCard	
2991	MicroRNA 584	MIR584		GeneCard	
2992	MicroRNA 590	MIR590		GeneCard	DisGenet
2993	MicroRNA 596	MIR596		GeneCard	
2994	MicroRNA 599	MIR599		GeneCard	
2995	MicroRNA 600	MIR600		GeneCard	
2996	MicroRNA 601	MIR601		GeneCard	
2997	MicroRNA 602	MIR602		GeneCard	
2998	MicroRNA 608	MIR608		GeneCard	
2999	MicroRNA 6089	MIR6089		GeneCard	
3000	MicroRNA 611	MIR611		GeneCard	
3001	MicroRNA 612	MIR612		GeneCard	DisGenet
3002	MicroRNA 613	MIR613		GeneCard	
3003	MicroRNA 6132	MIR6132		GeneCard	
3004	MicroRNA 615	MIR615		GeneCard	

300 5	MicroRNA 616	MIR616		GeneCard	
300 6	MicroRNA 622	MIR622		GeneCard	
300 7	MicroRNA 624	MIR624		GeneCard	
300 8	MicroRNA 629	MIR629		GeneCard	
300 9	MicroRNA 630	MIR630		GeneCard	
301 0	MicroRNA 636	MIR636		GeneCard	
301 1	MicroRNA 637	MIR637		GeneCard	
301 2	MicroRNA 638	MIR638		GeneCard	
301 3	MicroRNA 642a	MIR642A		GeneCard	
301 4	MicroRNA 647	MIR647		GeneCard	DisGenet
301 5	MicroRNA 652	MIR652			DisGenet
301 6	MicroRNA 654	MIR654		GeneCard	
301 7	MicroRNA 657	MIR657		GeneCard	
301 8	MicroRNA 658	MIR658		GeneCard	
301 9	MicroRNA 659	MIR659		GeneCard	
302 0	MicroRNA 662	MIR662		GeneCard	
302 1	MicroRNA 663a	MIR663A		GeneCard	
302 2	MicroRNA 664a	MIR664A		GeneCard	DisGenet
302 3	MicroRNA 6742	MIR6742		GeneCard	
302 4	MicroRNA 6873	MIR6873		GeneCard	

302<br>5	MicroRNA 6886	MIR6886		GeneCard	
302<br>6	MicroRNA 6891	MIR6891		GeneCard	
302<br>7	MicroRNA 758	MIR758		GeneCard	DisGenet
302<br>8	MicroRNA 761	MIR761		GeneCard	
302<br>9	MicroRNA 769	MIR769		GeneCard	
303<br>0	MicroRNA 873	MIR873		GeneCard	
303<br>1	MicroRNA 876	MIR876			DisGenet
303<br>2	MicroRNA 9-1	MIR9-1		GeneCard	
303<br>3	MicroRNA 9-2	MIR9-2		GeneCard	
303<br>4	MicroRNA 92a-1	MIR92A1		GeneCard	
303<br>5	MicroRNA 92a-2	MIR92A2		GeneCard	
303<br>6	MicroRNA 92b	MIR92B		GeneCard	
303<br>7	MicroRNA 93	MIR93		GeneCard	
303<br>8	MicroRNA 942	MIR942		GeneCard	
303<br>9	MicroRNA 98	MIR98		GeneCard	DisGenet
304<br>0	MicroRNA 99a	MIR99A		GeneCard	DisGenet
304<br>1	MicroRNA Let-7a-1	MIRLET7A1		GeneCard	
304<br>2	MicroRNA Let-7a-2	MIRLET7A2		GeneCard	
304<br>3	MicroRNA Let-7a-3	MIRLET7A3		GeneCard	
304<br>4	MicroRNA Let-7b	MIRLET7B		GeneCard	DisGenet

3045	MicroRNA Let-7c	MIRLET7C		GeneCard	
3046	MicroRNA Let-7d	MIRLET7D		GeneCard	
3047	MicroRNA Let-7e	MIRLET7E		GeneCard	
3048	MicroRNA Let-7f-1	MIRLET7F1		GeneCard	
3049	MicroRNA Let-7g	MIRLET7G		GeneCard	DisGenet
3050	Mitochondrial Matrix Import Factor 23	MIX23		GeneCard	
3051	Marker Of Proliferation Ki-67	MKI67		GeneCard	
3052	MAPK Interacting Serine/Threonine Kinase 1	MKNK1		GeneCard	
3053	Modulator of VRAC Current 1	MLC1			DisGenet
3054	MutL Homolog 1	MLH1		GeneCard	
3055	MutL Homolog 3	MLH3		GeneCard	
3056	Mixed Lineage Kinase Domain Like Pseudokinase	MLKL		GeneCard	DisGenet
3057	Motilin	MLN		GeneCard	
3058	MTOR Associated Protein, LST8 Homolog	MLST8		GeneCard	
3059	MAX Dimerization Protein MLX	MLX		GeneCard	
3060	MLX Interacting Protein Like	MLXIPL		GeneCard	DisGenet
3061	Metabolism Of Cobalamin Associated C	MMACHC	OMIM	GeneCard	
3062	Monocyte to Macrophage Differentiation Associated	MMD			DisGenet
3063	Membrane Metalloendopeptidase	MME		GeneCard	
3064	Matrix Metallopeptidase 1	MMP1		GeneCard	DisGenet

3065	Matrix Metallopeptidase 10	MMP10		GeneCard	DisGenet
3066	Matrix Metallopeptidase 11	MMP11		GeneCard	DisGenet
3067	Matrix Metallopeptidase 12	MMP12	OMIM	GeneCard	DisGenet
3068	Matrix Metallopeptidase 13	MMP13		GeneCard	DisGenet
3069	Matrix Metallopeptidase 14	MMP14		GeneCard	DisGenet
3070	Matrix Metallopeptidase 15	MMP15		GeneCard	
3071	Matrix Metallopeptidase 17	MMP17		GeneCard	DisGenet
3072	Matrix Metallopeptidase 19	MMP19		GeneCard	
3073	Matrix Metallopeptidase 2	MMP2		GeneCard	DisGenet
3074	Matrix Metallopeptidase 26	MMP26		GeneCard	
3075	Matrix Metalloproteinase 3 (stromelysin 1, progelatinase)	MMP3	OMIM		DisGenet
3076	Matrix Metallopeptidase 7	MMP7		GeneCard	DisGenet
3077	Matrix Metallopeptidase 8	MMP8		GeneCard	DisGenet
3078	Matrix Metallopeptidase 9	MMP9		GeneCard	DisGenet
3079	Multimerin 1	MMRN1		GeneCard	DisGenet
3080	Malignant Mesothelioma	MMS			DisGenet
3081	Meiotic Nuclear Divisions 1	MND1		GeneCard	
3082	Myeloid Cell Nuclear Differentiation Antigen	MNDA		GeneCard	DisGenet
3083	Meiosis Specific Nuclear Structural 1	MNS1		GeneCard	
3084	Myelin Associated Oligodendrocyte Basic Protein	MOBP		GeneCard	

308 5	Molybdenum Cofactor Sulfurase	MOCOS		GeneCard	DisGenet
308 6	Molybdenum Cofactor Synthesis 1	MOCS1		GeneCard	
308 7	Molybdenum Cofactor Synthesis 2	MOCS2		GeneCard	
308 8	MOK protein kinase	MOK			DisGenet
308 9	MORC Family CW-Type Zinc Finger 1	MORC1		GeneCard	
309 0	Macrophage Expressed 1	MPEG1			DisGenet
309 1	Mannose Phosphate Isomerase	MPI		GeneCard	
309 2	MPL Proto-Oncogene, Thrombopoietin Receptor	MPL		GeneCard	
309 3	Myeloperoxidase	MPO		GeneCard	DisGenet
309 4	MAGUK P55 Scaffold Protein 1	MPP1		GeneCard	
309 5	MAGUK P55 Scaffold Protein 7	MPP7		GeneCard	
309 6	Myosin Phosphatase Rho Interacting Protein	MPRIP		GeneCard	DisGenet
309 7	Myelin Protein Zero Like 2	MPZL2			DisGenet
309 8	Muscle RAS Oncogene Homolog	MRAS		GeneCard	
309 9	Mannose Receptor C-Type 1	MRC1		GeneCard	DisGenet
310 0	MRE11 Homolog, Double Strand Break Repair Nuclease	MRE11		GeneCard	
310 1	Maestro Heat Like Repeat Family Member 5 (Gene/Pseudogene)	MROH5		GeneCard	
310 2	Mitochondrial Ribosomal Protein L10	MRPL10		GeneCard	
310 3	Mitochondrial Ribosomal Protein L14	MRPL14		GeneCard	
310 4	Mitochondrial Ribosomal Protein S22	MRPS22		GeneCard	

3105	Mitochondrial Ribosomal Protein S36 Pseudogene 3	MRPS36P3		GeneCard	
3106	Myocardin Related Transcription Factor A	MRTFA		GeneCard	DisGenet
3107	Myocardin Related Transcription Factor B	MRTFB		GeneCard	
3108	Membrane Spanning 4-Domains A1	MS4A1			DisGenet
3109	Membrane Spanning 4-Domains A13	MS4A13		GeneCard	
3110	Membrane Spanning 4-Domains A2	MS4A2		GeneCard	
3111	Membrane Spanning 4-Domains A4E	MS4A4E		GeneCard	
3112	Minisatellite Binding Protein 1	MSBP1		GeneCard	
3113	Minisatellite Binding Protein 2	MSBP2		GeneCard	
3114	MSC Antisense RNA 1	MSC-AS1		GeneCard	
3115	MutS Homolog 3	MSH3		GeneCard	
3116	MutS Homolog 4	MSH4		GeneCard	
3117	MutS Homolog 5	MSH5		GeneCard	
3118	Musashi RNA Binding Protein 2	MSI2		GeneCard	
3119	Methylsterol Monooxygenase 1	MSMO1		GeneCard	
3120	Macrophage scavenger receptor	MSR1	OMIM	GeneCard	DisGenet
3121	Methionine Sulfoxide Reductase A	MSRA		GeneCard	DisGenet
3122	Methionine Sulfoxide Reductase B1	MSRB1		GeneCard	
3123	Macrophage Stimulating 1	MST1			DisGenet
3124	Macrophage Stimulating 1 Receptor	MST1R			DisGenet

3125	Myostatin	MSTN		GeneCard	DisGenet
3126	Msh Homeobox 2	MSX2		GeneCard	
3127	Metallothionein 1F	MT1F			DisGenet
3128	Metallothionein 1X	MT1X		GeneCard	
3129	Metallothionein 2A	MT2A		GeneCard	DisGenet
3130	Metallothionein 3	MT3		GeneCard	
3131	Metastasis Associated 1	MTA1		GeneCard	
3132	Metastasis Associated 1 Family Member 2	MTA2		GeneCard	
3133	Metastasis Associated 1 Family Member 3	MTA3		GeneCard	
3134	Methylthioadenosine Phosphorylase	MTAP		GeneCard	DisGenet
3135	Mitochondrially Encoded ATP Synthase Membrane Subunit 6	MT-ATP6		GeneCard	
3136	Mitochondrially Encoded Cytochrome C Oxidase I	MT-CO1		GeneCard	
3137	Mitochondrially Encoded Cytochrome C Oxidase II	MT-CO2		GeneCard	
3138	MT-CO2 Pseudogene 12	MTCO2P12			DisGenet
3139	Mitochondrially Encoded Cytochrome C Oxidase III	MT-CO3		GeneCard	
3140	Mitochondrially Encoded Cytochrome B	MT-CYB		GeneCard	
3141	Mitochondrial Fission Process 1	MTFP1		GeneCard	
3142	Methylenetetrahydrofolate Dehydrogenase, Cyclohydrolase and Formyltetrahydrofolate Synthetase 1	MTHFD1		GeneCard	
3143	Methylenetetrahydrofolate Dehydrogenase (NADP+ Dependent) 1 Like	MTHFD1L		GeneCard	
3144	Methylenetetrahydrofolate Dehydrogenase (NADP+ Dependent) 2, Methenyltetrahydrofolate Cyclohydrolase	MTHFD2		GeneCard	DisGenet

3145	Methylenetetrahydrofolate Reductase	MTHFR		GeneCard	DisGenet
3146	Methenyltetrahydrofolate Synthetase	MTHFS		GeneCard	
3147	Mitochondrially Encoded Long Non-Coding Cardiac Associated RNA	MT-LIPCAR		GeneCard	
3148	Myotubularin 1	MTM1		GeneCard	
3149	Myotubularin Related Protein 2	MTMR2		GeneCard	
3150	Myotubularin Related Protein 6	MTMR6		GeneCard	
3151	Mitochondrially Encoded NADH:Ubiquinone Oxidoreductase Core Subunit 1	MT-ND1		GeneCard	
3152	Mitochondrially Encoded NADH:Ubiquinone Oxidoreductase Core Subunit 2	MT-ND2		GeneCard	
3153	Mitochondrially Encoded NADH:Ubiquinone Oxidoreductase Core Subunit 6	MT-ND6		GeneCard	
3154	Melatonin Receptor 1A	MTNR1A		GeneCard	
3155	Melatonin Receptor 1B	MTNR1B		GeneCard	
3156	Mitochondrial TRNA Translation Optimization 1	MTO1		GeneCard	
3157	Mechanistic Target of Rapamycin Kinase	MTOR		GeneCard	DisGenet
3158	Myotrophin	MTPN		GeneCard	
3159	5-Methyltetrahydrofolate-Homocysteine Methyltransferase	MTR		GeneCard	
3160	Mitochondrially Encoded 16S RRNA	MT-RNR2		GeneCard	
3161	MT-RNR2 Like 8 (Pseudogene)	MTRNR2L8		GeneCard	
3162	5-Methyltetrahydrofolate-Homocysteine Methyltransferase Reductase	MTRR		GeneCard	DisGenet
3163	MTSS I-BAR Domain Containing 1	MTSS1			DisGenet
3164	Mitochondrially Encoded TRNA-Ile (AUU/C)	MT-TI		GeneCard	

3165	Mitochondrially Encoded TRNA-Leu (UUA/G) 1	MT-TL1		GeneCard	
3166	Microsomal Triglyceride Transfer Protein	MTTP	OMIM	GeneCard	DisGenet
3167	Microtubule Associated Scaffold Protein 1	MTUS1		GeneCard	
3168	Mucin 1, Cell Surface Associated	MUC1		GeneCard	DisGenet
3169	Mucin 16, Cell Surface Associated	MUC16		GeneCard	DisGenet
3170	MUS81 Structure-Specific Endonuclease Subunit	MUS81		GeneCard	
3171	Muscle Associated Receptor Tyrosine Kinase	MUSK		GeneCard	
3172	MutY DNA Glycosylase	MUTYH		GeneCard	
3173	Mevalonate Kinase	MVK		GeneCard	
3174	Major Vault Protein	MVP		GeneCard	DisGenet
3175	MX Dynamin Like GTPase 1	MX1		GeneCard	
3176	MAX Dimerization Protein 1	MXD1		GeneCard	
3177	MAX Dimerization Protein 3	MXD3		GeneCard	
3178	Matrix Remodeling Associated 7	MXRA7		GeneCard	
3179	MYB Proto-Oncogene, Transcription Factor	MYB		GeneCard	
3180	MYB Proto-Oncogene Like 2	MYBL2		GeneCard	
3181	Myosin Binding Protein C1	MYBPC1		GeneCard	
3182	Myosin Binding Protein C2	MYBPC2		GeneCard	
3183	Myosin Binding Protein C3	MYBPC3		GeneCard	
3184	Myosin Binding Protein H Like	MYBPHL		GeneCard	

318<br>5	MYC Proto-Oncogene, BHLH Transcription Factor	MYC		GeneCard	DisGenet
318<br>6	MYCN Proto-Oncogene, BHLH Transcription Factor	MYCN		GeneCard	
318<br>7	MYD88 Innate Immune Signal Transduction Adaptor	MYD88	OMIM	GeneCard	DisGenet
318<br>8	Myeloid Derived Growth Factor	MYDGF		GeneCard	DisGenet
318<br>9	Myosin Heavy Chain 10	MYH10		GeneCard	
319<br>0	Myosin Heavy Chain 11	MYH11		GeneCard	DisGenet
319<br>1	Myosin Heavy Chain 14	MYH14		GeneCard	
319<br>2	Myosin Heavy Chain 15	MYH15		GeneCard	DisGenet
319<br>3	Myosin Heavy Chain 6	MYH6		GeneCard	
319<br>4	Myosin Heavy Chain 7	MYH7		GeneCard	
319<br>5	Myosin Heavy Chain 7B	MYH7B		GeneCard	
319<br>6	Myosin Heavy Chain 9	MYH9		GeneCard	DisGenet
319<br>7	Myosin Light Chain 12A	MYL12A		GeneCard	
319<br>8	Myosin Light Chain 2	MYL2		GeneCard	
319<br>9	Myosin Light Chain 3	MYL3		GeneCard	
320<br>0	Myosin Light Chain 4	MYL4		GeneCard	
320<br>1	Myosin Light Chain 6	MYL6		GeneCard	
320<br>2	Myosin Regulatory Light Chain Interacting Protein	MYLIP		GeneCard	
320<br>3	Myosin Light Chain Kinase	MYLK		GeneCard	DisGenet
320<br>4	Myosin Light Chain Kinase 3	MYLK3		GeneCard	

3205	MYLK Antisense RNA 1	MYLK-AS1		GeneCard	
3206	Moyamoya Disease 4	MYMY4		GeneCard	
3207	Myosin IE	MYO1E		GeneCard	
3208	Myosin VA	MYO5A		GeneCard	
3209	Myosin VIIA	MYO7A		GeneCard	
3210	Myosin VIIB	MYO7B		GeneCard	
3211	Myocardin	MYOCD		GeneCard	DisGenet
3212	Myogenic Differentiation 1	MYOD1		GeneCard	
3213	Myomesin 1	MYOM1		GeneCard	
3214	Myomesin 3	MYOM3		GeneCard	
3215	Myotilin	MYOT		GeneCard	
3216	Myozenin 1	MYOZ1		GeneCard	
3217	Myozenin 2	MYOZ2		GeneCard	
3218	Myopalladin	MYPN		GeneCard	
3219	Myocardial Zonula Adherens Protein	MYZAP		GeneCard	
3220	Marginal Zone B And B1 Cell Specific Protein	MZB1			DisGenet
3221	N-Alpha-Acetyltransferase 25, Natb Auxiliary Subunit	NAA25			DisGenet
3222	N-Acylethanolamine Acid Amidase	NAAA			DisGenet
3223	NGFI-A Binding Protein 1	NAB1		GeneCard	
3224	NGFI-A Binding Protein 2	NAB2		GeneCard	

3225	Nucleic Acid Binding Protein 1	NABP1		GeneCard	
3226	N-Acetyl-Alpha-Glucosaminidase	NAGLU			DisGenet
3227	NALCN Channel Auxiliary Factor 1	NALF1		GeneCard	
3228	Nicotinamide Phosphoribosyltransferase	NAMPT		GeneCard	DisGenet
3229	Nanos C2HC-Type Zinc Finger 2	NANOS2			DisGenet
3230	Nanos C2HC-Type Zinc Finger 3	NANOS3		GeneCard	DisGenet
3231	N-Acetylneuraminate Synthase	NANS			DisGenet
3232	Nucleosome Assembly Protein 1 Like 1 Pseudogene 1	NAP1L1P1		GeneCard	
3233	NSF Attachment Protein Gamma	NAPG		GeneCard	
3234	Nuclear Autoantigenic Sperm Protein Pseudogene 1	NASPP1		GeneCard	
3235	N-Acetyltransferase 1	NAT1		GeneCard	
3236	N-Acetyltransferase 10	NAT10		GeneCard	
3237	N-Acetyltransferase 2	NAT2		GeneCard	DisGenet
3238	N-Acetyltransferase 8 (Putative)	NAT8		GeneCard	
3239	Neuron Navigator 1	NAV1		GeneCard	
3240	NAD(P)HX Epimerase	NAXE		GeneCard	DisGenet
3241	NBAS subunit of NRZ tethering complex	NBAS			DisGenet
3242	Neurobeachin Like 2	NBEAL2			DisGenet
3243	NBL1, DAN Family BMP Antagonist	NBL1		GeneCard	DisGenet
3244	Nibrin	NBN		GeneCard	DisGenet

324<br>5	NBPF Member 3	NBPF3		GeneCard	
324<br>6	Non-SMC Condensin II Complex Subunit H2	NCAPH2		GeneCard	
324<br>7	Neutral Cholesterol Ester Hydrolase 1	NCEH1	OMIM	GeneCard	DisGenet
324<br>8	Neutrophil Cytosolic Factor 1	NCF1		GeneCard	DisGenet
324<br>9	Neutrophil Cytosolic Factor 2	NCF2		GeneCard	DisGenet
325<br>0	NCK Adaptor Protein 1	NCK1		GeneCard	
325<br>1	Nucleolin	NCL		GeneCard	
325<br>2	Nuclear Receptor Coactivator 1	NCOA1		GeneCard	
325<br>3	Nuclear Receptor Coactivator 2	NCOA2		GeneCard	
325<br>4	Nuclear Receptor Coactivator 3	NCOA3		GeneCard	
325<br>5	Nuclear Receptor Coactivator 5	NCOA5		GeneCard	
325<br>6	Nuclear Receptor Corepressor 1	NCOR1		GeneCard	DisGenet
325<br>7	Nuclear Receptor Corepressor 2	NCOR2		GeneCard	
325<br>8	Natural Cytotoxicity Triggering Receptor 2	NCR2		GeneCard	
325<br>9	Non-Protein Coding RNA, Upstream Of F2R/PAR1	NCRUPAR		GeneCard	
326<br>0	Nicastrin	NCSTN		GeneCard	
326<br>1	NudE Neurodevelopment Protein 1	NDE1		GeneCard	
326<br>2	Norrin Cystine Knot Growth Factor NDP	NDP		GeneCard	
326<br>3	N-Myc Downstream Regulated 1	NDRG1		GeneCard	
326<br>4	NDRG Family Member 2	NDRG2		GeneCard	

3265	NADH:Ubiquinone Oxidoreductase Subunit A10	NDUFA10		GeneCard	
3266	NADH:Ubiquinone Oxidoreductase Subunit A2	NDUFA2			DisGenet
3267	NADH:Ubiquinone Oxidoreductase Subunit A9	NDUFA9		GeneCard	
3268	NADH:Ubiquinone Oxidoreductase Subunit AB1	NDUFAB1			DisGenet
3269	NADH:Ubiquinone Oxidoreductase Complex Assembly Factor 2	NDUFAF2		GeneCard	
3270	NADH:Ubiquinone Oxidoreductase Subunit B4	NDUFB4		GeneCard	
3271	NADH:Ubiquinone Oxidoreductase Subunit B8	NDUFB8		GeneCard	
3272	NADH:Ubiquinone Oxidoreductase Core Subunit S2	NDUFS2		GeneCard	
3273	NADH:Ubiquinone Oxidoreductase Subunit S4	NDUFS4		GeneCard	
3274	NADH:Ubiquinone Oxidoreductase Subunit S6	NDUFS6		GeneCard	
3275	NADH:Ubiquinone Oxidoreductase Core Subunit V1	NDUFV1		GeneCard	
3276	Nuclear Paraspeckle Assembly Transcript 1	NEAT1		GeneCard	DisGenet
3277	Nebulette	NEBL		GeneCard	
3278	Nectin Cell Adhesion Molecule 1	NECTIN1			DisGenet
3279	Nectin Cell Adhesion Molecule 2	NECTIN2		GeneCard	DisGenet
3280	NEDD4 E3 Ubiquitin Protein Ligase	NEDD4		GeneCard	DisGenet
3281	NEDD8 Ubiquitin Like Modifier	NEDD8		GeneCard	
3282	Neurofilament Light Chain	NEFL		GeneCard	
3283	Nei Like DNA Glycosylase 1	NEIL1		GeneCard	
3284	Nei Like DNA Glycosylase 2	NEIL2		GeneCard	

3285	Nei Like DNA Glycosylase 3	NEIL3		GeneCard	
3286	NIMA Related Kinase 7	NEK7		GeneCard	
3287	NIMA Related Kinase 9	NEK9		GeneCard	
3288	Nestin	NES		GeneCard	DisGenet
3289	Neuropilin And Tolloid Like 1	NETO1		GeneCard	
3290	Neuraminidase 1	NEU1	OMIM	GeneCard	DisGenet
3291	Neuraminidase 3	NEU3		GeneCard	
3292	Neuralized E3 Ubiquitin Protein Ligase 1	NEURL1		GeneCard	
3293	Nexilin F-Actin Binding Protein	NEXN	OMIM	GeneCard	DisGenet
3294	NEXN Antisense RNA 1	NEXN-AS1	OMIM	GeneCard	DisGenet
3295	Neurofibromin 1	NF1		GeneCard	DisGenet
3296	NF2, Moesin-Ezrin-Radixin Like (MERLIN) Tumor Suppressor	NF2		GeneCard	
3297	NFAT Activating Protein With ITAM Motif 1	NFAM1		GeneCard	
3298	Nuclear Factor Of Activated T Cells 5	NFAT5		GeneCard	DisGenet
3299	Nuclear Factor Of Activated T Cells 1	NFATC1		GeneCard	
3300	Nuclear Factor Of Activated T Cells 2	NFATC2		GeneCard	
3301	Nuclear Factor Of Activated T Cells 3	NFATC3		GeneCard	
3302	Nuclear Factor Of Activated T Cells 4	NFATC4		GeneCard	
3303	Nuclear Factor, Erythroid 2	NFE2			DisGenet
3304	NFE2 Like BZIP Transcription Factor 1	NFE2L1		GeneCard	

3305	NFE2 Like BZIP Transcription Factor 2	NFE2L2		GeneCard	DisGenet
3306	Nuclear Factor I A	NFIA		GeneCard	DisGenet
3307	NFIA Antisense RNA 1	NFIA-AS1		GeneCard	
3308	Nuclear Factor Kappa B Subunit 1	NFKB1	OMIM	GeneCard	DisGenet
3309	Nuclear Factor Kappa B Subunit 2	NFKB2	OMIM	GeneCard	DisGenet
3310	NFKB Inhibitor Alpha	NFKBIA		GeneCard	DisGenet
3311	NFKB Inhibitor Beta	NFKBIB		GeneCard	
3312	NFKB Inhibitor Epsilon	NFKBIE		GeneCard	
3313	NFKB Inhibitor Like 1	NFKBIL1		GeneCard	
3314	NFS1 Cysteine Desulfurase	NFS1		GeneCard	
3315	Neuroglobin	NGB		GeneCard	DisGenet
3316	Neuronal Guanine Nucleotide Exchange Factor	NGEF		GeneCard	
3317	Nerve Growth Factor	NGF		GeneCard	
3318	Nerve Growth Factor Receptor	NGFR		GeneCard	DisGenet
3319	Non-Homologous End Joining Factor 1	NHEJ1		GeneCard	
3320	NHL Repeat Containing E3 Ubiquitin Protein Ligase 1	NHLRC1		GeneCard	
3321	NGG1 Interacting Factor 3 Like 1	NIF3L1			DisGenet
3322	Ninjurin 1	NINJ1		GeneCard	
3323	Ninjurin 2	NINJ2		GeneCard	DisGenet
3324	Nipsnap Homolog 3B	NIPSNAP3B		GeneCard	

332<br>5	Nischarin	NISCH		GeneCard	DisGenet
332<br>6	Nitrilase Family Member 2	NIT2		GeneCard	
332<br>7	NFKB Activating Protein Like	NKAPL		GeneCard	
332<br>8	NK2 Homeobox 5	NKX2-5		GeneCard	DisGenet
332<br>9	NLR Family CARD Domain Containing 4	NLRC4		GeneCard	
333<br>0	NLR Family Pyrin Domain Containing 1	NLRP1		GeneCard	
333<br>1	NLR Family Pyrin Domain Containing 11	NLRP11		GeneCard	
333<br>2	NLR Family Pyrin Domain Containing 12	NLRP12		GeneCard	
333<br>3	NLR Family, Pyrin Domain Containing 3	NLRP3	OMIM	GeneCard	DisGenet
333<br>4	NLR Family Pyrin Domain Containing 5	NLRP5		GeneCard	
333<br>5	NME1-NME2 readthrough	NME1-NME2			DisGenet
333<br>6	NME/NM23 Nucleoside Diphosphate Kinase 2	NME2		GeneCard	DisGenet
333<br>7	NME/NM23 Nucleoside Diphosphate Kinase 4	NME4		GeneCard	
333<br>8	NME/NM23 Family Member 8	NME8		GeneCard	
333<br>9	N-Myc And STAT Interactor	NMI		GeneCard	
334<br>0	Nicotinamide N-Methyltransferase	NNMT		GeneCard	DisGenet
334<br>1	Nicotinamide Nucleotide Transhydrogenase	NNT			DisGenet
334<br>2	NOBOX Oogenesis Homeobox	NOBOX		GeneCard	
334<br>3	Nucleotide Binding Oligomerization Domain Containing 1	NOD1		GeneCard	DisGenet
334<br>4	Nucleotide Binding Oligomerization Domain Containing 2	NOD2		GeneCard	DisGenet

3345	Noggin	NOG		GeneCard	
3346	Nucleolar Protein 3	NOL3		GeneCard	
3347	NODAL Modulator 1	NOMO1		GeneCard	
3348	NODAL Modulator 2	NOMO2		GeneCard	
3349	NODAL Modulator 3	NOMO3		GeneCard	
3350	NOP2 Nucleolar Protein	NOP2			DisGenet
3351	Non-Coding RNA Activated by DNA Damage	NORAD		GeneCard	DisGenet
3352	Nitric Oxide Synthase 1	NOS1		GeneCard	DisGenet
3353	Nitric Oxide Synthase 1 Adaptor Protein	NOS1AP		GeneCard	DisGenet
3354	Nitric Oxide Synthase 2	NOS2		GeneCard	DisGenet
3355	Nitric oxide synthase 3, endothelial cell	NOS3	OMIM	GeneCard	DisGenet
3356	Nitric Oxide Synthase Interacting Protein	NOSIP		GeneCard	
3357	Notch Receptor 1	NOTCH1		GeneCard	DisGenet
3358	Notch Receptor 2	NOTCH2		GeneCard	DisGenet
3359	Notch Receptor 3	NOTCH3		GeneCard	DisGenet
3360	Notch Receptor 4	NOTCH4		GeneCard	
3361	NADPH Oxidase 1	NOX1		GeneCard	DisGenet
3362	NADPH Oxidase 4	NOX4		GeneCard	DisGenet
3363	NADPH Oxidase 5	NOX5		GeneCard	DisGenet
3364	NADPH Oxidase Activator 1	NOXA1		GeneCard	DisGenet

3365	NADPH Oxidase Organizer 1	NOXO1		GeneCard	
3366	Neuropeptide B	NPB		GeneCard	
3367	NPC Intracellular Cholesterol Transporter 1	NPC1		GeneCard	DisGenet
3368	NPC1-like intracellular cholesterol transporter 1	NPC1L1	OMIM	GeneCard	DisGenet
3369	NPC1 Like Intracellular Cholesterol Transporter 1	NPC2		GeneCard	
3370	NPC Intracellular Cholesterol Transporter 2	NPEPPS		GeneCard	DisGenet
3371	NPHS1 Adhesion Molecule, Nephrin	NPHS1		GeneCard	
3372	NPHS2 Stomatin Family Member, Podocin	NPHS2		GeneCard	DisGenet
3373	Nucleophosmin 1	NPM1		GeneCard	
3374	Nephronectin	NPNT		GeneCard	
3375	Natriuretic Peptide A	NPPA		GeneCard	DisGenet
3376	Natriuretic Peptide B	NPPB		GeneCard	DisGenet
3377	Natriuretic Peptide C	NPPC		GeneCard	DisGenet
3378	Natriuretic Peptide Receptor 1	NPR1		GeneCard	
3379	Natriuretic Peptide Receptor 2	NPR2		GeneCard	
3380	Natriuretic Peptide Receptor 3	NPR3		GeneCard	
3381	Neuropeptide S	NPS		GeneCard	
3382	Neuronal Pentraxin Receptor	NPTXR		GeneCard	
3383	Neuropeptide VF Precursor	NPVF		GeneCard	
3384	Neuropeptide Y	NPY	OMIM	GeneCard	DisGenet

3385	Neuropeptide Y Receptor Y1	NPY1R		GeneCard	
3386	Neuropeptide Y Receptor Y4	NPY4R		GeneCard	
3387	Neuropeptide Y Receptor Y5	NPY5R		GeneCard	
3388	NAD(P)H Quinone Dehydrogenase 1	NQO1		GeneCard	DisGenet
3389	Nuclear Receptor Subfamily 0 Group B Member 1	NR0B1		GeneCard	DisGenet
3390	Nuclear Receptor Subfamily 0 Group B Member 2	NR0B2			DisGenet
3391	Nuclear Receptor Subfamily 1 Group D Member 1	NR1D1		GeneCard	DisGenet
3392	Nuclear Receptor Subfamily 1 Group D Member 2	NR1D2		GeneCard	
3393	Nuclear Receptor Subfamily 1 Group H Member 2	NR1H2		GeneCard	
3394	Nuclear Receptor Subfamily 1 Group H Member 3	NR1H3	OMIM	GeneCard	DisGenet
3395	Nuclear Receptor Subfamily 1 Group H Member 4	NR1H4		GeneCard	DisGenet
3396	Nuclear Receptor Subfamily 1 Group I Member 2	NR1I2		GeneCard	DisGenet
3397	Nuclear Receptor Subfamily 1 Group I Member 3	NR1I3		GeneCard	DisGenet
3398	Nuclear Receptor Subfamily 2 Group F Member 2	NR2F2		GeneCard	DisGenet
3399	Nuclear Receptor Subfamily 3 Group C Member 1	NR3C1		GeneCard	DisGenet
3400	Nuclear Receptor Subfamily 3 Group C Member 2	NR3C2		GeneCard	DisGenet
3401	Nuclear Receptor Subfamily 4 Group A Member 1	NR4A1		GeneCard	DisGenet
3402	Nuclear Receptor Subfamily 4 Group A Member 2	NR4A2		GeneCard	
3403	Nuclear Receptor Subfamily 4 Group A Member 3	NR4A3		GeneCard	DisGenet
3404	Nuclear Receptor Subfamily 5 Group A Member 1	NR5A1		GeneCard	

340<br>5	Nuclear Receptor Subfamily 5 Group A Member 2	NR5A2		GeneCard	DisGenet
340<br>6	Nuclear Receptor Subfamily 6 Group A Member 1	NR6A1		GeneCard	DisGenet
340<br>7	Nebulin Related Anchoring Protein	NRAP		GeneCard	
340<br>8	Nuclear Receptor Binding Factor 2	NRBF2		GeneCard	
340<br>9	Nuclear Respiratory Factor 1	NRF1		GeneCard	DisGenet
341<br>0	Neuregulin 1	NRG1		GeneCard	DisGenet
341<br>1	Neuregulin 3	NRG3		GeneCard	
341<br>2	Neuregulin 4	NRG4		GeneCard	DisGenet
341<br>3	Nuclear Receptor Interacting Protein 1	NRIP1		GeneCard	DisGenet
341<br>4	Neuropilin 1	NRP1		GeneCard	DisGenet
341<br>5	Neuropilin 2	NRP2		GeneCard	
341<br>6	Nuclear Receptor Binding SET Domain Protein 1	NSD1		GeneCard	
341<br>7	N-Ethylmaleimide Sensitive Factor, Vesicle Fusing ATPase	NSF		GeneCard	
341<br>8	NOP2/Sun RNA Methyltransferase 5	NSUN5			DisGenet
341<br>9	5'-Nucleotidase Ecto	NT5E		GeneCard	DisGenet
342<br>0	Neurotrimin	NTM		GeneCard	
342<br>1	Netrin 1	NTN1		GeneCard	DisGenet
342<br>2	Netrin G1	NTNG1		GeneCard	
342<br>3	Neurotrophic Receptor Tyrosine Kinase 1	NTRK1		GeneCard	
342<br>4	Neurotrophic Receptor Tyrosine Kinase 2	NTRK2		GeneCard	

342<br>5	Negative Regulator of Ubiquitin Like Proteins 1	NUB1		GeneCard	
342<br>6	Nucleobindin 2	NUCB2		GeneCard	
342<br>7	NudC Domain Containing 1	NUDCD1		GeneCard	
342<br>8	Nudix Hydrolase 10	NUDT10		GeneCard	
342<br>9	Nudix Hydrolase 6	NUDT6	OMIM	GeneCard	
343<br>0	Nuclear GTPase, Germinal Center Associated	NUGGC		GeneCard	
343<br>1	Nuclear Mitotic Apparatus Protein 1	NUMA1		GeneCard	
343<br>2	NUMB Endocytic Adaptor Protein	NUMB		GeneCard	
343<br>3	Nucleoporin 205	NUP205		GeneCard	
343<br>4	Nucleoporin 62	NUP62			DisGenet
343<br>5	Nucleoporin 93	NUP93		GeneCard	
343<br>6	Nucleoporin 98 And 96 Precursor	NUP98		GeneCard	
343<br>7	NUS1 Dehydrodolichyl Diphosphate Synthase Subunit	NUS1		GeneCard	
343<br>8	Nuclear RNA Export Factor 5	NXF5		GeneCard	
343<br>9	2'-5'-Oligoadenylate Synthetase 1	OAS1		GeneCard	
344<br>0	2'-5'-Oligoadenylate Synthetase Like	OASL		GeneCard	
344<br>1	Ornithine Decarboxylase Antizyme 1	OAZ1		GeneCard	
344<br>2	Obscurin, Cytoskeletal Calmodulin and Titin-Interacting RhoGEF	OBSCN		GeneCard	
344<br>3	Occludin	OCLN			DisGenet
344<br>4	Ornithine Decarboxylase 1	ODC1		GeneCard	

3445	Orofacial Cleft 1 Candidate 1	OFCC1		GeneCard	
3446	O-GlcNAcase	OGA			DisGenet
3447	Oxoglutarate Dehydrogenase	OGDH		GeneCard	
3448	8-Oxoguanine DNA Glycosylase	OGG1		GeneCard	DisGenet
3449	Osteoglycin	OGN		GeneCard	DisGenet
3450	Opa Interacting Protein 5	OIP5		GeneCard	
3451	OIP5 Antisense RNA 1	OIP5-AS1		GeneCard	
3452	Olfactomedin Like 2B	OLFML2B		GeneCard	
3453	Low density lipoprotein, oxidized, receptor 1	OLR1	OMIM	GeneCard	DisGenet
3454	Osteomodulin	OMD		GeneCard	
3455	Olfactory Marker Protein	OMP		GeneCard	
3456	Opsin 3	OPN3		GeneCard	
3457	Opioid Receptor Kappa 1	OPRK1			DisGenet
3458	Opioid Receptor Mu 1	OPRM1		GeneCard	
3459	Optineurin	OPTN		GeneCard	
3460	Olfactory Receptor Family 10 Subfamily A Member 4	OR10A4			DisGenet
3461	Olfactory Receptor Family 13 Subfamily G Member 1	OR13G1		GeneCard	
3462	Olfactory Receptor Family 2 Subfamily B Member 7 Pseudogene	OR2B7P		GeneCard	
3463	Olfactory Receptor Family 6 Subfamily A Member 2	OR6A2		GeneCard	
3464	ORAI Calcium Release-Activated Calcium Modulator 1	ORAI1		GeneCard	DisGenet

3465	Orosomucoid 1	ORM1		GeneCard	
3466	ORMDL Sphingolipid Biosynthesis Regulator 3	ORMDL3		GeneCard	DisGenet
3467	Oxysterol Binding Protein	OSBP		GeneCard	DisGenet
3468	Oxysterol Binding Protein 2	OSBP2		GeneCard	
3469	Oxysterol Binding Protein Like 10	OSBPL10		GeneCard	
3470	Oxysterol Binding Protein Like 1A	OSBPL1A		GeneCard	DisGenet
3471	Oxysterol Binding Protein Like 5	OSBPL5		GeneCard	
3472	Oxysterol Binding Protein Like 6	OSBPL6		GeneCard	
3473	Oxysterol Binding Protein Like 8	OSBPL8		GeneCard	DisGenet
3474	Oxysterol Binding Protein Like 9	OSBPL9		GeneCard	
3475	Oxysterol Binding Protein Like 9 Pseudogene 2	OSBPL9P2		GeneCard	
3476	Osteoclast Associated Ig-Like Receptor	OSCAR		GeneCard	DisGenet
3477	Oxidative Stress Induced Growth Inhibitor 1	OSCP1		GeneCard	DisGenet
3478	Oxidative Stress Induced Growth Inhibitor 1	OSGIN1			DisGenet
3479	Oncostatin M	OSM		GeneCard	DisGenet
3480	Oncostatin M Receptor	OSMR		GeneCard	DisGenet
3481	Otoferlin	OTOF		GeneCard	
3482	OTU Deubiquitinase 7A	OTUD7A		GeneCard	
3483	OTU Deubiquitinase 7B	OTUD7B		GeneCard	
3484	OXA1L Mitochondrial Inner Membrane Protein	OXA1L		GeneCard	

348<br>5	Oxoglutarate Receptor 1	OXGR1		GeneCard	
348<br>6	Oxytocin/Neurophysin I Prepropeptide	OXT		GeneCard	
348<br>7	Oxytocin Receptor	OXTR		GeneCard	
348<br>8	Purinergic Receptor P2X 1	P2RX1		GeneCard	DisGenet
348<br>9	Purinergic Receptor P2X 2	P2RX2		GeneCard	DisGenet
349<br>0	Purinergic Receptor P2X 3	P2RX3			DisGenet
349<br>1	Purinergic Receptor P2X 4	P2RX4		GeneCard	DisGenet
349<br>2	Purinergic Receptor P2X 5	P2RX5			DisGenet
349<br>3	P2RX5-TAX1BP3 readthrough (NMD candidate)	P2RX5-TAX1BP3			DisGenet
349<br>4	Purinergic Receptor P2X 6	P2RX6			DisGenet
349<br>5	Purinergic Receptor P2X 7	P2RX7		GeneCard	DisGenet
349<br>6	Purinergic Receptor P2Y1	P2RY1		GeneCard	DisGenet
349<br>7	Purinergic Receptor P2Y12	P2RY12	OMIM	GeneCard	DisGenet
349<br>8	Purinergic Receptor P2Y13	P2RY13			DisGenet
349<br>9	Purinergic Receptor P2Y2	P2RY2		GeneCard	DisGenet
350<br>0	Pyrimidinergic Receptor P2Y4	P2RY4		GeneCard	
350<br>1	Prolyl 4-Hydroxylase Subunit Alpha 1	P4HA1		GeneCard	
350<br>2	Prolyl 4-Hydroxylase Subunit Alpha 2	P4HA2		GeneCard	
350<br>3	Prolyl 4-Hydroxylase Subunit Alpha 3	P4HA3	OMIM	GeneCard	
350<br>4	Prolyl 4-Hydroxylase Subunit Beta	P4HB		GeneCard	DisGenet

3505	Phosphofurin Acidic Cluster Sorting Protein 2	PACS2			DisGenet
3506	Peptidyl Arginine Deiminase 1	PADI1			DisGenet
3507	Peptidyl Arginine Deiminase 4	PADI4		GeneCard	DisGenet
3508	progestagen associated endometrial protein	PAEP			DisGenet
3509	Platelet Activating Factor Acetylhydrolase 1b Regulatory Subunit 1	PAFAH1B1		GeneCard	
3510	Platelet Activating Factor Acetylhydrolase 2	PAFAH2		GeneCard	
3511	Phosphoprotein Membrane Anchor With Glycosphingolipid Microdomains 1	PAG1		GeneCard	
3512	Phenylalanine Hydroxylase	PAH		GeneCard	DisGenet
3513	P21 (RAC1) Activated Kinase 1	PAK1		GeneCard	DisGenet
3514	Phosphatase Domain Containing Paladin 1	PALD1			DisGenet
3515	Palladin, Cytoskeletal Associated Protein	PALLD		GeneCard	DisGenet
3516	Pannexin 1	PANX1		GeneCard	DisGenet
3517	Peripheral Arterial Occlusive Disease 1	PAOD1		GeneCard	
3518	Polyamine Oxidase	PAOX			DisGenet
3519	Pappalysin 1	PAPPA	OMIM	GeneCard	DisGenet
3520	Progestin and AdipoQ Receptor Family Member 5	PAQR5		GeneCard	
3521	Progestin and AdipoQ Receptor Family Member 7	PAQR7			DisGenet
3522	Par-3 Family Cell Polarity Regulator	PARD3		GeneCard	
3523	Parkinsonism Associated Deglycase	PARK7		GeneCard	
3524	Poly(ADP-Ribose) Polymerase 1	PARP1		GeneCard	DisGenet

3525	Poly(ADP-Ribose) Polymerase Family Member 14	PARP14		GeneCard	
3526	Poly(ADP-Ribose) Polymerase 2	PARP2		GeneCard	
3527	Poly(ADP-ribose) Polymerase Family Member 3	PARP3			DisGenet
3528	Poly(ADP-Ribose) Polymerase Family Member 9	PARP9		GeneCard	
3529	Prostate Androgen-Regulated Transcript 1	PART1			DisGenet
3530	Parvin Alpha	PARVA		GeneCard	
3531	Parvin Beta	PARVB		GeneCard	
3532	PATJ Crumbs Cell Polarity Complex Component	PATJ		GeneCard	
3533	POZ/BTB And AT Hook Containing Zinc Finger 1	PATZ1		GeneCard	DisGenet
3534	Pro-Apoptotic WT1 Regulator	PAWR		GeneCard	
3535	Paired Box 6	PAX6		GeneCard	
3536	PDZ Binding Kinase	PBK		GeneCard	
3537	Polybromo 1	PBRM1		GeneCard	
3538	Pyruvate Carboxylase	PC			DisGenet
3539	Poly(RC) Binding Protein 1	PCBP1		GeneCard	
3540	Propionyl-CoA Carboxylase Subunit Alpha	PCCA		GeneCard	
3541	Propionyl-CoA Carboxylase Subunit Beta	PCCB		GeneCard	
3542	Protocadherin Related 15	PCDH15		GeneCard	
3543	Protocadherin 8	PCDH8			DisGenet
3544	Protocadherin 9	PCDH9		GeneCard	

3545	Phosphorylated CTD Interacting Factor 1	PCIF1		GeneCard	
3546	Phosphoenolpyruvate Carboxykinase 1	PCK1		GeneCard	
3547	Phosphoenolpyruvate Carboxykinase 2, Mitochondrial	PCK2		GeneCard	
3548	PCNA Clamp Associated Factor	PCLAF			DisGenet
3549	Protein-L-Isoaspartate (D-Aspartate) O-Methyltransferase	PCMT1		GeneCard	
3550	Proliferating Cell Nuclear Antigen	PCNA		GeneCard	DisGenet
3551	Procollagen C-Endopeptidase Enhancer	PCOLCE			DisGenet
3552	Procollagen C-Endopeptidase Enhancer 2	PCOLCE2		GeneCard	DisGenet
3553	Polycystic Ovary Syndrome 1	PCOS1			DisGenet
3554	Purkinje Cell Protein 4	PCP4			DisGenet
3555	Proprotein Convertase Subtilisin/Kexin Type 1	PCSK1		GeneCard	DisGenet
3556	Proprotein Convertase Subtilisin/Kexin Type 1 Inhibitor	PCSK1N		GeneCard	
3557	Proprotein Convertase Subtilisin/Kexin Type 2	PCSK2		GeneCard	
3558	Proprotein Convertase Subtilisin/Kexin Type 5	PCSK5	OMIM	GeneCard	DisGenet
3559	Proprotein Convertase Subtilisin/Kexin Type 6	PCSK6		GeneCard	DisGenet
3560	Proprotein Convertase Subtilisin/Kexin Type 7	PCSK7		GeneCard	
3561	Proprotein Convertase Subtilisin/Kexin Type 9	PCSK9		GeneCard	DisGenet
3562	Prenylcysteine Oxidase 1	PCYOX1		GeneCard	
3563	Phosphate Cytidylyltransferase 1A, Choline	PCYT1A		GeneCard	
3564	Phosphate Cytidylyltransferase 1, Choline, Beta	PCYT1B			DisGenet

3565	Programmed Cell Death 1	PDCD1		GeneCard	
3566	Programmed Cell Death 10	PDCD10		GeneCard	
3567	Programmed Cell Death 4	PDCD4		GeneCard	DisGenet
3568	Programmed Cell Death 5	PDCD5		GeneCard	DisGenet
3569	Programmed Cell Death 6 Interacting Protein	PDCD6IP		GeneCard	DisGenet
3570	Phosphodiesterase 1A	PDE1A	OMIM	GeneCard	DisGenet
3571	Phosphodiesterase 1B	PDE1B		GeneCard	
3572	Phosphodiesterase 1C	PDE1C		GeneCard	
3573	Phosphodiesterase 3A	PDE3A		GeneCard	
3574	Phosphodiesterase 3B	PDE3B		GeneCard	
3575	Phosphodiesterase 4A	PDE4A		GeneCard	DisGenet
3576	Phosphodiesterase 4B	PDE4B		GeneCard	
3577	Phosphodiesterase 4D	PDE4D	OMIM	GeneCard	DisGenet
3578	Phosphodiesterase 5A	PDE5A		GeneCard	DisGenet
3579	Phosphodiesterase 6A	PDE6A		GeneCard	
3580	Phosphodiesterase 6H	PDE6H		GeneCard	
3581	Phosphodiesterase 7B	PDE7B		GeneCard	
3582	Phosphodiesterase 9A	PDE9A		GeneCard	DisGenet
3583	PDE9A Antisense RNA 1	PDE9A-AS1		GeneCard	
3584	Platelet Derived Growth Factor Subunit A	PDGFA	OMIM	GeneCard	DisGenet

358<br>5	Platelet Derived Growth Factor Subunit B	PDGFB		GeneCard	DisGenet
358<br>6	Platelet Derived Growth Factor C	PDGFC		GeneCard	DisGenet
358<br>7	Platelet Derived Growth Factor D	PDGFD		GeneCard	DisGenet
358<br>8	Platelet Derived Growth Factor Receptor Alpha	PDGFRA		GeneCard	
358<br>9	Platelet Derived Growth Factor Receptor Beta	PDGFRB		GeneCard	DisGenet
359<br>0	Protein Disulfide Isomerase Family A Member 2	PDIA2		GeneCard	DisGenet
359<br>1	Protein Disulfide Isomerase Family A Member 3	PDIA3		GeneCard	
359<br>2	Protein Disulfide Isomerase Family A Member 2	PDIK1L			DisGenet
359<br>3	Pyruvate Dehydrogenase Kinase 1	PDK1			DisGenet
359<br>4	Pyruvate Dehydrogenase Kinase 4	PDK4		GeneCard	DisGenet
359<br>5	PDZ And LIM Domain 3	PDLIM3		GeneCard	
359<br>6	PDZ And LIM Domain 5	PDLIM5		GeneCard	DisGenet
359<br>7	PDZ And LIM Domain 7	PDLIM7		GeneCard	DisGenet
359<br>8	Pyruvate Dehydrogenase Phosphatase Catalytic Subunit 1	PDP1		GeneCard	
359<br>9	3-Phosphoinositide Dependent Protein Kinase 1	PDPK1		GeneCard	
360<br>0	Podoplanin	PDPN		GeneCard	
360<br>1	Decaprenyl Diphosphate Synthase Subunit 2	PDSS2		GeneCard	
360<br>2	Pancreatic And Duodenal Homeobox 1	PDX1		GeneCard	
360<br>3	Pyridoxal Phosphatase	PDXP		GeneCard	
360<br>4	Prodynorphin	PDYN		GeneCard	

3605	PDZ Domain Containing 1	PDZK1		GeneCard	DisGenet
3606	PDZK1 Interacting Protein 1	PDZK1IP1		GeneCard	
3607	Platelet Endothelial Aggregation Receptor 1	PEAR1		GeneCard	
3608	Phosphatidylethanolamine Binding Protein 1	PEBP1		GeneCard	DisGenet
3609	Phosphatidylethanolamine Binding Protein 1 Pseudogene 2	PEBP1P2		GeneCard	
3610	Platelet And Endothelial Cell Adhesion Molecule 1	PECAM1		GeneCard	DisGenet
3611	Plaque Enriched LncRNA In Atherosclerotic And Inflammatory Bowel Macrophage Regulation	PELATON		GeneCard	
3612	Phosphatidylethanolamine N-Methyltransferase	PEMT		GeneCard	DisGenet
3613	Peptidase D	PEPD		GeneCard	
3614	Peroxisomal Biogenesis Factor 11 Alpha	PEX11A		GeneCard	
3615	Peroxisomal Biogenesis Factor 19	PEX19		GeneCard	
3616	Peroxisomal Biogenesis Factor 3	PEX3		GeneCard	
3617	Peroxisomal Biogenesis Factor 6	PEX6		GeneCard	
3618	Platelet Factor 4	PF4		GeneCard	DisGenet
3619	Platelet Factor 4 Variant 1	PF4V1		GeneCard	
3620	Prefoldin Subunit 1	PFDN1		GeneCard	
3621	Profilin 1	PFN1		GeneCard	
3622	Profilin 3	PFN3		GeneCard	
3623	Progastricsin	PGC		GeneCard	DisGenet
3624	Placental Growth Factor	PGF		GeneCard	DisGenet

362<br>5	Phosphoglycerate Kinase 1	PGK1		GeneCard	
362<br>6	Peptidoglycan Recognition Protein 1	PGLYRP1		GeneCard	
362<br>7	Phosphoglucomutase 1	PGM1		GeneCard	
362<br>8	Pyroglutamyl-Peptidase I	PGPEP1			DisGenet
362<br>9	Progesterone Receptor	PGR		GeneCard	
363<br>0	PGR antisense RNA 1	PGR-AS1			DisGenet
363<br>1	Progesterone Receptor Membrane Component 1	PGRMC1		GeneCard	
363<br>2	Phosphatase And Actin Regulator 1	PHACTR1		GeneCard	DisGenet
363<br>3	Phosphate Regulating Endopeptidase Homolog X-Linked	PHEX		GeneCard	
363<br>4	PHD Finger Protein 14	PHF14			DisGenet
363<br>5	Phosphorylase Kinase Regulatory Subunit Alpha 2	PHKA2		GeneCard	
363<br>6	Pleckstrin Homology Like Domain Family A Member 1	PHLDA1	OMIM	GeneCard	
363<br>7	Phosphoethanolamine/Phosphocholine Phosphatase 1	PHOSPHO1		GeneCard	
363<br>8	Paired like Homeobox 2B	PHOX2B			DisGenet
363<br>9	PHD And Ring Finger Domains 1	PHRF1		GeneCard	
364<br>0	Peptidase Inhibitor 16	PI16		GeneCard	
364<br>1	Peptidase Inhibitor 3	PI3		GeneCard	
364<br>2	Protein Inhibitor Of Activated STAT 1	PIAS1		GeneCard	
364<br>3	Protein Inhibitor Of Activated STAT 4	PIAS4		GeneCard	
364<br>4	Phosphotyrosine Interaction Domain Containing 1	PID1		GeneCard	

3645	Piezo Type Mechanosensitive Ion Channel Component 1	PIEZO1		GeneCard	DisGenet
3646	Phosphatidylinositol Glycan Anchor Biosynthesis Class A	PIGA		GeneCard	
3647	Phosphatidylinositol Glycan Anchor Biosynthesis Class M	PIGM		GeneCard	
3648	Polymeric Immunoglobulin Receptor	PIGR		GeneCard	
3649	Phosphatidylinositol-4-Phosphate 3-Kinase Catalytic Subunit Type 2 Alpha	PIK3C2A		GeneCard	
3650	Phosphatidylinositol-4-Phosphate 3-Kinase Catalytic Subunit Type 2 Gamma	PIK3C2G		GeneCard	
3651	Phosphatidylinositol-4,5-Bisphosphate 3-Kinase Catalytic Subunit Alpha	PIK3CA		GeneCard	DisGenet
3652	Phosphatidylinositol-4,5-Bisphosphate 3-Kinase Catalytic Subunit Beta	PIK3CB		GeneCard	DisGenet
3653	Phosphatidylinositol-4,5-Bisphosphate 3-Kinase Catalytic Subunit Delta	PIK3CD			DisGenet
3654	Phosphatidylinositol-4,5-Bisphosphate 3-Kinase Catalytic Subunit Gamma	PIK3CG		GeneCard	DisGenet
3655	Phosphoinositide-3-Kinase Regulatory Subunit 1	PIK3R1		GeneCard	
3656	Phosphoinositide-3-Kinase Regulatory Subunit 3	PIK3R3		GeneCard	DisGenet
3657	Phosphoinositide-3-Kinase Regulatory Subunit 5	PIK3R5			DisGenet
3658	Pim-1 Proto-Oncogene, Serine/Threonine Kinase	PIM1		GeneCard	
3659	Peptidylprolyl Cis/Trans Isomerase, NIMA-Interacting 1	PIN1		GeneCard	DisGenet
3660	PTEN Induced Kinase 1	PINK1		GeneCard	DisGenet
3661	PIN2 (TERF1) Interacting Telomerase Inhibitor 1	PINX1		GeneCard	DisGenet
3662	PINX1 Divergent Transcript	PINX1-DT		GeneCard	
3663	Prolactin Induced Protein	PIP			DisGenet
3664	Phosphatidylinositol-4-Phosphate 5-Kinase Like 1	PIP5KL1		GeneCard	

3665	Piwi-Interacting RNA Cluster 66	PIRC66		GeneCard	
3666	Phosphatidylinositol Transfer Protein Alpha	PITPNA		GeneCard	
3667	Pitrilysin Metallopeptidase 1	PITRM1		GeneCard	
3668	Paired Like Homeodomain 1	PITX1		GeneCard	
3669	Paired Like Homeodomain 2	PITX2		GeneCard	DisGenet
3670	Paired Like Homeodomain 3	PITX3			DisGenet
3671	Piwi Like RNA-Mediated Gene Silencing 1	PIWIL1		GeneCard	
3672	Piwi Like RNA-Mediated Gene Silencing 4	PIWIL4		GeneCard	
3673	Praja Ring Finger Ubiquitin Ligase 1	PJA1		GeneCard	
3674	Polycystin 1, Transient Receptor Potential Channel Interacting	PKD1		GeneCard	
3675	Polycystin 2, Transient Receptor Potential Cation Channel	PKD2		GeneCard	
3676	Polycystin 2 Like 1, Transient Receptor Potential Cation Channel	PKD2L1		GeneCard	
3677	Pyruvate Kinase M1/2	PKM		GeneCard	DisGenet
3678	Protein Kinase N1	PKN1		GeneCard	
3679	Protein Kinase N2	PKN2		GeneCard	
3680	Plakophilin 2	PKP2		GeneCard	
3681	Phospholipase A2 Group X	PLA2G10		GeneCard	DisGenet
3682	Phospholipase A2 Group XIIA	PLA2G12A			DisGenet
3683	Phospholipase A2 Group XV	PLA2G15		GeneCard	DisGenet
3684	Phospholipase A2 Group IB	PLA2G1B		GeneCard	DisGenet

3685	Phospholipase A2 Group IIA	PLA2G2A		GeneCard	DisGenet
3686	Phospholipase A2 Group IID	PLA2G2D		GeneCard	DisGenet
3687	Phospholipase A2 Group III	PLA2G3		GeneCard	DisGenet
3688	Phospholipase A2 Group IVA	PLA2G4A	OMIM	GeneCard	DisGenet
3689	Phospholipase A2 Group IVC	PLA2G4C			DisGenet
3690	Phospholipase A2 Group V	PLA2G5		GeneCard	
3691	Phospholipase A2 Group VI	PLA2G6		GeneCard	DisGenet
3692	Phospholipase A2, group VII (platelet-activating factor acetylhydrolase)	PLA2G7	OMIM	GeneCard	DisGenet
3693	Phospholipase A2 Receptor 1	PLA2R1		GeneCard	
3694	Phospholipase A and Acyltransferase 1	PLAAT1			DisGenet
3695	PLAG1 Zinc Finger	PLAG1		GeneCard	DisGenet
3696	Plasminogen Activator, Tissue Type	PLAT		GeneCard	DisGenet
3697	Plasminogen Activator, Urokinase	PLAU	OMIM	GeneCard	DisGenet
3698	Plasminogen Activator, Urokinase Receptor	PLAUR		GeneCard	DisGenet
3699	Phospholipase B1	PLB1			DisGenet
3700	Phospholipase C Beta 1	PLCB1		GeneCard	
3701	Phospholipase C Beta 2	PLCB2		GeneCard	
3702	Phospholipase C Beta 3	PLCB3	OMIM	GeneCard	
3703	Phospholipase C Gamma 1	PLCG1		GeneCard	
3704	Phospholipase C Gamma 2	PLCG2		GeneCard	

3705	Phospholipase C Eta 1	PLCH1		GeneCard	
3706	Phospholipase C Like 2	PLCL2		GeneCard	
3707	Phospholipase D2	PLD2		GeneCard	
3708	Phospholipase D Family Member 5	PLD5		GeneCard	
3709	Plectin	PLEC		GeneCard	
3710	Pleckstrin	PLEK		GeneCard	
3711	Pleckstrin Homology Domain Containing A1	PLEKHA1		GeneCard	
3712	Pleckstrin Homology And RUN Domain Containing M2	PLEKHM2		GeneCard	
3713	Pleckstrin Homology Domain Containing O1	PLEKHO1		GeneCard	DisGenet
3714	Plasminogen	PLG		GeneCard	DisGenet
3715	Perilipin 1	PLIN1		GeneCard	DisGenet
3716	Perilipin 2	PLIN2		GeneCard	DisGenet
3717	Perilipin 3	PLIN3		GeneCard	
3718	Perilipin 5	PLIN5			DisGenet
3719	Phospholamban	PLN		GeneCard	
3720	Procollagen-Lysine,2-Oxoglutarate 5-Dioxygenase 1	PLOD1		GeneCard	
3721	Proteolipid Protein 1	PLP1		GeneCard	
3722	Phospholipid Phosphatase 3	PLPP3		GeneCard	DisGenet
3723	Phospholipid Transfer Protein	PLTP		GeneCard	DisGenet
3724	Plexin A2	PLXNA2			DisGenet

3725	Plexin A4	PLXNA4		GeneCard	
3726	Plexin B1	PLXNB1		GeneCard	
3727	Plexin B2	PLXNB2		GeneCard	
3728	Plexin D1	PLXND1	OMIM	GeneCard	
3729	Prostate Transmembrane Protein, Androgen Induced 1	PMEPA1		GeneCard	
3730	PML Nuclear Body Scaffold	PML		GeneCard	
3731	Phosphomannomutase 2	PMM2		GeneCard	
3732	Peptidase, Mitochondrial Processing Subunit Alpha	PMPCA		GeneCard	DisGenet
3733	Peptidase, Mitochondrial Processing Subunit Beta	PMPCB		GeneCard	
3734	PMS1 Homolog 2, Mismatch Repair System Component	PMS2		GeneCard	
3735	Phosphomevalonate Kinase	PMVK		GeneCard	
3736	PNKD metallo-beta-lactamase domain containing	PNKD			DisGenet
3737	Pancreatic Lipase	PNLIP		GeneCard	
3738	Pancreatic Lipase Related Protein 1	PNLIPRP1		GeneCard	
3739	Partner Of NOB1 Homolog	PNO1			DisGenet
3740	Purine Nucleoside Phosphorylase	PNP			DisGenet
3741	Patatin Like Phospholipase Domain Containing 2	PNPLA2		GeneCard	DisGenet
3742	Patatin Like Phospholipase Domain Containing 3	PNPLA3		GeneCard	DisGenet
3743	Patatin Like Phospholipase Domain Containing 5	PNPLA5		GeneCard	
3744	Podocan	PODN		GeneCard	DisGenet

374<br>5	Podocalyxin Like	PODXL		GeneCard	DisGenet
374<br>6	POF1B Actin Binding Protein	POF1B		GeneCard	
374<br>7	DNA Polymerase Beta	POLB		GeneCard	
374<br>8	DNA Polymerase Delta Interacting Protein 2	POLDIP2		GeneCard	DisGenet
374<br>9	DNA Polymerase Gamma, Catalytic Subunit	POLG		GeneCard	
375<br>0	DNA Polymerase Gamma 2, Accessory Subunit	POLG2		GeneCard	
375<br>1	RNA Polymerase I Subunit A	POLR1A		GeneCard	
375<br>2	RNA Polymerase I And III Subunit C	POLR1C		GeneCard	
375<br>3	RNA Polymerase I Subunit G	POLR1G		GeneCard	
375<br>4	RNA Polymerase I Subunit H	POLR1H		GeneCard	
375<br>5	RNA Polymerase II Subunit A	POLR2A		GeneCard	
375<br>6	RNA Polymerase II Subunit B	POLR2B		GeneCard	
375<br>7	RNA Polymerase II Subunit D	POLR2D		GeneCard	
375<br>8	RNA Polymerase II, I And III Subunit L	POLR2L		GeneCard	
375<br>9	RNA Polymerase III Subunit H	POLR3H		GeneCard	
376<br>0	POM121 Transmembrane Nucleoporin Like 3, Pseudogene	POM121L3P		GeneCard	
376<br>1	Proopiomelanocortin	POMC		GeneCard	DisGenet
376<br>2	Protein O-Linked Mannose N-Acetylglucosaminyltransferase 2 (Beta 1,4-)	POMGNT2		GeneCard	
376<br>3	Paraoxonase-1	PON1	OMIM	GeneCard	DisGenet
376<br>4	Paraoxonase 2	PON2		GeneCard	DisGenet

3765	Paraoxonase-3	PON3	OMIM	GeneCard	DisGenet
3766	Cytochrome P450 Oxidoreductase	POR		GeneCard	
3767	Periostin	POSTN		GeneCard	DisGenet
3768	Protection Of Telomeres 1	POT1			DisGenet
3769	POTE Ankyrin Domain Family Member F	POTEF			DisGenet
3770	POTE Ankyrin Domain Family Member K, Pseudogene	POTEKP		GeneCard	
3771	POU Class 1 Homeobox 1	POU1F1		GeneCard	
3772	POU Class 2 Homeobox 1	POU2F1		GeneCard	DisGenet
3773	POU Class 2 Homeobox 3	POU2F3		GeneCard	DisGenet
3774	POU Class 5 Homeobox 1	POU5F1		GeneCard	
3775	Peroxisome Proliferator Activated Receptor Alpha	PPARA		GeneCard	DisGenet
3776	Peroxisome proliferative activated receptor, delta	PPARD	OMIM	GeneCard	DisGenet
3777	Peroxisome proliferator activated receptor, gamma	PPARG	OMIM	GeneCard	DisGenet
3778	PPARG Coactivator 1 Alpha	PPARGC1A		GeneCard	DisGenet
3779	PPARG Coactivator 1 Beta	PPARGC1B		GeneCard	
3780	Pro-Platelet Basic Protein	PPBP		GeneCard	DisGenet
3781	Phosphopantothenoylcysteine Synthetase	PPCS		GeneCard	
3782	PTPRF Interacting Protein Alpha 2	PPFIA2		GeneCard	
3783	Peptidylprolyl Isomerase A	PPIA		GeneCard	DisGenet
3784	Peptidylprolyl Isomerase A Like 4A	PPIAL4A		GeneCard	

3785	Peptidylprolyl Isomerase F	PPIF		GeneCard	
3786	Peptidylprolyl Isomerase G	PPIG		GeneCard	DisGenet
3787	Protein Phosphatase, Mg2+/Mn2+ Dependent 1A	PPM1A		GeneCard	
3788	Protein Phosphatase, Mg2+/Mn2+ Dependent 1B	PPM1B		GeneCard	
3789	Protein Phosphatase, Mg2+/Mn2+ Dependent 1D	PPM1D			DisGenet
3790	Protein Phosphatase, Mg2+/Mn2+ Dependent 1K	PPM1K		GeneCard	
3791	Protein Phosphatase 1 Catalytic Subunit Gamma	PPP1CC		GeneCard	
3792	Protein Phosphatase 1 Regulatory Inhibitor Subunit 11	PPP1R11		GeneCard	
3793	Protein Phosphatase 1 Regulatory Subunit 12A	PPP1R12A		GeneCard	
3794	Protein Phosphatase 1 Regulatory Subunit 12B	PPP1R12B		GeneCard	
3795	Protein Phosphatase 1 Regulatory Subunit 12C	PPP1R12C		GeneCard	
3796	Protein Phosphatase 1 Regulatory Subunit 13 Like	PPP1R13L		GeneCard	
3797	Protein Phosphatase 1 Regulatory Subunit 15A	PPP1R15A		GeneCard	
3798	Protein Phosphatase 1 Regulatory Subunit 17	PPP1R17		GeneCard	
3799	Protein Phosphatase 1 Regulatory Inhibitor Subunit 1A	PPP1R1A		GeneCard	DisGenet
3800	Protein Phosphatase 1 Regulatory Subunit 3A	PPP1R3A		GeneCard	
3801	Protein Phosphatase 1 Regulatory Subunit 3B	PPP1R3B		GeneCard	DisGenet
3802	Protein Phosphatase 2 Catalytic Subunit Beta	PPP2CB		GeneCard	
3803	Protein Phosphatase 2 Regulatory Subunit B, Alpha	PPP2R2A			DisGenet
3804	Protein Phosphatase 3 Regulatory Subunit B, Alpha	PPP3R1		GeneCard	

380<br>5	Palmitoyl-Protein Thioesterase 2	PPT2		GeneCard	
380<br>6	Proline Rich Acidic Protein 1	PRAP1			DisGenet
380<br>7	PR/SET Domain 1	PRDM1		GeneCard	DisGenet
380<br>8	PR/SET Domain 16	PRDM16		GeneCard	
380<br>9	PR/SET Domain 9	PRDM9		GeneCard	
381<br>0	Peroxiredoxin 1	PRDX1	OMIM	GeneCard	DisGenet
381<br>1	Peroxiredoxin 4	PRDX4		GeneCard	DisGenet
381<br>2	Peroxiredoxin 5	PRDX5		GeneCard	
381<br>3	Peroxiredoxin 6	PRDX6		GeneCard	
381<br>4	PRELI Domain Containing 1	PRELID1		GeneCard	
381<br>5	Prolyl Endopeptidase	PREP			DisGenet
381<br>6	Prolyl Endopeptidase Like	PREPL		GeneCard	
381<br>7	Perforin 1	PRF1		GeneCard	
381<br>8	Proteoglycan 4	PRG4		GeneCard	DisGenet
381<br>9	Prickle Planar Cell Polarity Protein 1	PRICKLE1		GeneCard	
382<br>0	Prickle Planar Cell Polarity Protein 2	PRICKLE2		GeneCard	
382<br>1	Protein Kinase AMP-Activated Catalytic Subunit Alpha 1	PRKAA1		GeneCard	DisGenet
382<br>2	Protein Kinase AMP-Activated Catalytic Subunit Alpha 2	PRKAA2		GeneCard	DisGenet
382<br>3	Protein Kinase AMP-Activated Non-Catalytic Subunit Beta 1	PRKAB1		GeneCard	DisGenet
382<br>4	Protein Kinase AMP-Activated Non-Catalytic Subunit Beta 2	PRKAB2		GeneCard	

3825	Protein Kinase AMP-Activated Non-Catalytic Subunit Gamma 1	PRKAG1		GeneCard	
3826	Protein Kinase AMP-Activated Non-Catalytic Subunit Gamma 2	PRKAG2		GeneCard	
3827	Protein Kinase AMP-Activated Non-Catalytic Subunit Gamma 3	PRKAG3		GeneCard	
3828	Protein Kinase CAMP-Dependent Type I Regulatory Subunit Alpha	PRKAR1A		GeneCard	DisGenet
3829	Protein Kinase C Alpha	PRKCA		GeneCard	DisGenet
3830	Protein Kinase C Beta	PRKCB		GeneCard	DisGenet
3831	Protein Kinase C Delta	PRKCD		GeneCard	
3832	Protein Kinase C Epsilon	PRKCE		GeneCard	
3833	Protein Kinase C Gamma	PRKCG		GeneCard	
3834	Protein Kinase C Eta	PRKCH		GeneCard	
3835	Protein Kinase C Iota	PRKCI		GeneCard	
3836	Protein Kinase C Zeta	PRKCZ		GeneCard	
3837	Protein Kinase D1	PRKD1		GeneCard	
3838	Protein Kinase D2	PRKD2		GeneCard	
3839	Protein Kinase, DNA-Activated, Catalytic Subunit	PRKDC		GeneCard	
3840	Protein Kinase CGMP-Dependent 1	PRKG1		GeneCard	
3841	Protein Kinase CGMP-Dependent 2	PRKG2		GeneCard	
3842	Parkin RBR E3 Ubiquitin Protein Ligase	PRKN		GeneCard	
3843	Prolactin	PRL		GeneCard	DisGenet
3844	Prolactin Receptor	PRLR		GeneCard	

3845	Protein Arginine Methyltransferase 1	PRMT1		GeneCard	
3846	Protein Arginine Methyltransferase 3	PRMT3		GeneCard	DisGenet
3847	Protein Arginine Methyltransferase 5 Pseudogene 1	PRMT5P1		GeneCard	
3848	Protein Arginine Methyltransferase 7	PRMT7		GeneCard	
3849	Prion Protein	PRNP		GeneCard	DisGenet
3850	Protein C, Inactivator Of Coagulation Factors Va And VIIIa	PROC		GeneCard	DisGenet
3851	Protein C Receptor	PROCR		GeneCard	DisGenet
3852	Proline Dehydrogenase 1	PRODH		GeneCard	
3853	Prominin 1	PROM1		GeneCard	
3854	Protein S	PROS1		GeneCard	DisGenet
3855	Prospero Homeobox 1	PROX1			DisGenet
3856	Protein Z, Vitamin K Dependent Plasma Glycoprotein	PROZ		GeneCard	
3857	Pre-MRNA Processing Factor 8	PRPF8		GeneCard	
3858	Phosphoribosyl Pyrophosphate Synthetase 1	PRPS1		GeneCard	
3859	Proline Rich 7, Synaptic	PRR7		GeneCard	
3860	Paired Related Homeobox 1	PRRX1		GeneCard	DisGenet
3861	Serine Protease 1	PRSS1		GeneCard	
3862	Serine Protease 2	PRSS2		GeneCard	
3863	Serine Protease 33	PRSS33		GeneCard	
3864	Serine Protease 55	PRSS55			DisGenet

3865	Phosphoribosyl Transferase Domain Containing 1	PRTFDC1		GeneCard	
3866	Proteinase 3	PRTN3		GeneCard	DisGenet
3867	Prosaposin	PSAP		GeneCard	
3868	Phosphoserine Aminotransferase 1	PSAT1			DisGenet
3869	Presenilin 1	PSEN1		GeneCard	
3870	Presenilin 2	PSEN2		GeneCard	
3871	Pregnancy Specific Beta-1-Glycoprotein 1	PSG1		GeneCard	
3872	Pregnancy Specific Beta-1-Glycoprotein 2	PSG2		GeneCard	
3873	PC4 And SFRS1 Interacting Protein 1 Pseudogene 1	PSIP1P1		GeneCard	
3874	Proteasome 20S Subunit Alpha 6	PSMA6		GeneCard	DisGenet
3875	Proteasome 20S Subunit Beta 8	PSMB8		GeneCard	DisGenet
3876	Proteasome 20S Subunit Beta 9	PSMB9		GeneCard	
3877	PSMC3 Interacting Protein	PSMC3IP		GeneCard	
3878	Proteasome 26S Subunit, ATPase 5	PSMC5		GeneCard	DisGenet
3879	Proteasome 26S Subunit, ATPase 6	PSMC6		GeneCard	
3880	Proteasome 26S Subunit, Non-ATPase 6	PSMD6		GeneCard	
3881	Proteasome 26S Subunit, Non-ATPase 7	PSMD7			DisGenet
3882	Proteasome 26S Subunit, Non-ATPase 9	PSMD9		GeneCard	DisGenet
3883	Proteasome Activator Subunit 3	PSME3		GeneCard	DisGenet
3884	Proline And Serine Rich Coiled-Coil 1	PSRC1		GeneCard	DisGenet

3885	Potocki–Shaffer Syndrome	PSS			DisGenet
3886	Proline-Serine-Threonine Phosphatase Interacting Protein 2	PSTPIP2			DisGenet
3887	Platelet Activating Factor Receptor	PTAFR		GeneCard	DisGenet
3888	Polypyrimidine Tract Binding Protein 1	PTBP1			DisGenet
3889	Patched 1	PTCH1		GeneCard	
3890	Papillary Thyroid Carcinoma Susceptibility Candidate 3	PTCSC3		GeneCard	
3891	Phosphatase And Tensin Homolog	PTEN		GeneCard	DisGenet
3892	Prostaglandin D2 Receptor	PTGDR		GeneCard	
3893	Prostaglandin D2 Synthase	PTGDS	OMIM	GeneCard	DisGenet
3894	Prostaglandin E Receptor 1	PTGER1		GeneCard	
3895	Prostaglandin E Receptor 2	PTGER2		GeneCard	
3896	Prostaglandin E Receptor 3	PTGER3		GeneCard	DisGenet
3897	Prostaglandin E Receptor 4	PTGER4		GeneCard	DisGenet
3898	Prostaglandin E Synthase	PTGES		GeneCard	DisGenet
3899	Prostaglandin E Synthase 2	PTGES2		GeneCard	
3900	Prostaglandin E Synthase 3	PTGES3		GeneCard	
3901	Prostaglandin I2 Receptor	PTGIR		GeneCard	DisGenet
3902	Prostaglandin I2 Synthase	PTGIS		GeneCard	DisGenet
3903	Prostaglandin Reductase 1	PTGR1		GeneCard	
3904	Prostaglandin-Endoperoxide Synthase 1	PTGS1		GeneCard	DisGenet

3905	Prostaglandin-Endoperoxide Synthase 2	PTGS2		GeneCard	DisGenet
3906	Parathyroid Hormone	PTH		GeneCard	DisGenet
3907	Parathyroid Hormone 1 Receptor	PTH1R		GeneCard	
3908	Parathyroid Hormone 2 Receptor	PTH2R		GeneCard	
3909	Parathyroid Hormone Like Hormone	PTHLH		GeneCard	DisGenet
3910	Protein Tyrosine Kinase 2	PTK2		GeneCard	
3911	Protein Tyrosine Kinase 2 Beta	PTK2B		GeneCard	DisGenet
3912	Pleiotrophin	PTN		GeneCard	DisGenet
3913	PTOV1 Extended AT-Hook Containing Adaptor Protein	PTOV1		GeneCard	DisGenet
3914	Protein Phosphatase 2 Phosphatase Activator	PTPA		GeneCard	DisGenet
3915	Protein Tyrosine Phosphatase Non-Receptor Type 1	PTPN1		GeneCard	DisGenet
3916	Protein Tyrosine Phosphatase Non-Receptor Type 11	PTPN11		GeneCard	DisGenet
3917	Protein Tyrosine Phosphatase Non-Receptor Type 2	PTPN2		GeneCard	DisGenet
3918	Protein Tyrosine Phosphatase Non-Receptor Type 22	PTPN22		GeneCard	DisGenet
3919	Protein Tyrosine Phosphatase Non-Receptor Type 3	PTPN3		GeneCard	
3920	Protein Tyrosine Phosphatase Non-Receptor Type 6	PTPN6		GeneCard	DisGenet
3921	Protein Tyrosine Phosphatase Receptor Type A	PTPRA		GeneCard	
3922	Protein Tyrosine Phosphatase Receptor Type C	PTPRC		GeneCard	
3923	Protein Tyrosine Phosphatase Receptor Type D	PTPRD		GeneCard	
3924	Protein Tyrosine Phosphatase Receptor Type E	PTPRE		GeneCard	

3925	Protein Tyrosine Phosphatase Receptor Type F	PTPRF		GeneCard	DisGenet
3926	Protein Tyrosine Phosphatase Receptor Type N2	PTPRN2		GeneCard	
3927	6-Pyruvoyltetrahydropterin Synthase	PTS		GeneCard	
3928	Pituitary Tumor-Transforming 2	PTTG2		GeneCard	
3929	Pentraxin 3	PTX3		GeneCard	DisGenet
3930	P53 Upregulated Regulator Of P53 Levels	PURPL		GeneCard	
3931	PVR Cell Adhesion Molecule	PVR			DisGenet
3932	Pvt1 Oncogene	PVT1		GeneCard	
3933	Prader Willi/Angelman region RNA 1	PWAR1			DisGenet
3934	Peroxidasin	PXDN		GeneCard	
3935	PX Domain Containing Serine/Threonine Kinase Like	PXK		GeneCard	
3936	Paxillin	PXN		GeneCard	
3937	PYD And CARD Domain Containing	PYCARD		GeneCard	DisGenet
3938	Glycogen Phosphorylase B	PYGB		GeneCard	
3939	Glycogen Phosphorylase, Muscle Associated	PYGM		GeneCard	
3940	Peptide YY	PYY		GeneCard	
3941	QKI, KH Domain Containing RNA Binding	QKI		GeneCard	
3942	Glutaminyl-TRNA Amidotransferase Subunit QRSL1	QRSL1		GeneCard	
3943	Quiescin Sulfhydryl Oxidase 1	QSOX1		GeneCard	
3944	Queuine TRNA-Ribosyltransferase Catalytic Subunit 1	QTRT1		GeneCard	

3945	RAB20, Member RAS Oncogene Family	RAB20		GeneCard	
3946	RAB22A, Member RAS Oncogene Family	RAB22A		GeneCard	
3947	RAB5A, Member RAS Oncogene Family	RAB5A		GeneCard	
3948	RAB7A, Member RAS Oncogene Family	RAB7A		GeneCard	
3949	RAB9 Effector Protein with Kelch Motifs	RABEPK			DisGenet
3950	Rac Family Small GTPase 1	RAC1		GeneCard	DisGenet
3951	Rac Family Small GTPase 2	RAC2		GeneCard	DisGenet
3952	Receptor For Activated C Kinase 1	RACK1		GeneCard	
3953	RAD21 Cohesin Complex Component	RAD21		GeneCard	
3954	RAD51 Recombinase	RAD51		GeneCard	
3955	RAD51 Associated Protein 1	RAD51AP1		GeneCard	
3956	RAD51 Paralog C	RAD51C		GeneCard	
3957	RAD51 Paralog D	RAD51D		GeneCard	
3958	RAD51L3-RFFL Readthrough	RAD51L3-RFFL		GeneCard	
3959	RAD52 Homolog, DNA Repair Protein	RAD52		GeneCard	
3960	RAD54 Like	RAD54L		GeneCard	
3961	RPA1 Related Single Stranded DNA Binding Protein, X-Linked	RADX		GeneCard	
3962	Retinoic Acid Early Transcript 1E	RAET1E		GeneCard	
3963	Raf-1 Proto-Oncogene, Serine/Threonine Kinase	RAF1		GeneCard	
3964	Recombination Activating 1	RAG1			DisGenet

3965	Recombination Activating 2	RAG2			DisGenet
3966	RAS Like Proto-Oncogene A	RALA		GeneCard	
3967	RalA Binding Protein 1	RALBP1		GeneCard	
3968	RALY RNA Binding Protein Like	RALYL		GeneCard	
3969	Receptor Activity Modifying Protein 3	RAMP3		GeneCard	
3970	RAN, Member RAS Oncogene Family	RAN		GeneCard	
3971	RAP1A, Member Of RAS Oncogene Family	RAP1A		GeneCard	
3972	RAP1B, Member Of RAS Oncogene Family	RAP1B		GeneCard	
3973	RAP2A, Member Of RAS Oncogene Family	RAP2A		GeneCard	
3974	Rap Guanine Nucleotide Exchange Factor 3	RAPGEF3		GeneCard	
3975	Rap Guanine Nucleotide Exchange Factor 5	RAPGEF5		GeneCard	
3976	Retinoic Acid Receptor Alpha	RARA		GeneCard	DisGenet
3977	Retinoic Acid Receptor Beta	RARB		GeneCard	DisGenet
3978	Retinoic Acid Receptor Gamma	RARG		GeneCard	
3979	Retinoic Acid Receptor Responder 1	RARRES1		GeneCard	
3980	Retinoic Acid Receptor Responder 2	RARRES2		GeneCard	DisGenet
3981	Arginyl-TRNA Synthetase 1	RARS1		GeneCard	
3982	Arginyl-TRNA Synthetase 2, Mitochondrial	RARS2		GeneCard	
3983	RAS P21 Protein Activator 1	RASA1		GeneCard	DisGenet
3984	RAS Protein Specific Guanine Nucleotide Releasing Factor 1	RASGRF1			DisGenet

3985	RAS Guanyl Releasing Protein 1	RASGRP1		GeneCard	
3986	RAS Guanyl Releasing Protein 3	RASGRP3		GeneCard	
3987	RB Transcriptional Corepressor 1	RB1		GeneCard	
3988	RB Binding Protein 4, Chromatin Remodeling Factor	RBBP4		GeneCard	
3989	RB Binding Protein 7, Chromatin Remodeling Factor	RBBP7		GeneCard	
3990	RANBP2-Type And C3HC4-Type Zinc Finger Containing 1	RBCK1		GeneCard	
3991	RNA Binding Fox-1 Homolog 1	RBFOX1		GeneCard	
3992	RB Transcriptional Corepressor Like 1	RBL1		GeneCard	
3993	RB Transcriptional Corepressor Like 2	RBL2		GeneCard	
3994	RNA Binding Motif Protein 20	RBM20		GeneCard	
3995	RNA Binding Motif Single Stranded Interacting Protein 3	RBMS3			DisGenet
3996	RNA Binding Motif Protein Y-Linked Family 1 Member A1	RBMY1A1		GeneCard	
3997	Retinol Binding Protein 4	RBP4		GeneCard	DisGenet
3998	Ring-Box 1	RBX1		GeneCard	
3999	Regulator Of Calcineurin 1	RCAN1		GeneCard	DisGenet
4000	RCC1 And BTB Domain Containing Protein 1	RCBTB1		GeneCard	DisGenet
4001	RAS Converting CAAX Endopeptidase 1	RCE1			DisGenet
4002	Reticulocalbin 2	RCN2		GeneCard	DisGenet
4003	Reticulocalbin 3	RCN3		GeneCard	
4004	REC8 Meiotic Recombination Protein	REC8		GeneCard	

4005	Reversion Inducing Cysteine Rich Protein With Kazal Motifs	RECK		GeneCard	
4006	RecQ Like Helicase	RECQL		GeneCard	
4007	RecQ Like Helicase 4	RECQL4		GeneCard	
4008	RecQ Like Helicase 5	RECQL5		GeneCard	
4009	Regenerating Family Member 1 Alpha	REG1A			DisGenet
4010	RELA Proto-Oncogene, NF-KB Subunit	RELA		GeneCard	DisGenet
4011	RELB Proto-Oncogene, NF-KB Subunit	RELB		GeneCard	
4012	Reelin	RELN		GeneCard	DisGenet
4013	Renin	REN		GeneCard	DisGenet
4014	Renin Binding Protein	RENBP			DisGenet
4015	RE1 Silencing Transcription Factor	REST		GeneCard	
4016	Ret Proto-Oncogene	RET		GeneCard	
4017	Resistin	RETN		GeneCard	DisGenet
4018	Resistin Like Beta	RETNLB		GeneCard	DisGenet
4019	Reticulophagy Regulator 1	RETREG1		GeneCard	
4020	Replication Factor C Subunit 1	RFC1			DisGenet
4021	Replication Factor C Subunit 2	RFC2			DisGenet
4022	Replication Factor C Subunit 4	RFC4		GeneCard	
4023	Ret Finger Protein Like 1	RFPL1		GeneCard	
4024	Ret Finger Protein Like 4A	RFPL4A		GeneCard	

402<br>5	RFT1 Homolog	RFT1		GeneCard	
402<br>6	Regulatory Factor X1	RFX1			DisGenet
402<br>7	Regulatory Factor X5	RFX5		GeneCard	DisGenet
402<br>8	Regulator Of Cell Cycle	RGCC		GeneCard	DisGenet
402<br>9	Regulator Of G Protein Signaling 1	RGS1		GeneCard	DisGenet
403<br>0	Regulator Of G Protein Signaling 14	RGS14		GeneCard	
403<br>1	Regulator Of G Protein Signaling 16	RGS16		GeneCard	
403<br>2	Regulator Of G Protein Signaling 2	RGS2		GeneCard	DisGenet
403<br>3	Regulator Of G Protein Signaling 3	RGS3		GeneCard	DisGenet
403<br>4	Regulator Of G Protein Signaling 4	RGS4		GeneCard	
403<br>5	Regulator Of G Protein Signaling 5	RGS5		GeneCard	DisGenet
403<br>6	Regulator Of G Protein Signaling 6	RGS6		GeneCard	
403<br>7	Regulator Of G Protein Signaling 7	RGS7			DisGenet
403<br>8	Regulator Of G Protein Signaling 9	RGS9		GeneCard	
403<br>9	Regulator Of G Protein Signaling 9 Binding Protein	RGS9BP		GeneCard	
404<br>0	Rh Associated Glycoprotein	RHAG		GeneCard	
404<br>1	Rhomboid 5 Homolog 2	RHBDF2		GeneCard	DisGenet
404<br>2	RHEB Pseudogene 1	RHEBP1			DisGenet
404<br>3	Rhodopsin	RHO		GeneCard	
404<br>4	Ras Homolog Family Member A	RHOA		GeneCard	DisGenet

4045	Ras Homolog Family Member B	RHOB		GeneCard	
4046	Rho Related BTB Domain Containing 3	RHOBTB3		GeneCard	
4047	Ras Homolog Family Member D	RHOD		GeneCard	
4048	Ras Homolog Family Member G	RHOG		GeneCard	
4049	Ras Homolog Family Member H	RHOH		GeneCard	
4050	RPTOR Independent Companion Of MTOR Complex 2	RICTOR		GeneCard	
4051	Receptor Interacting Serine/Threonine Kinase 1	RIPK1		GeneCard	
4052	Receptor Interacting Serine/Threonine Kinase 2	RIPK2		GeneCard	DisGenet
4053	Receptor Interacting Serine/Threonine Kinase 3	RIPK3		GeneCard	DisGenet
4054	Ras Like Without CAAX 2	RIT2		GeneCard	
4055	Retinaldehyde Binding Protein 1	RLBP1		GeneCard	
4056	Relaxin 2	RLN2		GeneCard	DisGenet
4057	Regulator Of MON1-CCZ1	RMC1			DisGenet
4058	RNA Component Of Mitochondrial RNA Processing Endoribonuclease	RMRP		GeneCard	
4059	Rhabdomyosarcoma 2 Associated Transcript	RMST		GeneCard	
4060	RNA, 7SL, Cytoplasmic 263, Pseudogene	RN7SL263P			DisGenet
4061	Ribonuclease A Family Member 1, Pancreatic	RNASE1			DisGenet
4062	Ribonuclease A Family Member 3	RNASE3		GeneCard	DisGenet
4063	Ring Finger Protein 111	RNF111		GeneCard	
4064	Ring Finger Protein 113B	RNF113B		GeneCard	

4065	Ring Finger Protein 157	RNF157		GeneCard	
4066	Ring Finger Protein 168	RNF168		GeneCard	
4067	Ring Finger Protein 19A, RBR E3 ubiquitin protein ligase	RNF19A			DisGenet
4068	Ring Finger Protein 213	RNF213		GeneCard	DisGenet
4069	Ring Finger Protein 39	RNF39		GeneCard	
4070	Renalase, FAD Dependent Amine Oxidase	RNLS		GeneCard	
4071	RNA Binding Region (RNP1, RRM) Containing 3	RNPC3		GeneCard	
4072	s-rRNA	RNR1			DisGenet
4073	l-rRNA	RNR2			DisGenet
4074	RNA, U1 Small Nuclear 1	RNU1-1			DisGenet
4075	RNA, U6 Small Nuclear 392, pseudogene	RNU6-392P			DisGenet
4076	RNA, U7 Small Nuclear 51 Pseudogene	RNU7-51P		GeneCard	
4077	Ro60, Y RNA Binding Protein	RO60		GeneCard	
4078	Roundabout Guidance Receptor 2	ROBO2		GeneCard	
4079	Roundabout Guidance Receptor 3	ROBO3			DisGenet
4080	Roundabout Guidance Receptor 4	ROBO4		GeneCard	
4081	Rho Associated Coiled-Coil Containing Protein Kinase 1	ROCK1		GeneCard	DisGenet
4082	Rho Associated Coiled-Coil Containing Protein Kinase 2	ROCK2		GeneCard	DisGenet
4083	Receptor Tyrosine Kinase Like Orphan Receptor 2	ROR2		GeneCard	DisGenet
4084	RAR Related Orphan Receptor A	RORA		GeneCard	DisGenet

4085	ROS Proto-Oncogene 1, Receptor Tyrosine Kinase	ROS1		GeneCard	DisGenet
4086	RP1 Like 1	RP1L1		GeneCard	
4087	Replication Protein A1	RPA1		GeneCard	
4088	Retinitis Pigmentosa GTPase Regulator	RPGR		GeneCard	
4089	RPGRIP1 Like	RPGRIP1L		GeneCard	
4090	Ribosomal Protein L10a Pseudogene 3	RPL10AP3		GeneCard	
4091	Ribosomal Protein L15 Pseudogene 15	RPL15P15		GeneCard	
4092	Ribosomal Protein L18a Pseudogene 13	RPL18AP13		GeneCard	
4093	Ribosomal Protein L21 Pseudogene 103	RPL21P103		GeneCard	
4094	Ribosomal Protein L21 Pseudogene 108	RPL21P108		GeneCard	
4095	Ribosomal Protein L21 Pseudogene 41	RPL21P41		GeneCard	
4096	Ribosomal Protein L21 Pseudogene 42	RPL21P42		GeneCard	
4097	Ribosomal Protein L21 Pseudogene 99	RPL21P99		GeneCard	
4098	Ribosomal Protein L23 Pseudogene 4	RPL23P4		GeneCard	
4099	Ribosomal Protein L28 Pseudogene 3	RPL28P3		GeneCard	
4100	Ribosomal Protein L29 Pseudogene 27	RPL29P27		GeneCard	
4101	Ribosomal Protein L29 Pseudogene 29	RPL29P29		GeneCard	
4102	Ribosomal Protein L31 Pseudogene 23	RPL31P23		GeneCard	
4103	Ribosomal Protein L31 Pseudogene 26	RPL31P26		GeneCard	
4104	Ribosomal Protein L32 Pseudogene 12	RPL32P12		GeneCard	

4105	Ribosomal Protein L34 Pseudogene 26	RPL34P26		GeneCard	
4106	Ribosomal Protein L35a Pseudogene 15	RPL35AP15		GeneCard	
4107	Ribosomal Protein L35a Pseudogene 22	RPL35AP22		GeneCard	
4108	Ribosomal Protein L35 Pseudogene 4	RPL35P4		GeneCard	
4109	Ribosomal Protein L36a Pseudogene 23	RPL36AP23		GeneCard	
4110	Ribosomal Protein L3 Like	RPL3L		GeneCard	
4111	Ribosomal Protein L5 Pseudogene 19	RPL5P19		GeneCard	
4112	Ribosomal Protein L5 Pseudogene 25	RPL5P25		GeneCard	
4113	Ribosomal Protein L7	RPL7		GeneCard	
4114	Ribosomal Protein L7a Pseudogene 57	RPL7AP57		GeneCard	
4115	Ribosomal Protein L7a Pseudogene 58	RPL7AP58		GeneCard	
4116	Ribosomal Protein L9 Pseudogene 21	RPL9P21		GeneCard	
4117	Ribosomal Protein Lateral Stalk Subunit P0	RPLP0		GeneCard	
4118	Ribophorin I	RPN1		GeneCard	
4119	Ribosomal Protein S15 Pseudogene 6	RPS15P6		GeneCard	
4120	Ribosomal Protein S19	RPS19			DisGenet
4121	Ribosomal Protein S25	RPS25		GeneCard	
4122	Ribosomal Protein S26 Pseudogene 57	RPS26P57		GeneCard	
4123	Ribosomal Protein S27a	RPS27A		GeneCard	
4124	RPS27A Pseudogene 2	RPS27AP2		GeneCard	

412<br>5	Ribosomal Protein S27 Pseudogene 1	RPS27P1		GeneCard	
412<br>6	Ribosomal Protein S27 Pseudogene 21	RPS27P21		GeneCard	
412<br>7	Ribosomal Protein S28 Pseudogene 1	RPS28P1		GeneCard	
412<br>8	RPS3A Pseudogene 9	RPS3AP9		GeneCard	
412<br>9	Ribosomal Protein S3 Pseudogene 6	RPS3P6		GeneCard	
413<br>0	Ribosomal Protein S4X Pseudogene 2	RPS4XP2		GeneCard	
413<br>1	Ribosomal Protein S5	RPS5		GeneCard	
413<br>2	Ribosomal Protein S6 Kinase A1	RPS6KA1		GeneCard	DisGenet
413<br>3	Ribosomal Protein S6 Kinase A5	RPS6KA5		GeneCard	
413<br>4	Ribosomal Protein S6 Kinase B1	RPS6KB1		GeneCard	
413<br>5	Ribosomal Protein S6 Pseudogene 20	RPS6P20		GeneCard	
413<br>6	Ribosomal Protein S7 Pseudogene 9	RPS7P9		GeneCard	
413<br>7	Ribosomal Protein SA	RPSA			DisGenet
413<br>8	Regulatory Associated Protein Of MTOR Complex 1	RPTOR		GeneCard	
413<br>9	RNA Pseudouridine Synthase D4	RPUSD4		GeneCard	
414<br>0	Ras Related GTP Binding C	RRAGC		GeneCard	
414<br>1	RAS Related	RRAS		GeneCard	
414<br>2	Ras Responsive Element Binding Protein 1	RREB1		GeneCard	
414<br>3	Ribonucleotide Reductase Catalytic Subunit M1	RRM1		GeneCard	
414<br>4	Ribonucleotide Reductase Regulatory Subunit M2	RRM2		GeneCard	

414 5	Radical S-Adenosyl Methionine Domain Containing 2	RSAD2		GeneCard	DisGenet
414 6	Remodeling And Spacing Factor 1	RSF1		GeneCard	
414 7	R-Spondin 2	RSPO2		GeneCard	
414 8	Rhotekin	RTKN		GeneCard	
414 9	Reticulon 3	RTN3		GeneCard	
415 0	Reticulon 4	RTN4		GeneCard	
415 1	RUNX Family Transcription Factor 2	RUNX2	OMIM	GeneCard	DisGenet
415 2	RUNX Family Transcription Factor 3	RUNX3		GeneCard	
415 3	RuvB Like AAA ATPase 2	RUVBL2		GeneCard	
415 4	Retinoid X Receptor Alpha	RXRA	OMIM	GeneCard	DisGenet
415 5	Retinoid X Receptor Beta	RXRB		GeneCard	
415 6	Retinoid X Receptor Gamma	RXRG		GeneCard	
415 7	Ryanodine Receptor 1	RYR1		GeneCard	
415 8	Ryanodine Receptor 2	RYR2		GeneCard	
415 9	Ryanodine Receptor 3	RYR3		GeneCard	DisGenet
416 0	RYR3 Divergent Transcript	RYR3-DT		GeneCard	
416 1	S100 Calcium Binding Protein A1	S100A1		GeneCard	DisGenet
416 2	S100 Calcium Binding Protein A12	S100A12		GeneCard	DisGenet
416 3	S100 Calcium Binding Protein A4	S100A4		GeneCard	
416 4	S100 Calcium Binding Protein A7	S100A7			DisGenet

4165	S100 Calcium Binding Protein A7A	S100A7A			DisGenet
4166	S100 Calcium Binding Protein A8	S100A8		GeneCard	DisGenet
4167	S100 Calcium Binding Protein A9	S100A9		GeneCard	DisGenet
4168	S100 Calcium Binding Protein B	S100B		GeneCard	DisGenet
4169	Sphingosine-1-Phosphate Receptor 1	S1PR1		GeneCard	DisGenet
4170	Sphingosine-1-Phosphate Receptor 2	S1PR2		GeneCard	
4171	Sphingosine-1-Phosphate Receptor 3	S1PR3		GeneCard	
4172	Serum Amyloid A1	SAA1		GeneCard	DisGenet
4173	Serum Amyloid A2	SAA2		GeneCard	DisGenet
4174	Serum Amyloid A3, Pseudogene	SAA3P		GeneCard	DisGenet
4175	Serum Amyloid A4, Constitutive	SAA4		GeneCard	
4176	Scaffold Attachment Factor B	SAFB		GeneCard	
4177	S-Antigen Visual Arrestin	SAG		GeneCard	
4178	Sterile Alpha Motif Domain Containing 1	SAMD1		GeneCard	DisGenet
4179	Sterile Alpha Motif Domain Containing 4A	SAMD4A			DisGenet
4180	Sterile Alpha Motif Domain Containing 9	SAMD9		GeneCard	DisGenet
4181	SAM And HD Domain Containing Deoxynucleoside Triphosphate Triphosphohydrolase 1	SAMHD1		GeneCard	
4182	SAM Domain, SH3 Domain And Nuclear Localization Signals 1	SAMSN1		GeneCard	DisGenet
4183	Secretion Associated Ras Related GTPase 1B	SAR1B		GeneCard	
4184	Sarcosine Dehydrogenase	SARDH		GeneCard	

4185	Sterile Alpha And TIR Motif Containing 1	SARM1		GeneCard	
4186	SAM And SH3 Domain Containing 1	SASH1		GeneCard	DisGenet
4187	Spermidine/Spermine N1-Acetyltransferase 1	SAT1			DisGenet
4188	SET Binding Factor 1 Pseudogene 1	SBF1P1		GeneCard	
4189	SET Binding Factor 2	SBF2		GeneCard	
4190	Sterol-C5-Desaturase	SC5D		GeneCard	
4191	SREBF Chaperone	SCAP		GeneCard	DisGenet
4192	Scavenger Receptor Class A Member 3	SCARA3		GeneCard	
4193	Scavenger Receptor Class A Member 5	SCARA5		GeneCard	DisGenet
4194	Scavenger receptor class B, member 1 (CD36 antigen-like 1)	SCARB1	OMIM	GeneCard	DisGenet
4195	Scavenger Receptor Class B Member 2	SCARB2		GeneCard	
4196	Scavenger Receptor Class F Member 1	SCARF1		GeneCard	
4197	Scavenger Receptor Family Member Expressed On T Cells 1	SCART1		GeneCard	
4198	Stearoyl-CoA Desaturase	SCD		GeneCard	DisGenet
4199	Stearoyl-CoA Desaturase 5	SCD5		GeneCard	
4200	Secretogranin II	SCG2		GeneCard	DisGenet
4201	Secretogranin III	SCG3		GeneCard	
4202	Secretoglobin Family 1A Member 1	SCGB1A1		GeneCard	
4203	Stem Cell Inhibitory RNA Transcript	SCIRT		GeneCard	
4204	Scm Polycomb Group Protein Like 4	SCML4		GeneCard	

4205	Sodium Voltage-Gated Channel Alpha Subunit 1	SCN1A		GeneCard	
4206	Sodium Voltage-Gated Channel Beta Subunit 4	SCN4B		GeneCard	
4207	Sodium Voltage-Gated Channel Alpha Subunit 5	SCN5A		GeneCard	
4208	Sodium Voltage-Gated Channel Alpha Subunit 7	SCN7A			DisGenet
4209	Sodium Channel Epithelial 1 Subunit Alpha	SCNN1A		GeneCard	
4210	Sodium Channel Epithelial 1 Subunit Beta	SCNN1B		GeneCard	
4211	Sodium Channel Epithelial 1 Subunit Gamma	SCNN1G		GeneCard	
4212	Synthesis Of Cytochrome C Oxidase 2	SCO2		GeneCard	DisGenet
4213	Sterol Carrier Protein 2	SCP2		GeneCard	DisGenet
4214	Signal Peptide, CUB Domain And EGF Like Domain Containing 1	SCUBE1		GeneCard	
4215	Signal Peptide, CUB Domain And EGF Like Domain Containing 2	SCUBE2		GeneCard	
4216	Syndecan 1	SDC1		GeneCard	DisGenet
4217	Syndecan 2	SDC2		GeneCard	
4218	Syndecan 4	SDC4		GeneCard	DisGenet
4219	Syndecan Binding Protein	SDCBP		GeneCard	
4220	Succinate Dehydrogenase Complex Iron Sulfur Subunit B	SDHB		GeneCard	
4221	Succinate Dehydrogenase Complex Subunit C	SDHC		GeneCard	
4222	SEBOX Homeobox	SEBOX		GeneCard	
4223	SEC11 Homolog B, Signal Peptidase Complex Subunit (Pseudogene)	SEC11B		GeneCard	
4224	SEC14 Like Lipid Binding 2	SEC14L2		GeneCard	

4225	SEC23 Interacting Protein	SEC23IP		GeneCard	
4226	Selectin E (endothelial leukocyte adhesion molecule-1)	SELE	OMIM	GeneCard	DisGenet
4227	Selenium Binding Protein 1	SELENBP1			DisGenet
4228	Selenoprotein P	SELENOP		GeneCard	
4229	Selenoprotein S	SELENOS		GeneCard	DisGenet
4230	Selenoprotein T Pseudogene 2	SELENOTP2		GeneCard	
4231	Selectin L	SELL		GeneCard	DisGenet
4232	Selectin P (granulocyte membrane protein, 140kD; antigen CD62)	SELP	OMIM	GeneCard	DisGenet
4233	Selectin P Ligand	SELPLG		GeneCard	DisGenet
4234	Semaphorin 3A	SEMA3A		GeneCard	
4235	Semaphorin 3C	SEMA3C		GeneCard	
4236	Semaphorin 3E	SEMA3E		GeneCard	DisGenet
4237	Semaphorin 3F	SEMA3F		GeneCard	
4238	Semaphorin 4A	SEMA4A		GeneCard	
4239	Semaphorin 4D	SEMA4D		GeneCard	DisGenet
4240	Semaphorin 5A	SEMA5A		GeneCard	
4241	Semaphorin 6A	SEMA6A			DisGenet
4242	Semaphorin 6D	SEMA6D		GeneCard	
4243	Semaphorin 7A (John Milton Hagen Blood Group)	SEMA7A		GeneCard	DisGenet
4244	Smooth Muscle And Endothelial Cell Enriched Migration/Differentiation-Associated LncRNA	SENCR		GeneCard	

4245	SUMO Specific Peptidase 2	SENP2		GeneCard	DisGenet
4246	SUMO Peptidase Family Member, NEDD8 Specific	SENP8		GeneCard	
4247	Septin 4	SEPTIN4		GeneCard	
4248	Serpin Family A Member 1	SERPINA1		GeneCard	DisGenet
4249	Serpin Family A Member 10	SERPINA10		GeneCard	
4250	Serpin Family A Member 12	SERPINA12		GeneCard	DisGenet
4251	Serpin Family A Member 3	SERPINA3		GeneCard	DisGenet
4252	Serpin Family A Member 4	SERPINA4		GeneCard	
4253	Serpin Family A Member 5	SERPINA5		GeneCard	
4254	Serpin Family A Member 9	SERPINA9		GeneCard	DisGenet
4255	Serpin Family B Member 1	SERPINB1		GeneCard	
4256	Serpin Family B Member 2	SERPINB2		GeneCard	
4257	Serpin Family B Member 9	SERPINB9		GeneCard	
4258	Serpin Family C Member 1	SERPINC1		GeneCard	
4259	Serpin Family D Member 1	SERPIND1		GeneCard	DisGenet
4260	Serpin Family E Member 1	SERPINE1	OMIM	GeneCard	DisGenet
4261	Serpin Family E Member 2	SERPINE2		GeneCard	
4262	Serpin Family F Member 1	SERPINF1		GeneCard	DisGenet
4263	Serpin Family F Member 2	SERPINF2		GeneCard	DisGenet
4264	Serpin Family G Member 1	SERPING1		GeneCard	DisGenet

4265	Serpin Family H Member 1	SERPINH1		GeneCard	
4266	Serpin Family I Member 1	SERPINI1		GeneCard	
4267	Sestrin 2	SESN2		GeneCard	DisGenet
4268	SET Domain Containing 2, Histone Lysine Methyltransferase	SETD2			DisGenet
4269	SET Domain Bifurcated Histone Lysine Methyltransferase 1	SETDB1		GeneCard	
4270	Seizure Related 6 Homolog Like	SEZ6L		GeneCard	
4271	Splicing Factor 3a Subunit 1	SF3A1		GeneCard	
4272	Splicing Factor 3a Subunit 2	SF3A2		GeneCard	
4273	Secreted Frizzled Related Protein 4	SFRP4		GeneCard	DisGenet
4274	Secreted Frizzled Related Protein 5	SFRP5		GeneCard	DisGenet
4275	Surfactant Protein A1	SFTPA1			DisGenet
4276	Surfactant Protein A2	SFTPA2			DisGenet
4277	Surfactant Protein B	SFTPB		GeneCard	
4278	Surfactant Protein D	SFTPD	OMIM	GeneCard	DisGenet
4279	Sarcoglycan Alpha	SGCA		GeneCard	
4280	Sarcoglycan Beta	SGCB		GeneCard	DisGenet
4281	Sarcoglycan Delta	SGCD		GeneCard	
4282	Sarcoglycan Zeta	SGCZ		GeneCard	
4283	SH3GL Interacting Endocytic Adaptor 1	SGIP1		GeneCard	
4284	Serum/Glucocorticoid Regulated Kinase 1	SGK1		GeneCard	

4285	Sphingomyelin Synthase 1	SGMS1		GeneCard	DisGenet
4286	Sphingomyelin Synthase 2	SGMS2		GeneCard	DisGenet
4287	Shugoshin 2	SGO2		GeneCard	
4288	Sphingosine-1-Phosphate Lyase 1	SGPL1		GeneCard	DisGenet
4289	Small G Protein Signaling Modulator 3	SGSM3			DisGenet
4290	SH2B Adaptor Protein 1	SH2B1		GeneCard	
4291	SH2B Adaptor Protein 3	SH2B3		GeneCard	DisGenet
4292	SH2 Domain Containing 1A	SH2D1A		GeneCard	
4293	SH2 Domain Containing 2A	SH2D2A		GeneCard	
4294	SH3 Domain Containing 21	SH3D21		GeneCard	
4295	SH3 Domain Containing GRB2 Like 1, Endophilin A2	SH3GL1		GeneCard	
4296	SH3 Domain Containing GRB2 Like 2, Endophilin A1	SH3GL2		GeneCard	
4297	SHANK Associated RH Domain Interactor	SHARPIN		GeneCard	
4298	SH2 Domain Containing Adaptor Protein B	SHB		GeneCard	
4299	Sex Hormone Binding Globulin	SHBG		GeneCard	DisGenet
4300	SHC Adaptor Protein 1	SHC1		GeneCard	DisGenet
4301	Sonic Hedgehog Signaling Molecule	SHH		GeneCard	
4302	Serine Hydroxymethyltransferase 1	SHMT1		GeneCard	
4303	Short Stature Homeobox	SHOX		GeneCard	
4304	Sedoheptulokinase	SHPK		GeneCard	

4305	Sialic Acid Acetylesterase	SIAE		GeneCard	
4306	Single Ig And TIR Domain Containing	SIGIRR			DisGenet
4307	Sialic Acid Binding Ig Like Lectin 1	SIGLEC1		GeneCard	DisGenet
4308	sialic acid binding Ig like lectin 9	SIGLEC9			DisGenet
4309	Salt Inducible Kinase 2	SIK2		GeneCard	
4310	SIN3 Transcription Regulator Family Member B	SIN3B		GeneCard	
4311	Signal Regulatory Protein Alpha	SIRPA		GeneCard	DisGenet
4312	Sirtuin, S. cerevisiae, homolog 1	SIRT1	OMIM	GeneCard	DisGenet
4313	SIRT1 Antisense RNA	SIRT1-AS			DisGenet
4314	Sirtuin 2	SIRT2		GeneCard	DisGenet
4315	Sirtuin 3	SIRT3		GeneCard	DisGenet
4316	Sirtuin 4	SIRT4		GeneCard	
4317	Sirtuin 5	SIRT5		GeneCard	
4318	Sirtuin 6	SIRT6		GeneCard	DisGenet
4319	Sirtuin 7	SIRT7		GeneCard	DisGenet
4320	S-Phase Kinase Associated Protein 1	SKP1		GeneCard	
4321	S-Phase Kinase Associated Protein 2	SKP2		GeneCard	DisGenet
4322	Signaling Lymphocytic Activation Molecule Family Member 1	SLAMF1		GeneCard	
4323	SLAM Family Member 6	SLAMF6		GeneCard	
4324	SLAM Family Member 7	SLAMF7		GeneCard	DisGenet

4325	Solute Carrier Family 10 Member 1	SLC10A1		GeneCard	
4326	Solute Carrier Family 10 Member 2	SLC10A2		GeneCard	DisGenet
4327	Solute Carrier Family 11 Member 1	SLC11A1		GeneCard	
4328	Solute Carrier Family 11 Member 2	SLC11A2		GeneCard	
4329	Solute Carrier Family 12 Member 1	SLC12A1		GeneCard	
4330	Solute Carrier Family 12 Member 3	SLC12A3		GeneCard	DisGenet
4331	Solute Carrier Family 12 Member 4	SLC12A4		GeneCard	
4332	SLC12A5 And MMP9 Antisense RNA 1	SLC12A5-AS1		GeneCard	
4333	Solute Carrier Family 15 Member 4	SLC15A4		GeneCard	
4334	Solute Carrier Family 16 Member 3	SLC16A3		GeneCard	DisGenet
4335	Solute Carrier Family 16 Member 8	SLC16A8			DisGenet
4336	Solute Carrier Family 17 Member 1	SLC17A1		GeneCard	
4337	Solute Carrier Family 17 Member 3	SLC17A3		GeneCard	
4338	Solute Carrier Family 17 Member 4	SLC17A4		GeneCard	DisGenet
4339	Solute Carrier Family 17 Member 5	SLC17A5		GeneCard	DisGenet
4340	Solute Carrier Family 18 Member A1	SLC18A1		GeneCard	
4341	Solute Carrier Family 19 Member 1	SLC19A1		GeneCard	
4342	Solute Carrier Family 1 Member 1	SLC1A1		GeneCard	
4343	Solute Carrier Family 1 Member 4	SLC1A4		GeneCard	
4344	Solute Carrier Family 20 Member 1	SLC20A1		GeneCard	

434 5	Solute Carrier Family 20 Member 2	SLC20A2		GeneCard	
434 6	Solute Carrier Family 22 Member 1	SLC22A1		GeneCard	DisGenet
434 7	Solute Carrier Family 22 Member 11	SLC22A11		GeneCard	
434 8	Solute Carrier Family 22 Member 12	SLC22A12		GeneCard	
434 9	Solute Carrier Family 22 Member 18	SLC22A18			DisGenet
435 0	Solute Carrier Family 22 Member 4	SLC22A4		GeneCard	
435 1	Solute Carrier Family 22 Member 5	SLC22A5		GeneCard	
435 2	Solute Carrier Family 23 Member 2	SLC23A2		GeneCard	
435 3	Solute Carrier Family 24 Member 3	SLC24A3		GeneCard	
435 4	Solute Carrier Family 25 Member 1	SLC25A1		GeneCard	
435 5	Solute Carrier Family 25 Member 17	SLC25A17		GeneCard	
435 6	Solute Carrier Family 25 Member 20	SLC25A20			DisGenet
435 7	Solute Carrier Family 25 Member 22	SLC25A22		GeneCard	
435 8	Solute Carrier Family 25 Member 24	SLC25A24		GeneCard	
435 9	Solute Carrier Family 25 Member 4	SLC25A4		GeneCard	
436 0	Solute Carrier Family 25 Member 5	SLC25A5		GeneCard	
436 1	Solute Carrier Family 26 Member 4	SLC26A4			DisGenet
436 2	Solute Carrier Family 26 Member 7	SLC26A7		GeneCard	
436 3	Solute Carrier Family 27 Member 1	SLC27A1		GeneCard	
436 4	Solute Carrier Family 27 Member 6	SLC27A6		GeneCard	

4365	Solute Carrier Family 29 Member 1 (Augustine Blood Group)	SLC29A1		GeneCard	
4366	Solute Carrier Family 29 Member 2	SLC29A2		GeneCard	
4367	Solute Carrier Family 2 Member 1	SLC2A1		GeneCard	DisGenet
4368	Solute Carrier Family 2 Member 10	SLC2A10		GeneCard	
4369	Solute Carrier Family 2 Member 13	SLC2A13		GeneCard	
4370	Solute Carrier Family 2 Member 2	SLC2A2		GeneCard	
4371	Solute Carrier Family 2 Member 3	SLC2A3		GeneCard	
4372	Solute Carrier Family 2 Member 4	SLC2A4		GeneCard	
4373	Solute Carrier Family 2 Member 4 Regulator	SLC2A4RG			DisGenet
4374	Solute Carrier Family 2 Member 5	SLC2A5		GeneCard	
4375	Solute Carrier Family 2 Member 6	SLC2A6			DisGenet
4376	Solute Carrier Family 2 Member 9	SLC2A9	OMIM	GeneCard	DisGenet
4377	Solute Carrier Family 30 Member 8	SLC30A8		GeneCard	
4378	Solute Carrier Family 31 Member 1	SLC31A1		GeneCard	
4379	Solute Carrier Family 33 Member 1	SLC33A1			DisGenet
4380	Solute Carrier Family 34 Member 1	SLC34A1		GeneCard	
4381	Solute Carrier Family 35 Member A1	SLC35A1			DisGenet
4382	Solute Carrier Family 39 Member 1	SLC39A1			DisGenet
4383	Solute Carrier Family 39 Member 2	SLC39A2		GeneCard	
4384	Solute Carrier Family 39 Member 8	SLC39A8			DisGenet

4385	Solute Carrier Family 3 Member 2	SLC3A2			DisGenet
4386	Solute Carrier Family 40 Member 1	SLC40A1		GeneCard	DisGenet
4387	Solute Carrier Family 44 Member 3	SLC44A3		GeneCard	
4388	Solute Carrier Family 45 Member 3	SLC45A3		GeneCard	
4389	Solute Carrier Family 46 Member 1	SLC46A1		GeneCard	
4390	Solute Carrier Family 4 Member 1 (Diego Blood Group)	SLC4A1		GeneCard	
4391	Solute Carrier Family 4 Member 5	SLC4A5		GeneCard	
4392	Solute Carrier Family 52 Member 1	SLC52A1			DisGenet
4393	Solute Carrier Family 52 Member 2	SLC52A2			DisGenet
4394	Solute Carrier Family 5 Member 1	SLC5A1		GeneCard	
4395	Solute Carrier Family 5 Member 10	SLC5A10		GeneCard	DisGenet
4396	Solute Carrier Family 5 Member 11	SLC5A11		GeneCard	
4397	Solute Carrier Family 5 Member 2	SLC5A2			DisGenet
4398	Solute Carrier Family 5 Member 7	SLC5A7		GeneCard	
4399	Solute Carrier Family 6 Member 13	SLC6A13		GeneCard	
4400	Solute Carrier Family 6 Member 2	SLC6A2		GeneCard	
4401	Solute Carrier Family 6 Member 3	SLC6A3		GeneCard	
4402	Solute Carrier Family 6 Member 4	SLC6A4		GeneCard	DisGenet
4403	Solute Carrier Family 6 Member 8	SLC6A8			DisGenet
4404	Solute Carrier Family 7 Member 5	SLC7A5			DisGenet

4405	Solute Carrier Family 8 Member A1	SLC8A1		GeneCard	DisGenet
4406	Solute Carrier Family 9 Member A1	SLC9A1		GeneCard	DisGenet
4407	SLC9A3 Regulator 2	SLC9A3R2		GeneCard	
4408	Solute Carrier Organic Anion Transporter Family Member 5A1	SLCO5A1		GeneCard	
4409	Solute Carrier Organic Anion Transporter Family Member 6A1	SLCO6A1			DisGenet
4410	Systemic Lupus Erythematosus, Susceptibility To, 12	SLEB12		GeneCard	
4411	Systemic Lupus Erythematosus, Susceptibility To, 13	SLEB13		GeneCard	
4412	Systemic Lupus Erythematosus, Susceptibility To, 14	SLEB14		GeneCard	
4413	Systemic Lupus Erthematosus, Susceptibility To, 15	SLEB15		GeneCard	
4414	Systemic Lupus Erythematosus Susceptibility 3	SLEB3		GeneCard	
4415	Systemic Lupus Erythematosus, Susceptibility To, 4	SLEB4		GeneCard	
4416	Systemic Lupus Erythematosus, Susceptibility To, 5	SLEB5		GeneCard	
4417	Systemic Lupus Erythematosus, Susceptibility To, 7	SLEB7		GeneCard	
4418	Systemic Lupus Erythematosus, Susceptibility To, 8	SLEB8		GeneCard	
4419	Systemic Lupus Erythematosus With Hemolytic Anemia 1	SLEH1		GeneCard	
4420	Systemic Lupus Erythematosus With Nephritis 1	SLEN1		GeneCard	
4421	Systemic Lupus Erythematosus With Nephritis 2	SLEN2		GeneCard	
4422	Systemic Lupus Erythematosus With Nephritis 3	SLEN3		GeneCard	
4423	Slit Guidance Ligand 2	SLIT2		GeneCard	DisGenet
4424	SLIT And NTRK Like Family Member 3	SLITRK3		GeneCard	

4425	Secretory Leukocyte Peptidase Inhibitor	SLPI		GeneCard	DisGenet
4426	Secreted LY6/PLAUR Domain Containing 1	SLURP1		GeneCard	
4427	SMAD Family Member 1	SMAD1		GeneCard	DisGenet
4428	SMAD Family Member 2	SMAD2		GeneCard	
4429	SMAD Family Member 3	SMAD3		GeneCard	DisGenet
4430	SMAD Family Member 4	SMAD4		GeneCard	
4431	SMAD Family Member 5	SMAD5		GeneCard	
4432	SMAD Family Member 6	SMAD6		GeneCard	
4433	SMAD Family Member 7	SMAD7		GeneCard	DisGenet
4434	SWI/SNF Related, Matrix Associated, Actin Dependent Regulator Of Chromatin, Subfamily A, Member 1	SMARCA1		GeneCard	
4435	SWI/SNF Related, Matrix Associated, Actin Dependent Regulator Of Chromatin, Subfamily A, Member 2	SMARCA2		GeneCard	
4436	SWI/SNF Related, Matrix Associated, Actin Dependent Regulator Of Chromatin, Subfamily A, Member 4	SMARCA4		GeneCard	DisGenet
4437	SWI/SNF Related, Matrix Associated, Actin Dependent Regulator Of Chromatin, Subfamily A, Member 5	SMARCA5		GeneCard	
4438	SWI/SNF Related, Matrix Associated, Actin Dependent Regulator Of Chromatin, Subfamily A Like 1	SMARCAL1	OMIM	GeneCard	
4439	Structural Maintenance Of Chromosomes 1B	SMC1B		GeneCard	
4440	Sperm Mitochondria Associated Cysteine Rich Protein	SMCP			DisGenet
4441	Smooth Muscle Induced LncRNA, Enhancer Of Proliferation	SMILR		GeneCard	
4442	Small Integral Membrane Protein 35	SMIM35		GeneCard	
4443	Survival Of Motor Neuron 1, Telomeric	SMN1			DisGenet
4444	Survival Of Motor Neuron 2, Centromeric	SMN2			DisGenet

4445	Smoothened, Frizzled Class Receptor	SMO		GeneCard	
4446	SPARC Related Modular Calcium Binding 1	SMOC1		GeneCard	
4447	SPARC Related Modular Calcium Binding 2	SMOC2		GeneCard	
4448	Spermine Oxidase	SMOX			DisGenet
4449	Sphingomyelin Phosphodiesterase 1	SMPD1		GeneCard	DisGenet
4450	Sphingomyelin Phosphodiesterase 2	SMPD2		GeneCard	DisGenet
4451	Sphingomyelin Phosphodiesterase 3	SMPD3		GeneCard	DisGenet
4452	Sphingomyelin Phosphodiesterase Acid Like 3A	SMPDL3A		GeneCard	
4453	Spermine Synthase	SMS			DisGenet
4454	Smoothelin	SMTN		GeneCard	
4455	single-strand-selective monofunctional uracil-DNA glycosylase 1	SMUG1			DisGenet
4456	Snail Family Transcriptional Repressor 1	SNAI1		GeneCard	DisGenet
4457	Snail Family Transcriptional Repressor 2	SNAI2			DisGenet
4458	Synuclein Alpha	SNCA		GeneCard	
4459	Synuclein Alpha Interacting Protein	SNCAIP		GeneCard	
4460	Synuclein Beta	SNCB		GeneCard	
4461	Synuclein Gamma	SNCG		GeneCard	
4462	SNF8 Subunit Of ESCRT-II	SNF8		GeneCard	DisGenet
4463	Small Nucleolar RNA Host Gene 12	SNHG12		GeneCard	
4464	Small Nucleolar RNA Host Gene 16	SNHG16		GeneCard	DisGenet

4465	Small Nucleolar RNA Host Gene 7	SNHG7		GeneCard	
4466	Small Nucleolar RNA Host Gene 8	SNHG8		GeneCard	
4467	Stannin	SNN		GeneCard	
4468	Small Nuclear Ribonucleoprotein U1 Subunit 70	SNRNP70		GeneCard	
4469	Small Nuclear Ribonucleoprotein Polypeptides B And B1	SNRPB		GeneCard	
4470	Small Nuclear Ribonucleoprotein Polypeptide B2	SNRPB2		GeneCard	DisGenet
4471	Small Nuclear Ribonucleoprotein D1 Polypeptide	SNRPD1		GeneCard	
4472	Small Nuclear Ribonucleoprotein D3 Polypeptide	SNRPD3		GeneCard	
4473	Small Nuclear Ribonucleoprotein Polypeptide E	SNRPE		GeneCard	
4474	Small Nuclear Ribonucleoprotein Polypeptide N	SNRPN		GeneCard	
4475	Syntrophin Alpha 1	SNTA1		GeneCard	
4476	Sorting Nexin 19	SNX19			DisGenet
4477	Sorting Nexin 10	SNX10		GeneCard	
4478	Sorting Nexin 16	SNX16		GeneCard	
4479	Sorting Nexin 17	SNX17		GeneCard	
4480	Sorting Nexin 19	SNX19		GeneCard	
4481	Sterol O-acyltransferase 1 (acyl-Coenzyme A: cholesterol acyltransferase 1)	SOAT1	OMIM	GeneCard	DisGenet
4482	Sterol O-acyltransferase 2	SOAT2	OMIM	GeneCard	DisGenet
4483	Suppressor Of Cytokine Signaling 1	SOCS1		GeneCard	DisGenet
4484	Suppressor Of Cytokine Signaling 3	SOCS3		GeneCard	DisGenet

4485	Suppressor Of Cytokine Signaling 5	SOCS5		GeneCard	
4486	Suppressor Of Cytokine Signaling 6	SOCS6		GeneCard	
4487	Superoxide Dismutase 1	SOD1		GeneCard	DisGenet
4488	Superoxide Dismutase 2	SOD2		GeneCard	DisGenet
4489	Superoxide Dismutase 3	SOD3		GeneCard	DisGenet
4490	Spermatogenesis And Oogenesis Specific Basic Helix-Loop-Helix 1	SOHLH1	OMIM	GeneCard	
4491	Spermatogenesis And Oogenesis Specific Basic Helix-Loop-Helix 2	SOHLH2		GeneCard	
4492	Sorbin And SH3 Domain Containing 3	SORBS3		GeneCard	DisGenet
4493	Sortilin Related VPS10 Domain Containing Receptor 1	SORCS1		GeneCard	
4494	Sortilin Related VPS10 Domain Containing Receptor 2	SORCS2		GeneCard	
4495	Sorbitol Dehydrogenase	SORD		GeneCard	
4496	Sortilin Related Receptor 1	SORL1	OMIM	GeneCard	DisGenet
4497	Sortilin 1	SORT1		GeneCard	DisGenet
4498	SOS Ras/Rac Guanine Nucleotide Exchange Factor 1	SOS1		GeneCard	DisGenet
4499	Sclerostin	SOST		GeneCard	
4500	SRY-Box Transcription Factor 18	SOX18		GeneCard	DisGenet
4501	SRY-Box Transcription Factor 6	SOX6		GeneCard	DisGenet
4502	SRY-Box Transcription Factor 7	SOX7		GeneCard	
4503	SRY-Box Transcription Factor 9	SOX9		GeneCard	
4504	Sp1 Transcription Factor	SP1		GeneCard	DisGenet

4505	SP110 Nuclear Body Protein	SP110		GeneCard	
4506	Sp3 Transcription Factor	SP3		GeneCard	
4507	Sp6 transcription factor	SP6			DisGenet
4508	Sp7 Transcription Factor	SP7		GeneCard	
4509	Small Regulatory Polypeptide Of Amino Acid Response	SPAAR		GeneCard	
4510	Secreted Protein Acidic And Cysteine Rich	SPARC		GeneCard	DisGenet
4511	SPARC Like 1	SPARCL1		GeneCard	
4512	Spermatogenesis Associated 6 Like	SPATA6L		GeneCard	
4513	Spermatogenesis Associated 7	SPATA7		GeneCard	
4514	SPC24 Component Of NDC80 Kinetochore Complex	SPC24		GeneCard	
4515	Striated Muscle Enriched Protein Kinase	SPEG		GeneCard	
4516	SPG21 Abhydrolase Domain Containing, Maspardin	SPG21		GeneCard	
4517	SPG7 Matrix AAA Peptidase Subunit, Paraplegin	SPG7		GeneCard	DisGenet
4518	Sphingosine Kinase 1	SPHK1		GeneCard	DisGenet
4519	Sphingosine Kinase 2	SPHK2			DisGenet
4520	SPHK1 Interactor, AKAP Domain Containing	SPHKAP		GeneCard	
4521	Spi-1 Proto-Oncogene	SPI1		GeneCard	DisGenet
4522	Scaffold Protein Involved In DNA Repair	SPIDR		GeneCard	
4523	Serine Peptidase Inhibitor Kazal Type 1	SPINK1		GeneCard	
4524	SPO11 Initiator Of Meiotic Double Stranded Breaks	SPO11		GeneCard	

4525	Spondin 2	SPON2			DisGenet
4526	Secreted phosphoprotein-1 (osteopontin, bone sialoprotein)	SPP1	OMIM	GeneCard	DisGenet
4527	Signal Peptide Peptidase Like 2A	SPPL2A		GeneCard	
4528	Signal Peptide Peptidase Like 2B	SPPL2B		GeneCard	
4529	Sepiapterin Reductase	SPR		GeneCard	
4530	Small Proline Rich Protein 3	SPRR3		GeneCard	
4531	Sprouty RTK Signaling Antagonist 1	SPRY1		GeneCard	DisGenet
4532	Sprouty RTK Signaling Antagonist 2	SPRY2		GeneCard	
4533	Sprouty RTK Signaling Antagonist 4	SPRY4		GeneCard	
4534	SPRY Domain Containing 4	SPRYD4		GeneCard	
4535	Spectrin Beta, Erythrocytic	SPTB		GeneCard	
4536	Serine Palmitoyltransferase Long Chain Base Subunit 1	SPTLC1		GeneCard	
4537	Spexin Hormone	SPX		GeneCard	DisGenet
4538	Spermatogenic Leucine Zipper 1	SPZ1			DisGenet
4539	Squalene Epoxidase	SQLE		GeneCard	
4540	Sulfide Quinone Oxidoreductase	SQOR		GeneCard	
4541	Sequestosome 1	SQSTM1		GeneCard	DisGenet
4542	Steroid Receptor RNA Activator 1	SRA1		GeneCard	
4543	SRC Proto-Oncogene, Non-Receptor Tyrosine Kinase	SRC		GeneCard	DisGenet
4544	Steroid 5 Alpha-Reductase 1	SRD5A1		GeneCard	

4545	Steroid 5 Alpha-Reductase 2	SRD5A2		GeneCard	
4546	Sterol Regulatory Element Binding Transcription Factor 1	SREBF1		GeneCard	DisGenet
4547	Sterol Regulatory Element Binding Transcription Factor 2	SREBF2		GeneCard	DisGenet
4548	Serum Response Factor	SRF		GeneCard	
4549	Serum Response Factor Binding Protein 1	SRFBP1		GeneCard	
4550	Serglycin	SRGN		GeneCard	
4551	Sorcin	SRI			DisGenet
4552	Sorcin Pseudogene 1	SRIP1		GeneCard	
4553	Serine/Arginine repetitive matrix 2	SRRM2			DisGenet
4554	Serine and Arginine Rich Splicing Factor 1	SRSF1			DisGenet
4555	Serine and Arginine Rich Splicing Factor 2	SRSF2		GeneCard	DisGenet
4556	Serine and Arginine Rich Splicing Factor 3	SRSF3		GeneCard	
4557	Serine and Arginine Rich Splicing Factor 5	SRSF5		GeneCard	
4558	Serine and Arginine Rich Splicing Factor 7	SRSF7		GeneCard	
4559	Sulfiredoxin 1	SRXN1		GeneCard	
4560	Sex Determining Region Y	SRY		GeneCard	DisGenet
4561	Small RNA Binding Exonuclease Protection Factor La	SSB		GeneCard	
4562	Single Stranded DNA Binding Protein 1	SSBP1			DisGenet
4563	Single Stranded DNA Binding Protein 3	SSBP3		GeneCard	
4564	Slingshot Protein Phosphatase 1	SSH1		GeneCard	

4565	Structure Specific Recognition Protein 1	SSRP1		GeneCard	DisGenet
4566	Somatostatin	SST		GeneCard	DisGenet
4567	Somatostatin Receptor 2	SSTR2		GeneCard	DisGenet
4568	Somatostatin Receptor 4	SSTR4			DisGenet
4569	ST13 Hsp70 Interacting Protein	ST13		GeneCard	
4570	ST14 Transmembrane Serine Protease Matriptase	ST14		GeneCard	DisGenet
4571	Suppression Of Tumorigenicity 2	ST2		GeneCard	DisGenet
4572	ST3 Beta-Galactoside Alpha-2,3-Sialyltransferase 1	ST3GAL1		GeneCard	
4573	ST3 Beta-Galactoside Alpha-2,3-Sialyltransferase 4	ST3GAL4		GeneCard	DisGenet
4574	ST3 Beta-Galactoside Alpha-2,3-Sialyltransferase 5	ST3GAL5		GeneCard	
4575	ST6 Beta-Galactoside Alpha-2,6-Sialyltransferase 1	ST6GAL1		GeneCard	DisGenet
4576	ST8 Alpha-N-Acetyl-Neuraminide Alpha-2,8-Sialyltransferase 1	ST8SIA1		GeneCard	DisGenet
4577	ST8 Alpha-N-Acetyl-Neuraminide Alpha-2,8-Sialyltransferase 4	ST8SIA4		GeneCard	
4578	Stabilin 1	STAB1		GeneCard	DisGenet
4579	Stabilin 2	STAB2		GeneCard	
4580	Stromal Antigen 3	STAG3		GeneCard	
4581	Stromal Antigen 3-Like 4 (Pseudogene)	STAG3L4		GeneCard	
4582	STAM Binding Protein	STAMBP		GeneCard	
4583	Signal Transducing Adaptor Family Member 1	STAP1		GeneCard	
4584	Steroidogenic Acute Regulatory Protein	STAR		GeneCard	DisGenet

4585	StAR Related Lipid Transfer Domain Containing 7	STARD7		GeneCard	
4586	Steroidogenic Acute Regulatory Protein Pseudogene 1	STARP1		GeneCard	
4587	Signal Transducer And Activator Of Transcription 1	STAT1		GeneCard	DisGenet
4588	Signal Transducer And Activator Of Transcription 3	STAT3		GeneCard	DisGenet
4589	Signal Transducer And Activator Of Transcription 4	STAT4		GeneCard	DisGenet
4590	Signal Transducer And Activator Of Transcription 5A	STAT5A		GeneCard	
4591	Signal Transducer And Activator Of Transcription 5B	STAT5B		GeneCard	
4592	Signal Transducer And Activator Of Transcription 6	STAT6		GeneCard	DisGenet
4593	Staufen Double-Stranded RNA Binding Protein 1	STAU1		GeneCard	
4594	Stanniocalcin 1	STC1		GeneCard	
4595	Stanniocalcin 2	STC2		GeneCard	DisGenet
4596	STEAP Family Member 1	STEAP1		GeneCard	
4597	STEAP Family Member 1B	STEAP1B		GeneCard	
4598	STEAP1B Antisense RNA 1	STEAP1B-AS1		GeneCard	
4599	STEAP2 Antisense RNA 1	STEAP2-AS1		GeneCard	
4600	STEAP4 Metalloreductase	STEAP4		GeneCard	DisGenet
4601	Stromal Interaction Molecule 1	STIM1		GeneCard	DisGenet
4602	Serine/Threonine Kinase 11	STK11		GeneCard	DisGenet
4603	Serine/Threonine Kinase 25	STK25			DisGenet
4604	Serine/Threonine Kinase 38	STK38		GeneCard	

4605	Serine/Threonine Kinase 38 Like	STK38L		GeneCard	
4606	Serine/Threonine Kinase 40	STK40		GeneCard	
4607	Stathmin 1	STMN1		GeneCard	
4608	Stimulated By Retinoic Acid 8	STRA8		GeneCard	
4609	Striatin Interacting Protein 2	STRIP2			DisGenet
4610	Steroid Sulfatase	STS		GeneCard	DisGenet
4611	STIP1 Homology And U-Box Containing Protein 1	STUB1		GeneCard	DisGenet
4612	Syntaxin Binding Protein 3	STXBP3		GeneCard	
4613	Syntaxin Binding Protein 5	STXBP5		GeneCard	
4614	SUB1 Regulator Of Transcription	SUB1		GeneCard	
4615	Succinate-Coa Ligase ADP-Forming Subunit Beta	SUCLA2			DisGenet
4616	SUCLA2 Pseudogene 2	SUCLA2P2		GeneCard	
4617	Succinate-CoA Ligase GDP/ADP-Forming Subunit Alpha	SUCLG1		GeneCard	
4618	Succinate Receptor 1	SUCNR1		GeneCard	DisGenet
4619	Sulfatase 2	SULF2		GeneCard	
4620	Sulfotransferase Family 1A Member 3	SULT1A3		GeneCard	
4621	Sulfotransferase Family 1E Member 1	SULT1E1		GeneCard	
4622	Sulfotransferase Family 2A Member 1	SULT2A1		GeneCard	DisGenet
4623	Sulfotransferase Family 2B Member 1	SULT2B1		GeneCard	
4624	Small Ubiquitin Like Modifier 1	SUMO1		GeneCard	DisGenet

4625	Sad1 And UNC84 Domain Containing 2	SUN2		GeneCard	
4626	SPT3 Homolog, SAGA And STAGA Complex Component	SUPT3H		GeneCard	
4627	SURF1 Cytochrome C Oxidase Assembly Factor	SURF1		GeneCard	
4628	Surfeit 2	SURF2		GeneCard	
4629	Surfeit 4	SURF4		GeneCard	
4630	Sushi Domain Containing 2	SUSD2		GeneCard	
4631	SUV39H1 Histone Lysine Methyltransferase	SUV39H1		GeneCard	
4632	SUV39H2 Histone Lysine Methyltransferase	SUV39H2		GeneCard	
4633	Sushi, Von Willebrand Factor Type A, EGF And Pentraxin Domain Containing 1	SVEP1		GeneCard	
4634	Synaptonemal Complex Central Element Protein 1	SYCE1		GeneCard	
4635	Synaptonemal Complex Protein 3	SYCP3		GeneCard	
4636	Spleen Associated Tyrosine Kinase	SYK		GeneCard	DisGenet
4637	Synapsin II	SYN2		GeneCard	
4638	Spectrin Repeat Containing Nuclear Envelope Protein 1	SYNE1		GeneCard	
4639	Spectrin Repeat Containing Nuclear Envelope Protein 2	SYNE2		GeneCard	
4640	Spectrin Repeat Containing Nuclear Envelope Family Member 3	SYNE3		GeneCard	
4641	Synemin	SYNM		GeneCard	
4642	Synaptopodin 2	SYNPO2		GeneCard	
4643	Synaptopodin 2 Like	SYNPO2L		GeneCard	
4644	Synaptophysin	SYP		GeneCard	

4645	Synaptotagmin 1	SYT1			DisGenet
4646	Synaptotagmin 15	SYT15		GeneCard	
4647	Synaptotagmin 7	SYT7		GeneCard	
4648	Synoviolin 1	SYVN1		GeneCard	
4649	TGF-Beta Activated Kinase 1 (MAP3K7) Binding Protein 2	TAB2		GeneCard	
4650	Tachykinin Precursor 1	TAC1			DisGenet
4651	Tachykinin Receptor 1	TACR1		GeneCard	
4652	Transcriptional Adaptor 1	TADA1			DisGenet
4653	TATA-Box Binding Protein Associated Factor 3	TAF3			DisGenet
4654	TATA-Box Binding Protein Associated Factor 7	TAF7		GeneCard	
4655	TAFA Chemokine Like Family Member 5	TAFA5		GeneCard	
4656	Tafazzin, Phospholipid-Lysophospholipid Transacylase	TAFAZZIN		GeneCard	
4657	Transgelin	TAGLN		GeneCard	DisGenet
4658	Transgelin 2	TAGLN2		GeneCard	
4659	Myeloproliferative syndrome, transient	TAM			DisGenet
4660	Tetratricopeptide Repeat, Ankyrin Repeat And Coiled-Coil Containing 1	TANC1		GeneCard	
4661	TRAF Family Member Associated NFKB Activator	TANK		GeneCard	
4662	Transporter 2, ATP Binding Cassette Subfamily B Member	TAP2		GeneCard	
4663	TAR DNA Binding Protein	TARDBP		GeneCard	
4664	TCR Gamma Alternate Reading Frame Protein	TARP		GeneCard	

4665	Taste 2 Receptor Member 50	TAS2R50		GeneCard	
4666	TLR Adaptor Interacting With Endolysosomal SLC15A4	TASL		GeneCard	
4667	Tyrosine Aminotransferase	TAT		GeneCard	
4668	Tafazzin	TAZ			DisGenet
4669	TBC1 Domain Family Member 1	TBC1D1		GeneCard	
4670	TBC1 Domain Family Member 4	TBC1D4		GeneCard	
4671	Tubulin Folding Cofactor A Pseudogene 1	TBCAP1		GeneCard	
4672	TANK Binding Kinase 1	TBK1		GeneCard	
4673	TBL1X/Y Related 1	TBL1XR1		GeneCard	
4674	TATA-Box Binding Protein Like 2	TBPL2			DisGenet
4675	Transforming Growth Factor Beta Regulator 1	TBRG1		GeneCard	
4676	T-Box Transcription Factor 18	TBX18		GeneCard	DisGenet
4677	T-Box Transcription Factor 20	TBX20		GeneCard	DisGenet
4678	T-Box Transcription Factor 21	TBX21		GeneCard	
4679	T-Box Transcription Factor 4	TBX4		GeneCard	
4680	T-Box Transcription Factor 5	TBX5		GeneCard	
4681	Thromboxane A2 Receptor	TBXA2R		GeneCard	DisGenet
4682	Thromboxane A Synthase 1	TBXAS1		GeneCard	DisGenet
4683	T-Box Transcription Factor T	TBXT		GeneCard	
4684	Titin-Cap	TCAP		GeneCard	

4685	Transcription Elongation Factor A1	TCEA1		GeneCard	
4686	Transcription Elongation Factor A3	TCEA3		GeneCard	
4687	Transcription Factor 15	TCF15			DisGenet
4688	Transcription Factor 21	TCF21		GeneCard	DisGenet
4689	Transcription Factor 4	TCF4		GeneCard	
4690	Transcription Factor 7	TCF7		GeneCard	
4691	Transcription Factor 7 Like 2	TCF7L2		GeneCard	DisGenet
4692	T Cell Immune Regulator 1, ATPase H+ Transporting V0 Subunit A3	TCIRG1		GeneCard	DisGenet
4693	Transcobalamin 1	TCN1		GeneCard	
4694	Transcobalamin 2	TCN2		GeneCard	
4695	T-Complex 1	TCP1		GeneCard	
4696	T-Complex 11 Like 1	TCP11L1		GeneCard	
4697	Tryptophan 2,3-Dioxygenase	TDO2			DisGenet
4698	Tudor Domain Containing 9	TDRD9			DisGenet
4699	TEA Domain Transcription Factor 1	TEAD1		GeneCard	
4700	TEK Receptor Tyrosine Kinase	TEK		GeneCard	DisGenet
4701	Teneurin Transmembrane Protein 2	TENM2		GeneCard	
4702	Terminal Nucleotidyltransferase 4B	TENT4B		GeneCard	
4703	Telomerase RNA Component	TERC		GeneCard	
4704	Telomeric Repeat Binding Factor 1	TERF1		GeneCard	

470<br>5	Telomeric Repeat Binding Factor 2	TERF2		GeneCard	
470<br>6	TERF2 Interacting Protein	TERF2IP		GeneCard	
470<br>7	Telomerase Reverse Transcriptase	TERT		GeneCard	
470<br>8	Testin LIM Domain Protein	TES		GeneCard	
470<br>9	Tescalcin	TESC			DisGenet
471<br>0	Tet Methylcytosine Dioxygenase 2	TET2	OMIM	GeneCard	DisGenet
471<br>1	Tet Methylcytosine Dioxygenase 3	TET3			DisGenet
471<br>2	Transferrin	TF		GeneCard	
471<br>3	Transcription Factor A, Mitochondrial	TFAM		GeneCard	DisGenet
471<br>4	Transcription Factor AP-2 Alpha	TFAP2A		GeneCard	
471<br>5	Transcription Factor AP-2 Beta	TFAP2B		GeneCard	
471<br>6	Transcription Factor CP2	TFCP2		GeneCard	
471<br>7	Transcription Factor EB	TFEB		GeneCard	DisGenet
471<br>8	Trefoil Factor 3	TFF3		GeneCard	
471<br>9	Tissue Factor Pathway Inhibitor	TFPI		GeneCard	DisGenet
472<br>0	Tissue Factor Pathway Inhibitor 2	TFPI2		GeneCard	DisGenet
472<br>1	TCF3 Fusion Partner	TFPT		GeneCard	
472<br>2	Transferrin Receptor	TFRC		GeneCard	DisGenet
472<br>3	Thyroglobulin	TG		GeneCard	
472<br>4	Transforming Growth Factor Alpha	TGFA		GeneCard	

4725	Transforming Growth Factor Beta 1	TGFB1		GeneCard	DisGenet
4726	Transforming Growth Factor Beta 1 Induced Transcript 1	TGFB1I1		GeneCard	DisGenet
4727	Transforming Growth Factor Beta 2	TGFB2		GeneCard	DisGenet
4728	Transforming Growth Factor Beta 3	TGFB3		GeneCard	
4729	Transforming Growth Factor Beta Induced	TGFBI		GeneCard	DisGenet
4730	Transforming Growth Factor Beta Receptor 1	TGFBR1		GeneCard	
4731	Transforming Growth Factor Beta Receptor 2	TGFBR2		GeneCard	
4732	Transforming Growth Factor Beta Receptor 3	TGFBR3		GeneCard	DisGenet
4733	TGFB Induced Factor Homeobox 1	TGIF1		GeneCard	
4734	Transglutaminase 2	TGM2		GeneCard	DisGenet
4735	Trimethylguanosine Synthase 1	TGS1		GeneCard	
4736	Tyrosine Hydroxylase	TH		GeneCard	
4737	Thrombomodulin	THBD		GeneCard	DisGenet
4738	Thrombospondin 1	THBS1		GeneCard	DisGenet
4739	Thrombospondin 2	THBS2		GeneCard	
4740	Thrombospondin 4	THBS4		GeneCard	DisGenet
4741	Thioesterase Superfamily Member 4	THEM4		GeneCard	
4742	Thymoma	THM			DisGenet
4743	THO Complex 5	THOC5		GeneCard	
4744	Thrombopoietin	THPO		GeneCard	

4745	Thyroid Hormone Receptor Beta	THRB		GeneCard	
4746	Thrombospondin Type 1 Domain Containing 1	THSD1	OMIM	GeneCard	
4747	Thrombospondin Type 1 Domain Containing 4	THSD4		GeneCard	
4748	Thrombospondin Type 1 Domain Containing 7A	THSD7A		GeneCard	
4749	Thy-1 Cell Surface Antigen	THY1			DisGenet
4750	TIA1 Cytotoxic Granule Associated RNA Binding Protein	TIA1		GeneCard	
4751	TIAM Rac1 Associated GEF 1	TIAM1		GeneCard	
4752	TIR Domain Containing Adaptor Molecule 1	TICAM1		GeneCard	
4753	Toll Like Receptor Adaptor Molecule 2	TICAM2			DisGenet
4754	Tyrosine Kinase With Immunoglobulin Like And EGF Like Domains 1	TIE1		GeneCard	DisGenet
4755	Timeless Circadian Regulator	TIMELESS			DisGenet
4756	TIMP Metallopeptidase Inhibitor 1	TIMP1		GeneCard	DisGenet
4757	TIMP Metallopeptidase Inhibitor 2	TIMP2		GeneCard	
4758	TIMP Metallopeptidase Inhibitor 3	TIMP3		GeneCard	DisGenet
4759	TIMP Metallopeptidase Inhibitor 4	TIMP4		GeneCard	
4760	TIMELESS Interacting Protein	TIPIN		GeneCard	
4761	Tight Junction Protein 1	TJP1		GeneCard	
4762	Transketolase	TKT		GeneCard	
4763	Tousled Like Kinase 1	TLK1		GeneCard	
4764	Tolloid Like 1	TLL1		GeneCard	

4765	Talin 1	TLN1		GeneCard	
4766	Talin 2	TLN2		GeneCard	
4767	Toll Like Receptor 1	TLR1		GeneCard	DisGenet
4768	Toll Like Receptor 10	TLR10		GeneCard	
4769	Toll Like Receptor 2	TLR2	OMIM	GeneCard	DisGenet
4770	Toll Like Receptor 3	TLR3		GeneCard	DisGenet
4771	Toll Like Receptor 4	TLR4	OMIM	GeneCard	DisGenet
4772	Toll Like Receptor 5	TLR5		GeneCard	DisGenet
4773	Toll Like Receptor 6	TLR6		GeneCard	DisGenet
4774	Toll Like Receptor 7	TLR7		GeneCard	DisGenet
4775	Toll Like Receptor 8	TLR8		GeneCard	DisGenet
4776	Toll Like Receptor 9	TLR9		GeneCard	DisGenet
4777	TLX1 Neighbor	TLX1NB			DisGenet
4778	Transmembrane 4 L Six Family Member 19	TM4SF19		GeneCard	
4779	Transmembrane 4 L Six Family Member 5	TM4SF5		GeneCard	
4780	Transmembrane 6 Superfamily Member 2	TM6SF2		GeneCard	DisGenet
4781	Transmembrane 7 Superfamily Member 2	TM7SF2			DisGenet
4782	Transmembrane BAX Inhibitor Motif Containing 1	TMBIM1	OMIM	GeneCard	
4783	Transmembrane And Coiled-Coil Domains 1	TMCO1		GeneCard	
4784	Transmembrane And Coiled-Coil Domains 5A	TMCO5A		GeneCard	

4785	Transmembrane And Coiled-Coil Domains 5B (Pseudogene)	TMCO5B		GeneCard	
4786	Transmembrane p24 Trafficking Protein 7	TMED7			DisGenet
4787	TMED7-TICAM2 readthrough	TMED7-TICAM2			DisGenet
4788	Transmembrane Protein 106B	TMEM106B		GeneCard	
4789	Transmembrane Protein 132B	TMEM132B		GeneCard	
4790	Transmembrane Protein 150B	TMEM150B		GeneCard	
4791	Transmembrane Protein 161B	TMEM161B		GeneCard	
4792	Transmembrane Protein 170A	TMEM170A		GeneCard	
4793	Transmembrane Protein 18	TMEM18		GeneCard	DisGenet
4794	Transmembrane Protein 199	TMEM199		GeneCard	
4795	Transmembrane Protein 248 Pseudogene 1	TMEM248P1		GeneCard	
4796	Transmembrane Protein 258	TMEM258		GeneCard	
4797	Transmembrane Protein 43	TMEM43		GeneCard	
4798	Transmembrane Protein 59	TMEM59		GeneCard	
4799	Transmembrane Protein 68	TMEM68		GeneCard	
4800	Transmembrane Protein 97	TMEM97		GeneCard	
4801	Transmembrane Protein 98	TMEM98			DisGenet
4802	Thymopoietin	TMPO		GeneCard	
4803	Transmembrane Serine Protease 6	TMPRSS6		GeneCard	
4804	Thymosin Beta 10	TMSB10		GeneCard	DisGenet

480<br>5	Thymosin Beta 4 X-Linked	TMSB4X		GeneCard	
480<br>6	Transmembrane O-Mannosyltransferase Targeting Cadherins 1	TMTC1		GeneCard	
480<br>7	Tenascin C	TNC		GeneCard	DisGenet
480<br>8	Tumor Necrosis Factor	TNF		GeneCard	DisGenet
480<br>9	TNF Alpha Induced Protein 1	TNFAIP1		GeneCard	
481<br>0	TNF Alpha Induced Protein 3	TNFAIP3	OMIM	GeneCard	
481<br>1	TNF Alpha Induced Protein 6	TNFAIP6		GeneCard	DisGenet
481<br>2	TNF Alpha Induced Protein 8	TNFAIP8	OMIM	GeneCard	
481<br>3	TNF Alpha Induced Protein 8 Like 1	TNFAIP8L1		GeneCard	DisGenet
481<br>4	TNF Alpha Induced Protein 8 Like 2	TNFAIP8L2			DisGenet
481<br>5	TNF Receptor Superfamily Member 10a	TNFRSF10A		GeneCard	
481<br>6	TNF Receptor Superfamily Member 10b	TNFRSF10B		GeneCard	
481<br>7	TNF Receptor Superfamily Member 10c	TNFRSF10C		GeneCard	
481<br>8	TNF Receptor Superfamily Member 11a	TNFRSF11A		GeneCard	DisGenet
481<br>9	TNF Receptor Superfamily Member 11b	TNFRSF11B	OMIM	GeneCard	DisGenet
482<br>0	TNF Receptor Superfamily Member 12A	TNFRSF12A		GeneCard	DisGenet
482<br>1	TNF Receptor Superfamily Member 13B	TNFRSF13B		GeneCard	
482<br>2	TNF Receptor Superfamily Member 13C	TNFRSF13C		GeneCard	DisGenet
482<br>3	TNF Receptor Superfamily Member 14	TNFRSF14		GeneCard	DisGenet
482<br>4	TNF Receptor Superfamily Member 17	TNFRSF17		GeneCard	

4825	TNF Receptor Superfamily Member 18	TNFRSF18		GeneCard	
4826	TNF Receptor Superfamily Member 1A	TNFRSF1A	OMIM	GeneCard	DisGenet
4827	TNF Receptor Superfamily Member 1B	TNFRSF1B		GeneCard	DisGenet
4828	TNF Receptor Superfamily Member 25	TNFRSF25		GeneCard	DisGenet
4829	TNF Receptor Superfamily Member 4	TNFRSF4		GeneCard	DisGenet
4830	TNF Receptor Superfamily Member 6b	TNFRSF6B		GeneCard	DisGenet
4831	TNF Receptor Superfamily Member 9	TNFRSF9		GeneCard	DisGenet
4832	TNF Superfamily Member 10	TNFSF10		GeneCard	DisGenet
4833	TNF Superfamily Member 11	TNFSF11		GeneCard	DisGenet
4834	TNF Superfamily Member 12	TNFSF12		GeneCard	DisGenet
4835	TNFSF12-TNFSF13 readthrough	TNFSF12-TNFSF13			DisGenet
4836	TNF Superfamily Member 13	TNFSF13		GeneCard	DisGenet
4837	TNF Superfamily Member 13b	TNFSF13B		GeneCard	DisGenet
4838	TNF Superfamily Member 14	TNFSF14		GeneCard	DisGenet
4839	TNF Superfamily Member 15	TNFSF15		GeneCard	DisGenet
4840	TNF Superfamily Member 4	TNFSF4	OMIM	GeneCard	DisGenet
4841	TNF Superfamily Member 9	TNFSF9		GeneCard	DisGenet
4842	TNFAIP3 Interacting Protein 1	TNIP1		GeneCard	
4843	TNFAIP3 Interacting Protein 3	TNIP3		GeneCard	
4844	Tankyrase	TNKS		GeneCard	

4845	Troponin C1, Slow Skeletal And Cardiac Type	TNNC1		GeneCard	
4846	Troponin C2, Fast Skeletal Type	TNNC2		GeneCard	
4847	Troponin I1, Slow Skeletal Type	TNNI1		GeneCard	
4848	Troponin I2, Fast Skeletal Type	TNNI2		GeneCard	
4849	Troponin I3, Cardiac Type	TNNI3		GeneCard	DisGenet
4850	TNNI3 Interacting Kinase	TNNI3K		GeneCard	
4851	Troponin T1, Slow Skeletal Type	TNNT1		GeneCard	
4852	Troponin T2, Cardiac Type	TNNT2		GeneCard	DisGenet
4853	Transportin 1	TNPO1		GeneCard	
4854	Tenascin R	TNR		GeneCard	
4855	Tenascin XA (Pseudogene)	TNXA		GeneCard	
4856	Tenascin XB	TNXB		GeneCard	
4857	Toll Interacting Protein	TOLLIP		GeneCard	DisGenet
4858	Translocase Of Outer Mitochondrial Membrane 40	TOMM40		GeneCard	
4859	Tonsoku Like, DNA Repair Protein	TONSL		GeneCard	
4860	DNA Topoisomerase I	TOP1		GeneCard	
4861	TOP1 Binding Arginine/Serine Rich Protein, E3 Ubiquitin Ligase	TOPORS		GeneCard	
4862	Torsin Family 1 Member A	TOR1A		GeneCard	
4863	Torsin Family 2 Member A	TOR2A		GeneCard	DisGenet
4864	Thymocyte Selection Associated High Mobility Group Box	TOX		GeneCard	

4865	TOX High Mobility Group Box Family Member 3	TOX3		GeneCard	
4866	Tumor Protein P53	TP53		GeneCard	DisGenet
4867	Tumor Protein P53 Pathway Corepressor 1	TP53COR1		GeneCard	DisGenet
4868	Tumor Protein P63	TP63		GeneCard	DisGenet
4869	Tumor Protein P73	TP73		GeneCard	DisGenet
4870	TP73 Antisense RNA 1	TP73-AS1		GeneCard	
4871	Tumor Protein D52	TPD52		GeneCard	
4872	TPD52 Like 3	TPD52L3		GeneCard	
4873	Tryptophan Hydroxylase 1	TPH1		GeneCard	DisGenet
4874	Triosephosphate Isomerase 1	TPI1			DisGenet
4875	Tropomyosin 1	TPM1		GeneCard	
4876	Tropomyosin 2	TPM2		GeneCard	DisGenet
4877	Thyroid Peroxidase	TPO		GeneCard	DisGenet
4878	Transmembrane Protein Adipocyte Associated 1	TPRA1		GeneCard	
4879	Tryptase Alpha/Beta 1	TPSAB1		GeneCard	
4880	Tyrosylprotein Sulfotransferase 1	TPST1		GeneCard	
4881	Tyrosylprotein Sulfotransferase 2	TPST2		GeneCard	
4882	Tumor Protein, Translationally-Controlled 1	TPT1			DisGenet
4883	T Cell Receptor Alpha Locus	TRA		GeneCard	
4884	TNFRSF1A Associated Via Death Domain	TRADD		GeneCard	

4885	TNF Receptor Associated Factor 1	TRAF1		GeneCard	DisGenet
4886	TNF Receptor Associated Factor 2	TRAF2		GeneCard	DisGenet
4887	TNF Receptor Associated Factor 3	TRAF3		GeneCard	
4888	TRAF3 Interacting Protein 2	TRAF3IP2			DisGenet
4889	TNF Receptor Associated Factor 4	TRAF4		GeneCard	DisGenet
4890	TNF Receptor Associated Factor 6	TRAF6		GeneCard	DisGenet
4891	TNF Receptor Associated Factor 7	TRAF7		GeneCard	
4892	Trafficking Kinesin Protein 2	TRAK2		GeneCard	
4893	Translocation Associated Membrane Protein 1	TRAM1		GeneCard	DisGenet
4894	T Cell Receptor Associated Transmembrane Adaptor 1	TRAT1			DisGenet
4895	T Cell Receptor Beta Locus	TRB		GeneCard	
4896	T cell receptor beta variable 20/OR9-2 (non-functional)	TRBV20OR9-2			DisGenet
4897	T Cell Receptor Delta Locus	TRD		GeneCard	
4898	Triadin	TRDN		GeneCard	
4899	Trehalase	TREH		GeneCard	
4900	Triggering Receptor Expressed On Myeloid Cells 1	TREM1		GeneCard	DisGenet
4901	Triggering Receptor Expressed On Myeloid Cells 2	TREM2		GeneCard	
4902	Triggering Receptor Expressed On Myeloid Cells Like 1	TREML1		GeneCard	
4903	Triggering Receptor Expressed On Myeloid Cells Like 4	TREML4		GeneCard	
4904	Three Prime Repair Exonuclease 1	TREX1		GeneCard	

490 5	T Cell Receptor Gamma Locus	TRG		GeneCard	
490 6	Thyrotropin Releasing Hormone	TRH		GeneCard	
490 7	Thyrotropin Releasing Hormone Degrading Enzyme	TRHDE		GeneCard	
490 8	Tribbles Pseudokinase 1	TRIB1		GeneCard	
490 9	Tribbles Pseudokinase 2	TRIB2		GeneCard	
491 0	Tribbles Pseudokinase 3	TRIB3		GeneCard	DisGenet
491 1	Tripartite Motif Containing 10	TRIM10		GeneCard	
491 2	Tripartite Motif Containing 11	TRIM11		GeneCard	
491 3	Tripartite Motif Containing 13	TRIM13			DisGenet
491 4	Tripartite Motif Containing 14	TRIM14		GeneCard	DisGenet
491 5	Tripartite Motif Containing 15	TRIM15		GeneCard	
491 6	Tripartite Motif Containing 21	TRIM21		GeneCard	DisGenet
491 7	Tripartite Motif Containing 24	TRIM24		GeneCard	
491 8	Tripartite Motif Containing 26	TRIM26		GeneCard	
491 9	Tripartite Motif Containing 27	TRIM27		GeneCard	
492 0	Tripartite Motif Containing 28	TRIM28		GeneCard	DisGenet
492 1	Tripartite Motif Containing 31	TRIM31		GeneCard	
492 2	Tripartite Motif Containing 40	TRIM40		GeneCard	
492 3	Tripartite Motif Containing 63	TRIM63		GeneCard	
492 4	Tripartite Motif Containing 68	TRIM68		GeneCard	

4925	Tripartite Motif Containing 7	TRIM7		GeneCard	
4926	Tripartite Motif Containing 9	TRIM9		GeneCard	
4927	tRNA-Leu (anticodon TAG) 1-1	TRL-TAG1-1			DisGenet
4928	tRNA	TRNL1			DisGenet
4929	tRNA	TRNL2			DisGenet
4930	Transient Receptor Potential Cation Channel Subfamily A Member 1	TRPA1		GeneCard	DisGenet
4931	Transient Receptor Potential Cation Channel Subfamily C Member 1	TRPC1		GeneCard	DisGenet
4932	Transient Receptor Potential Cation Channel Subfamily C Member 3	TRPC3		GeneCard	DisGenet
4933	Transient Receptor Potential Cation Channel Subfamily C Member 4	TRPC4		GeneCard	
4934	Transient Receptor Potential Cation Channel Subfamily C Member 4 Associated Protein	TRPC4AP		GeneCard	
4935	Transient Receptor Potential Cation Channel Subfamily C Member 5	TRPC5		GeneCard	DisGenet
4936	Transient Receptor Potential Cation Channel Subfamily C Member 6	TRPC6		GeneCard	DisGenet
4937	Transient Receptor Potential Cation Channel Subfamily M Member 1	TRPM1		GeneCard	
4938	Transient Receptor Potential Cation Channel Subfamily M Member 2	TRPM2		GeneCard	
4939	Transient Receptor Potential Cation Channel Subfamily M Member 3	TRPM3		GeneCard	
4940	Transient Receptor Potential Cation Channel Subfamily M Member 6	TRPM6		GeneCard	DisGenet
4941	Transient Receptor Potential Cation Channel Subfamily M Member 7	TRPM7		GeneCard	
4942	Transcriptional Repressor GATA Binding 1	TRPS1		GeneCard	
4943	Transient Receptor Potential Cation Channel Subfamily V Member 1	TRPV1		GeneCard	DisGenet
4944	Transient Receptor Potential Cation Channel Subfamily V Member 2	TRPV2		GeneCard	

494<br>5	Transient Receptor Potential Cation Channel Subfamily V Member 4	TRPV4		GeneCard	DisGenet
494<br>6	Transient Receptor Potential Cation Channel Subfamily V Member 5	TRPV5			DisGenet
494<br>7	Transient Receptor Potential Cation Channel Subfamily V Member 6	TRPV6		GeneCard	
494<br>8	Testis Expressed Basic Protein 1	TSBP1		GeneCard	
494<br>9	TSC Complex Subunit 1	TSC1		GeneCard	DisGenet
495<br>0	TSC Complex Subunit 2	TSC2		GeneCard	
495<br>1	Ts Translation Elongation Factor, Mitochondrial	TSFM		GeneCard	
495<br>2	Thyroid Stimulating Hormone Subunit Beta	TSHB		GeneCard	
495<br>3	Tsukushi, Small Leucine Rich Proteoglycan	TSKU		GeneCard	
495<br>4	Thymic Stromal Lymphopoietin	TSLP		GeneCard	DisGenet
495<br>5	Tetraspanin 16	TSPAN16		GeneCard	
495<br>6	Tetraspanin 2	TSPAN2		GeneCard	
495<br>7	Tetraspanin 31	TSPAN31			DisGenet
495<br>8	Tetraspanin 32	TSPAN32		GeneCard	
495<br>9	Tetraspanin 6	TSPAN6		GeneCard	
496<br>0	Translocator Protein	TSPO		GeneCard	DisGenet
496<br>1	TSPOAP1, SUPT4H1 And RNF43 Antisense RNA 1	TSPOAP1-AS1		GeneCard	
496<br>2	Tetratricopeptide Repeat Domain 27	TTC27		GeneCard	
496<br>3	Tetratricopeptide Repeat Domain 39B	TTC39B	OMIM	GeneCard	
496<br>4	Transcription Termination Factor 2	TTF2		GeneCard	

4965	Tubulin Tyrosine Ligase Like 11	TTLL11		GeneCard	
4966	Titin	TTN		GeneCard	
4967	TTN Antisense RNA 1	TTN-AS1		GeneCard	
4968	Alpha Tocopherol Transfer Protein	TTPA	OMIM	GeneCard	DisGenet
4969	Transthyretin	TTR		GeneCard	DisGenet
4970	Tubulin Alpha 1b	TUBA1B		GeneCard	
4971	Tubulin Alpha 4a	TUBA4A		GeneCard	
4972	Tubulin Beta Class I	TUBB		GeneCard	
4973	Tubulin Beta 4A Class IVa	TUBB4A		GeneCard	
4974	Tu Translation Elongation Factor, Mitochondrial	TUFM			DisGenet
4975	Taurine Up-Regulated 1	TUG1		GeneCard	DisGenet
4976	Tumor Suppressor Candidate 1	TUSC1		GeneCard	
4977	Tumor Suppressor Candidate 3	TUSC3		GeneCard	
4978	Twist Family BHLH Transcription Factor 1	TWIST1		GeneCard	DisGenet
4979	Twinkle MtDNA Helicase	TWNK		GeneCard	
4980	Thioredoxin	TXN		GeneCard	DisGenet
4981	Thioredoxin Domain-Containing Protein 5	TXNDC5	OMIM		
4982	Thioredoxin Interacting Protein	TXNIP		GeneCard	DisGenet
4983	Thioredoxin Like 4B	TXNL4B		GeneCard	
4984	Thioredoxin Reductase 1	TXNRD1		GeneCard	DisGenet

498 5	Thioredoxin Reductase 2	TXNRD2		GeneCard	DisGenet
498 6	Tyrosine Kinase 2	TYK2		GeneCard	
498 7	Thymidine Phosphorylase	TYMP		GeneCard	DisGenet
498 8	Thymidylate Synthetase	TYMS		GeneCard	
498 9	TYRO3 Protein Tyrosine Kinase	TYRO3		GeneCard	
499 0	Transmembrane Immune Signaling Adaptor TYROBP	TYROBP		GeneCard	DisGenet
499 1	Ubiquitin Like Modifier Activating Enzyme 1	UBA1		GeneCard	
499 2	Ubiquitin A-52 Residue Ribosomal Protein Fusion Product 1	UBA52		GeneCard	
499 3	Ubiquitin A-52 Residue Ribosomal Protein Fusion Product 1 Pseudogene 6	UBA52P6		GeneCard	
499 4	UBA Domain Containing 2	UBAC2		GeneCard	
499 5	Ubiquitin Conjugating Enzyme E2 D2	UBE2D2		GeneCard	
499 6	Ubiquitin Conjugating Enzyme E2 I	UBE2I		GeneCard	
499 7	Ubiquitin Conjugating Enzyme E2 K	UBE2K		GeneCard	
499 8	Ubiquitin Conjugating Enzyme E2 L3	UBE2L3		GeneCard	
499 9	UBE2Q2 Pseudogene 1	UBE2Q2P1		GeneCard	
500 0	Ubiquitin Conjugating Enzyme E2 Z	UBE2Z		GeneCard	DisGenet
500 1	Ubiquitination Factor E4A	UBE4A		GeneCard	
500 2	UbiA Prenyltransferase Domain Containing 1	UBIAD1		GeneCard	
500 3	Ubiquitin Like Domain Containing CTD Phosphatase 1	UBLCP1		GeneCard	
500 4	Ubiquilin 2	UBQLN2		GeneCard	

500<br>5	UBX Domain Protein 7	UBXN7		GeneCard	
500<br>6	Urothelial Cancer Associated 1	UCA1		GeneCard	DisGenet
500<br>7	Ubiquitin C-Terminal Hydrolase L1	UCHL1		GeneCard	
500<br>8	Upper Zone Of Growth Plate And Cartilage Matrix Associated	UCMA		GeneCard	
500<br>9	Urocortin	UCN		GeneCard	
501<br>0	Uncoupling Protein 1	UCP1	OMIM	GeneCard	DisGenet
501<br>1	Uncoupling Protein 2	UCP2		GeneCard	DisGenet
501<br>2	Uncoupling Protein 3	UCP3		GeneCard	
501<br>3	Ubiquitin Fold Modifier 1	UFM1		GeneCard	DisGenet
501<br>4	UDP-Glucose Ceramide Glucosyltransferase	UGCG		GeneCard	
501<br>5	UDP-Glucose 6-Dehydrogenase	UGDH		GeneCard	
501<br>6	UDP Glucuronosyltransferase Family 1 Member A Complex Locus	UGT1A		GeneCard	
501<br>7	UDP Glucuronosyltransferase Family 1 Member A1	UGT1A1		GeneCard	
501<br>8	UDP Glucuronosyltransferase Family 1 Member A10	UGT1A10		GeneCard	
501<br>9	UDP Glucuronosyltransferase Family 1 Member A12, Pseudogene	UGT1A12P		GeneCard	
502<br>0	UDP Glucuronosyltransferase Family 1 Member A3	UGT1A3		GeneCard	
502<br>1	UDP Glucuronosyltransferase Family 1 Member A4	UGT1A4		GeneCard	
502<br>2	UDP Glucuronosyltransferase Family 1 Member A5	UGT1A5		GeneCard	
502<br>3	UDP Glucuronosyltransferase Family 1 Member A6	UGT1A6		GeneCard	
502<br>4	UDP Glucuronosyltransferase Family 1 Member A7	UGT1A7		GeneCard	

5025	UDP Glucuronosyltransferase Family 1 Member A8	UGT1A8		GeneCard	
5026	UDP Glucuronosyltransferase Family 1 Member A9	UGT1A9		GeneCard	
5027	UDP Glycosyltransferase Family 3 Member A1	UGT3A1		GeneCard	
5028	Ubiquitin Like With PHD And Ring Finger Domains 2	UHRF2		GeneCard	
5029	Ubiquitin Interaction Motif Containing 1	UIMC1		GeneCard	
5030	Unc-51 Like Kinase 4	ULK4		GeneCard	
5031	Uromodulin	UMOD		GeneCard	DisGenet
5032	Unc-13 Homolog C	UNC13C		GeneCard	
5033	Unc-5 Netrin Receptor B	UNC5B		GeneCard	DisGenet
5034	UNC Homeobox	UNCX		GeneCard	
5035	Uracil-DNA Glycosylase Pseudogene 1	UNGP1		GeneCard	
5036	URB1 Ribosome Biogenesis Homolog	URB1		GeneCard	
5037	URB2 Ribosome Biogenesis Homolog	URB2		GeneCard	
5038	Upstream Transcription Factor 1	USF1	OMIM	GeneCard	DisGenet
5039	Upstream Transcription Factor 2, C-Fos Interacting	USF2		GeneCard	
5040	USO1 Vesicle Transport Factor	USO1		GeneCard	
5041	Ubiquitin Specific Peptidase 10	USP10		GeneCard	
5042	Ubiquitin Specific Peptidase 14	USP14		GeneCard	
5043	Ubiquitin Specific Peptidase 15	USP15		GeneCard	
5044	Ubiquitin Specific Peptidase 17 Like Family Member 9, Pseudogene	USP17L9P		GeneCard	

5045	Ubiquitin Specific Peptidase 20	USP20		GeneCard	DisGenet
5046	Ubiquitin Specific Peptidase 24	USP24		GeneCard	
5047	Ubiquitin Specific Peptidase 3	USP3		GeneCard	
5048	Ubiquitin Specific Peptidase 7	USP7		GeneCard	
5049	UTP20 Small Subunit Processome Component	UTP20		GeneCard	
5050	Utrophin	UTRN		GeneCard	DisGenet
5051	Urotensin 2	UTS2		GeneCard	DisGenet
5052	Urotensin 2B	UTS2B		GeneCard	DisGenet
5053	Urotensin 2 Receptor	UTS2R		GeneCard	
5054	UV Stimulated Scaffold Protein A	UVSSA		GeneCard	
5055	Vesicle Associated Membrane Protein 3	VAMP3		GeneCard	
5056	Vesicle Associated Membrane Protein 8	VAMP8		GeneCard	DisGenet
5057	Valyl-tRNA Synthetase 1	VARS1			DisGenet
5058	Vasohibin 1	VASH1		GeneCard	
5059	Vasodilator Stimulated Phosphoprotein	VASP		GeneCard	
5060	Vav Guanine Nucleotide Exchange Factor 1	VAV1		GeneCard	DisGenet
5061	Vav Guanine Nucleotide Exchange Factor 2	VAV2		GeneCard	DisGenet
5062	Vav Guanine Nucleotide Exchange Factor 3	VAV3			DisGenet
5063	Vascular cell adhesion molecule-1	VCAM1	OMIM	GeneCard	DisGenet
5064	Versican	VCAN		GeneCard	DisGenet

506 5	Vinculin	VCL		GeneCard	DisGenet
506 6	Valosin Containing Protein	VCP		GeneCard	
506 7	Vitamin D Receptor	VDR		GeneCard	DisGenet
506 8	Vascular Endothelial Growth Factor A	VEGFA	OMIM	GeneCard	DisGenet
506 9	Vascular Endothelial Growth Factor B	VEGFB		GeneCard	
507 0	Vascular Endothelial Growth Factor C	VEGFC		GeneCard	
507 1	Vascular Endothelial Growth Factor D	VEGFD		GeneCard	DisGenet
507 2	VENT Homeobox Pseudogene 2	VENTXP2		GeneCard	
507 3	Ventricular Zone Expressed PH Domain Containing 1	VEPH1		GeneCard	
507 4	Von Hippel-Lindau Tumor Suppressor	VHL		GeneCard	
507 5	Vimentin	VIM		GeneCard	DisGenet
507 6	Vasoactive Intestinal Peptide	VIP		GeneCard	DisGenet
507 7	Vitamin K Epoxide Reductase Complex Subunit 1	VKORC1		GeneCard	DisGenet
507 8	Very Low Density Lipoprotein Receptor	VLDLR		GeneCard	DisGenet
507 9	Vanin 1	VNN1			DisGenet
508 0	Vanin 3, Pseudogene	VNN3P		GeneCard	
508 1	VPS33A Core Subunit Of CORVET And HOPS Complexes	VPS33A		GeneCard	
508 2	VPS41 Subunit Of HOPS Complex	VPS41		GeneCard	
508 3	VPS51 Subunit of GARP Complex	VPS51			DisGenet
508 4	V-Set and Immunoglobulin Domain Containing 4	VSIG4		GeneCard	

508<br>5	V-Set Immunoregulatory Receptor	VSIR		GeneCard	
508<br>6	V-Set and Transmembrane Domain Containing 4	VSTM4		GeneCard	
508<br>7	Visual System Homeobox 1	VSX1		GeneCard	
508<br>8	Vitronectin	VTN		GeneCard	DisGenet
508<br>9	Coagulation factor VIII VWF (von Willebrand factor)	VWF	OMIM	GeneCard	DisGenet
509<br>0	WAPL Cohesin Release Factor	WAPL		GeneCard	
509<br>1	WDFY Family Member 4	WDFY4		GeneCard	
509<br>2	WD Repeat and HMG-box DNA Binding Protein 1	WDHD1			DisGenet
509<br>3	WD Repeat Domain 1	WDR1		GeneCard	DisGenet
509<br>4	WD Repeat Domain 11	WDR11		GeneCard	
509<br>5	WD Repeat Domain 20	WDR20			DisGenet
509<br>6	WD Repeat Domain 33	WDR33		GeneCard	
509<br>7	WEE2 Antisense RNA 1	WEE2-AS1		GeneCard	
509<br>8	WAP Four-Disulfide Core Domain 21, Pseudogene	WFDC21P		GeneCard	
509<br>9	WNK Lysine Deficient Protein Kinase 1	WNK1		GeneCard	DisGenet
510<br>0	Wnt Family Member 1	WNT1		GeneCard	
510<br>1	Wnt Family Member 2	WNT2		GeneCard	
510<br>2	Wnt Family Member 3A	WNT3A			DisGenet
510<br>3	Wnt Family Member 4	WNT4		GeneCard	
510<br>4	Wnt Family Member 5A	WNT5A		GeneCard	DisGenet

510<br>5	WRN RecQ Like Helicase	WRN		GeneCard	DisGenet
510<br>6	WRN Helicase Interacting Protein 1	WRNIP1		GeneCard	
510<br>7	WT1 Transcription Factor	WT1		GeneCard	
510<br>8	WW And C2 Domain Containing 1	WWC1		GeneCard	
510<br>9	WW Domain Containing Oxidoreductase	WWOX		GeneCard	
511<br>0	WW Domain Containing E3 Ubiquitin Protein Ligase 1	WWP1		GeneCard	
511<br>1	WW Domain Containing E3 Ubiquitin Protein Ligase 2	WWP2		GeneCard	
511<br>2	WW Domain Containing Transcription Regulator 1	WWTR1		GeneCard	DisGenet
511<br>3	XPA Binding Protein 2	XAB2		GeneCard	
511<br>4	X-Box Binding Protein 1	XBP1		GeneCard	DisGenet
511<br>5	X-C Motif Chemokine Ligand 1	XCL1		GeneCard	
511<br>6	X-C Motif Chemokine Ligand 2	XCL2		GeneCard	
511<br>7	Xanthine Dehydrogenase	XDH		GeneCard	DisGenet
511<br>8	X-Linked Inhibitor of Apoptosis	XIAP		GeneCard	DisGenet
511<br>9	X Inactive Specific Transcript	XIST		GeneCard	DisGenet
512<br>0	X-Linked Kx Blood Group	XK		GeneCard	
512<br>1	XK Related 4	XKR4		GeneCard	
512<br>2	XPA, DNA Damage Recognition and Repair Factor	XPA		GeneCard	
512<br>3	XPC Complex Subunit, DNA Damage Recognition and Repair Factor	XPC		GeneCard	
512<br>4	X-Prolyl Aminopeptidase 2	XPNPEP2		GeneCard	

5125	Xenotropic and Polytropic Retrovirus Receptor 1	XPR1			DisGenet
5126	X-Ray Repair Cross Complementing 1	XRCC1		GeneCard	
5127	X-Ray Repair Cross Complementing 3	XRCC3		GeneCard	
5128	X-Ray Repair Cross Complementing 5	XRCC5		GeneCard	
5129	X-Ray Repair Cross Complementing 6	XRCC6		GeneCard	
5130	XXYLT1 Antisense RNA 2	XXYLT1-AS2		GeneCard	
5131	Xylosyltransferase 1	XYLT1		GeneCard	
5132	Xylosyltransferase 2	XYLT2		GeneCard	
5133	Yes1 Associated Transcriptional Regulator	YAP1		GeneCard	DisGenet
5134	Y-Box Binding Protein 1	YBX1		GeneCard	DisGenet
5135	Tyrosine 3-Monooxygenase/Tryptophan 5-Monooxygenase Activation Protein Epsilon	YWHAE		GeneCard	
5136	Tyrosine 3-Monooxygenase/Tryptophan 5-Monooxygenase Activation Protein Zeta	YWHAZ		GeneCard	DisGenet
5137	YY1 Transcription Factor	YY1		GeneCard	
5138	Zeta Chain of T Cell Receptor Associated Protein Kinase 70	ZAP70		GeneCard	
5139	Zinc Finger and BTB Domain Containing 16	ZBTB16		GeneCard	
5140	Zinc Finger and BTB Domain Containing 17	ZBTB17		GeneCard	
5141	Zinc Finger and BTB Domain Containing 20	ZBTB20		GeneCard	
5142	Zinc Finger and BTB Domain Containing 46	ZBTB46		GeneCard	DisGenet
5143	Zinc Finger CCCH-Type Containing 12A	ZC3H12A		GeneCard	
5144	Zinc Finger CCCH-Type Containing 12C	ZC3H12C		GeneCard	

5145	Zinc Finger C3HC-Type Containing 1	ZC3HC1		GeneCard	DisGenet
5146	Zinc Finger C4H2-Type Containing 2	ZC4H2			DisGenet
5147	ZDHHC8 Pseudogene 1	ZDHHC8P1		GeneCard	
5148	Zinc Finger E-box Binding Homeobox 1	ZEB1			DisGenet
5149	ZEB1 Antisense RNA 1	ZEB1-AS1		GeneCard	DisGenet
5150	Zinc Finger E-Box Binding Homeobox 2	ZEB2		GeneCard	
5151	ZNFX1 Antisense RNA 1	ZFAS1		GeneCard	
5152	Zinc Finger Homeobox 3	ZFHX3		GeneCard	
5153	ZFP36 Ring Finger Protein	ZFP36		GeneCard	DisGenet
5154	Zinc Finger Protein, FOG Family Member 2	ZFPM2		GeneCard	
5155	Zinc Finger FYVE-Type Containing 9	ZFYVE9		GeneCard	
5156	Zinc Finger GATA Like Protein 1	ZGLP1			DisGenet
5157	Zinc Finger GRF-Type Containing 1	ZGRF1		GeneCard	
5158	Zinc Fingers And Homeoboxes 2	ZHX2		GeneCard	DisGenet
5159	Zinc Metallopeptidase STE24	ZMPSTE24		GeneCard	DisGenet
5160	Zinc Finger Protein 148	ZNF148			DisGenet
5161	Zinc Finger Protein 202	ZNF202		GeneCard	DisGenet
5162	Zinc Finger Protein 213	ZNF213		GeneCard	
5163	ZNF213 Antisense RNA 1 (Head To Head)	ZNF213-AS1		GeneCard	
5164	Zinc Finger Protein 23	ZNF23		GeneCard	

5165	Zinc Finger Protein 248	ZNF248		GeneCard	
5166	Zinc Finger Protein 263	ZNF263			DisGenet
5167	ZNF295 Antisense RNA 1	ZNF295-AS1		GeneCard	
5168	Zinc Finger Protein 318	ZNF318			DisGenet
5169	Zinc Finger Protein 326	ZNF326		GeneCard	
5170	Zinc Finger Protein 366	ZNF366		GeneCard	
5171	Zinc Finger Protein 383	ZNF383		GeneCard	
5172	Zinc Finger Protein 385D	ZNF385D		GeneCard	
5173	Zinc Finger Protein 394	ZNF394		GeneCard	
5174	Zinc Finger Protein 441	ZNF441		GeneCard	
5175	Zinc Finger Protein 462	ZNF462		GeneCard	
5176	Zinc Finger Protein 491	ZNF491		GeneCard	
5177	Zinc Finger Protein 580	ZNF580		GeneCard	
5178	Zinc Finger Protein 592	ZNF592		GeneCard	
5179	Zinc Finger Protein 627	ZNF627		GeneCard	
5180	Zinc Finger Protein 687	ZNF687		GeneCard	
5181	Zinc Finger Protein 717	ZNF717		GeneCard	
5182	Zinc Finger Family Member 788, Pseudogene	ZNF788P		GeneCard	
5183	Zinc Finger Protein 823	ZNF823		GeneCard	
5184	Zinc Finger Protein 844	ZNF844		GeneCard	

518<br>5	Zinc Finger Protein 887, Pseudogene	ZNF887P		GeneCard	
518<br>6	Zinc Finger HIT-Type Containing 3	ZNHIT3		GeneCard	
518<br>7	Zinc Ribbon Domain Containing 2	ZNRD2			DisGenet
518<br>8	Zona Pellucida Glycoprotein 2	ZP2		GeneCard	
518<br>9	Zona Pellucida Glycoprotein 3	ZP3		GeneCard	
519<br>0	ZPR1 Zinc Finger	ZPR1		GeneCard	
519<br>1	Zinc Finger RANBP2-Type Containing 3	ZRANB3		GeneCard	
519<br>2	Zyxin	ZYX		GeneCard	

Supplementary Table S4: The degree value of nodes in protein–protein interaction network.

No	Target	Degree
1.	SRC	41
2.	PIK3R1	29
3.	PIK3CA	29
4.	AKT1	29
5.	RXRA	23
6.	PTPN11	21
7.	EGFR	20
8.	ESR1	20
9.	NFKB1	17
10.	APP	14
11.	MAPK8	14
12.	STAT1	13
13.	CYP3A4	13
14.	RXRB	13
15.	AR	13
16.	CDK1	13
17.	PTK2	12
18.	SYK	12
19.	PRKCA	12
20.	PDGFRB	12
21.	IGF1R	11
22.	PTPN1	11
23.	PIK3CB	11
24.	CDK5	10
25.	KDR	10
26.	PTPN6	10
27.	GSK3B	10
28.	CDK6	10
29.	NR3C1	10
30.	RAF1	9
31.	CCNA2	9
32.	PTPN2	9
33.	F2	9
34.	CCNB1	9
35.	CYP2C19	9
36.	PRKCD	9
37.	CDK2	9
38.	INSR	8
39.	PPARG	8
40.	MET	8
41.	PPARA	8
42.	CCNB2	7

43.	PLG	7
44.	PSEN1	7
45.	TERT	7
46.	CCNE1	7
47.	HIF1A	7
48.	PIK3CG	7
49.	FLT1	7
50.	ALOX5	7
51.	NOS2	7
52.	ALOX15	6
53.	PTGS2	6
54.	ROCK2	6
55.	NCSTN	6
56.	ROCK1	6
57.	PIN1	6
58.	MAPT	6
59.	NR1H3	6
60.	CAMK2B	6
61.	KIT	6
62.	BCL2	6
63.	AURKA	6
64.	TLR4	6
65.	PTGS1	6
66.	HDAC3	5
67.	SLC9A1	5
68.	ALOX12	5
69.	PDGFRA	5
70.	HMGCR	5
71.	PIK3CD	5
72.	PARP1	5
73.	NR1H2	5
74.	CBR1	5
75.	CAPN1	5
76.	CCNT1	5
77.	AHR	5
78.	APEX1	4
79.	KDM1A	4
80.	EDNRA	4
81.	NR4A1	4
82.	APH1B	4
83.	TRIM24	4
84.	NTRK1	4
85.	MMP9	4
86.	NOS1	4
87.	HSD11B1	4

88.	CETP	4
89.	MAOB	4
90.	BACE1	4
91.	AKR1B10	4
92.	SCD	4
93.	AKR1B1	4
94.	ADAM10	4
95.	AKR1A1	3
96.	DPP4	3
97.	ESR2	3
98.	DNMT1	3
99.	PLA2G4A	3
100.	CES1	3
101.	GUSB	3
102.	PGR	3
103.	PTGES	3
104.	KEAP1	3
105.	ARG1	3
106.	EPHX1	3
107.	MGLL	3
108.	MAPK9	3
109.	PSEN2	3
110.	PSMB9	3
111.	DHFR	3
112.	CYP19A1	3
113.	PPARD	3
114.	FPR1	3
115.	MMP3	3
116.	MAOA	3
117.	LIMK1	2
118.	CFTR	2
119.	POLB	2
120.	NFE2L2	2
121.	ESRRA	2
122.	CA9	2
123.	PKN1	2
124.	CPT2	2
125.	CES2	2
126.	EPAS1	2
127.	MPO	2
128.	SERPINE1	2
129.	FABP4	2
130.	CYP17A1	2
131.	NOX4	2
132.	MYLK	2

133.	ABCB1	2
134.	S1PR3	2
135.	AOC3	2
136.	PTGER1	2
137.	ODC1	2
138.	MMP2	2
139.	NR1I2	2
140.	PDE3B	2
141.	SQLE	2
142.	CYP1B1	2
143.	CYP51A1	2
144.	C5AR1	1
145.	NLRP3	1
146.	KCNH2	1
147.	MMP13	1
148.	ALK	1
149.	ACHE	1
150.	FABP5	1
151.	F2R	1
152.	PRMT1	1
153.	PIM1	1
154.	AXL	1
155.	MMP8	1
156.	MAPKAPK2	1
157.	MMP12	1
158.	SHBG	1
159.	AKR1C4	1
160.	PRKCH	1
161.	MAP4K4	1
162.	AHCY	1
163.	TTR	1
164.	F10	1
165.	S1PR2	1
166.	CD81	1
167.	GGPS1	1
168.	TOP1	1
169.	SLC5A2	1
170.	RPS6KA5	1
171.	CA2	1
172.	KLF5	1
173.	PTPRC	1
174.	TNKS	1
175.	CTSD	1
176.	FPR2	1
